# Water-Soluble Heptacyclic Oligo-Heteroaryls Targeting G‑Quadruplex DNA via Groove-Binding Interactions

**DOI:** 10.1021/acsomega.6c03686

**Published:** 2026-07-18

**Authors:** Giorgia Fracchioni, Alessio M. Caramiello, Matteo Nadai, Alberto Lena, Mauro Freccero, Sara N. Richter, Valentina Pirota, Filippo Doria

**Affiliations:** † Department of Chemistry, 19001 University of Pavia, Pavia 27100, Italy; ‡ Department of Molecular Medicine, University of Padova, Padova 35131, Italy; § Microbiology and Virology Unit, Padua University Hospital, Padua 35128, Italy; ∥ G4-INTERACT, USERN, Pavia 27100, Italy

## Abstract

G-quadruplexes (G4s) are noncanonical nucleic acid structures involved in the regulation of key biological processes and represent attractive targets for therapeutic intervention. In this study, we report the design, synthesis, and biophysical evaluation of a new family of water-soluble heptacyclic oligo-heteroaryl ligands tailored for G4 recognition. The synthetic route, based on a modular and convergent approach, enables late-stage functionalization and overcomes common limitations of macrocyclic scaffolds such as low yields and poor tractability. The new compounds incorporate 1,2,4-oxadiazole and 1,2,3-triazole units as bioisosteric replacements for oxazole and systematically vary the nature of the central atoms (C vs N) and pendant groups to investigate their effect on G4 binding. All derivatives displayed high aqueous solubility under physiological conditions and selective stabilization of G4 structures, particularly the parallel LTR-IV motif. Biophysical studies and docking calculations indicate that these ligands operate primarily through groove-binding interactions. Notably, no substantial differences were observed between nitrogen- and carbon-substituted analogues, suggesting a conserved binding profile across the series. These findings identify a versatile class of G4 binders with favorable solubility and structural tunability, laying the groundwork for the development of functionalized derivatives for chemical biology and therapeutic applications.

## Graphical abstract

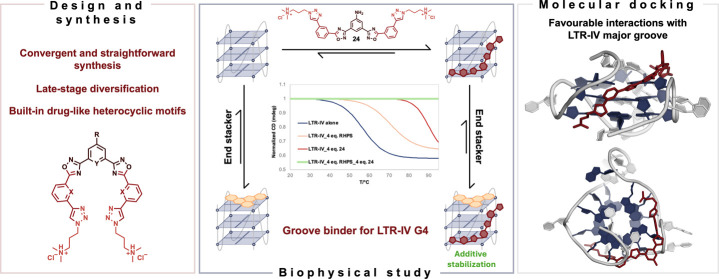

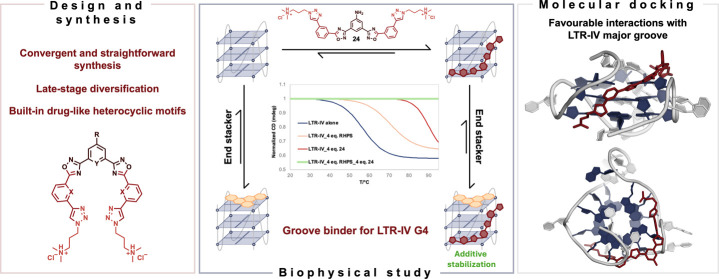

## Introduction

G-quadruplexes (G4s) are noncanonical secondary structures that form in guanine-rich regions of DNA and RNA. In these sequences, four guanine bases associate via Hoogsteen hydrogen bonds to form a planar tetrad known as a G-quartet, stabilized by monovalent cations, particularly potassium. Stacking of multiple G-tetrads on top of each other gives rise to the secondary structure known as G4. In the human genome, G4-forming sequences are broadly distributed and enriched in functionally relevant regions such as promoters and telomeres.^1–3^ Rather than acting as mere physical obstacles to transcription or replication, G4s are now increasingly recognized as dynamic molecular switches capable of modulating nucleic acid transactions and regulating gene expression in a context-dependent manner.^4^ These structures have been identified as key regulators of many biological and pathological processes in humans,^5,6^ such as cancer,^7,8^ neurodegenerative disorders,^9,10^ and infectious diseases caused by parasites,^11–13^ bacteria,^14–16^ and viruses.^17^

In this scenario, small molecules capable of selectively interacting with G4s have emerged as powerful tools to modulate their folding, stabilization, and functional outputs. Over the years, many high-affinity ligands have been developed and characterized.^2,18,19^ However, structural data consistently show that most G4 binders still rely on the same dominant recognition mode based on π-stacking at one or both terminal G-tetrads.^20^ While this end-stacking strategy is associated with high affinity, it often provides limited selectivity due to the structural similarity of exposed quartets across different G-quadruplexes and, in some cases, with other nucleic acid architectures. Consequently, end-stacking ligands frequently display limited discrimination among distinct G-quadruplex folds and insufficient selectivity over duplex DNA. To overcome this limitation, alternative design strategies have focused on expanding ligand recognition beyond the G-tetrad plane by exploiting grooves, loops, and higher-order features to introduce topology-biased or organism-biased discrimination.

Groove binders have emerged as promising scaffolds combining improved selectivity with more favorable drug-likeness, as they exploit the structurally diverse and topology-dependent grooves and loop regions of G4s rather than the highly conserved terminal G-quartets. By targeting variable groove widths and distinct patterns of hydrogen-bonding and electrostatic features, this binding mode enables enhanced molecular discrimination among different G4 conformations while inherently reducing off-target interactions with duplex DNA, whose grooves present a markedly different chemical and geometrical environment.^21^ However, despite their enhanced specificity, most groove-targeting ligands still display low affinity and modest stabilizing power, highlighting a persistent unmet need in the field: achieving high specificity without sacrificing binding potency.^22–31^

Following this rationale, recent efforts have shifted toward nonmacrocyclic heptacyclic scaffolds with enhanced conformational flexibility and modular architectures, enabling alternative binding modes on G4s. Inspired by a natural macrocyclic compound such as Telomestatin, Teulade-Fichou and co-workers introduced neutral, acyclic oligomeric scaffolds composed of alternating oxazole and pyridine units, providing one of the first examples explicitly conceived to explore noncanonical recognition geometries while retaining significant G4 binding affinity.^32–34^ Systematic structure–activity relationships highlighted that heterocycle identity and side-chain functionalization critically influence the balance between π-stacking contribution and groove-mediated interactions. Shorter pentacyclic derivatives, like BOxaPy,^33^ showed negligible affinity unless supplemented with cationic side chains, while replacing oxazole units with 1,2,4-oxadiazoles (in BOxAzaPy^33^ or ToxAzaPy^34^) improved both binding and G4 stabilization by alleviating steric constraints and increasing flexibility. G4-FID (Fluorescence Intercalator Displacement) and FRET melting assays from these works indicated a binding mode distinct from end-stacking that is consistent with engagement of grooves or loops, an interpretation further supported by the lack of significant binding to duplex DNA under competitive conditions for the most extended hepta-aryl analogues.^32,34^

In parallel, fragment-based approaches have underlined the importance of specific functional motifs, such as amidoxime derivatives, in establishing initial contacts with G4 scaffolds and guiding elaboration toward higher affinity and selectivity, thereby reinforcing the concept that nonplanar, modular heteroaryl frameworks can effectively explore underexploited structural features of G4 grooves.^21,35^

Taken together, these lines of evidence suggest that open heteroaryl ligands represent a strategic choice for several reasons: (1) they allow the synthetic route to bypass the typically low-yielding final macrocyclization step, (2) they enable the use of a symmetrical synthesis, avoiding protection/deprotection strategies, and (3) their flexibility may favor interactions within the grooves of G4 structures, potentially supporting noncanonical binding modes.

Inspired by these findings, this work investigates the still unexplored field of oligo-heteroaromatic ligands by extending the existing studies with a new family of water-soluble heptacyclic compounds. Our proposed heptacyclic scaffold (Figure 1, bottom) retains the same structural features of Telomestatin and Telomestatin-like compounds (Figure 1, top), such as the presence of nitrogen- and oxygen-containing five-membered rings, full conjugation provided by the aromatic systems, and the number of linked cycles. The structural modifications that we hypothesized are aimed at improving water solubility and binding affinity. To this end, the oxazole rings have been substituted with oxadiazoles and triazoles, the latter being implemented to exploit CuAAC chemistry and easily diversify the structures. Moreover, both oxadiazoles^36^ and triazoles^37,38^ are well-known pharmacophores in medicinal chemistry for their thermal and chemical stability in water.^39^ Furthermore, we envisioned the substitution of the central atoms (X and Y, Figure 1, bottom) on the six-membered rings to investigate whether this replacement would allow for a better recognition of the G4 structure. While nitrogen atoms can modulate electronic density and hydrogen-bonding potential, potentially enhancing interactions within the grooves of G-quadruplexes, the introduction of carbon atoms, being less polar, allows for the investigation of the impact of reduced polarity and altered steric profiles on the G4 affinity and selectivity. Finally, we planned the modification of the R group with a dual purpose: first, to evaluate whether the introduction of electron-withdrawing or electron-donating substituents on the central aromatic core influences G4 binding affinity and selectivity; and second, to enable future conjugation with functional moieties, such as a ligand or a substrate selectively targeting a DNA structure or a protein, a fluorophore, biotin, a radionuclide, an alkylating agent, a secondary recognition unit, for imaging or affinity-based applications.

**Figure 1:**
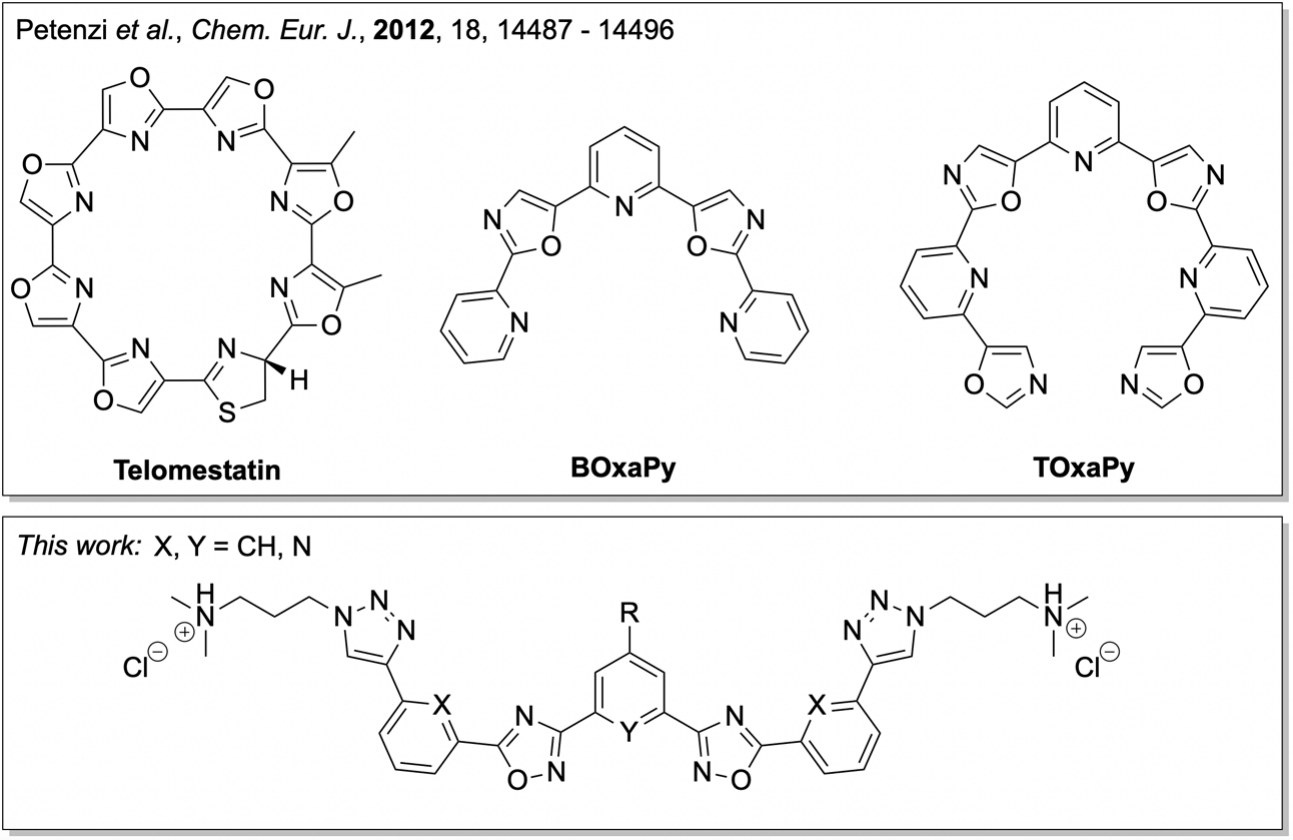
Previous work featuring Telomestatin and the first ideas of open heteroaryls (top), and chemical scaffold proposed in this paper (bottom).

We will discuss the synthesis and characterization of this novel class of compounds. To comprehensively evaluate the interaction of the newly synthesized heptacyclic ligands with DNA G4s, biophysical techniques were employed, not only to assess their stabilizing effects but also, more importantly, to elucidate their binding mode. G4-FID assays, circular dichroism (CD), CD melting experiments, and docking studies were carried out to confirm groove binding as the predominant interaction mechanism.

## Results and Discussion

### Synthesis of Water-Soluble Heptacyclic Ligands

To enable downstream functionalization and structural diversification, the development of a robust and flexible synthetic route was prioritized. There are different methods in the literature for the synthesis of substituted oxadiazoles. All of them share the low reaction yields typically observed in the synthesis of bifunctional oligoaryl systems, mainly due to the harsh conditions needed for the ring closure step.^40–42^ By analogy with the alternating 6- and 5-membered rings oligomeric scaffolds already described, an internal plane of symmetry was maintained to simplify the overall synthetic process.

To overcome the limitations associated with traditional approaches, we undertook a new retrosynthetic analysis aimed at designing a more-convergent and efficient synthetic route. This retrosynthetic approach (Scheme 1) depicts a convergent synthesis toward the first target molecule **8**. As reported in the literature,^43^ 1,2,4-oxadiazoles can be obtained through the condensation of a carboxylic acid and an amidoxime followed by intramolecular cyclodehydration. Amidoxime **2** could be preventively synthesized from nitrile **1**, while acid **7** was derived from the CuAAC reaction between azide **4** and alkyne **6** (both resulting from the derivatization of commercially available substrates).

**Scheme 1:**
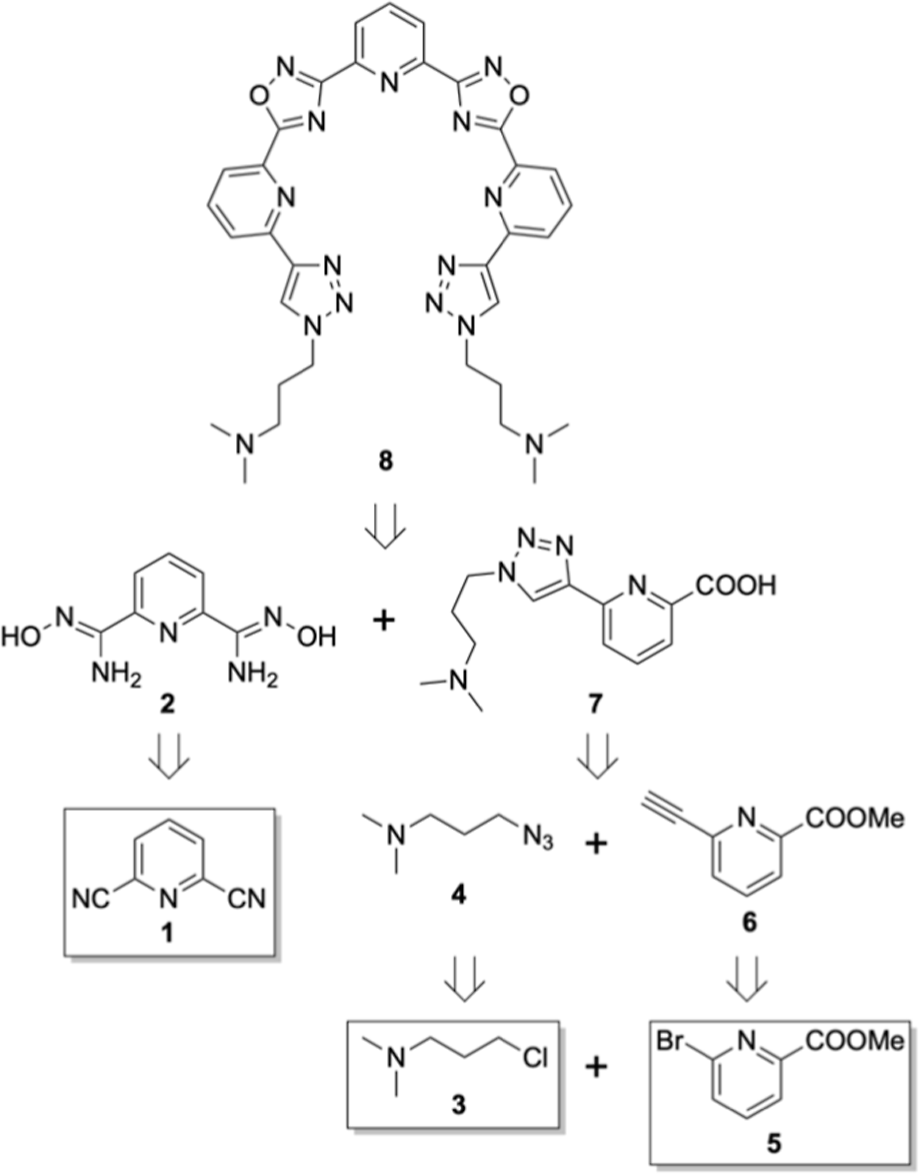
Retrosynthetic Analysis of the Heptacyclic Scaffold **8**

Diamidoxime **2** was obtained from pyridine-2,6-dicarbonitrile **1** upon treatment with hydroxylamine in aqueous ethanol. Concurrently, **3** was converted to azide **4**, while the Sonogashira coupling between picolinate **5** and trimethylsilylacetylene and subsequent TMS methanolysis^44^ smoothly allowed the introduction of a terminal alkyne at the right position. The key CuAAC between **4** and **6** cleanly afforded methyl ester **6a**, then basic hydrolysis and acidic workup yielded molecule **7** as a trifluoroacetate salt after preparative HPLC purification. Finally, carboxylic acid **7**, after CDI-mediated activation, was condensed with diamidoxime **2**, and base-promoted cyclodehydration resulted in the synthesis of compound **8** (Scheme 2).

**Scheme 2:**
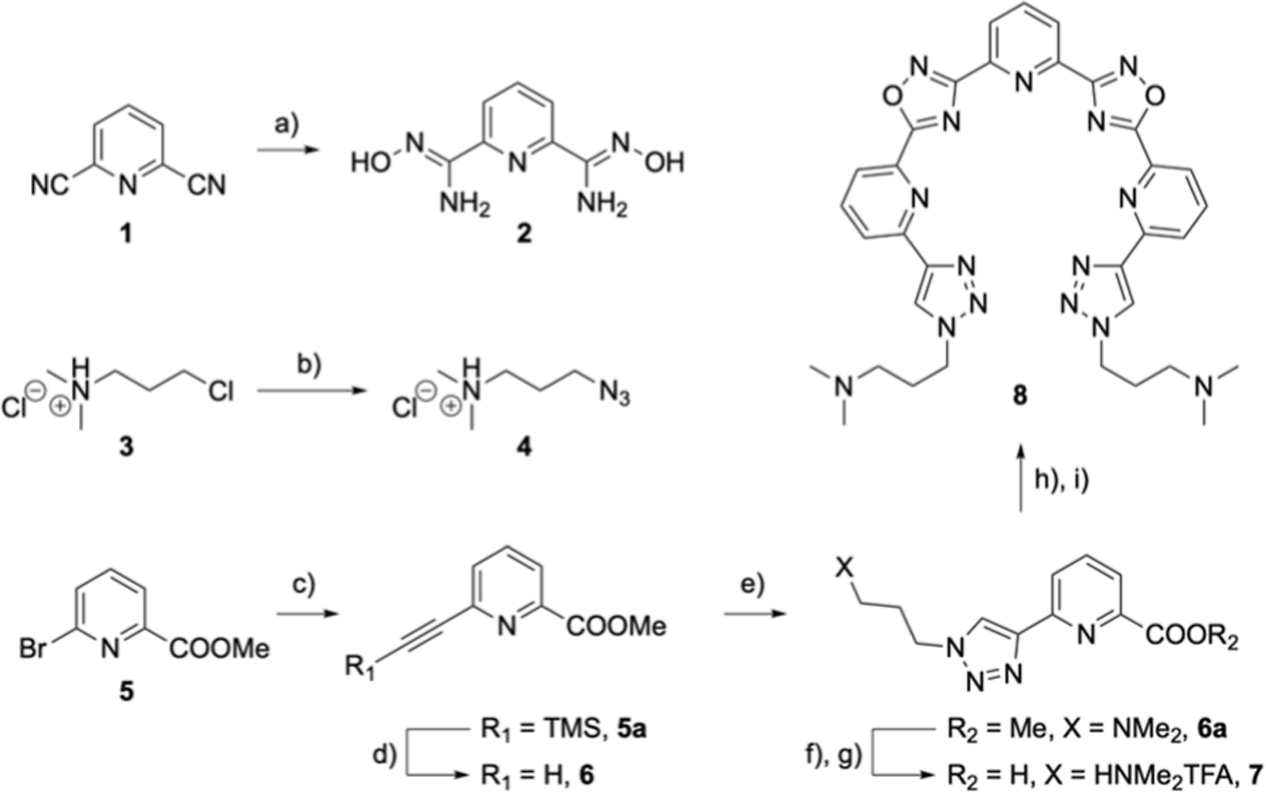
Synthesis of Water-Soluble Heptacyclic Oligo-Heteroaryl **8**. (a) NH_2_OH·HCl, Na_2_CO_3_, EtOH/H_2_O, rt, 16 h, 78%; (b) NaN_3_, H_2_O, 80 °C, 16 h, 91%; (c) Pd­(PPh_3_)_2_Cl_2_ 2%, PPh_3_ 4%, CuI 2%, iPr_2_NH, TMS-CCH, PhCH_3_, 80 °C, 1 h, 92%; (d) K_2_CO_3_, MeOH, rt, 2 h, 87%; (e) **4·HCl**, CuSO_4_·5H_2_O, Sodium Ascorbate, H_2_O/*t*-BuOH, rt, 24 h, 90%; (f) K_2_CO_3_, THF/H_2_O, Reflux, 2 h, Then (g) HCl, H_2_O, 98% (over 2 Steps); (h) CDI, **2**, DMF, rt, 16 h, Then (i) K_2_CO_3_, DMF, 60 °C, 2 h, 34% (over 2 Steps)

The synthetic process proposed benefited from high flexibility and convergence; in a similar fashion, other derivatives were obtained. The difference from scaffold **8** is the modification of the pyridine rings with simple benzene moieties to obtain the corresponding carbon analogues.

The carbon analogue building blocks (Scheme 3) were synthesized following the same synthetic scheme with some adaptations for the reaction conditions, starting from commercially available isophthalonitrile **9** and aryl bromide **11**.

**Scheme 3:**
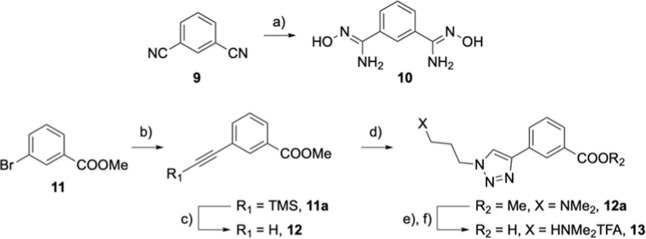
Synthesis of Carbon Analogue Building Blocks **10** and **13**. (a) NH_2_OH·HCl, Na_2_CO_3_, EtOH/H_2_O, Reflux, 16 h, 78%; (b) Pd­(PPh_3_)_2_Cl_2_ 2%, CuI 1%, Et_3_N, TMS-CCH, ACN, 50 °C, 24 h, 88%; (c) K_2_CO_3_, MeOH, rt, 2 h, 86%; (d) 4·HCl, CuSO_4_·5H_2_O, Sodium Ascorbate, H_2_O/*t*-BuOH, rt, 24 h, 95%; (e) K_2_CO_3_, THF/H_2_O, Reflux, 24 h, Then (f) HCl, H_2_O, 97% (over 2 Steps)

The synthesis of the three-carbon analogues **14–16** was achieved with the right combination of building blocks (Scheme 4). The substitution of pyridine with benzene had some impact on the cyclodehydration step. When X = N, the reaction proceeds smoothly, whereas if X = CH, longer reaction times are needed. This happens because benzene (X = CH) lowers the electrophilicity of the carboxylate, which then translates to lower reactivity. The worst combination of diamidoxime **2** and acid **13** led to at least a 50-fold decrease in the reactivity of the base-promoted cyclodehydration.

**Scheme 4:**
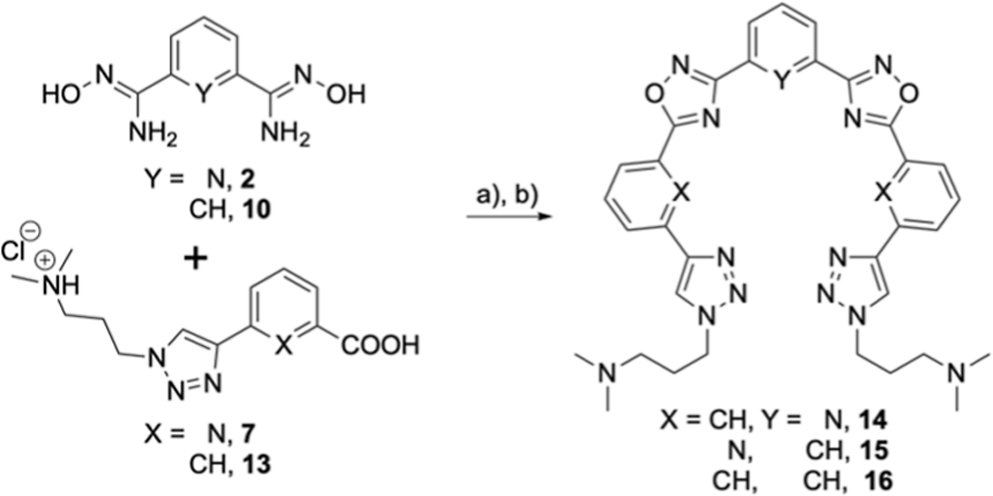
Synthesis of Heptacyclic Carbon Analogues **14–16**. (a) CDI, DMF, rt, 16 h and (b) K_2_CO_3_, DMF, 60 °C, 2 h – 4 d, 30–40% (over 2 Steps)

Another synthetic approach based on the already presented one was designed to modify the heptacyclic compounds to adapt them for conjugation with other functional groups. By adaptation of a known procedure,^45^ dimethyl-5-nitroisophthalate **17** was converted into isophthalonitrile **19** by amidation with ammonia and subsequent dehydration of intermediate diamide **18** with trifluoroacetic anhydride. The addition of hydroxylamine was effective in affording the amidoxime **20** (Scheme 5). The synthesis of nitro-substituted heptacycles **21** and **22** was achieved under the same reaction conditions described above for the simpler derivatives. Eventually, the nitroaryl was effectively reduced with SnCl_2_ to yield the amino-substituted heptacyclic oligoheteroaryls **23** and **24** (Scheme 5).

**Scheme 5:**
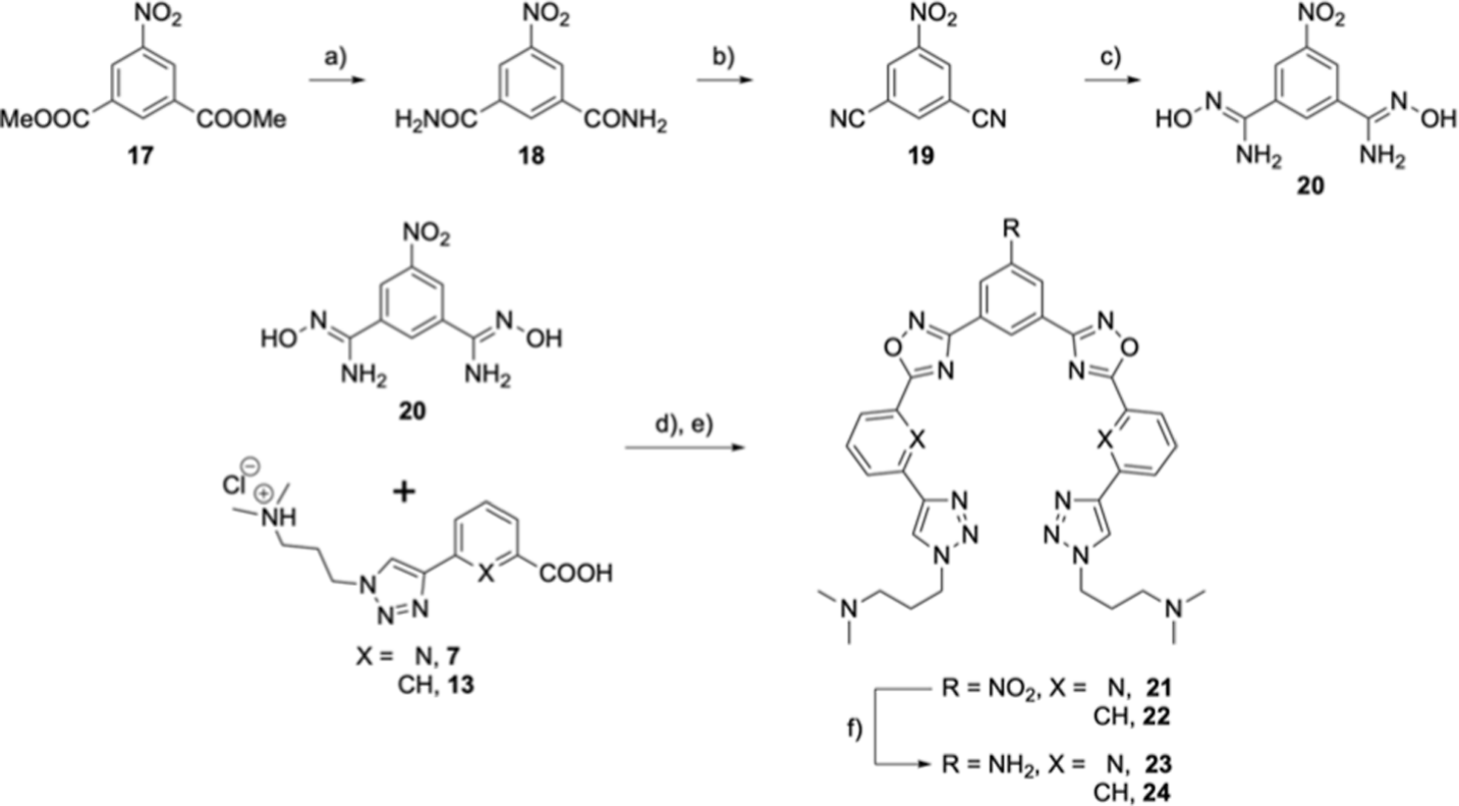
Synthesis of Substituted Heptacyclic Oligo-Heteroaryls **21–24**. (a) NH_3_, MeOH, rt, 16 h, 94%; (b) TFAA, Et_3_N, THF, rt, 3 h, 87%; (c) NH_2_OH·HCl, Na_2_CO_3_, EtOH/H_2_O, rt, 16 h, 86%; (d) CDI, DMF, rt, 16 h Then (e) K_2_CO_3_, DMF, 60 °C, 2 h – 4 d, 30–45% (over 2 Steps); and (f) SnCl_2_, HCl, EtOH, Reflux, 6 h, 82–86%

Remarkably, all the heptacyclic oligo-heteroaryls presented here (**8**, **14**–**16**, **21**–**24**) exhibited high values of water solubility under physiological conditions (ca. 10^–2^ M) due to the presence of the two tertiary amine side chains. This feature differentiated them from all the known nonmacrocyclic oligo-heteroaromatic G4 ligands, translating into a fundamental advantage in terms of drug bioavailability and permeability toward cellular and nuclear membranes. To experimentally assess the aqueous behavior of the heptacyclic ligands, UV–vis absorption measurements were performed at increasing ligand concentrations under physiological-like conditions. In all the cases, the absorbance increased linearly with concentration over the investigated range, without deviations from the Beer–Lambert behavior, spectral distortion, baseline scattering, or precipitation phenomena (Figures S1 and S2). The absence of nonlinearity or light-scattering effects suggests that the compounds remain molecularly dispersed in solution under the experimental conditions employed. These measurements support the high apparent aqueous solubility and operational stability of the ligands at concentrations relevant for the subsequent biophysical assays.

### Biophysical Binding Assays and Molecular Docking Studies toward G4 Structures

To enable a structurally informed evaluation of ligand binding, the G4 panel was selected to encompass a diverse range of topologies, loop architectures, and groove accessibility (Table S1). Both hybrid and parallel G4s were included to probe how structural features influence ligand recognition. Hybrid structures such as hTel (hybrid-1) and LTR-III (hybrid-2) are characterized by lateral and diagonal loops that partially occlude the grooves and generate heterogeneous binding environments, thus representing challenging systems for groove-targeting ligands. In contrast, parallel G4s, including c-myc, LTR-IV, hTert, and Pu24T, adopt propeller-type loop arrangements that leave the grooves more exposed and geometrically regular, features generally considered favorable for groove recognition. Within this group, Pu24T serves as a well-established, highly stable model parallel G4, while hTert and c-myc provide biologically relevant promoter sequences with distinct sequence compositions and groove geometries. LTR-IV, of viral origin, represents a structurally and biologically distinct target, enabling evaluation of ligand behavior beyond canonical human G4s. Finally, duplex DNA sequences (ds12 and ds26), hairpin, and scrambled sequences were included as controls to assess G4 preference over other nucleic acid structures. This panel, therefore, provides a structurally diverse framework to investigate how groove accessibility, loop arrangement, and topology influence ligand binding and stabilization.

The interaction of the heptacyclic ligands with DNA G4s was initially investigated using G4-FID assays. The ability of each compound to displace thiazole orange (TO), a fluorescent dye that binds preferentially to the terminal G-quartets of G4 structures, was assessed across the panel of G4-forming sequences (Figure 2; Table S1) against the ds26 sequence. Given the π–π stacking nature of TO binding, minimal displacement is typically expected for groove-binding ligands. Consistent with this expectation, most tested compounds showed poor TO displacement, even at high molar excess (up to 20 equiv). Remarkably, except for **21**, all ligands induced only low to moderate TO displacement, with most curves plateauing below 40% displacement, even at high ligand excess (Figure 2a). This behavior was in sharp contrast with the nearly quantitative displacement observed for effective end-stackers, such as the tetrasubstituted NDI derivative **NDIpip** ^46^ (Figure 2b), which displaced TO almost completely at just two equivalents.

**Figure 2:**
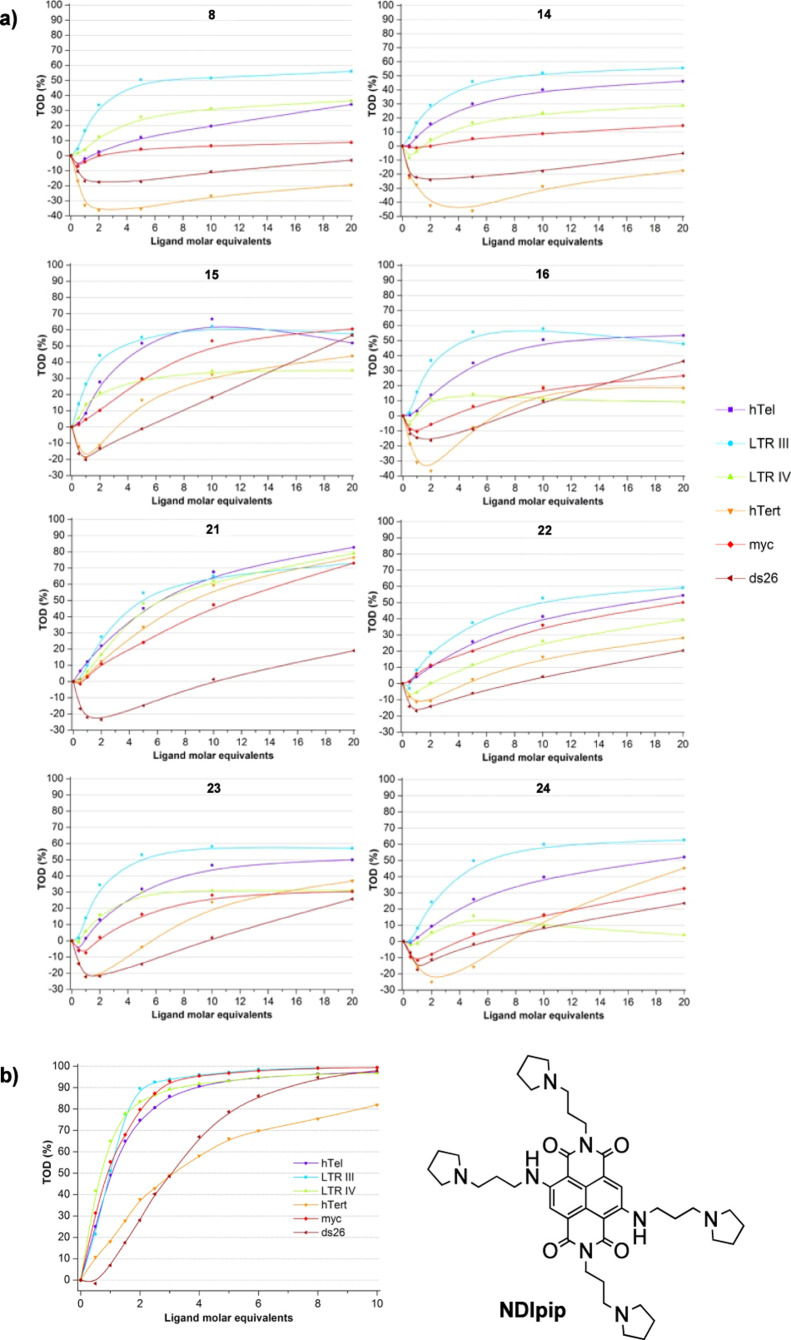
(a) G4-FID titration assay of heptacyclic ligands **8**, **14**–**16**, and **22**–**24** with five selected G4s (hTel, LTR-III, LTR-IV, hTert, and myc) and one dsDNA control sequence (ds26). Conditions: 1 μM DNA, 2 μM TO, 0–20 μM compounds, 100 mM KCl, in 10 mM lithium cacodylate buffer (pH 7.2). (b) G4-FID titration assay of the tetrasubstituted NDI derivative **NDIpip**, used as a reference end-stacking ligand, and its chemical structure.

Notably, this trend was particularly evident for the parallel myc and LTR-IV G4s, which are characterized by highly exposed terminal G-tetrads and therefore represent the most favorable substrates for end-stacking interactions. Rather than showing enhanced TO displacement, these sequences exhibited the lowest FID responses, frequently displaying negligible or even null displacement across a broad range of ligand equivalents. The persistence of TO binding in these systems, despite optimal tetrad accessibility, provides strong experimental evidence against a predominant terminal stacking mode. Instead, the FID profiles strongly support a binding mechanism dominated by groove recognition, in which ligand-G4 interactions are governed by groove topology and electrostatic complementarity rather than by π–π stacking on terminal G-quartets.

Particularly, ligands **8** and **14** emerged most effective for the myc G4, displaying only ∼10% TO displacement at 20 equiv. These two compounds also showed negligible displacement of TO from the duplex control ds26, indicating high selectivity for G4s. Similarly, ligands **16** and **24** were the top performers on LTR-IV, also showing very low TO displacement (∼10%) under the same conditions.

Conversely, LTR-III and hTel emerged as the most-responsive G4s to ligand-induced TO displacement, with several compounds reaching ∼50–60% displacement. However, the response profiles across compounds were too similar to enable meaningful discrimination of binding modes. hTert G4 was also excluded from further analysis due to the consistently poor-quality curves and a lack of differential ligand response, which precluded reliable interpretation. Ligand **21** stood out as the only compound capable of inducing substantial TO displacement across all G4 targets (∼80%) and even on the duplex control ds26 (∼20%), suggesting a nonselective binding profile likely dominated by end-stacking. However, the high ligand concentrations required (>20 equiv) point to weak binding affinity, distinguishing it from canonical high-affinity end-stackers like **NDIpip** and excluding it from further consideration. Additionally, ligand **15** exhibited appreciable TO displacement on ds26, suggesting reduced selectivity toward DNA G4s. Taken together, these findings support the hypothesis that the tested heptacyclic ligands predominantly act as groove binders. Among them, compounds **8**, **14**, **16**, and **24** showed the most promising profiles on the more challenging G4 targets myc and LTR-IV, while ligands **22** and **23** also displayed favorable selectivity and limited TO displacement.

To evaluate whether direct TO-ligand interactions could influence the interpretation of the FID data, UV–vis absorption experiments were performed under the same concentration conditions used in the assay (2 μM). The absorption spectra of TO, the individual ligands, and their corresponding 1:1 mixtures were compared. In all the cases, the experimental spectra of the mixtures closely matched the mathematical sum of the individual components, with no significant spectral shifts or new absorption bands observed (Figure S3). These results indicate the absence of strong ground-state interactions between TO and the heptacyclic ligands under the experimental conditions, supporting the interpretation of the FID responses in terms of ligand-G4 interactions rather than direct TO perturbation.

To further assess the selectivity of the heptacyclic ligands toward G4 structures, FRET-melting competition experiments were performed using a panel of competitor nucleic acid structures, including duplex (ds26 and ds12), a hairpin, and single-stranded scrambled DNA (ss-scr). The experiments were conducted on the two most promising targets identified by FID analysis, myc and LTR-IV (Figure 3 and Table S2). Overall, the addition of competitor sequences produced only marginal variations in the observed melting temperatures for all of the tested ligands. In both the myc and LTR-IV systems, the stabilizing effect of the heptacyclic compounds was largely preserved even in the presence of a large excess of competing nucleic acids, indicating that ligand binding to the target G4 structures is not readily displaced under competitive conditions. The absence of significant reductions in *T*_m_ suggests a marked preference of these ligands for the target G4 structures over those of alternative DNA conformations.

**Figure 3:**
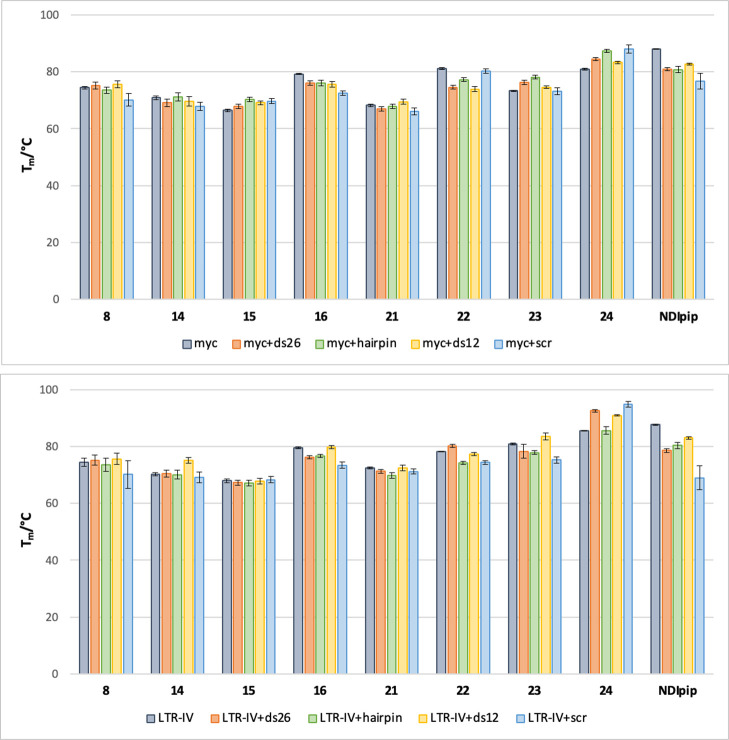
Resume of FRET-melting competition experiments performed with 0.25 μM of F­(myc)­T (top) and F­(LTR-IV)­T (bottom) in 10 mM lithium cacodylate buffer (pH = 7.2) containing 5 mM KCl and 95 mM LiCl for F­(myc)­T and 100 mM KCl for F­(LTR-IV)­T. Experiments were carried out in the presence of 4 equiv (1 μM) of each heptacyclic ligand and **NDIpip**, followed by the addition of 2.5 μM of competitor nucleic acid sequences (ds26, hairpin, ds12, and ss-scr).

In this context, **NDIpip** was included as a stringent reference ligand due to its well-established high G4 affinity and end-stacking binding mode. In a previous study, **NDIpip** demonstrated a strong stabilization of G4 structure (Δ*T*_m_ ≈35 °C) compared to minimal effects on duplex DNA (ΔTm ≈2 °C),^46^ consistent with a highly G4-preferential binding profile. In the present competitive FRET-melting experiments, the addition of competing nucleic acid structures resulted in a moderate reduction of thermal stabilization for **NDIpip**, with a decrease in Δ*T*_m_ in the range of approximately 5–12 °C depending on the competitor and the target sequence. This partial decrease reflects the dynamic nature of end-stacking interactions under conditions of nucleic acid competition, without compromising the overall preference for G4 structures. Importantly, despite the exceptionally strong performance of **NDIpip** as a reference end-stacking ligand, the heptacyclic compounds retain comparable or higher levels of stabilization across all competitive conditions tested. This behavior is particularly significant considering the fundamentally different binding mode of the new ligands, which act primarily as groove binders. The superior or at least noninferior performance of the heptacyclic scaffold in this stringent competitive setup further supports groove binding as a favorable determinant for robust and competition-resistant G4 recognition.

To further investigate the binding mode, CD experiments were first performed on the myc G4 in the presence of 4 equiv of ligands **8**, **14**, **16**, **22**, **23**, and **24**. No significant changes were observed in the overall CD spectral profiles upon ligand binding, indicating that the wild-type parallel G4 topology was preserved (Figure S4). CD-melting experiments, however, revealed that all tested ligands significantly stabilize the G4 structure (Figure 4). Among them, ligand **24** exhibited the strongest stabilizing effect, with a Δ*T*_m_ higher than 36.3 °C, while ligand **23** was the “least effective”, showing a Δ*T*_m_ of 26.6 ± 0.9 °C (Figures 4 and S5; Table S3).

**Figure 4:**
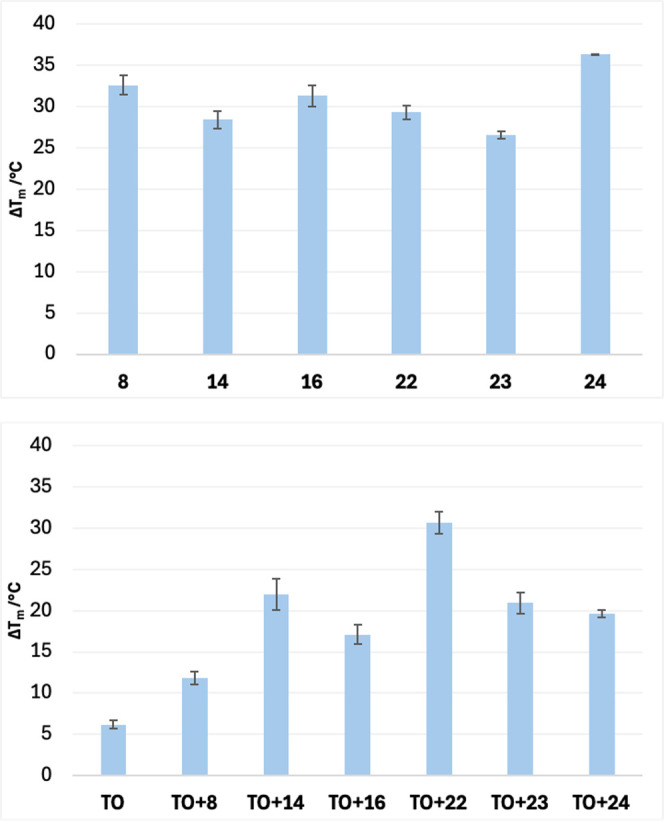
CD-melting stabilization temperatures of myc upon treatment with heptacyclic ligands **8**, **14**–**16**, and **22**–**24** alone (top) or in combination with TO (bottom). Conditions: 2 μM myc, 8 μM TO, 8 μM ligands, 1 mM KCl, 99 mM LiCl in 10 mM lithium cacodylate buffer (pH 7.2).

To evaluate the potential overlap between heptacyclic ligands and TO binding sites, myc G4 was preincubated with 4 equiv of TO for 4 h at room temperature, followed by the addition of the ligands and further 4 h incubation. Under these conditions, the resulting Δ*T*_m_ values were consistently lower than those observed in the ligand-alone experiments (Figure 4, bottom, S6–S7, and Table S3). This lack of additivity in thermal stabilization suggests that the ligands may interact, at least in part, with the external G-tetrads via π–π stacking interactions, indicating partial competition for overlapping binding sites between TO and the ligands.

To further clarify the observation from G4-FID assays and CD-melting experiments, ligand **14** was selected for molecular docking studies on the experimental NMR structure of myc-G4. This ligand was chosen due to its strong performance in the FID assay (substantially no TO displacement) and the marked Δ*T*_m_ enhancement observed in CD melting upon coincubation with TO, compared to TO alone. Specifically, while TO alone increased the myc melting temperature by 6 ± 1 °C, coincubation with ligand **14** resulted in a Δ*T*_m_ of 22 ± 4 °C, significantly higher than the 12 ± 2 °C observed for TO with ligand **8**. This disparity suggested a potential major groove binding contribution, making ligand **14** a suitable candidate for deeper investigation of this binding mode. The lowest-energy docking poses were analyzed in detail. Attention was devoted to identifying the nature of the stabilizing contacts established within the G4 grooves, including hydrogen-bonding, hydrophobic, and π-stacking interactions. Across all inspected poses, a common trend emerged: the heptacyclic ligand established multiple directional hydrogen-bonding contacts involving the heteroatoms of the oxadiazole/triazole-rich scaffold and the nucleobases or phosphate backbone of the G4 structures. In contrast, extensive π-stacking interactions with terminal G-tetrads were generally absent or limited, while hydrophobic contacts appeared to play only a minor role. These observations suggest that groove recognition is primarily driven by a network of polar interactions rather than by classical tetrad stacking. Computational docking studies revealed that one-half of the oligoaryl structure of ligand **14** inserted into the groove, while the other half protruded toward the outer G-tetrad (Figures 5 and S8A), consistent with dual groove and stacking interactions.^47^ Inspection of the docking pose revealed that ligand **14** is primarily aligned along the groove of the myc G4, where the heteroatom-rich heptacyclic scaffold is stabilized by an extended network of noncovalent interactions involving groove-exposed nucleobases (Figures S8B and S9). In particular, the binding mode is supported by a combination of hydrogen bonds, π-cation interactions, and salt bridges distributed along the groove-facing surface of the ligand. Several hydrogen-bond acceptor atoms located within the triazole, oxadiazole, and pyridine rings establish directional contacts with nucleobases lining the groove, while the protonated dimethylamino side chains participate in additional electrostatic interactions, including π-cation contacts and salt bridges. Together, these interactions anchor both termini of the ligand within the groove environment and contribute to the overall stability of the complex. The elongated architecture of the scaffold closely follows the contour of the groove, allowing the simultaneous formation of multiple contacts and maximizing shape complementarity with the G4 surface. The high density of nitrogen and oxygen atoms distributed across the scaffold, therefore, provides a favorable platform for the recognition of the polar groove environment, supporting groove recognition as the predominant binding mode. Notably, while most of the scaffold remains embedded within the groove region, one extremity of the molecule extends toward the outer G-tetrad, resulting in a limited surface overlap with the terminal quartet. Although this interaction is insufficient to support a classical end-stacking binding mode, it may account for the partial competition observed in the TO coincubation experiments and the reduced additivity of the corresponding Δ*T*_m_ values.

**Figure 5:**
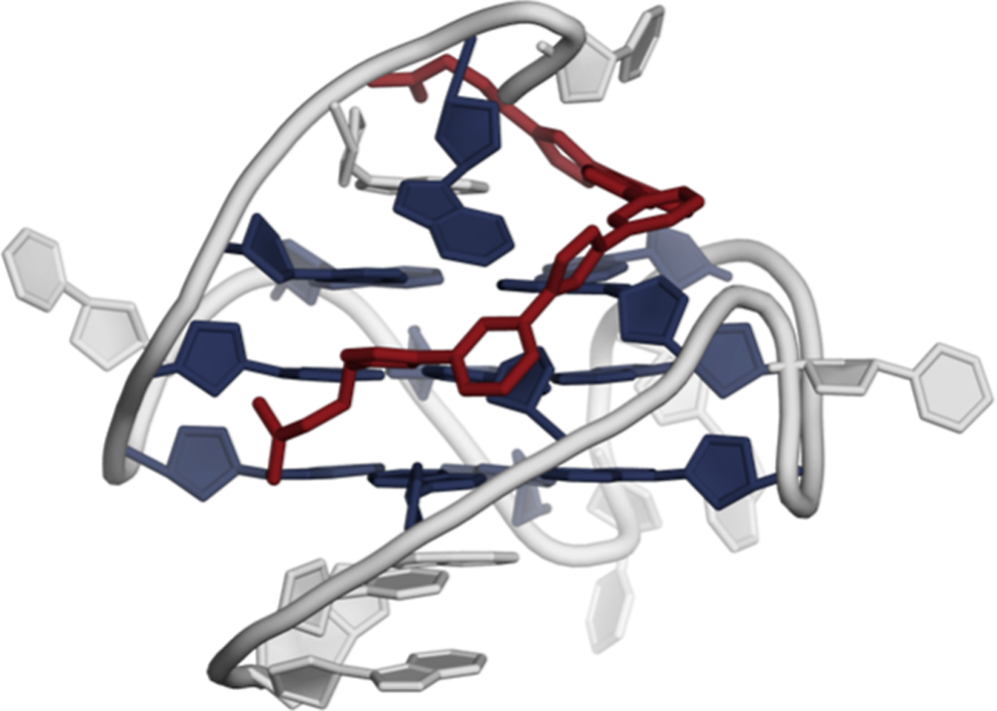
Docking of **14** with myc (PDB: 1XAV), showing a mixed groove-tetrad binding mode. One-half of the heptacyclic scaffold is effectively hindering one end-tetrad. Ligand **14** is represented in red, the guanine tetrads in blue, and the DNA backbone and other nucleobases in gray.

The calculated binding energy of −8.34 kcal/mol further supports the strong stabilization observed in CD-melting experiments.

A parallel set of studies was conducted on the LTR-IV G4, yielding contrasting results. CD spectra recorded in the presence of 4 equiv of the selected heptacyclic ligands showed no significant alterations compared to the spectrum of the unbound G4, confirming preservation of the native G4 topology (Figure S10). CD-melting experiments demonstrated that all tested ligands acted as strong stabilizers of the LTR-IV structure, with stabilization effects significantly exceeding those observed with TO alone (Figures 6 and S11; Table S4). Interestingly, when TO and the ligands were added sequentially (TO first, followed by the heptacyclic ligand, Figure S12), the resulting Δ*T*_m_ values were additive. In all cases, the final melting temperatures exceeded 95 °C, reflecting strong cooperative stabilization (Figure S13 and Table S4).

**Figure 6:**
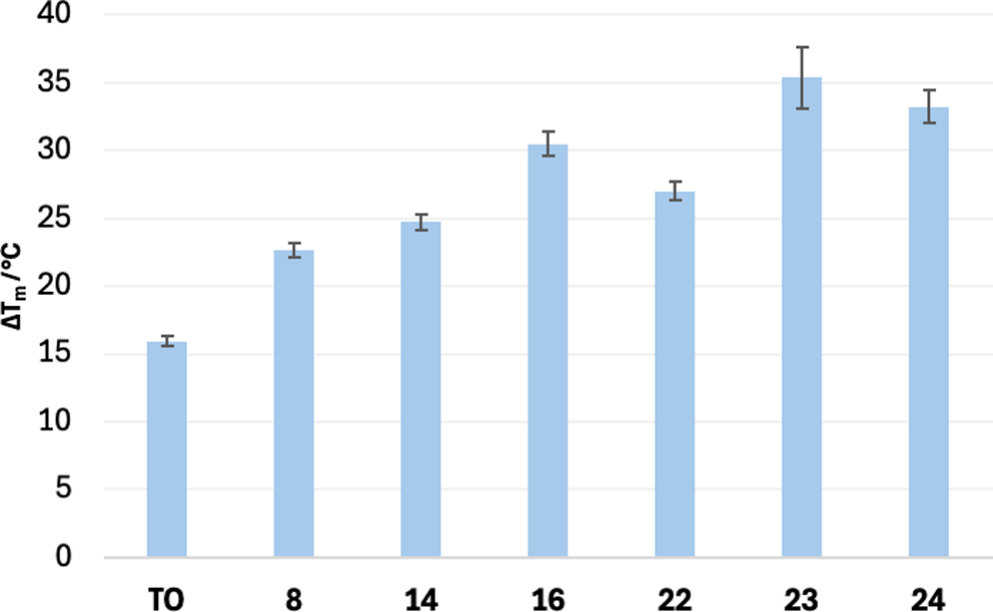
CD-melting stabilization temperatures of LTR-IV upon treatment with TO or heptacyclic ligands **8**, **14**–**16**, and **21**–**24**. Conditions: 2 μM LTR-IV, 8 μM compounds, 100 mM KCl, in 10 mM lithium cacodylate buffer (pH 7.2).

This additive behavior, unlike what was observed with myc G4, strongly suggests that TO and these new heptacyclic ligands bind to distinct sites on LTR-IV, without competing for the same structural features. To further clarify the experimental observations, ligand **24** was selected for computational studies based on G4-FID and CD-melting results. Molecular docking experiments corroborated the experimental findings, revealing that the heptacyclic ligands preferentially engage the LTR-IV G4 through groove binding rather than end-stacking with the terminal G-tetrads, with a binding energy of −10.25 kcal/mol (Figures 7 and S14). In detail, ligand **24** is deeply accommodated within the groove region, where it establishes an extended network of hydrogen-bonding interactions involving both the heteroaryl scaffold and the terminal amino substituent (Figure S15). Importantly, only a marginal overlap with the terminal G-tetrads is observed, and no significant π-stacking interactions are detected. Likewise, hydrophobic contacts contribute only minimally to the overall binding mode. These findings indicate that directional hydrogen-bonding interactions mainly drive stabilization of the complex, consistent with the negligible TO displacement observed in FID experiments and with the additive stabilization detected in the TO coincubation experiments.

**Figure 7:**
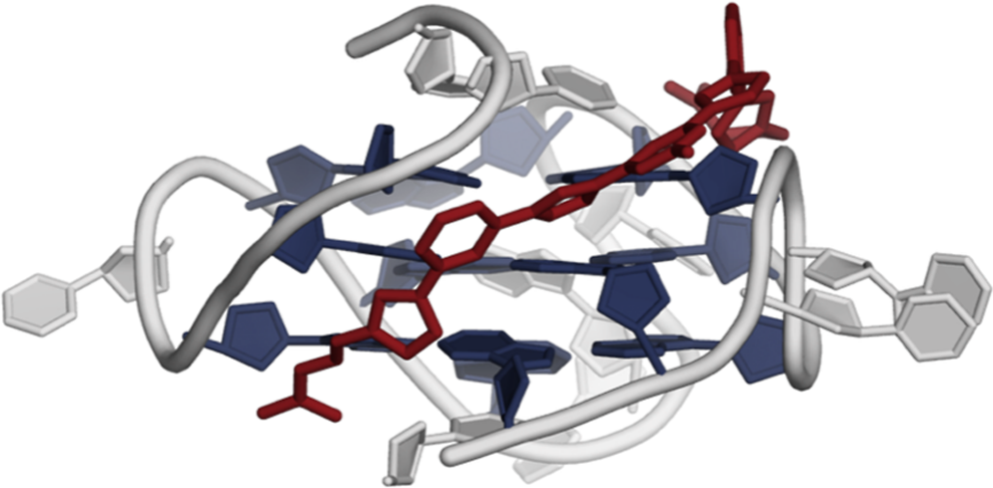
Docking of **24** with LTR-IV (PDB: 2N4Y), showing a groove-binding interaction. Ligand **24** is represented in red, the guanine tetrads in blue, and the DNA backbone and other nucleobases in gray.

In detail, ligand **24** is deeply accommodated within the groove region, where the heptacyclic scaffold adopts a conformation that closely matches the topology of the LTR-IV groove (Figures S14B and S15). Analysis of the docking pose reveals a dense pattern of stabilizing contacts involving both the heteroaryl core and the protonated side chains. The oxadiazole and triazole rings engage in multiple hydrogen-bonding interactions with groove-accessible nucleobases, whereas the dimethylamino substituents establish additional electrostatic contacts, including π-cation interactions and salt bridges, with neighboring phosphate groups and nucleobase surfaces. These interactions are distributed over the entire length of the ligand, effectively locking the scaffold within the groove cavity and promoting a highly complementary fit between the ligand and the G4 surface. Notably, the interaction pattern does not rely on a single dominant contact but rather on the cumulative contribution of several directional interactions, which collectively stabilize the complex. This behavior is consistent with the structural features of the heptacyclic scaffold, whose high density of nitrogen and oxygen atoms provides multiple interaction points capable of engaging the polar environment of the groove. In contrast, only marginal contact is observed between the aromatic core and the terminal G-tetrads, and no extended cofacial π-stacking arrangement can be identified. The absence of significant tetrad recognition, together with the extensive network of groove-mediated interactions, supports a binding mode primarily governed by groove accommodation rather than by classical end-stacking. This interpretation is fully consistent with both the negligible TO displacement observed in FID experiments and the additive stabilization detected in the TO coincubation experiments.

To probe the complementarity between groove-binding and end-stacking interactions, CD-melting experiments were performed in the presence of RHPS4, a well-established π-stacking G4 ligand.^48–51^ LTR-IV alone displayed a melting temperature of 56.7 ± 0.3 °C, which increased to 70.5 ± 0.7 °C upon addition of RHPS4. Remarkably, the simultaneous presence of RHPS4 and ligand **24** resulted in an extraordinary stabilization, with the melting temperature exceeding 95 °C (Figure S16). This pronounced increase strongly suggests a synergistic effect arising from the simultaneous engagement of distinct recognition sites on the G4.

To obtain preliminary quantitative information on ligand affinity, UV–visible titration experiments were carried out on selected heptacyclic derivatives that showed the most promising biophysical profiles against LTR-IV. Increasing concentrations of each ligand were added to a fixed concentration of LTR-IV (2 μM), and the resulting spectral changes were analyzed using HyperSpec software,^52^ assuming both 1:1 and 1:2 binding models. The fitting procedures yielded apparent association constants (K_1:1_) ranging from 1.24 × 10^5^ to 1.07 × 10^6^ M^–1^ (Table S5 and Figures S17 and S18), confirming the ability of this class of compounds to recognize and stabilize the target G4 structure efficiently. In particular, ligands **16** and **24** showed the highest affinities within the series, with K_1_:_1_ values of 1.07 × 10^6^ and 8.76 × 10^5^ M^–1^, respectively, consistent with their superior performance in the biophysical assays.

Taken together, these findings clearly identify the tested heptacyclic ligands as potent and selective groove binders of the LTR-IV G4, providing compelling proof of concept for the combined exploitation of groove-binding and end-stacking interactions. Notably, ligand **24** features an amine handle suitable for future functionalization, enabling the design of conjugates and multivalent constructs. This integrated strategy paves the way for future target-guided synthesis approaches aimed at incorporating these two complementary binding motifs into a single molecular framework with the goal of developing highly selective and exceptionally stable ligands for LTR-IV.

The limited differences observed among nitrogen- and carbon-substituted analogues can be rationalized considering the groove-binding mode identified through both biophysical and computational studies. Molecular docking revealed that recognition of the LTR-IV G4 is primarily driven by an extended network of groove-mediated interactions involving the heteroaryl scaffold and the protonated side chains, while only marginal contacts are established with the terminal G-tetrads. In this context, the overall binding energy is distributed across multiple hydrogen-bonding, electrostatic, and π-cation interactions rather than being dominated by a single contact or by extensive aromatic stacking.

Consequently, subtle electronic variations arising from the replacement of pyridine nitrogen atoms with carbon atoms are expected to have only a limited impact on their overall affinity. Instead, binding appears to be governed mainly by the ability of the heptacyclic framework to achieve a highly complementary fit within the groove environment and establish a dense network of cooperative interactions along its entire length. The conformational adaptability of the scaffold likely enables different analogues to converge toward similarly favorable groove-bound geometries, thereby compensating for minor structural and electronic perturbations.

Overall, these observations indicate that molecular recognition is primarily dictated by shape complementarity and multivalent groove-mediated interactions rather than by the electronic nature of the individual aromatic units. This interpretation is fully consistent with the comparable affinity values measured across the series and with the docking results, which highlight groove accommodation as the dominant determinant of ligand binding to the LTR-IV G4.

## Conclusions

In this work, we have developed a new family of water-soluble oligo-heteroaromatic heptacycles through a versatile and efficient synthetic route. The strategy relies on a modular and convergent approach that enables the late-stage introduction of diverse structural motifs under mild conditions, circumventing the low yields and harsh conditions typically associated with the macrocyclic ligand synthesis. Biophysical analyses confirmed that the newly synthesized compounds act as effective groove binders for G-quadruplex structures, particularly for the parallel LTR-IV topology, where they induce strong thermal stabilization without competing with end-stacking ligands, such as thiazole orange. Despite the deliberate variation of central atoms (*X* and *Y* = C or N) to modulate electronic and steric properties, no significant differences in binding affinity or G4 selectivity were observed among the analogues, suggesting that this region of the scaffold may tolerate further functional diversity. A key feature of these ligands is their excellent water solubility under physiological conditions owing to the presence of terminal tertiary amine groups. This differentiates them from previously reported nonmacrocyclic G4 binders, many of which suffer from poor solubility and limited biological applicability. Overall, these findings position the new heptacyclic ligands as promising and tractable candidates for further development in G-quadruplex-targeting strategies, including functional conjugation for imaging or therapeutic applications. While the present study provides a detailed biophysical and mechanistic characterization of a new heptacyclic scaffold for G-quadruplex recognition, biological validation in cellular systems remains an important next step. Future investigations will, therefore, focus on evaluating the cellular activity of these compounds, including their ability to enter cells, engage G4 targets in a native chromatin context, and modulate downstream biological processes associated with G4-containing genomic regions. These studies will be essential to establish whether the strong in vitro binding and stabilization properties observed translate into functional effects in biologically relevant environments, where additional factors such as molecular competition, chromatin organization, and intracellular distribution may significantly influence ligand behavior.

## Materials and Methods

Reagents, solvents, and chemicals were purchased from commercial sources (Alfa Aesar, Sigma-Aldrich, TCI, or Fluorochem) and used without further purification. The oligonucleotide sequences (Table S1) were purchased from Biomers as HPLC-purified compounds with a purity of >99%. TLC analyses were conducted on silica gel (Merck 60F-254) with visualization at 254 and 366 nm. For flash column chromatography purification, an Isolera ONE Flash Chromatography System (MPLC, Biotage), combined with a tunable wavelength UV–vis detector, was used (SNAP KP-SIL 25 or 50 g columns, Biotage). All the syntheses assisted by microwaves were performed using a Discover microwave synthesizer (CEM). HPLC analyses were performed using an Agilent system SERIES 1260 with a SepaChrom ROBUSTA C18 column (3 μm, 4.6 × 50 mm) and a flow rate of 1.4 mL/min. The following method was used: isocratic elution over 2 min at 95% of 0.1% trifluoroacetic acid in water/5% acetonitrile, gradually increasing to 60% acetonitrile over 8 min, and then isocratic elution for 4 min (λ = 256 nm). Compounds were purified on an Agilent Technologies 1260 Infinity preparative HPLC using a Waters XSelect CSH Prep C18 OBDTM column (5 μm, 100 × 30 mm), with a 30 mL/min flow.

^1^H- and ^13^C NMR spectra were recorded on a Bruker ADVANCE 200 MHz, Bruker AVANCE 300 MHz, or a Bruker ADVANCE 400 MHz spectrometer. UPLC-MS data were recorded using a Surveyor UHPLC system (Thermo Finnigan, San Jose, CA, USA) equipped with a BEH Acquity UPLC column (1.7 μm) 2.1 × 50 mm and an LCQ ADV MAX ion-trap mass spectrometer, with an ESI ion source.

UV–vis absorption experiments were performed on an Agilent Cary 60 spectrophotometer equipped with a Cary Single Cell Peltier temperature controller. Solutions of the heptacyclic ligands were prepared in water at increasing concentrations in the 2–20 μM range. Absorption spectra were recorded for each concentration under identical experimental conditions. For each ligand, the absorbance maximum was plotted as a function of concentration to evaluate compliance with the Beer–Lambert behavior.

The fluorescence intercalation displacement (FID) assay was performed using an Agilent Cary Eclipse spectrofluorometer. All oligonucleotides were diluted from stocks of 500 μM in water at a final concentration of 1 μM in lithium cacodylate buffer (10 mM, pH 7.2) containing 100 mM KCl. The solutions were annealed by heating at 95 °C for 5 min, followed by slow cooling to room temperature over 4 h. TO was added to each G4 solution at a final concentration of 2 μM (from a 10 mM stock in DMSO), and the samples were incubated for 30 min at room temperature to allow for equilibrium binding. Titrations were then performed by incremental addition of the test ligands (from 0 to 20 μM final concentration) with fluorescence recorded after each addition. Fluorescence emission was measured at 535 nm (excitation at 501 nm) using a 1 cm path length quartz cuvette. The percentage of TO displacement was calculated using the formula:

$$Displacement\;\%=\left[1-\frac{F_{obs}}{F_{TO}}\right]\times 100$$

where *F*_obs_ is the fluorescence intensity at a given ligand concentration and *F*_TO_ is the fluorescence of the DNA-TO complex before ligand addition. The FID assay was performed for compounds 8, 14–16, and 21–24 using five G-quadruplex-forming sequences (hTel, LTR-III, LTR-IV, hTert, and myc) and one duplex DNA control (ds26). For comparison, titrations were also performed with the reference G4 ligand **NDIpip**, which is a tetrasubstituted naphthalenediimide.

Stock solutions of TO and the heptacyclic ligands were prepared in water and diluted to a final concentration of 2 μM in 10 mM lithium cacodylate buffer (pH = 7.2) with 100 mM KCl. Absorption spectra of TO alone and of each individual ligand were first recorded separately. Subsequently, mixtures containing TO (2 μM) and each heptacyclic ligand (2 μM) were prepared under identical buffer conditions, and their absorption spectra were acquired. The experimental spectra of the mixtures were then compared with the mathematical sum of the spectra of the corresponding individual components.

FRET-melting competition experiments were performed using FAM-TAMRA-labeled G4-forming oligonucleotides. The myc oligonucleotide was folded in 10 mM lithium cacodylate buffer (pH = 7.2) containing 5 mM KCl and 95 mM LiCl, while LTR-IV was folded in 10 mM lithium cacodylate buffer (pH = 7.2) containing 100 mM KCl. Oligonucleotide solutions were heated at 95 °C for 5 min and then cooled on ice for 30 min prior to use. Folded oligonucleotides (0.25 μM) were incubated with the heptacyclic ligands and **NDIpip** (1 μM) for 4 h at room temperature. Subsequently, competitor nucleic acid sequences (ds26, ds12, hairpin, and SS-scr) were added, and the mixtures were further incubated for 4 h at room temperature. FRET-melting competition experiments were carried out in 96-well plates by monitoring fluorescence as a function of temperature from 20 to 95 °C, using a heating rate of 1 °C/min. Fluorescence measurements were acquired throughout the melting process, and melting temperatures (*T*_m_) were determined from the resulting denaturation profiles using OriginPro 8.5.

Circular Dichroism spectra were recorded using a JASCO J-1500 spectropolarimeter (JASCO Corporation, Easton, MD, USA), equipped with a Peltier temperature controller, in a quartz cuvette with an optical path length of 1 cm. All oligonucleotides were diluted from 500 μM stock solutions in water to the final concentration (2 μM) in lithium cacodylate buffer (10 mM, pH 7.2) containing different concentrations of KCl (1 mM for myc and 100 mM for LTRIV). For myc, the ionic strength was kept constant by adding 99 mM of LiCl. The solutions were annealed by heating at 95 °C for 5 min and gradually cooling to room temperature over 4 h. CD spectra were recorded at 293 K, with an instrument scanning speed of 200 nm/min, a response of 2 s over a wavelength range of 210–340 nm, and a 1 nm sampling interval. The reported spectra of each sample represent the average of 4 scans and are baseline-corrected for signal contributions due to the buffer mixture. CD spectra were normalized to Δε (M^–1^ cm^–1^) according to the relationship:

$$\Delta \varepsilon =\frac{\vartheta }{32980\;Cl}$$

where ϑ corresponds to observed ellipticities (in millidegrees), C is the DNA molar concentration, and l is the path length in centimeters. For the CD-melting experiments of G4 structures (2 μM), the ellipticity was recorded at 264 nm for myc and LTRIV every 0.5 °C, with a temperature scan rate of 1 °C/min in the range of 20–95 °C. Data were averaged over three independent experiments. Melting temperature values were calculated according to the Boltzmann equation applied for a two-state transition from a folded to unfolded state, assuming that the heat capacities of the folded and unfolded states are equal. Δ*T*_m_ was calculated as the difference between *T*_m_ in the presence and absence of the compounds. For CD and CD-melting studies, heptacyclic ligands were diluted from 10 mM DMSO stock solutions to a final concentration of 8 μM (4 equiv relative to the oligonucleotide) and incubated for 4 h with G4s at room temperature before measurements. In combination experiments with TO, TO was first added from a 10 mM DMSO stock to a final concentration of 8 μM and incubated for 4 h at room temperature with G4s. Then, the heptacyclic ligand was added under the same conditions (8 μM, 4 equiv), followed by an additional 4 h incubation before recording the CD spectrum and melting curve.

Docking studies were performed on the NMR-deposited structure of the cMyc22 (PDB:1XAV) and the LTR-IV (PDB:2N4Y) G4 structures. Molecular docking calculations were carried out using AutoDock 4 with the aid of its graphical user interface, AutoDockTools 1.5.7. The ligand and DNA oligonucleotides were prepared by use of AutoDock Tools 1.5.7 by assigning bond orders, adding hydrogen atoms, and generating the appropriate protonation states. The ligand and DNA oligonucleotides were then converted to proper Autodock PDBQT file formats, and the Gasteiger charges were assigned. The number of torsions was set as the total number of rotatable bonds in both ligands, and specifically, TORSDOF = 14. The 3D grid box dimensions were defined, including the whole DNA macromolecule. The docking area was centered on the DNA center of mass, and grid boxes of 33 Å × 24 Å × 31 Å for cMyc22 and 27 Å × 27 Å × 27 Å for LTRIV G4s were used, respectively, with a 0.603 Å and 0.681 Å spacing. One hundred docking poses were generated by using as docking parameters seed = random, exhaustiveness = 24, and number of binding modes = 20 for each of the three runs performed for both DNA/ligand systems. Docking poses were clustered based on their root mean square deviation (RMSD) and ranked based on their binding energy. Molecular modeling figures were drawn by PyMOL. To gain further insight into the molecular determinants of binding, the representative lowest-energy docking poses obtained from the AutoDock calculations and the corresponding experimental G4 structures retrieved from the Protein Data Bank (PDB: 1XAV and 2N4Y) were imported into Maestro (Schrödinger Release 2025-1, Schrödinger LLC, New York, NY, USA) for further analysis. Two-dimensional interaction maps were generated from the docked complexes to identify and visualize the noncovalent contacts established between the ligands and the G4 structures. Hydrogen-bonding interactions, salt bridges, π-cation interactions, hydrophobic contacts, and π-stacking interactions were systematically inspected and used to support the structural interpretation of the docking results and the proposed groove-binding recognition mode. Molecular interaction diagrams reported in the Supporting Information were generated using this workflow.

UV–vis absorption measurements were performed on an Agilent Cary 60 spectrophotometer equipped with a Cary Single Cell Peltier temperature controller. Titration experiments were carried out using a fixed concentration of LTR-IV G4 (2 μM) and increasing concentrations of the heptacyclic ligands (0–10 μM) in 10 mM lithium cacodylate buffer (pH = 7.2) containing 100 mM KCl. After preparation, samples were incubated for 4 h at room temperature to allow equilibration of the ligand-G4 complexes prior to spectral acquisition. To account for the intrinsic absorbance contribution of the ligands, independent UV–vis titrations of each ligand were performed under identical experimental conditions and over the same concentration range (0–10 μM) in the absence of LTR-IV. The corresponding spectra were mathematically subtracted from those recorded for the ligand-G4 mixtures, yielding spectra associated with the interaction between the ligand and the G4 target. Titration data of G4-ligand species were processed with the Hyperquad package,^52^ by determining the log K values from the plots of absorbance values, corrected for dilution.

### Synthesis of *N* ^2^, *N* ^6^-Dihydroxypyridine-2,6-bis­(carboximidamide) 2

To a solution of **1** (3.87 g, 30 mmol, 1.0 equiv) in 100 mL EtOH, 100 mL of a 0.7 M aqueous solution of hydroxylamine hydrochloride in water (4.86 g, 70 mmol, 2.3 equiv) previously neutralized with solid sodium hydroxide (2.8 g, 70 mmol, 2.3 equiv) was added. The reaction mixture was stirred at 70 °C for 30 min, then cooled to 10 °C (RP-TLC H_2_O/MeCN 6:4 + 0.1% TFA, *R*_f_(**1**) = 0.16, *R*_f_(**2**) = 0.42). The resulting solid was filtered and washed with water. The product was obtained as a crystalline solid without further purification. 5.21 g, 89% yield. *N* ^2^, *N* ^6^-dihydroxypyridine-2,6-bis­(carboximidamide) **2**: ^1^H NMR (300 MHz, DMSO-*d*_6_), δ: (ppm) = 2.51 (DMSO-*d*_6_), 3.41 (DMSO-*d*_6_), 6.31 (br s, 4H), 7.75–7.89 (m, 3H), 9.88 (s, 2H). ^13^C NMR (75 MHz, DMSO-*d*_6_), δ: (ppm) = 119.2, 136.7, 149.0, 149.6.

### Synthesis of *N*′^1^, *N*′^3^-Dihydroxyisophthalimidamide 10

To a solution of **9** (1.06 g, 8.3 mmol, 1.0 equiv) in 40 mL of ethanol, 25 mL of a 0.8 M solution of hydroxylamine hydrochloride (1.45 g, 20.9 mmol, 2.5 equiv) in water previously neutralized with potassium carbonate (1.46 g, 10.6 mmol, 1.3 equiv) was added. The reaction was stirred at reflux for 16 h (TLC CHCl_3_/MeOH 8:2, *R*_f_(**9**) = 0.91, *R*_f_(monosub.) = 0.85, *R*_f_(10) = 0.40). The volatiles were removed at reduced pressure, and the crude was purified using FCC (Gradient CHCl_3_/MeOH). Pale-yellow crystals, 1.39 g, 86% yield. *N*′^1^, *N*′^3^-dihydroxyisophthalimidamide **10**: ^1^H NMR (300 MHz, CD_3_OD), δ: (ppm) = 7.37–7.48 (t, 1H), 7.65–7.74 (m, 2H), 7.90–7.95 (s, 1H).

### Synthesis of 5-Nitroisophthalamide 18

Dimethyl 5-nitroisophthalate **17** (2.51 g, 10.0 mmol, 1.0 equiv) was dissolved in methanol (10 mL). A 7 M solution of ammonia in methanol (10 mL, 70.0 mmol, 7.0 equiv) was added and stirred at room temperature for 16 h (TLC CHCl_3_/MeOH 9:1, *R*_f_(**17**) = 0.95, *R*_f_(**18**) = 0.20). Upon completion, water (100 mL) was added, and the resulting precipitate was filtered and washed with more water (3 × 50 mL), dried, and recrystallized from ethanol/water. Yellow solid, 1.93 g, 88% yield. 5-Nitroisophthalamide **18**: ^1^H NMR (300 MHz, DMSO-*d*_6_), δ: (ppm) = 7.80 (br s, 2H), 8.43 (br s, 2H), 8.81 (s, 3H). ^13^C NMR (75 MHz, DMSO-*d*_6_), δ: (ppm) = 38.6–40.2 (DMSO-*d*_6_), 124.5, 132.7, 136.0, 147.9, 165.4.

### Synthesis of 5-Nitroisophthalonitrile **19**

**18** (1.16 g, 5.54 mmol, 1.0 equiv) was dissolved in dry THF (30 mL) under an Ar atmosphere and cooled to 0 °C. Triethylamine (2.3 mL, 16.6 mmol, 3.0 equiv) was added, followed by a dropwise addition of trifluoroacetic anhydride (2.3 mL, 16.6 mmol, 3.0 equiv) over a 1h period. After this, the mixture was allowed to warm up at room temperature and stirred for 3 h (TLC CHCl_3_/MeOH 9:1, *R*_f_(**18**) = 0.20, *R*_f_(**19**) = 0.86). Upon completion, the mixture was cooled to 0 °C and quenched with 20 mL of sat. aq. NaHCO_3_. The volatiles were removed at reduced pressure, and the product was extracted with DCM (3 × 20 mL). The combined organic phases were dried over MgSO_4_, filtered, and concentrated. The crude was purified using FCC (Gradient Cyclohexane: CHCl_3_). Yellow solid, 777 mg, 82% yield. 5-Nitroisophthalonitrile **19**: ^1^H NMR (300 MHz, DMSO-*d*_6_), δ: (ppm) = 2.60 (DMSO-*d*_6_), 3.40 (DMSO-*d*_6_), 9.01 (s, 1H), 9.17 (s, 2H). ^13^C NMR (75 MHz, DMSO-*d*_6_), δ: (ppm) = 38.7–40.4­(DMSO-*d*_6_), 114.1, 115.7, 131.8, 142.1, 148.1.

### Synthesis of *N*′^1^, *N*′^3^-Dihydroxy-5-nitroisophthalimidamide **20**

To a solution of **19** (1.85 g, 10.7 mmol, 1.0 equiv) in 25 mL of ethanol, 25 mL of a 1.0 M solution of hydroxylamine hydrochloride (1.45 g, 25.1 mmol, 2.3 equiv) in water previously neutralized with sodium carbonate (1.33 g, 12.5 mmol, 1.2 equiv) was added. The reaction was stirred at room temperature for 16 h (TLC CHCl_3_/MeOH 8:2, *R*_f_(**19**) = 0.86, *R*_f_(**20**) = 0.31). The vessel was cooled in an ice bath, and the crystallized solid was filtered, washed with water (3 × 50 mL), dried, and recrystallized from ethanol/water. Yellow crystals, 2.20 g, 86% yield. *N*′^1^, *N*′^3^-dihydroxy-5-nitroisophthalimidamide **20**: ^1^H NMR (300 MHz, CD_3_OD), δ: (ppm) = 2.50 (DMSO-*d*_6_), 3.42 (DMSO-*d*_6_), 6.13 (s, 4H), 8.42 (s, 1H), 8.49 (s, 2H), 10.02 (s, 2H). ^13^C NMR (75 MHz, DMSO-*d*_6_), δ: (ppm) = 38.6–40.3­(DMSO-*d*_6_), 120.0, 128.2, 134.9, 147.7, 149.1.

### Synthesis of 3-Azido-*N*, *N*-dimethylpropan-1-aminium Chloride 4

**3** (1.8 g, 13.9 mmol, 1.0 equiv) was dissolved in 50 mL of water. NaN_3_ (2.7 g, 41.7 mmol, 3.0 equiv) was added, then the mixture was heated at 80 °C and stirred overnight. Upon completion (GC–MS), the mixture was neutralized at 0 °C with 2 M NaOH (6 mL), then extracted with Et_2_O (3 × 50 mL). HCl gas was bubbled in the ethereal mixture to form the hydrochloride salt, then the solvent was evaporated. Pale yellow oil, 1.6 g, quantitative yield. 3-azido-*N*, *N*-dimethylpropan-1-aminium chloride **4**: ^1^H NMR (300 MHz, CD_3_OD), δ: (ppm) = 1.99–2.11 (qui, 2H, *J* = 11.6 Hz, 8.0 Hz, 4.1 Hz), 2.93 (s, 6H), 3.22–3.37 (m, 2H), 3.50–3.60 (t, 2H, *J* = 9.9 Hz), 5.21 (s, 3H).

### Synthesis of Methyl 6-((trimethylsilyl)­ethynyl)­picolinate **5a**

To a solution of methyl 6-bromopicolinate (**5**) (300 mg, 1.39 mmol, 1.0 equiv) in 7 mL of dry toluene under a nitrogen atmosphere, copper iodide (6.0 mg, 0.032 mmol, 0.02 equiv), triphenylphosphine (15.0 mg, 0.022 mmol, 0.016 equiv), DIPEA (2.5 mL, 14.4 mmol, 10.3 equiv), and palladium­(II) bis­(triphenylphosphine) dichloride (20.0 mg, 0.029 mmol, 0.02 equiv) were added in sequence. The resulting pale-yellow solution was stirred for 10 min at room temperature, then trimethylsilyl acetylene (290 μL, 2.04 mmol, 1.5 equiv) was added, and the mixture was heated at 80 °C and stirred for 1 h under a nitrogen atmosphere (TLC Cyclohexane/AcOEt 8:2, *R*_f_(**5**) = 0.28, *R*_f_(**5a**) = 0.39). Upon completion, the mixture was filtered, and the organic solvent was washed with water (3 × 10 mL), dried over Na_2_SO_4_, filtered, and concentrated. The product was used without any further purification. Pale orange oil, 300 mg, 92% yield. Methyl 6-((trimethylsilyl)­ethynyl)­picolinate **5a**: ^1^H NMR (300 MHz, CDCl_3_), δ: (ppm) = 0.11 (s, 9H), 3.84 (s, 3H), 7.45–7.51 (d, 1H, *J* = 7.8 Hz), 7.64–7.73 (t, 1H, *J* = 7.8 Hz), 7.86–7.93 (d, 1H, *J* = 7.8 Hz). ^13^C NMR (75 MHz, CDCl_3_), δ: (ppm) = −0.62, 52.6, 77.0 (CDCl_3_), 96.0, 102.7, 124.0, 130.3, 137.0, 143.0, 148.0, 164.9.

### Synthesis of Methyl 6-Ethynylpicolinate 6

**5a** (1.37 g, 5.9 mmol, 1.0 equiv) was dissolved in MeOH (100 mL), and K_2_CO_3_ (200 mg, 1.5 mmol, 0.25 equiv) was added. The mixture was stirred at room temperature for 2 h (TLC Cyclohexane/AcOEt 8:2, *R*_f_(**5a**) = 0.39, *R*_f_(**6**) = 0.25), and upon completion, the solvent was removed at reduced pressure. The crude was purified using FCC (Cyclohexane/AcOEt 8:2). Yellow solid, 827 mg, 87% yield. Methyl 6-ethynylpicolinate **6**: ^1^H NMR (300 MHz, CDCl_3_), δ: (ppm) = 3.21 (s, 1H), 4.00 (s, 3H), 7.62–7.68 (d, 1H, *J* = 7.8 Hz), 7.79–7.88 (t, 1H, *J* = 7.8 Hz), 8.07–8.13 (d, 1H, *J* = 7.8 Hz). ^13^C NMR (75 MHz, CDCl_3_), δ: (ppm) = 52.9, 76.5–77.3 (CDCl_3_), 78.4, 81.9, 124.5, 128.3 (impurity), 130.4, 131.9 (impurity), 137.2, 142.4, 148.3, 165.0.

### Synthesis of Methyl 3-((trimethylsilyl)­ethynyl)­benzoate **11a**

The compound was synthesized according to the literature.^53^

### Synthesis of Methyl 3-Ethynylbenzoate 12

The compound was synthesized according to the literature.^54^

### Synthesis of Methyl 6-(1-(3-(dimethylamino)­propyl)-1H-1,2,3-triazol-4-yl)­picolinate **6a**

To a solution of **6** (200 mg, 1.24 mmol, 1.0 equiv) in tBuOH (5 mL), copper sulfate pentahydrate (31.0 mg, 0.12 mmol, 0.1 equiv), sodium ascorbate (246 mg, 1.24 mmol, 1.0 equiv), and a solution of **4** (226 mg, 1.37 mmol, 1.1 equiv) in water (5 mL) were added at room temperature in sequence. The mixture was stirred for 24 h at room temperature (TLC CHCl_3_/MeOH 98:2, *R*_f_(**6**) = 0.83, *R*_f_(**6a**) = 0.21). Upon completion, the organic solvent was removed at reduced pressure, and the residue was treated with sat. aq. NaHCO_3_ (10 mL) and extracted with DCM (3 × 15 mL). The organic phases were joined, dried over Na_2_SO_4_, filtered, and concentrated. The crude was purified using FCC (Gradient CHCl_3_/MeOH). Yellowish oil, 305 mg, 85% yield. Methyl 6-(1-(3-(dimethylamino)­propyl)-1*H*-1,2,3-triazol-4-yl)­picolinate **6a**: ^1^H NMR (300 MHz, CDCl_3_), δ: (ppm) = 2.10–2.21 (qui, 2H, *J* = 6.8 Hz), 2.25 (s, 6H), 2.30–2.39 (t, 2H, *J* = 6.8 Hz), 4.02 (s, 3H), 4.48–4.57 (t, 2H, *J* = 7.0 Hz), 7.90–7.99 (t, 1H, *J* = 7.8 Hz), 8.02–8.09 (d, 1H, *J* = 7.7 Hz), 8.36 (s, 1H), 8.36–8.42 (d, 1H, *J* = 7.9 Hz). ^13^C NMR (75 MHz, CDCl_3_), δ: (ppm) = 28.0, 45.2, 48.2, 52.6, 55.7, 76.6–77.5 (CDCl_3_), 122.9, 123.2, 123.9, 137.7, 147.3, 147.5, 150.7, 165.4.

### Synthesis of Methyl 3-(1-(3-(dimethylamino)­propyl)-1*H*-1,2,3-triazol-4-yl)­benzoate **12a**

To a solution of **12** (199 mg, 1.24 mmol, 1.0 equiv) in tBuOH (5 mL), copper sulfate pentahydrate (31.0 mg, 0.12 mmol, 0.1 equiv), sodium ascorbate (246 mg, 1.24 mmol, 1.0 equiv), and a solution of **4** (226 mg, 1.37 mmol, 1.1 equiv) in water (5 mL) were added at room temperature in sequence. The mixture was stirred for 24 h at room temperature (TLC CHCl_3_/MeOH 98:2, *R*_f_(**12**) = 0.80, *R*_f_(**12a**) = 0.22). Upon completion, the organic solvent was removed at reduced pressure, and the residue was treated with sat. aq. NaHCO_3_ (10 mL) and extracted with DCM (3 × 15 mL). The organic phases were joined, dried over Na_2_SO_4_, filtered, and concentrated. The crude was purified using FCC (Gradient CHCl_3_/MeOH). Yellowish oil, 340 mg, 95% yield. Methyl 3-(1-(3-(dimethylamino)­propyl)-1*H*-1,2,3-triazol-4-yl)­benzoate **12a**: ^1^H NMR (300 MHz, CDCl_3_), δ: (ppm) = 2.07–2.18 (qui, 2H, *J* = 6.8 Hz), 2.24 (s, 6H), 2.26–2.35 (t, 2H, *J* = 6.8 Hz), 3.94 (s, 3H), 4.45–4.54 (t, 2H, *J* = 6.9 Hz), 7.46–7.55 (t, 1H, *J* = 7.8 Hz), 7.89 (s, 1H), 7.96–8.03 (d, 1H, *J* = 7.8 Hz), 8.08–8.14 (d, 1H, *J* = 7.8 Hz), 8.42 (s, 1H). ^13^C NMR (75 MHz, CDCl_3_), δ: (ppm) = 28.0, 45.2, 48.1, 52.1, 55.6, 76.5–77.4 (CDCl_3_), 120.3, 126.6, 128.9, 129.9, 130.6, 131.0, 146.6, 166.7.

### Synthesis of 6-(1-(3-(dimethylamino)­propyl)-1*H*-1,2,3-triazol-4-yl)­picolinic Acid 7

**6a** (333 mg, 1.15 mmol, 1.0 equiv) and potassium carbonate (238 mg, 1.75 mmol, 1.5 equiv) were dissolved in a 1:1 water/THF mixture (30 mL). The reaction was heated to reflux for 2 h, after which the solvent was removed at reduced pressure. The crude was purified from inorganic salts using preparative HPLC (H_2_O + 0.1% TFA/MeCN). White solid, 448 mg, quantitative yield (TFA salt). 6-(1-(3-(dimethylamino)­propyl)-1*H*-1,2,3-triazol-4-yl)­picolinic acid **7**: ^1^H NMR (300 MHz, DMSO-*d*_6_), δ: (ppm) = 2.26–2.39 (qui, 2H, *J* = 8.0 Hz), 2.50 (DMSO-*d*_6_), 2.78 (s, 6H), 3.07–3.16 (m, 2H), 4.54–4.62 (t, 2H, *J* = 6.8 Hz), 7.96–8.03 (d, 1H, *J* = 7.6 Hz), 8.05–8.14 (t, 1H, *J* = 7.7 Hz), 8.22–8.28 (d, 1H, *J* = 7.7 Hz), 8.81 (s, 1H). ^13^C NMR (75 MHz, DMSO-*d*_6_), δ: (ppm) = 24.6, 38.7–40.4 (DMSO-*d*_6_), 42.1, 47.0, 53.9, 122.5, 123.6, 124.2, 138.6, 146.7, 148.3, 149.9, 158.5, 165.8.

### Synthesis of 3-(1-(3-(dimethylamino)­propyl)-1*H*-1,2,3-triazol-4-yl)­benzoic Acid 13

**12a** (332 mg, 1.15 mmol, 1.0 equiv) and potassium carbonate (238 mg, 1.75 mmol, 1.5 equiv) were dissolved in a 1:1 water/THF mixture (30 mL). The reaction was heated to reflux for 16 h, after which the solvent was removed at reduced pressure. The crude was purified from inorganic salts using preparative HPLC (H_2_O + 0.1% TFA/MeCN) and a recrystallization from iPrOH. White solid, 447 mg, quantitative yield (TFA salt). 3-(1-(3-(dimethylamino)­propyl)-1*H*-1,2,3-triazol-4-yl)­benzoic acid **13**: ^1^H NMR (300 MHz, DMSO-*d*_6_), δ: (ppm) = 1.23 (impurity), 2.36–2.30 (qui, 2H, *J* = 6.8 Hz), 2.50 (DMSO-*d*_6_), 2.74 (s, 6H), 3.05–3.14 (m, 2H), 3.37 (H_2_O), 4.51–4.59 (t, 2H, *J* = 6.8 Hz), 7.56–7.65 (t, 1H, *J* = 7.7 Hz), 7.88–7.95 (d, 1H, *J* = 7.8 Hz), 8.06–8.13 (d, 1H, *J* = 7.7 Hz), 8.42 (s, 1H), 8.79 (s, 1H), 10.7 (br s, 1H). ^13^C NMR (75 MHz, DMSO-*d*_6_), δ: (ppm) = 24.5, 29.0, 38.7–40.3 (DMSO-*d*_6_), 42.0, 47.0, 53.7, 122.1, 125.8, 128.6, 129.3, 131.1, 131.5, 145.6, 167.1.

### Synthesis of Heptacyclic Oligo-Heteroaryls 8, 14, 15, 16, 21, 22

To a solution of carboxylic acid (**7** or **13**, 1.0 equiv) in DMF (5 mL), 1′-carbonyldiimidazole (CDI; 1.5 equiv) was added, and the mixture was stirred at room temperature for 1 h. The corresponding diamidoxime (**2**, **10**, or **20**, 0.5 equiv) was added, and the solution was stirred at room temperature for 16 h. Then, potassium carbonate (1.2 equiv) was added, and the mixture was stirred at 60 °C for the appropriate amount of time (2 to 4 h when carboxylic acid **7** was used, 2 to 4 days when carboxylic acid **13** was used). The reaction was monitored with analytical HPLC (Gradient H_2_O + 0.1% TFA/MeCN), then the solvent was removed at reduced pressure. The residue was dissolved in 1% HCl and purified with preparative HPLC (Gradient H_2_O + 0.1% TFA/MeCN), and the collected fractions containing the product of interest were neutralized with a sat. aq. NaHCO_3_ solution and extracted with CHCl_3_ (3 × 50 mL). The combined organic phases were dried over Na_2_SO_4,_ and the solvent was removed at reduced pressure. 3,3′-(((pyridine-2,6-diylbis­(1,2,4-oxadiazole-3,5-diyl))­bis­(pyridine-6,2-diyl))­bis­(1*H*-1,2,3-triazole-4,1-diyl))­bis­(*N*,*N*-dimethylpropan-1-amine) 8. Light brown solid, isolated yield = 67%. ^1^H NMR (300 MHz, CDCl_3_), δ: (ppm) = 0.06 (TMS), 1.27 (impurity), 2.22–2.35 (qui, 4H, *J* = 6.8 Hz), 2.37 (s, 12H), 2.47–2.55 (t, 4H, *J* = 6.9 Hz), 2.92–2.98 (impurity), 4.56–4.64 (t, 4H, *J* = 6.8 Hz), 7.29 (CDCl_3_), 8.02–8.10 (t, 2H, *J* = 7.8 Hz), 8.10–8.20 (t, 1H, *J* = 7.9 Hz), 8.34–8.40 (d, 2H, *J* = 7.6 Hz), 8.42–8.50 (dd, 4H, *J* = 2.6, 7.9 Hz), 8.56 (s, 2H). ^13^C NMR (75 MHz, CDCl_3_), δ: (ppm) = 0.41–1.65 (TMS), 27.6, 29.6 (impurity), 44.9, 48.3, 55.7, 76.5–77.3 (CDCl_3_), 123.2, 123.3, 123.6, 125.1, 138.1, 138.4, 142.8, 146.8, 147.2, 151.5, 168.3, 175.2. ESI (MS) calculated for C_33_H_35_N_15_O_2_: 673.31 *m*/*z*; found: 1347.67 *m*/*z* ([2M + H]^+^), 674.58 *m*/*z* ([M + H]^+^), 337.83 *m*/*z* ([(M+2H)/2]^2+^). 3,3′-(((pyridine-2,6-diylbis­(1,2,4-oxadiazole-3,5-diyl))­bis­(3,1-phenylene))­bis­(1*H*-1,2,3-triazole-4,1-diyl))­bis­(*N*,*N*-dimethylpropan-1-amine) 14. White solid, isolated yield = 73%. ^1^H NMR (300 MHz, CDCl_3_), δ: (ppm) = 2.09–2.22 (m, 4H), 2.26 (s, 12H), 2.28–2.39 (t, 4H, *J* = 7.0 Hz), 4.49–4.58 (t, 4H, *J* = 6.9 Hz), 7.29 (CDCl_3_), 7.59–7.68 (t, 2H, *J* = 7.8 Hz), 8.05 (s, 2H), 8.05–8.14 (t, 1H, *J* = 7.6 Hz), 8.16–8.25 (m, 4H), 8.34–8.40 (d, 2H, *J* = 7.8 Hz), 8.67 (s, 2H). ^13^C NMR (75 MHz, CDCl_3_), δ: (ppm) = 28.0, 45.2, 48.2, 55.7, 76.6–77.4 (CDCl_3_), 120.8, 124.2, 125.0, 125.3, 127.5, 129.7, 129.9, 131.9, 138.3, 146.1, 146.9, 168.1, 176.2. ESI (MS) calculated for C_35_H_37_N_13_O_2_: 671.32 *m*/*z*; found: 1343.75 *m*/*z* ([2M + H]^+^), 672.58 *m*/*z* ([M + H]^+^), 336.75 *m*/*z* ([(M+2H)/2]^2+^). 3,3′-(((1,3-phenylenebis­(1,2,4-oxadiazole-3,5-diyl))­bis­(pyridine-6,2-diyl))­bis­(1*H*-1,2,3-triazole-4,1-diyl))­bis­(*N*,*N*-dimethylpropan-1-amine) 15. Off-white solid, isolated yield = 80%. ^1^H NMR (300 MHz, CDCl_3_), δ: (ppm) = 2.13–2.26 (qui, 4H, *J* = 6.7 Hz), 2.29 (s, 12H), 2.35–2.44 (t, 4H, *J* = 6.9 Hz), 4.53–4.62 (t, 4H, *J* = 6.9 Hz), 7.29 (CDCl_3_), 7.67–7.79 (t, 1H, *J* = 7.8 Hz), 8.02–8.11 (t, 2H, *J* = 7.8 Hz), 8.29–8.35 (d, 2H, *J* = 7.7 Hz), 8.39–8.51 (m, 6H), 9.11 (s, 1H). ^13^C NMR (75 MHz, CDCl_3_), δ: (ppm) = 14.0, 22.6, 28.0, 29.6 (impurity), 31.8, 45.2, 48.4, 55.8, 76.5–78.1 (CDCl_3_), 123.1, 123.2, 123.3, 126.8, 127.5, 129.5, 130.2, 138.1, 143.2, 147.2, 151.5, 168.6, 174.6. ESI (MS) calculated for C_34_H_36_N_14_O_2_: 672.31 *m*/*z*; found: 673.58 *m*/*z* ([M + H]^+^), 337.25 *m*/*z* ([(M+2H)/2]^2+^). 3,3′-(((1,3-phenylenebis­(1,2,4-oxadiazole-3,5-diyl))­bis­(3,1-phenylene))­bis­(1*H*-1,2,3-triazole-4,1-diyl))­bis­(*N*,*N*-dimethylpropan-1-amine) 16. White solid, isolated yield = 84%. ^1^H NMR (300 MHz, CDCl_3_), δ: (ppm) = 2.13–2.22 (qui, 4H, *J* = 6.8 Hz), 2.27 (s, 12H), 2.31–2.40 (t, 4H, *J* = 6.7 Hz), 4.51–4.59 (t, 4H, *J* = 6.8 Hz), 7.28 (CDCl_3_), 7.61–7.74 (m, 3H), 8.02 (s, 2H), 8.17–8.25 (t, 4H, *J* = 6.7 Hz), 8.33–8.40 (d, 2H, *J* = 7.8 Hz), 8.65 (s, 2H), 9.03 (s, 1H). ^13^C NMR (75 MHz, CDCl_3_), δ: (ppm) = 28.1, 45.2, 48.2, 55.7, 76.5–77.4 (CDCl_3_), 120.6, 124.6, 125.1, 126.6, 127.5, 127.7, 129.4, 129.7, 129.8, 130.0, 131.9, 146.2, 168.4, 175.6. ESI (MS) calculated for C_36_H_38_N_12_O_2_: 670.32 *m*/*z*; found: 1342.58 *m*/*z* ([2M + H]^+^), 671.58 *m*/*z* ([M + H]^+^), 336.33 *m*/*z* ([(M+2H)/2]^2+^). 3,3′-((((5-nitro-1,3-phenylene)­bis­(1,2,4-oxadiazole-3,5-diyl))­bis­(pyridine-6,2-diyl))­bis­(1*H*-1,2,3-triazole-4,1-diyl))­bis­(*N*,*N*-dimethylpropan-1-amine) 21. White solid, isolated yield = 79%. ^1^H NMR (300 MHz, CDCl_3_), δ: (ppm) = 1.25 (impurity), 2.14–2.24 (qui, 4H, *J* = 6.9 Hz), 2.27 (s, 12H), 2.32–2.41 (t, 4H, *J* = 6.5 Hz), 4.52–4.61 (t, 4H, *J* = 6.9 Hz), 7.28 (CDCl_3_), 8.00–8.09 (t, 2H, *J* = 7.8 Hz), 8.24–8.31 (d, 2H, *J* = 7.6 Hz), 8.43–8.48 (m, 4H), 9.19 (s, 2H), 9.32 (s, 1H). ^13^C NMR (75 MHz, CDCl_3_), δ: (ppm) = 28.1, 45.2, 48.5, 55.8, 76.5–77.4 (CDCl_3_), 123.3, 123.4, 124.7, 129.3, 131.4, 138.1, 142.7, 147.0, 149.0, 151.6, 167.0, 175.2. ESI (MS) calculated for C_34_H_35_N_15_O_4_: 717.30 *m*/*z*; found: 1435.25 *m*/*z* ([2M + H]^+^), 718.58 *m*/*z* ([M + H]^+^), 359.83 *m*/*z* ([(M+2H)/2]^2+^). 3,3′-((((5-nitro-1,3-phenylene)­bis­(1,2,4-oxadiazole-3,5-diyl))­bis­(3,1-phenylene))­bis­(1*H*-1,2,3-triazole-4,1-diyl))­bis­(*N*,*N*-dimethylpropan-1-amine) 22. Off-white solid, isolated yield = 77%. ^1^H NMR (300 MHz, CDCl_3_), δ: (ppm) = 1.12–2.10 (impurities), 2.17–2.30 (qui, 4H, *J* = 6.9 Hz), 2.33 (s, 12H), 2.42–2.50 (t, 4H, *J* = 6.9 Hz), 4.38 (br s, 3H), 4.52–4.61 (t, 4H, *J* = 6.9 Hz), 7.29 (CDCl_3_), 7.63–7.72 (t, 2H, *J* = 7.8 Hz), 8.07 (s, 2H), 8.18–8.25 (d, 4H, *J* = 7.9 Hz), 8.65 (s, 2H), 9.17 (s, 2H), 9.29 (s, 1H). ^13^C NMR (75 MHz, CDCl_3_), δ: (ppm) = 27.6, 44.8, 48.2, 55.5, 76.5–77.3 (CDCl_3_), 120.6, 124.1, 124.4, 125.2, 127.5, 129.6, 129.8, 130.2, 131.3, 132.0, 146.1, 166.8, 176.3. ESI (MS) calculated for C_36_H_37_N_13_O_4_: 715.31 *m*/*z*; found: 1432.33 *m*/*z* ([2M + H]^+^), 716.58 *m*/*z* ([M + H]^+^), 358.75 *m*/*z* ([(M+2H)/2]^2+^).

### Synthesis of Heptacyclic Oligo-Heteroaryls 23 and 24

A solution of the nitro derivative (**21** or **22**, 1.0 equiv) was dissolved in EtOH (5 mL) and 37% HCl (0.5 mL) was added. Subsequently, tin­(II) dichloride (6.0 equiv) was added in one portion, and the suspension was stirred at 85 °C for 6 h. The reaction was monitored with analytical HPLC (Gradient H_2_O + 0.1% TFA/MeCN). Upon completion, the solvent was removed at reduced pressure, and the residue was dissolved in 1 M NaOH and extracted with CHCl_3_ (3 × 20 mL). The combined organic phases were dried over Na_2_SO_4_, filtered, and concentrated in vacuo. The residue was dissolved in 1% HCl and purified with preparative HPLC (Gradient H_2_O + 0.1% TFA/MeCN), and the collected fractions containing the product of interest were neutralized with a sat. aq. NaHCO_3_ solution and extracted with CHCl_3_ (3 × 50 mL). The combined organic phases were dried over Na_2_SO_4,_ and the solvent was removed at reduced pressure. 3,3′-((((5-amino-1,3-phenylene)­bis­(1,2,4-oxadiazole-3,5-diyl))­bis­(pyridine-6,2-diyl))­bis­(1*H*-1,2,3-triazole-4,1-diyl))­bis­(*N*,*N*-dimethylpropan-1-amine) 23. White solid, isolated yield = 70%. ^1^H NMR (300 MHz, CDCl_3_), δ: (ppm) = 1.26 (impurity), 2.12–2.23 (qui, 4H, *J* = 6.9 Hz), 2.26 (s, 12H), 2.31–2.40 (t, 4H, *J* = 6.7 Hz), 4.15 (br s, 2H), 4.51–4.59 (t, 4H, *J* = 6.9 Hz), 7.28 (CDCl_3_), 7.68 (s, 2H), 7.97–8.06 (t, 2H, *J* = 7.8 Hz), 8.22–8.28 (d, 2H, *J* = 7.7 Hz), 8.39–8.46 (m, 3H). ^13^C NMR (75 MHz, CDCl_3_), δ: (ppm) = 28.2, 29.7, 45.4, 48.5, 55.9, 76.6–77.5 (CDCl_3_), 116.3, 116.9, 123.1, 123.2, 123.5, 128.4, 138.2, 143.3, 147.3, 147.5, 151.5, 168.8, 174.4. ESI (MS) calculated for C_34_H_37_N_15_O_2_: 687.33 *m*/*z*; found: 1375.58 *m*/*z* ([2M + H]^+^), 688.67 *m*/*z* ([M + H]^+^), 344.83 *m*/*z* ([(M+2H)/2]^2+^), 230.17 *m*/*z* ([(M+3H)/3]^3+^). 3,3′-((((5-amino-1,3-phenylene)­bis­(1,2,4-oxadiazole-3,5-diyl))­bis­(3,1-phenylene))­bis­(1*H*-1,2,3-triazole-4,1-diyl))­bis­(*N*,*N*-dimethylpropan-1-amine) 24. White solid, isolated yield = 77%. ^1^H NMR (300 MHz, CDCl_3_), δ: (ppm) = 0.09 (TMS), 0.85–1.50 (impurities), 2.10–2.25 (qui, 4H, *J* = 6.6 Hz), 2.28 (s, 12H), 2.32–2.40 (t, 4H, *J* = 6.9 Hz), 4.17 (br s, 2H), 4.51–4.60 (t, 4H, *J* = 6.9 Hz), 7.29 (CDCl_3_), 7.61–7.69 (m, 4H), 8.02 (s, 2H), 8.16–8.23 (m, 4H), 8.41 (s, 1H), 8.64 (s, 2H). ^13^C NMR (75 MHz, CDCl_3_), δ: (ppm) = 28.0, 29.6, 45.2, 48.2, 55.7, 76.5–77.3 (CDCl_3_), 116.0, 116.8, 120.6, 124.7, 125.1, 127.4, 128.5, 129.6, 129.7, 131.8, 146.2, 147.3, 168.5, 175.4. ESI (MS) calculated for C_36_H_39_N_13_O_2_: 685.33 *m*/*z*; found: 1372.58 *m*/*z* ([2M + H]^+^), 686.58 *m*/*z* ([M + H]^+^), 343.83 *m*/*z* ([(M+2H)/2]^2+^), 229.50 *m*/*z* ([(M+3H)/3]^3+^).

## Supplementary Material



## Notes

Synthetic procedures, molecular characterizations, and details about biophysical assays and molecular docking calculations (PDF)

## Abbreviations

CDI: carbonyldiimidazole
TFAA: trifluoroacetic anhydride
TMS-CCH: trimethylsilylacetylene
