# Supplementary material for: Water-Soluble Heptacyclic Oligo-Heteroaryls Targeting G‑Quadruplex DNA via Groove-Binding Interactions
Source: ACS Omega. 2026 Jul 18;11(30):45186–201. doi: 10.1021/acsomega.6c03686 (PMC13449210; doi:10.1021/acsomega.6c03686)
Supplement: Supplementary file 1 [file ao6c03686_si_001.pdf]

Water-soluble Heptacyclic Oligo-Heteroaryls Targeting G-Quadruplex DNA via Groove Binding Interactions

Giorgia Fracchioni,[1,#] Alessio M. Caramiello,[1,#] Matteo Nadai,[2] Sara Richter,[2,3] Valentina Pirota,[1,4]* and Filippo Doria[1,]*

[1] Department of Chemistry, University of Pavia, Pavia, Italy.

[2] Department of Molecular Medicine, University of Padova, Padova, Italy.

[3] Microbiology and Virology Unit, Padua University Hospital, 35128 Padua, Italy.

[4] G4-INTERACT, USERN, Pavia, Italy

MATERIALS AND METHODS 2

SYNTHETIC PROCEDURES 6

BIOPHYSICAL ASSAYS AND *IN SILICO* ANALYSIS 16

ANALYTICAL HPLC CHROMATOGRAMS 37

^{1}H AND ^{13}C NMR SPECTRA 41

MS SPECTRA FOR THE COMPOUNDS 8, 14-16, 21-24 66

Materials and Methods

Reagents, solvents, and chemicals were purchased from commercial sources (Alfa Aesar, Sigma-Aldrich, TCI, or Fluorochem) and used without further purification. The oligonucleotide sequences (Table S1) were purchased from Biomers as HPLC-purified compounds with >99% purity.

Table S1: *Sequences and topologies of the G-rich oligonucleotides analysed in this study. The table lists the names, sequences (5′→3′), and experimentally determined G-quadruplex topologies of each oligonucleotide. For each topology, the reference study is cited in the apex.*

Oligonucleotide	Sequence (5'-3')	Topology
hTel	TAGGGTTAGGGTTAGGGTTAGGG	Hybrid[1]
LTR III	GGTGAGTGGGTTTTGGGAGGGTGGG	Hybrid[2]
LTR IV	GGGCGGGACTGGGGAGTGGT	Parallel[3]
hTert	CGGGCGGGCGCGGTGGGGGCGGG	Parallel[4]
myc	TGAGGGTGGGTAGGGGTGGGTAA	Parallel[5]
Pu24T	TGAGGGTGGTGAGGGTGGGGAAGG	Parallel[6]
ds26	CAATCGGATCGAATTCGATCCGATTG	Double Strand
hairpin	CGCGAATTCGCGTTTCGCGAATTCGCG	Hairpin
ds12	CGCGAATTCGCG	Double strand
SS scr	GGATGTGAGTGTGAGTGTGAGG	Single strand
FAM-cMyc22-TAMRA	FAM-TGAGGGTGGGTAGGGGTGGGTAA-TAMRA	Parallel
FAM-LTRIV-TAMRA	FAM-GGGCGGGACTGGGGAGTGGT-TAMRA	Parallel

TLC analyses were conducted on silica gel (Merck 60F-254) with visualization at 254 and 366 nm. For flash column chromatography purification, Isolera ONE® Flash Chromatography System (MPLC, Biotage), combined with a tuneable wavelength UV-Vis detector, was used (SNAP KP-SIL 25 or 50 g columns, Biotage). All the syntheses assisted by microwaves were performed using a Discover microwave synthesizer (CEM). HPLC analyses were performed using an Agilent system SERIES 1260 with a SepaChrom ROBUSTA C18 column (3 μm, 4.6 x 50 mm) and a flow rate of 1.4 mL/min. The following method was used: isocratic elution over 2 min at 95% of 0.1% trifluoroacetic acid in water/5% acetonitrile, gradually increasing to 60% acetonitrile over 8 min, and then isocratic elution

for 4 min (λ = 256 nm). Compounds were purified on an Agilent Technologies 1260 Infinity preparative HPLC using a Waters XSelect® CSH Prep C18 OBDTM column (5 µm, 100 × 30 mm), with a 30 mL/min flow.

^{1}H- and ^{13}C-NMR spectra were recorded on a Bruker ADVANCE 200 MHz, Bruker AVANCE 300 MHz, or a Bruker ADVANCE 400 MHz spectrometer. UPLC-MS data were recorded using a Surveyor UHPLC system (Thermo Finnigan, San Jose, CA, USA) equipped with a BEH Acquity UPLC column (1.7 µm) 2.1 x 50 mm, and an LCQ ADV MAX ion-trap mass spectrometer, with an ESI ion source.

UV-Vis absorption experiments were performed on an Agilent Cary 60 spectrophotometer equipped with a Cary Single Cell Peltier temperature controller. Solutions of the heptacyclic ligands were prepared in water at increasing concentrations in the 2-20 µM range. Absorption spectra were recorded for each concentration under identical experimental conditions. For each ligand, the absorbance maximum was plotted as a function of concentration to evaluate compliance with Beer-Lambert behaviour.

Fluorescence intercalation displacement (FID) assay was performed using an Agilent Cary Eclipse spectrofluorometer. All oligonucleotides were diluted from stocks of 500 µM in water at a final concentration of 1 µM in lithium cacodylate buffer (10 mM, pH 7.2) containing 100 mM KCl. The solutions were annealed by heating at 95°C for 5 minutes, followed by slow cooling to room temperature over 4 hours. TO was added to each G4 solution at a final concentration of 2 µM (from a 10 mM stock in DMSO), and the samples were incubated for 30 minutes at room temperature to allow for equilibrium binding. Titrations were then performed by incremental addition of the test ligands (from 0 to 20 µM final concentration) with fluorescence recorded after each addition. Fluorescence emission was measured at 535 nm (excitation at 501 nm) using a 1 cm path length quartz cuvette. The percentage of TO displacement was calculated using the formula:

$$\text{Displacement}\,\% = \left[1 - \frac{\mathrm{F_{obs}}}{\mathrm{F_{TO}}}\right] \times\ 100$$

Where $\mathrm{F_{obs}}$ is the fluorescence intensity at a given ligand concentration, and $\mathrm{F_{TO}}$ is the fluorescence of the DNA-TO complex before ligand addition. The FID assay was performed for compounds **8**, **14-16**, and **21-24** using five G-quadruplex-forming sequences (hTel, LTR-III, LTR-IV, hTert, and myc) and one duplex DNA control (ds26). For comparison, titrations were also performed with the reference G4 ligand **NDIpip**, a tetrasubstituted naphthalenediimide.

Stock solutions of TO and the heptacyclic ligands were prepared in water and diluted to a final concentration of 2 µM in 10 mM lithium cacodylate buffer (pH=7.2) with 100 mM KCl. Absorption spectra of TO alone and of each individual ligand were first recorded separately. Subsequently, mixtures containing TO (2 µM) and each heptacyclic ligand (2 µM) were prepared under identical buffer conditions, and their absorption spectra were acquired. The experimental spectra of the

mixtures were then compared with the mathematical sum of the spectra of the corresponding individual components.

FRET-melting competition experiments were performed using FAM-TAMRA-labelled G4-forming oligonucleotides. The myc oligonucleotide was folded in 10 mM lithium cacodylate buffer (pH=7.2) containing 5 mM KCl and 95 mM LiCl, while LTR-IV was folded in 10 mM lithium cacodylate buffer (pH=7.2) containing 100 mM KCl. Oligonucleotide solutions were heated at 95 °C for 5 min and then cooled on ice for 30 min prior to use. Folded oligonucleotides (0.25 μM) were incubated with the heptacyclic ligands and **NDIpip** (1 μM) for 4 h at room temperature. Subsequently, competitor nucleic acid sequences (ds26, ds12, hairpin, and SS scr) were added, and the mixtures were further incubated for 4 h at room temperature. FRET-melting experiments were carried out in 96-well plates by monitoring fluorescence as a function of temperature from 20 to 95 °C, using a heating rate of 1 °C/min. Fluorescence measurements were acquired throughout the melting process, and melting temperatures (T_m) were determined from the resulting denaturation profiles using OriginPro 8.5.

Circular Dichroism spectra were recorded using a JASCO J-1500 spectropolarimeter (JASCO Corporation, Easton, MD, USA), equipped with a Peltier temperature controller, in a quartz cuvette with an optical path length of 1 cm. All oligonucleotides were diluted from 500 μM stock solutions in water to the final concentration (2 μM) in lithium cacodylate buffer (10 mM, pH 7.2) containing different concentrations of KCl (1 mM for myc and 100 mM for LTRIV). For myc, the ionic strength was kept constant by adding 99 mM of LiCl. The solutions were annealed by heating at 95°C for 5 min and gradually cooling to room temperature over 4 h. CD spectra were recorded at 293 K, with an instrument scanning speed of 200 nm/min, a response of 2 s over a wavelength range of 210–340 nm, and a 1 nm sampling interval. The reported spectra of each sample represent the average of 4 scans and are baseline-corrected for signal contributions due to the buffer mixture. CD spectra were normalized to Δε (M^{-1} cm^{-1}) according to the relationship:

$$\Delta\varepsilon = \frac{\vartheta}{32980\, C\, l}$$

where ϑ corresponds to observed ellipticities (in millidegrees), C is DNA molar concentration, and l is the path length in centimetres. For the CD-melting experiments of G4 structures (2 μM), the ellipticity was recorded at 264 nm for myc and LTRIV every 0.5°C, with a temperature scan rate of 1°C/min in the range of 20–95°C. Data were averaged over three independent experiments. Melting temperature values were calculated according to the Boltzmann equation applied for a two-state transition from a folded to unfolded state, assuming that the heat capacities of the folded and unfolded states are equal. ΔT_m was calculated as the difference between T_m in the presence and absence of the compounds. For CD and CD-melting studies, heptacyclic ligands were diluted from 10 mM DMSO stock solutions to a final concentration of 8 μM (4 equivalents relative to the oligonucleotide) and incubated for 4 hours with G4s at room temperature before measurements. In

combination experiments with TO, TO was first added from a 10 mM DMSO stock to a final concentration of 8 μM and incubated for 4 hours at room temperature with G4s. Then, the heptacyclic ligand was added under the same conditions (8 μM, 4 equivalents), followed by an additional 4-hour incubation before recording the CD spectrum and melting curve.

Docking studies were performed on the NMR-deposited structure of the cMyc22 (PDB:1XAV) and the LTRIV (PDB:2N4Y) G4 structures. Molecular docking calculations were carried out using AutoDock 4 with the aid of its graphical user interface, AutoDockTools 1.5.7. The ligand and DNA oligonucleotides were prepared by use of AutoDock Tools 1.5.7 by assigning bond orders, adding hydrogen atoms, and generating the appropriate protonation states. The ligand and DNA oligonucleotides were then converted to proper Autodock PDBQT file formats, and the Gasteiger charges were assigned. The number of torsions was set as the total number of rotatable bonds in both ligands, and specifically, TORSDOF = 14. The 3D grid box dimensions were defined, including the whole DNA macromolecule. The docking area was centered on the DNA center of mass, and grid boxes of 33 Å × 24 Å × 31 Å for cMyc22 and 27 Å × 27 Å × 27 Å for LTRIV G4s were used, respectively, with a 0.603 Å and 0.681 Å spacing. One hundred docking poses were generated by using as docking parameters seed = random, exhaustiveness = 24, and number of binding modes = 20 for each of the three runs performed for both DNA/ligand systems. Docking poses were clustered on the basis of their root-mean square deviation (RMSD) and ranked based on their binding energy. Molecular modeling figures were drawn by PyMOL.

UV–Vis absorption measurements were performed on an Agilent Cary 60 spectrophotometer equipped with a Cary Single Cell Peltier temperature controller. Titration experiments were carried out using a fixed concentration of LTR-IV G4 (2 μM) and increasing concentrations of the heptacyclic ligands (0-10 μM) in 10 mM lithium cacodylate buffer (pH=7.2) containing 100 mM KCl. After preparation, samples were incubated for 4 h at room temperature to allow equilibration of the ligand–G4 complexes prior to spectral acquisition. To account for the intrinsic absorbance contribution of the ligands, independent UV-Vis titrations of each ligand were performed under identical experimental conditions and over the same concentration range (0–10 μM) in the absence of LTR-IV. The corresponding spectra were mathematically subtracted from those recorded for the ligand–G4 mixtures, yielding spectra associated with the interaction between the ligand and the G4 target. Titration data of G4-ligand species were processed with the Hyperquad package by determining the log K values from the plots of absorbance values, corrected for dilution.

Synthetic Procedures

Synthesis of *N*2,*N*6-dihydroxypyridine-2,6-bis(carboximidamide) 2

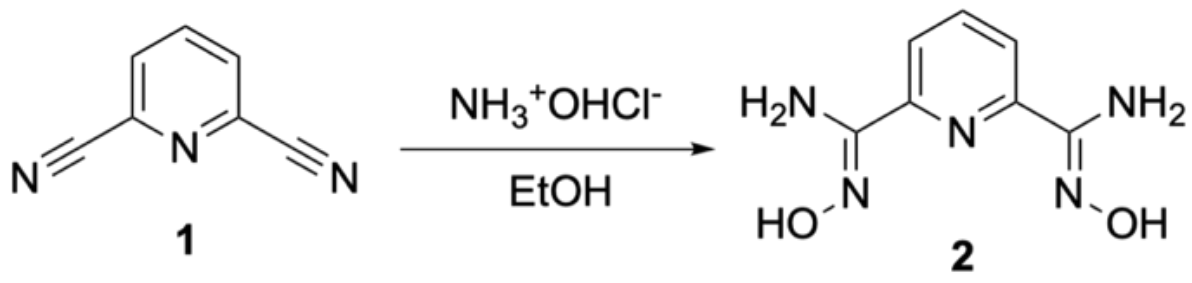

To a solution of **1** (3.87 g, 30 mmol, 1.0 eq.) in 100 mL EtOH, were added 100 mL of a 0.7M aqueous solution of hydroxylamine hydrochloride in water (4.86 g, 70 mmol, 2.3 eq.) previously neutralized with solid sodium hydroxide (2.8 g, 70 mmol, 2.3 eq.). The reaction mixture was stirred at 70°C for 30 min, then cooled to 10°C. (RP-TLC H_2O:MeCN 6:4 + 0.1% TFA, R_f(**1**) = 0.16, R_f(**2**) = 0.42). The resulting solid was filtered and washed with water. The product was obtained as a crystalline solid without further purification. 5.21 g, 89% yield.

*N*2,*N*6-dihydroxypyridine-2,6-bis(carboximidamide) **2**: ^{1}H-NMR (300MHz, DMSO-*d6*), δ (ppm) = 2.51 (DMSO-*d6*), 3.41 (DMSO-*d6*), 6.31 (bs, 4H), 7.75-7.89 (m, 3H), 9.88 (s, 2H). ^{13}C-NMR (75MHz, DMSO-*d6*), δ (ppm) = 119.2, 136.7, 149.0, 149.6.

Synthesis of *N'*1,*N'*3-dihydroxyisophthalimidamide 10

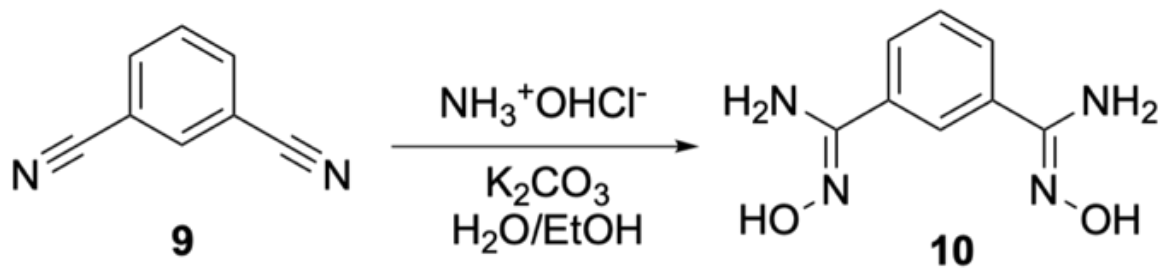

To a solution of **9** (1.06 g, 8.3 mmol, 1.0 eq.) in 40 mL of ethanol, were added 25 mL of a 0.8 M solution of hydroxylamine hydrochloride (1.45 g, 20.9 mmol, 2.5 eq.) in water previously neutralized with potassium carbonate (1.46 g, 10.6 mmol, 1.3 eq.). The reaction was stirred at reflux for 16h (TLC $CHCl_3$:MeOH 8:2, R_f(**9**) = 0.91, R_f(**monosub.**) = 0.85, R_f(**10**) = 0.40). The volatiles were removed at reduced pressure, and the crude was purified using FCC (Gradient $CHCl_3$:MeOH). Pale-yellow crystals, 1.39 g, 86% yield.

*N*1,*N*3-dihydroxyisophthalimidamide **10**: ^{1}H-NMR (300MHz, CD_3OD), δ (ppm) = 7.37-7.48 (t, 1H), 7.65-7.74 (m, 2H), 7.90-7.95 (s, 1H).

Synthesis of 5-nitroisophthalamide 18

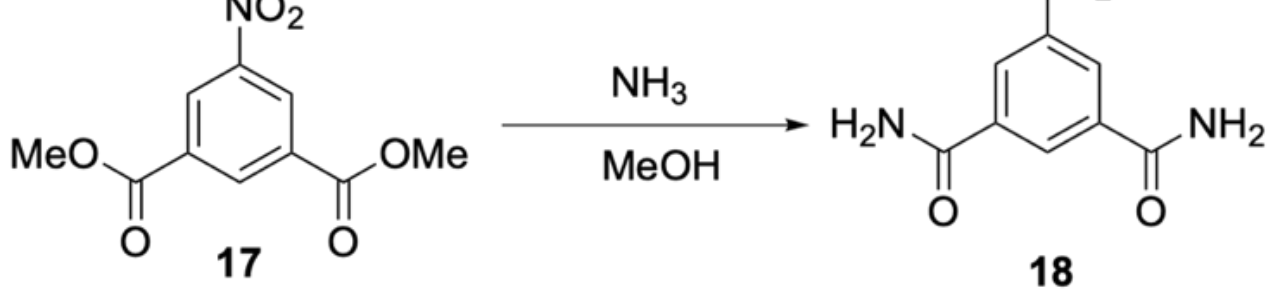

Dimethyl 5-nitroisophthalate **17** (2.51 g, 10.0 mmol, 1.0 eq.) was dissolved in methanol (10 mL). A 7 M solution of ammonia in methanol (10 mL, 70.0 mmol, 7.0 eq.) was added and stirred at room temperature for 16 h (TLC $CHCl_3$:MeOH 9:1, R_f(**17**) = 0.95, R_f(**18**) = 0.20). Upon completion, water

(100 mL) was added and the resulting precipitate was filtered and washed with more water (3 x 50 mL), dried, and recrystallized from ethanol/water. Yellow solid, 1.93 g, 88% yield.

5-nitroisophthalamide **18**: ^{1}H-NMR (300MHz, DMSO-*d6*), δ (ppm) = 7.80 (bs, 2H), 8.43 (bs, 2H), 8.81 (s, 3H). ^{13}C-NMR (75MHz, DMSO-*d6*), δ (ppm) = 38.6-40.2 (DMSO-*d6*), 124.5, 132.7, 136.0, 147.9, 165.4.

Synthesis of 5-nitroisophthalonitrile 19

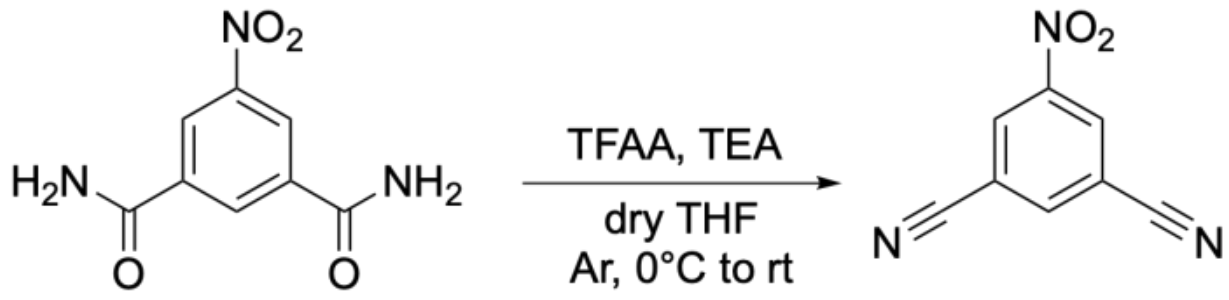

18 **19**

18 (1.16 g, 5.54 mmol, 1.0 eq.) was dissolved in dry THF (30 mL) under Ar atmosphere and cooled to 0°C. Triethylamine (2.3 mL, 16.6 mmol, 3.0 eq.) was added, followed by a dropwise addition of trifluoroacetic anhydride (2.3 mL, 16.6 mmol, 3.0 eq.) over a 1h period. After this, the mixture was allowed to warm up at room temperature and stirred for 3 hours (TLC $CHCl_3$:MeOH 9:1, R_f(**18**) = 0.20, R_f(**19**) = 0.86). Upon completion, the mixture was cooled to 0°C and quenched with 20 mL of sat. aq. $NaHCO_3$. The volatiles were removed at reduced pressure, and the product was extracted with DCM (3 x 20 mL). The combined organic phases were dried over $MgSO_4$, filtered, and concentrated. The crude was purified using FCC (Gradient Cyclohexane: $CHCl_3$). Yellow solid, 777 mg, 82% yield.

5-nitroisophthalonitrile **19**: ^{1}H-NMR (300MHz, DMSO-*d6*), δ (ppm) = 2.60 (DMSO-*d6*), 3.40 (DMSO-*d6*), 9.01 (s, 1H), 9.17 (s, 2H). ^{13}C-NMR (75MHz, DMSO-*d6*), δ (ppm) = 38.7-40.4(DMSO-d6), 114.1, 115.7, 131.8, 142.1, 148.1.

Synthesis of *N'*^{1},*N'*^{3}-dihydroxy-5-nitroisophthalimidamide 20

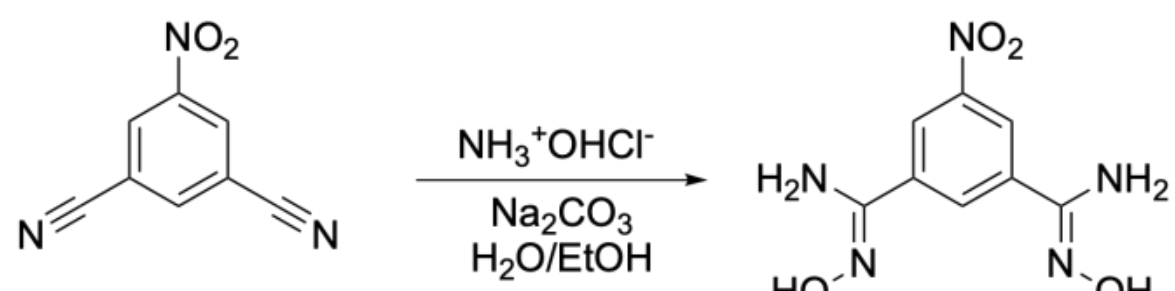

19 **20**

To a solution of **19** (1.85 g, 10.7 mmol, 1.0 eq.) in 25 mL of ethanol were added 25 mL of a 1.0 M solution of hydroxylamine hydrochloride (1.45 g, 25.1 mmol, 2.3 eq.) in water previously neutralized with sodium carbonate (1.33 g, 12.5 mmol, 1.2 eq.). The reaction was stirred at room temperature for 16h (TLC $CHCl_3$:MeOH 8:2, R_f(**19**) = 0.86, R_f(**20**) = 0.31). The vessel was cooled in an ice bath, and the crystallized solid was filtered, washed with water (3 x 50 mL), dried, and recrystallized from ethanol/water. Yellow crystals, 2.20 g, 86% yield.

N'^{1},*N'*^{3}-dihydroxy-5-nitroisophthalimidamide **20**: ^{1}H-NMR (300MHz, CD_3OD), δ (ppm) = 2.50 (DMSO-*d6*), 3.42 (DMSO-*d6*), 6.13 (s, 4H), 8.42 (s, 1H), 8.49 (s, 2H), 10.02 (s, 2H). ^{13}C-NMR (75MHz, DMSO-*d6*), δ (ppm) = 38.6-40.3(DMSO-d6), 120.0, 128.2, 134.9, 147.7, 149.1.

Synthesis of 3-azido-*N,N*-dimethylpropan-1-aminium chloride 4

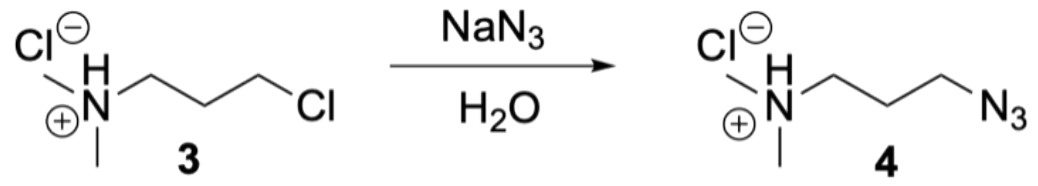

3 (1.8 g, 13.9 mmol, 1.0 eq.) was dissolved in 50 mL of water. NaN_3 (2.7 g, 41.7 mmol, 3.0 eq.) was added, then the mixture was heated at 80°C and stirred overnight. Upon completion (GC-MS), the mixture was neutralized at 0°C with 2M NaOH (6 mL), then extracted with Et_2O (3 x 50 mL). HCl gas was bubbled in the ethereal mixture to form the hydrochloride salt, then the solvent was evaporated. Pale yellow oil, 1.6 g, quantitative yield.

3-azido-*N,N*-dimethylpropan-1-aminium chloride **4**: ^{1}H-NMR (300MHz, CD_3OD), δ (ppm) = 1.99-2.11 (qui, 2H, *J* = 11.6 Hz, 8.0 Hz, 4.1 Hz,), 2.93 (s, 6H), 3.22-3.37 (m, 2H), 3.50-3.60 (t, 2H, *J* = 9.9 Hz), 5.21 (s, 3H).

Synthesis of methyl 6-((trimethylsilyl)ethynyl)picolinate 5a

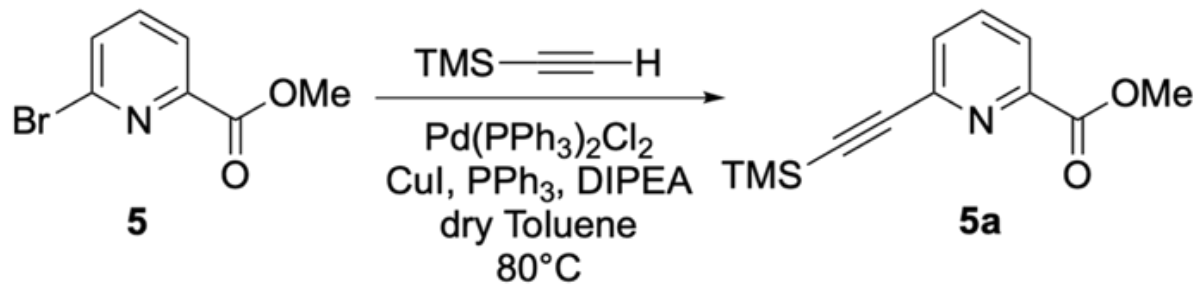

To a solution of methyl 6-bromopicolinate (**5**) (300 mg, 1.39 mmol, 1.0 eq.) in 7 mL dry toluene under nitrogen atmosphere were added in sequence copper iodide (6.0 mg, 0.032 mmol, 0.02 eq.), triphenylphosphine (15.0 mg, 0.022 mmol, 0.016 eq.), DIPEA (2.5 ml, 14.4 mmol, 10.3 eq.) and palladium (II) bis(triphenylphosphine) dichloride (20.0 mg, 0.029 mmol, 0.02 eq.). The resulting pale-yellow solution was stirred for 10 minutes at room temperature, then trimethylsilyl acetylene (290 µL, 2.04 mmol, 1.5 eq.) was added, and the mixture was heated at 80°C and stirred for 1 hour under a nitrogen atmosphere (TLC Cyclohexane:AcOEt 8:2, R_f(**5**) = 0.28, R_f(**5a**) = 0.39). Upon completion, the mixture was filtered, and the organic solvent was washed with water (3 x 10 mL), dried over Na_2SO_4, filtered, and concentrated. The product was used without any further purification. Pale orange oil, 300 mg, 92% yield.

Methyl 6-((trimethylsilyl)ethynyl)picolinate **5a**: ^{1}H-NMR (300MHz, $CDCl_3$), δ (ppm) = 0.11 (s, 9H), 3.84 (s, 3H), 7.45-7.51 (d, 1H, *J* = 7.8 Hz), 7.64-7.73 (t, 1H, *J* = 7.8 Hz), 7.86-7.93 (d, 1H, *J* = 7.8 Hz). ^{13}C-NMR (75MHz, $CDCl_3$), δ (ppm) = -0.62, 52.6, 77.0 ($CDCl_3$), 96.0, 102.7, 124.0, 130.3, 137.0, 143.0, 148.0, 164.9.

Synthesis of methyl 6-ethynylpicolinate 6

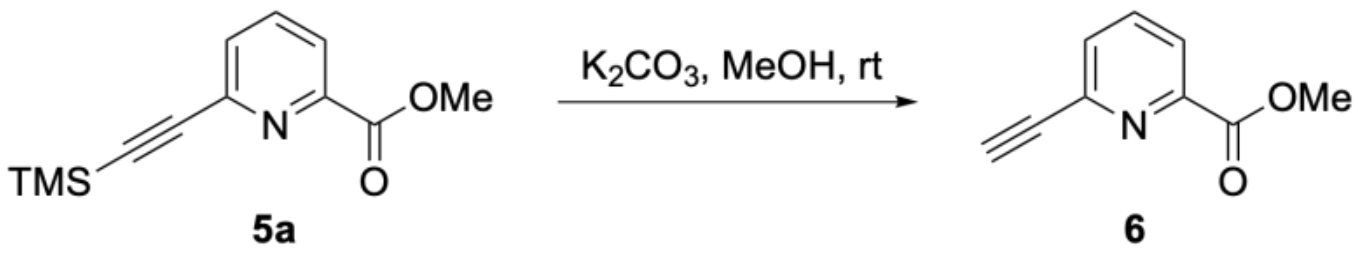

5a (1.37 g, 5.9 mmol, 1.0 eq.) was dissolved in MeOH (100 mL), and K_2CO_3 (200 mg, 1.5 mmol, 0.25 eq.) was added. The mixture was stirred at room temperature for 2 hours (TLC

Cyclohexane:AcOEt 8:2, R_f(**5a**) = 0.39, R_f(**6**) = 0.25) and upon completion, the solvent was removed at reduced pressure. The crude was purified using FCC (Cyclohexane:AcOEt 8:2). Yellow solid, 827 mg, 87% yield.

Methyl 6-ethynylpicolinate **6**: ^{1}H-NMR (300MHz, $CDCl_3$), δ (ppm) = 3.21 (s, 1H), 4.00 (s, 3H), 7.62-7.68 (d, 1H, *J* = 7.8 Hz), 7.79-7.88 (t, 1H, *J* = 7.8 Hz), 8.07-8.13 (d, 1H, *J* = 7.8 Hz). ^{13}C-NMR (75MHz, $CDCl_3$), δ (ppm) = 52.9, 76.5-77.3 ($CDCl_3$), 78.4, 81.9, 124.5, 128.3 (impurity), 130.4, 131.9 (impurity), 137.2, 142.4, 148.3, 165.0.

Synthesis of methyl 3-((trimethylsilyl)ethynyl)benzoate 11a

The compound was synthesized according to the literature.[7]

Synthesis of methyl 3-ethynylbenzoate 12

The compound was synthesized according to the literature.[8]

Synthesis of methyl 6-(1-(3-(dimethylamino)propyl)-1*H*-1,2,3-triazol-4-yl)picolinate 6a

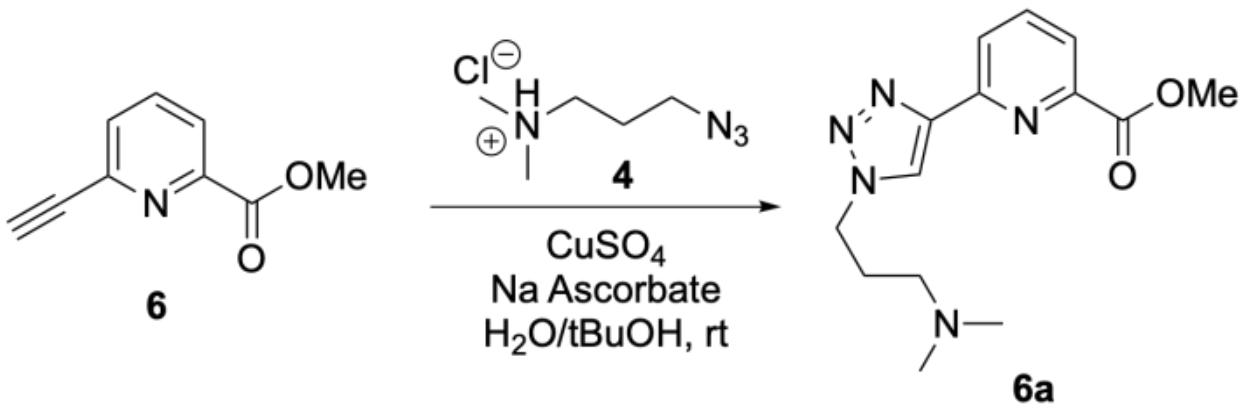

To a solution of **6** (200 mg, 1.24 mmol, 1.0 eq) in tBuOH (5 mL), were added at room temperature in sequence copper sulphate pentahydrate (31.0 mg, 0.12 mmol, 0.1 eq.), sodium ascorbate (246 mg, 1.24 mmol, 1.0 eq.) and a solution of **4** (226 mg, 1.37 mmol, 1.1 eq.) in water (5 mL). The mixture was stirred for 24h at room temperature (TLC $CHCl_3$:MeOH 98:2, R_f(**6**) = 0.83, R_f(**6a**) = 0.21). Upon completion, the organic solvent was removed at reduced pressure, and the residue was treated with sat. aq. $NaHCO_3$ (10 mL) and extracted with DCM (3 x 15 mL). The organic phases were joined, dried over Na_2SO_4, filtered, and concentrated. The crude was purified using FCC (Gradient $CHCl_3$:MeOH). Yellowish oil, 305 mg, 85% yield.

Methyl 6-(1-(3-(dimethylamino)propyl)-1*H*-1,2,3-triazol-4-yl)picolinate **6a**: ^{1}H-NMR (300MHz, $CDCl_3$), δ (ppm) = 2.10-2.21 (qui, 2H, *J* = 6.8 Hz), 2.25 (s, 6H), 2.30-2.39 (t, 2H, *J* = 6.8 Hz), 4.02 (s, 3H), 4.48-4.57 (t, 2H, *J* = 7.0 Hz), 7.90-7.99 (t, 1H, *J* = 7.8 Hz), 8.02-8.09 (d, 1H, *J* = 7.7 Hz), 8.36 (s, 1H), 8.36-8.42 (d, 1H, *J* = 7.9 Hz). ^{13}C-NMR (75MHz, $CDCl_3$), δ (ppm) = 28.0, 45.2, 48.2, 52.6, 55.7, 76.6-77.5 ($CDCl_3$), 122.9, 123.2, 123.9, 137.7, 147.3, 147.5, 150.7, 165.4.

Synthesis of methyl 3-(1-(3-(dimethylamino)propyl)-1*H*-1,2,3-triazol-4-yl)benzoate 12a

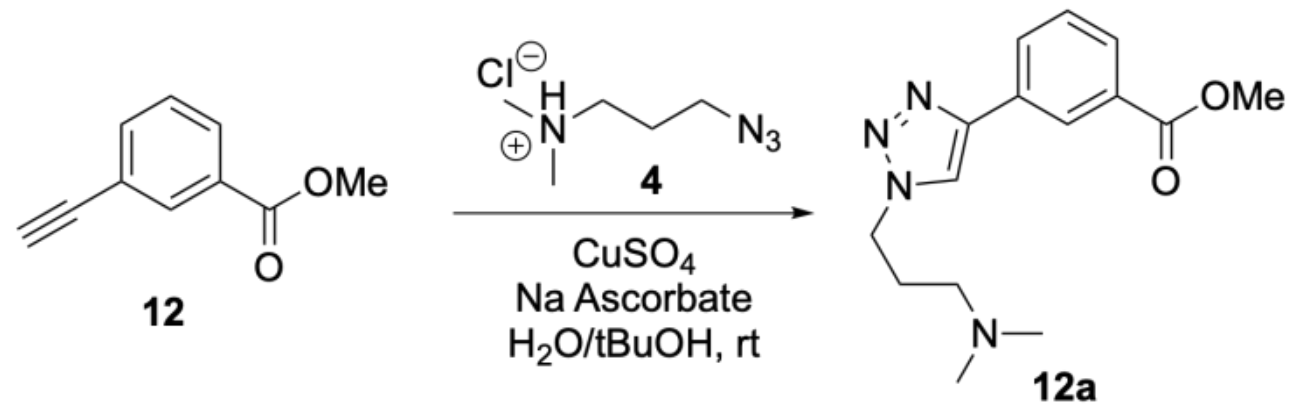

To a solution of **12** (199 mg, 1.24 mmol, 1.0 eq.) in tBuOH (5 mL), were added at room temperature in sequence copper sulphate pentahydrate (31.0 mg, 0.12 mmol, 0.1 eq.), sodium ascorbate (246 mg, 1.24 mmol, 1.0 eq.) and a solution of **4** (226 mg, 1.37 mmol, 1.1 eq.) in water (5 mL). The mixture was stirred for 24h at room temperature (TLC $CHCl_3$:MeOH 98:2, R_f(**12**) = 0.80, R_f(**12a**) = 0.22). Upon completion, the organic solvent was removed at reduced pressure, and the residue was treated with sat. aq. $NaHCO_3$ (10 mL) and extracted with DCM (3 x 15 mL). The organic phases were joined, dried over Na_2SO_4, filtered, and concentrated. The crude was purified using FCC (Gradient $CHCl_3$:MeOH). Yellowish oil, 340 mg, 95% yield.

Methyl 3-(1-(3-(dimethylamino)propyl)-1*H*-1,2,3-triazol-4-yl)benzoate **12a**: ^{1}H-NMR (300MHz, $CDCl_3$), δ (ppm) = 2.07-2.18 (qui, 2H, *J* = 6.8 Hz), 2.24 (s, 6H), 2.26-2.35 (t, 2H, *J* = 6.8 Hz), 3.94 (s, 3H), 4.45-4.54 (t, 2H, *J* = 6.9 Hz), 7.46-7.55 (t, 1H, *J* = 7.8 Hz), 7.89 (s, 1H), 7.96-8.03 (d, 1H, *J* = 7.8 Hz), 8.08-8.14 (d, 1H, *J* = 7.8 Hz), 8.42 (s, 1H). ^{13}C-NMR (75MHz, $CDCl_3$), δ (ppm) = 28.0, 45.2, 48.1, 52.1, 55.6, 76.5-77.4 ($CDCl_3$), 120.3, 126.6, 128.9, 129.9, 130.6, 131.0, 146.6, 166.7.

Synthesis of 6-(1-(3-(dimethylamino)propyl)-1*H*-1,2,3-triazol-4-yl)picolinic acid 7

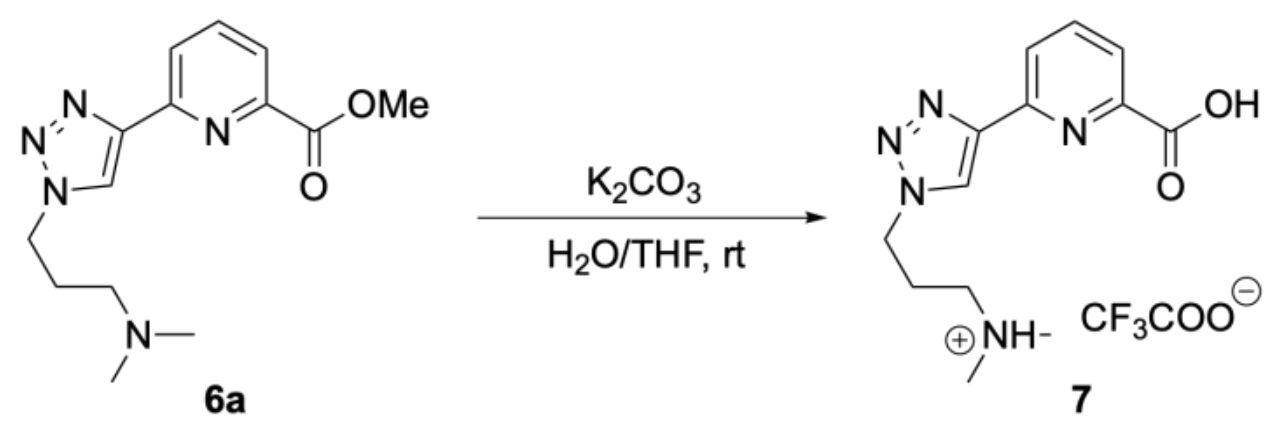

6a (333 mg, 1.15 mmol, 1.0 eq.) and potassium carbonate (238 mg, 1.75 mmol, 1.5 eq.) were dissolved in a 1:1 water:THF mixture (30 mL). The reaction was heated to reflux for 2 hours, after which the solvent was removed at reduced pressure. The crude was purified from inorganic salts using preparative HPLC (H_2O + 0.1% TFA:MeCN). White solid, 448 mg, quantitative yield (TFA salt).

6-(1-(3-(dimethylamino)propyl)-1*H*-1,2,3-triazol-4-yl)picolinic acid **7**: ^{1}H-NMR (300MHz, DMSO-*d6*), δ (ppm) = 2.26-2.39 (qui, 2H, *J* = 8.0 Hz), 2.50 (DMSO-*d6*), 2.78 (s, 6H), 3.07-3.16 (m, 2H), 4.54-4.62 (t, 2H, *J* = 6.8 Hz), 7.96-8.03 (d, 1H, *J* = 7.6 Hz), 8.05-8.14 (t, 1H, *J* = 7.7 Hz), 8.22-8.28 (d, 1H, *J* = 7.7 Hz), 8.81 (s, 1H). ^{13}C-NMR (75MHz, DMSO-*d6*), δ (ppm) = 24.6, 38.7-40.4 (DMSO-*d6*), 42.1, 47.0, 53.9, 122.5, 123.6, 124.2, 138.6, 146.7, 148.3, 149.9, 158.5, 165.8.

Synthesis of 3-(1-(3-(dimethylamino)propyl)-1*H*-1,2,3-triazol-4-yl)benzoic acid 13

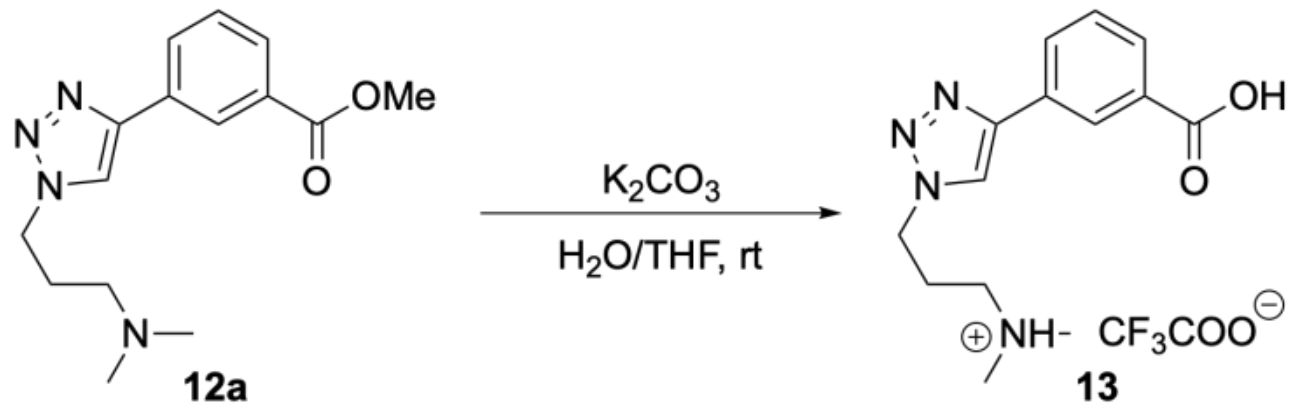

12a (332 mg, 1.15 mmol, 1.0 eq.) and potassium carbonate (238 mg, 1.75 mmol, 1.5 eq.) were dissolved in a 1:1 water:THF mixture (30 mL). The reaction was heated to reflux for 16 hours, after which the solvent was removed at reduced pressure. The crude was purified from inorganic salts using preparative HPLC (H_2O + 0.1% TFA:MeCN) and a recrystallisation from iPrOH. White solid, 447 mg, quantitative yield (TFA salt).

3-(1-(3-(dimethylamino)propyl)-1*H*-1,2,3-triazol-4-yl)benzoic acid **13**: ^{1}H-NMR (300MHz, DMSO-*d6*), δ (ppm) = 1.23 (impurity), 2.36-2.30 (qui, 2H, *J* = 6.8 Hz), 2.50 (DMSO-*d6*), 2.74 (s, 6H), 3.05-3.14 (m, 2H), 3.37 (H_2O), 4.51-4.59 (t, 2H, *J* = 6.8 Hz), 7.56-7.65 (t, 1H, *J* = 7.7 Hz), 7.88-7.95 (d, 1H, *J* = 7.8 Hz), 8.06-8.13 (d, 1H, *J* = 7.7 Hz), 8.42 (s, 1H), 8.79 (s, 1H), 10.7 (bs, 1H). ^{13}C-NMR (75MHz, DMSO-*d6*), δ (ppm) = 24.5, 29.0, 38.7-40.3 (DMSO-*d6*), 42.0, 47.0, 53.7, 122.1, 125.8, 128.6, 129.3, 131.1, 131.5, 145.6, 167.1.

Synthesis of heptacyclic oligo-heteroaryls 8, 14, 15, 16, 21, 22

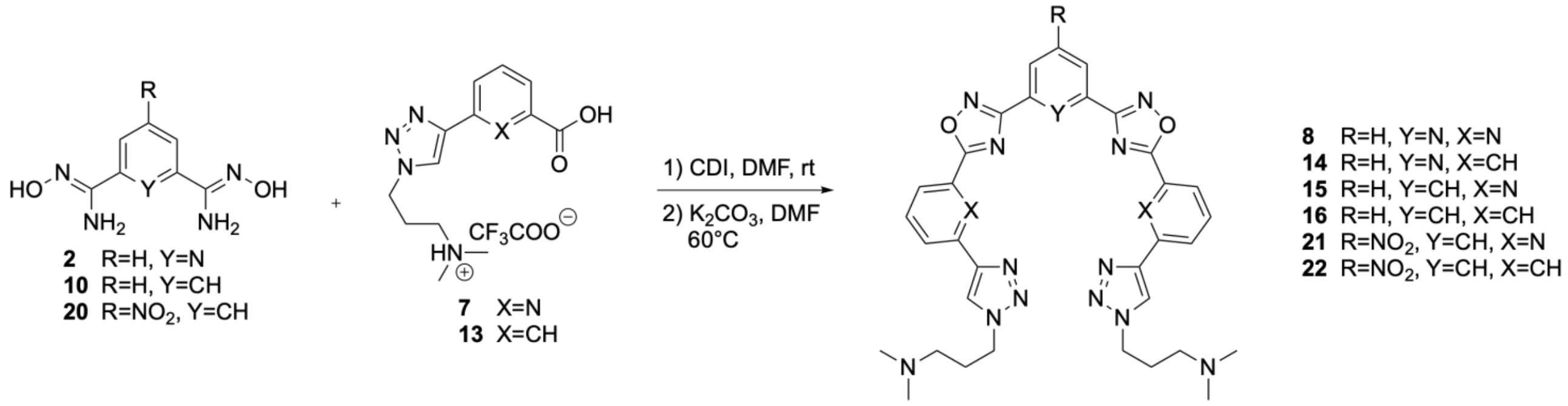

General procedure

To a solution of carboxylic acid (**7** or **13**, 1.0 eq.) in DMF (5 mL) was added 1'-carbonyldiimidazole (CDI; 1.5 eq.), and the mixture was stirred at room temperature for 1 hour. The corresponding diamidoxime (**2**, **10**, or **20**, 0.5 eq.) was added, and the solution was stirred at room temperature for 16 hours. Hence, potassium carbonate (1.2 eq.) was added, and the mixture was stirred at 60°C for the appropriate amount of time (2 to 4 hours when carboxylic acid **7** was used, 2 to 4 days when carboxylic acid **13** was used). The reaction was monitored with analytical HPLC (Gradient H_2O + 0.1% TFA:MeCN), then the solvent was removed at reduced pressure. The residue was dissolved in 1% HCl and purified with preparative HPLC (Gradient H_2O + 0.1% TFA:MeCN), and the collected fractions containing the product of interest were neutralized with a sat. aq. $NaHCO_3$ solution and

extracted with $CHCl_3$ (3 x 50 mL). The combined organic phases were dried over Na_2SO_4, and the solvent was removed at reduced pressure.

3,3'-(((pyridine-2,6-diylbis(1,2,4-oxadiazole-3,5-diyl))bis(pyridine-6,2-diyl))bis(1*H*-1,2,3-triazole-4,1-diyl))bis(*N*,*N*-dimethylpropan-1-amine) 8

Light brown solid, isolated yield = 67%. ^{1}H-NMR (300MHz, $CDCl_3$), δ (ppm) = 0.06 (TMS), 1.27 (impurity), 2.22-2.35 (qui, 4H, *J* = 6.8 Hz), 2.37 (s, 12H), 2.47-2.55 (t, 4H, *J* = 6.9 Hz), 2.92-2.98 (impurity), 4.56-4.64 (t, 4H, *J* = 6.8 Hz), 7.29 ($CDCl_3$), 8.02-8.10 (t, 2H, *J* = 7.8 Hz), 8.10-8.20 (t, 1H, *J* = 7.9 Hz), 8.34-8.40 (d, 2H, *J* = 7.6 Hz), 8.42-8.50 (dd, 4H, *J* = 2.6, 7.9 Hz), 8.56 (s, 2H). ^{13}C-NMR (75MHz, $CDCl_3$), δ (ppm) = 0.41-1.65 (TMS), 27.6, 29.6 (impurity), 44.9, 48.3, 55.7, 76.5-77.3 ($CDCl_3$), 123.2, 123.3, 123.6, 125.1, 138.1, 138.4, 142.8, 146.8, 147.2, 151.5, 168.3, 175.2.
ESI (MS) calculated for $C_{33}H_{35}N_{15}O_2$: 673.31 m/z; found: 1347.67 m/z ($[2M+H]^+$), 674.58 m/z ($[M+H]^+$), 337.83 m/z ($[(M+2H)/2]^{2+}$).

3,3'-(((pyridine-2,6-diylbis(1,2,4-oxadiazole-3,5-diyl))bis(3,1-phenylene))bis(1*H*-1,2,3-triazole-4,1-diyl))bis(*N*,*N*-dimethylpropan-1-amine) 14

White solid, isolated yield = 73%. ^{1}H-NMR (300MHz, $CDCl_3$), δ (ppm) = 2.09-2.22 (m, 4H), 2.26 (s, 12H), 2.28-2.39 (t, 4H, *J* = 7.0 Hz), 4.49-4.58 (t, 4H, *J* = 6.9 Hz), 7.29 ($CDCl_3$), 7.59-7.68 (t, 2H, *J* = 7.8 Hz), 8.05 (s, 2H), 8.05-8.14 (t, 1H, *J* = 7.6 Hz), 8.16-8.25 (m, 4H), 8.34-8.40 (d, 2H, *J* = 7.8 Hz), 8.67 (s, 2H). ^{13}C-NMR (75MHz, $CDCl_3$), δ (ppm) = 28.0, 45.2, 48.2, 55.7, 76.6-77.4 ($CDCl_3$), 120.8, 124.2, 125.0, 125.3, 127.5, 129.7, 129.9, 131.9, 138.3, 146.1, 146.9, 168.1, 176.2.
ESI (MS) calculated for $C_{35}H_{37}N_{13}O_2$: 671.32 m/z; found: 1343.75 m/z ($[2M+H]^+$), 672.58 m/z ($[M+H]^+$), 336.75 m/z ($[(M+2H)/2]^{2+}$).

3,3'-(((1,3-phenylenebis(1,2,4-oxadiazole-3,5-diyl))bis(pyridine-6,2-diyl))bis(1*H*-1,2,3-triazole-4,1-diyl))bis(*N*,*N*-dimethylpropan-1-amine) 15

Off-white solid, isolated yield = 80%. ^{1}H-NMR (300MHz, $CDCl_3$), δ (ppm) = 2.13-2.26 (qui, 4H, *J* = 6.7 Hz), 2.29 (s, 12H), 2.35-2.44 (t, 4H, *J* = 6.9 Hz), 4.53-4.62 (t, 4H, *J* = 6.9 Hz), 7.29 ($CDCl_3$), 7.67-7.79 (t, 1H, *J* = 7.8 Hz), 8.02-8.11 (t, 2H, *J* = 7.8 Hz), 8.29-8.35 (d, 2H, *J* = 7.7 Hz), 8.39-8.51 (m, 6H), 9.11 (s, 1H). ^{13}C-NMR (75MHz, $CDCl_3$), δ (ppm) = 14.0, 22.6, 28.0, 29.6 (impurity), 31.8, 45.2, 48.4, 55.8, 76.5-78.1 ($CDCl_3$), 123.1, 123.2, 123.3, 126.8, 127.5, 129.5, 130.2, 138.1, 143.2, 147.2, 151.5, 168.6, 174.6.
ESI (MS) calculated for $C_{34}H_{36}N_{14}O_2$: 672.31 m/z; found: 673.58 m/z ($[M+H]^+$), 337.25 m/z ($[(M+2H)/2]^{2+}$).

3,3'-(((1,3-phenylenebis(1,2,4-oxadiazole-3,5-diyl))bis(3,1-phenylene))bis(1*H*-1,2,3-triazole-4,1-diyl))bis(*N*,*N*-dimethylpropan-1-amine) 16

White solid, isolated yield = 84%. ^{1}H-NMR (300MHz, $CDCl_3$), δ (ppm) = 2.13-2.22 (qui, 4H, *J* = 6.8 Hz), 2.27 (s, 12H), 2.31-2.40 (t, 4H, *J* = 6.7 Hz), 4.51-4.59 (t, 4H, *J* = 6.8 Hz), 7.28 ($CDCl_3$), 7.61-7.74 (m, 3H), 8.02 (s, 2H), 8.17-8.25 (t, 4H, *J* = 6.7 Hz), 8.33-8.40 (d, 2H, *J* = 7.8 Hz), 8.65 (s, 2H), 9.03 (s, 1H). ^{13}C-NMR (75MHz, $CDCl_3$), δ (ppm) = 28.1, 45.2, 48.2, 55.7, 76.5-77.4 ($CDCl_3$), 120.6, 124.6, 125.1, 126.6, 127.5, 127.7, 129.4, 129.7, 129.8, 130.0, 131.9, 146.2, 168.4, 175.6.

ESI (MS) calculated for $C_{36}H_{38}N_{12}O_2$: 670.32 m/z; found: 1342.58 m/z ($[2M+H]^+$), 671.58 m/z ($[M+H]^+$), 336.33 m/z ($[(M+2H)/2]^{2+}$).

3,3'-((((5-nitro-1,3-phenylene)bis(1,2,4-oxadiazole-3,5-diyl))bis(pyridine-6,2-diyl))bis(1*H*-1,2,3-triazole-4,1-diyl))bis(*N*,*N*-dimethylpropan-1-amine) 21

White solid, isolated yield = 79%. ^{1}H-NMR (300MHz, $CDCl_3$), δ (ppm) = 1.25 (impurity), 2.14-2.24 (qui, 4H, *J* = 6.9 Hz), 2.27 (s, 12H), 2.32-2.41 (t, 4H, *J* = 6.5 Hz), 4.52-4.61 (t, 4H, *J* = 6.9 Hz), 7.28 ($CDCl_3$), 8.00-8.09 (t, 2H, *J* = 7.8 Hz), 8.24-8.31 (d, 2H, *J* = 7.6 Hz), 8.43-8.48 (m, 4H), 9.19 (s, 2H), 9.32 (s, 1H). ^{13}C-NMR (75MHz, $CDCl_3$), δ (ppm) = 28.1, 45.2, 48.5, 55.8, 76.5-77.4 ($CDCl_3$), 123.3, 123.4, 124.7, 129.3, 131.4, 138.1, 142.7, 147.0, 149.0, 151.6, 167.0, 175.2.

ESI (MS) calculated for $C_{34}H_{35}N_{15}O_4$: 717.30 m/z; found: 1435.25 m/z ($[2M+H]^+$), 718.58 m/z ($[M+H]^+$), 359.83 m/z ($[(M+2H)/2]^{2+}$).

3,3'-((((5-nitro-1,3-phenylene)bis(1,2,4-oxadiazole-3,5-diyl))bis(3,1-phenylene))bis(1*H*-1,2,3-triazole-4,1-diyl))bis(*N*,*N*-dimethylpropan-1-amine) 22

Off-white solid, isolated yield = 77%. ^{1}H-NMR (300MHz, $CDCl_3$), δ (ppm) = 1.12-2.10 (impurities), 2.17-2.30 (qui, 4H, *J* = 6.9 Hz), 2.33 (s, 12H), 2.42-2.50 (t, 4H, *J* = 6.9 Hz), 4.38 (bs, 3H), 4.52-4.61 (t, 4H, *J* = 6.9 Hz), 7.29 ($CDCl_3$), 7.63-7.72 (t, 2H, *J* = 7.8 Hz), 8.07 (s, 2H), 8.18-8.25 (d, 4H, *J* = 7.9 Hz), 8.65 (s, 2H), 9.17 (s, 2H), 9.29 (s, 1H). ^{13}C-NMR (75MHz, $CDCl_3$), δ (ppm) = 27.6, 44.8, 48.2, 55.5, 76.5-77.3 ($CDCl_3$), 120.6, 124.1, 124.4, 125.2, 127.5, 129.6, 129.8, 130.2, 131.3, 132.0, 146.1, 166.8, 176.3.

ESI (MS) calculated for $C_{36}H_{37}N_{13}O_4$: 715.31 m/z; found: 1432.33 m/z ($[2M+H]^+$), 716.58 m/z ($[M+H]^+$), 358.75 m/z ($[(M+2H)/2]^{2+}$).

Synthesis of heptacyclic oligo-heteroaryls 23 and 24

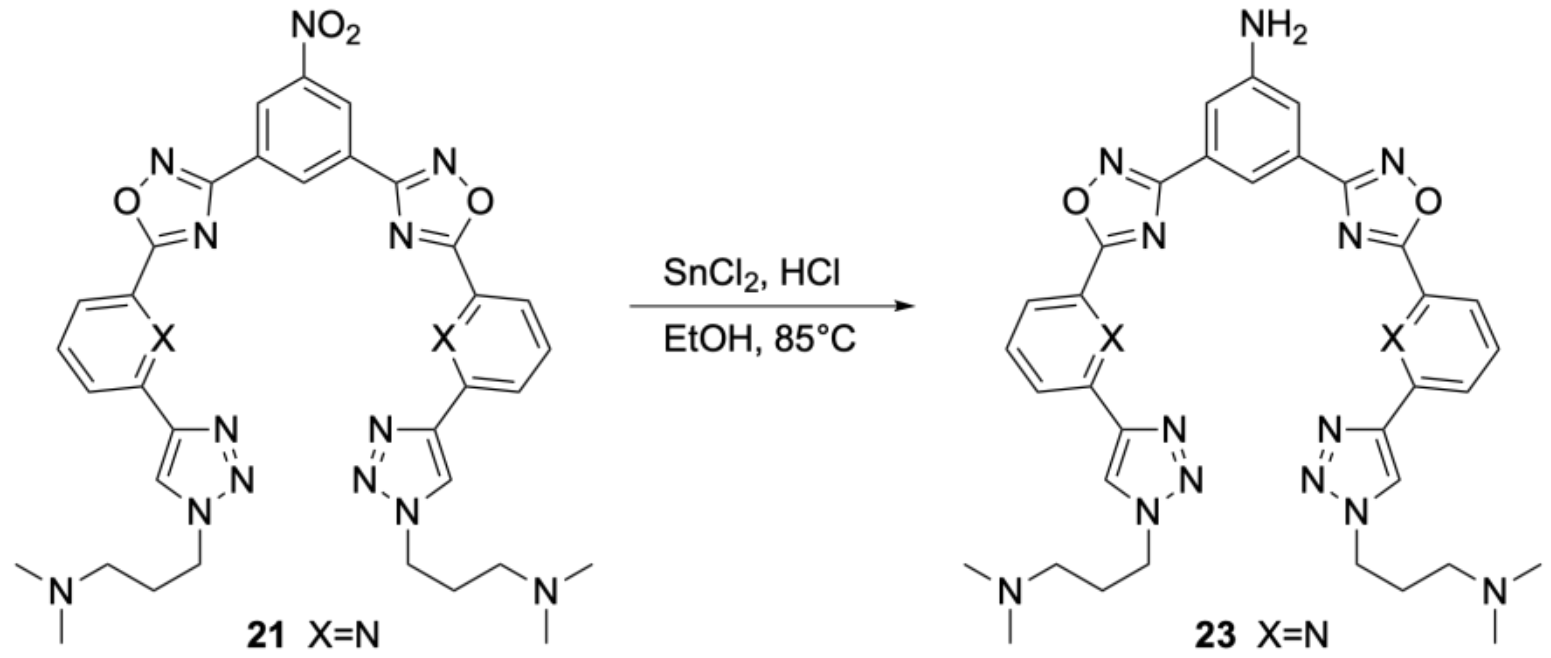

General Procedure

To a solution of the nitro derivative (**21** or **22**, 1.0 eq.) was dissolved in EtOH (5 mL), and 37% HCl (0.5 mL) was added. Subsequently, tin (II) dichloride (6.0 eq.) was added in one portion, and the suspension was stirred at 85°C for 6 hours. The reaction was monitored with analytical HPLC (Gradient H_2O + 0.1% TFA:MeCN). Upon completion, the solvent was removed at reduced pressure, and the residue was dissolved in 1M NaOH and extracted with $CHCl_3$ (3 x 20 mL). The combined organic phases were dried over Na_2SO_4, filtered, and concentrated *in vacuo*. The residue was dissolved in 1% HCl and purified with preparative HPLC (Gradient H_2O + 0.1% TFA:MeCN), and the collected fractions containing the product of interest were neutralized with a sat. aq. $NaHCO_3$ solution and extracted with $CHCl_3$ (3 x 50 mL). The combined organic phases were dried over Na_2SO_4, and the solvent was removed at reduced pressure.

3,3'-((((5-amino-1,3-phenylene)bis(1,2,4-oxadiazole-3,5-diyl))bis(pyridine-6,2-diyl))bis(1*H*-1,2,3-triazole-4,1-diyl))bis(*N*,*N*-dimethylpropan-1-amine) 23

White solid, isolated yield = 70%. ^{1}H-NMR (300MHz, $CDCl_3$), δ (ppm) = 1.26 (impurity), 2.12-2.23 (qui, 4H, *J* = 6.9 Hz), 2.26 (s, 12H), 2.31-2.40 (t, 4H, *J* = 6.7 Hz), 4.15 (bs, 2H), 4.51-4.59 (t, 4H, *J* = 6.9 Hz), 7.28 ($CDCl_3$), 7.68 (s, 2H), 7.97-8.06 (t, 2H, *J* = 7.8 Hz), 8.22-8.28 (d, 2H, *J* = 7.7 Hz), 8.39-8.46 (m, 3H). ^{13}C-NMR (75MHz, $CDCl_3$), δ (ppm) = 28.2, 29.7, 45.4, 48.5, 55.9, 76.6-77.5 ($CDCl_3$), 116.3, 116.9, 123.1, 123.2, 123.5, 128.4, 138.2, 143.3, 147.3, 147.5, 151.5, 168.8, 174.4.

ESI (MS) calculated for $C_{34}H_{37}N_{15}O_2$: 687.33 m/z; found: 1375.58 m/z ($[2M+H]^+$), 688.67 m/z ($[M+H]^+$), 344.83 m/z ($[(M+2H)/2]^{2+}$), 230.17 m/z ($[(M+3H)/3]^{3+}$).

3,3'-((((5-amino-1,3-phenylene)bis(1,2,4-oxadiazole-3,5-diyl))bis(3,1-phenylene))bis(1*H*-1,2,3-triazole-4,1-diyl))bis(*N*,*N*-dimethylpropan-1-amine) 24

White solid, isolated yield = 77%. ^{1}H-NMR (300MHz, $CDCl_3$), δ (ppm) = 0.09 (TMS), 0.85-1.50 (impurities), 2.10-2.25 (qui, 4H, *J* = 6.6 Hz), 2.28 (s, 12H), 2.32-2.40 (t, 4H, *J* = 6.9 Hz), 4.17 (bs, 2H), 4.51-4.60 (t, 4H, *J* = 6.9 Hz), 7.29 ($CDCl_3$), 7.61-7.69 (m, 4H), 8.02 (s, 2H), 8.16-8.23 (m, 4H),

8.41 (s, 1H), 8.64 (s, 2H). ^{13}C-NMR (75MHz, $CDCl_3$), δ (ppm) = 28.0, 29.6, 45.2, 48.2, 55.7, 76.5-77.3 ($CDCl_3$), 116.0, 116.8, 120.6, 124.7, 125.1, 127.4, 128.5, 129.6, 129.7, 131.8, 146.2, 147.3, 168.5, 175.4.

ESI (MS) calculated for $C_{36}H_{39}N_{13}O_2$: 685.33 m/z; found: 1372.58 m/z ($[2M+H]^+$), 686.58 m/z ($[M+H]^+$), 343.83 m/z ($[(M+2H)/2]^{2+}$), 229.50 m/z ($[(M+3H)/3]^{3+}$).

Biophysical assays and *in silico* analysis

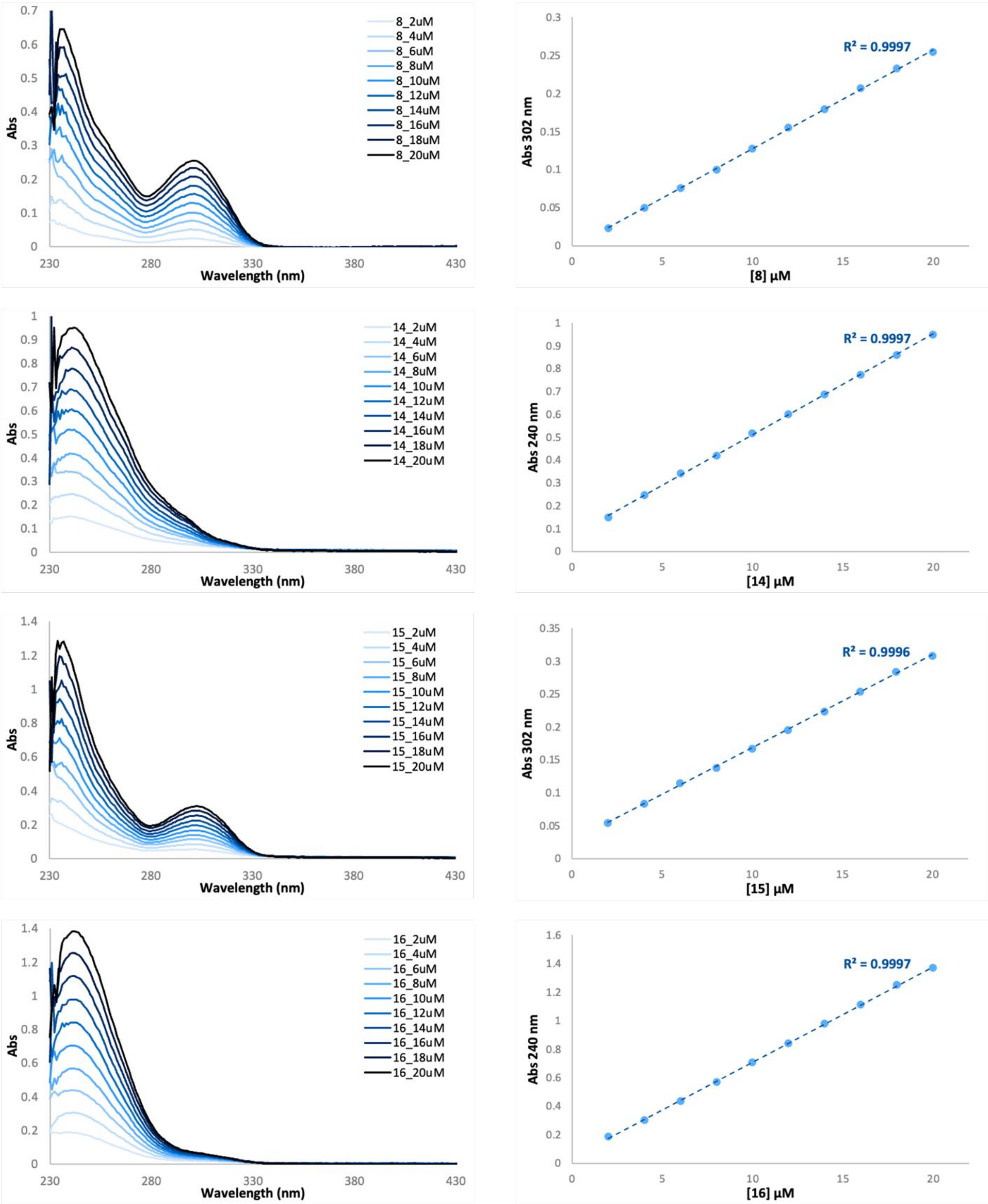

Figure S1. *Left: overlapped UV-Vis absorption spectra of **8**, **14**, **15**, and **16** recorded at increasing concentrations (2-20 µM) under aqueous conditions. Right: linear correlation between absorbance (Abs) and ligand concentration, showing preservation of Beer–Lambert behaviour across the investigated concentration range.*

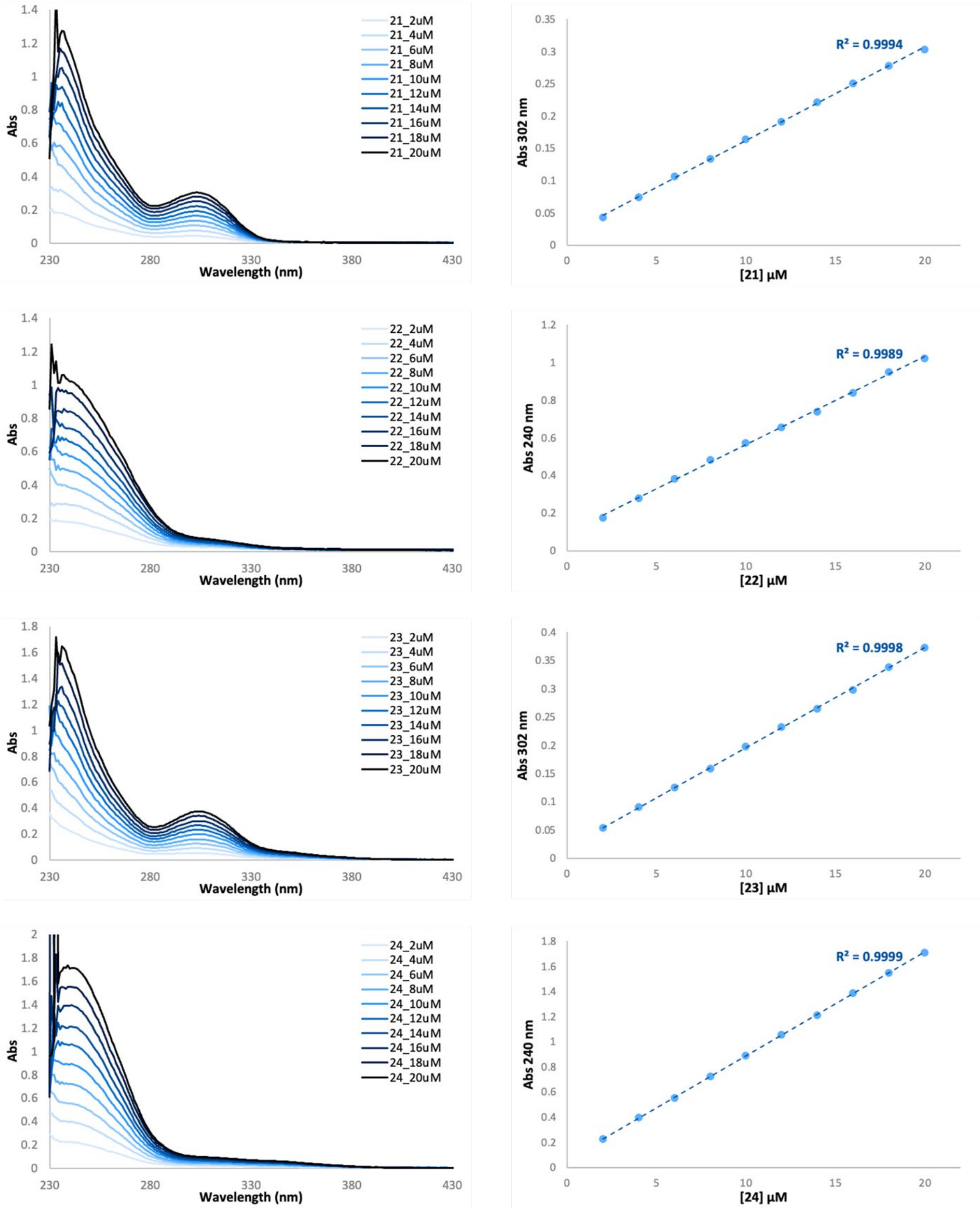

Figure S2. *Left: overlapped UV-Vis absorption spectra of **21**, **22**, **23**, and **24** recorded at increasing concentrations (2-20 µM) under aqueous conditions. Right: linear correlation between absorbance (Abs) and ligand concentration, showing preservation of Beer–Lambert behaviour across the investigated concentration range.*

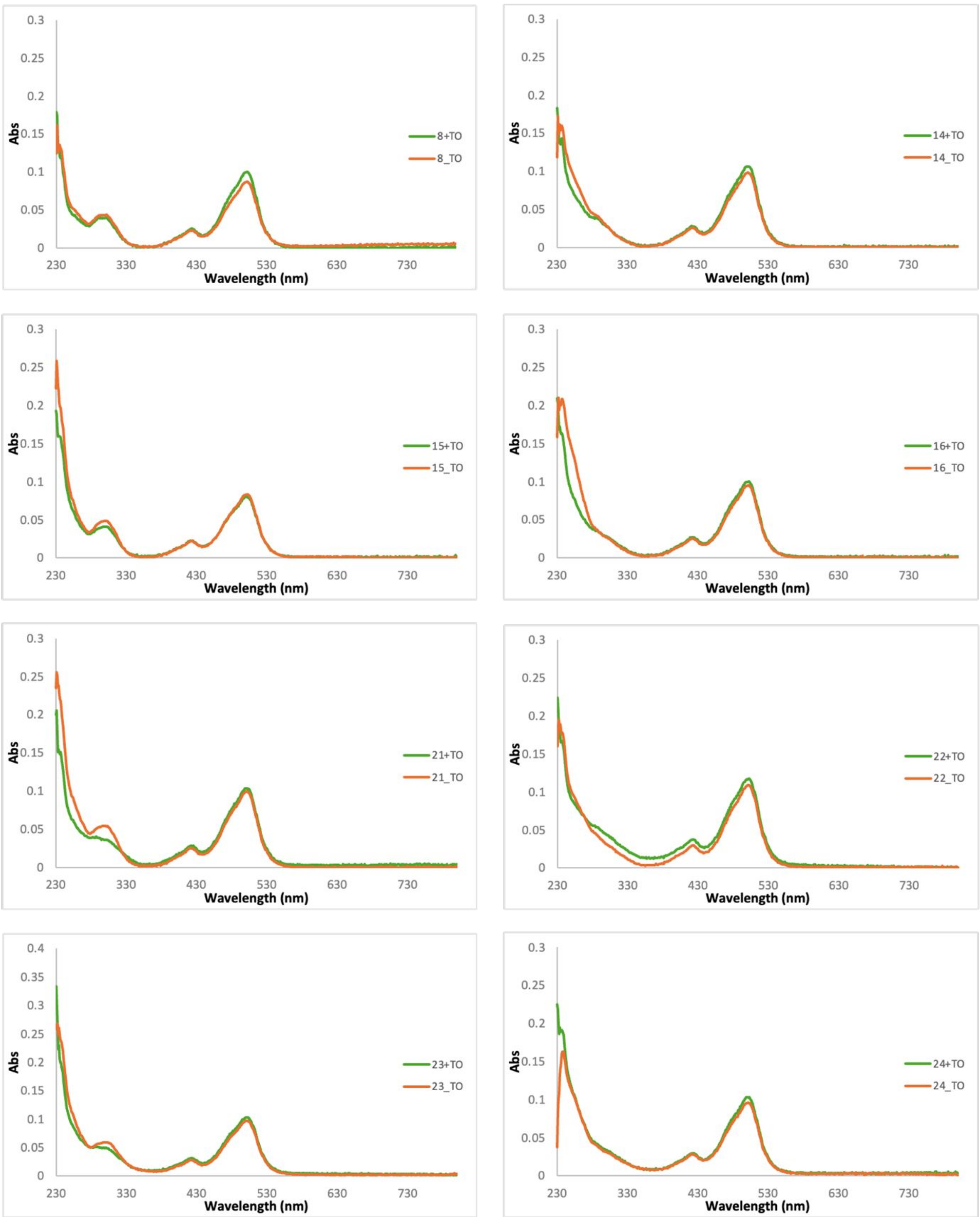

Figure S3. *UV-Vis absorption spectra of TO and heptacyclic ligands (**8**, **14**, **15**, **16**, **21**, **22**, **23**, and **24**) at 2 µM concentration, in 100 mM KCl and 10 mM lithium cacodylate buffer (pH=7.2). The green traces correspond to the mathematical sum of the individual spectra of TO and each ligand (2 µM + 2 µM), while the orange traces represent the experimentally recorded spectra of the corresponding TO/ligand mixtures under the same conditions.*

Table S2. *Melting temperature determined by FRET-melting experiments for FAM/TAMRA-labelled myc and LTR-IV G4s in the presence of 4 equivalents (1 µM) of the indicated ligands and after addition of 2.5 µM of competitor nucleic acid structures (ds26, ds12, hairpin, and ss-scr). Melting temperature values are reported as mean ± SD from three independent experiments.* ***NDIpip*** *was used as a reference end-stacking ligand.*

	Melting temperature (°C)								
	**8**	**14**	**15**	**16**	**21**	**22**	**23**	**24**	**NDIpip**
myc	75 ± 1	71 ± 2	66.5 ± 0.9	79.2 ± 0.5	68.2 ± 0.9	81.2 ± 0.7	73.4 ± 0.5	81.0 ± 0.6	88.0 ± 0.2
myc+ds26	75 ± 2	69 ± 3	68 ± 2	76 ± 2	67 ± 2	75 ± 1	76 ± 2	85 ± 1	81 ± 1
myc+hairpin	74 ± 2	71 ± 3	70 ± 1	76 ± 2	68 ± 2	77 ± 1	78 ± 1	87 ± 1	81 ±2
myc+ds12	76 ± 2	70 ± 3	69 ± 2	76 ± 2	70 ± 2	74 ± 2	74.6 ± 0.9	83 ± 1	82.7 ± 0.7
myc+scr	70 ± 4	68 ± 3	70 ± 2	73 ± 2	66 ± 2	80 ± 1	73 ± 3	88 ± 3	77 ± 6
LTR-IV	75 ± 3	70 ± 1	68 ±1	79.5 ± 0.5	72.5 ± 0.7	78.2 ± 0.4	80.9 ± 0.7	85.5 ± 0.4	87.7 ± 0.4
LTR-IV+ds26	75 ± 4	71 ± 3	67 ± 2	76 ± 1	71 ± 1	80 ± 1	78 ± 5	92.5 ± 0.8	79 ± 1
LTR-IV+hairpin	74 ± 5	70 ± 3	67 ± 2	77 ± 1	70 ± 2	74 ± 1	78 ± 1	86 ±.3	80 ± 2
LTR-IV+ds12	76 ± 4	75 ± 2	68 ± 2	80 ± 1	73 ± 2	77 ± 1	84 ± 2	90.9 ± 0.6	83 ± 1
LTR-IV+scr	70 ± 9	69 ± 4	68 ± 2	73 ± 2	71 ± 2	74 ± 1	75 ± 2	94 ± 2	69 ± 8

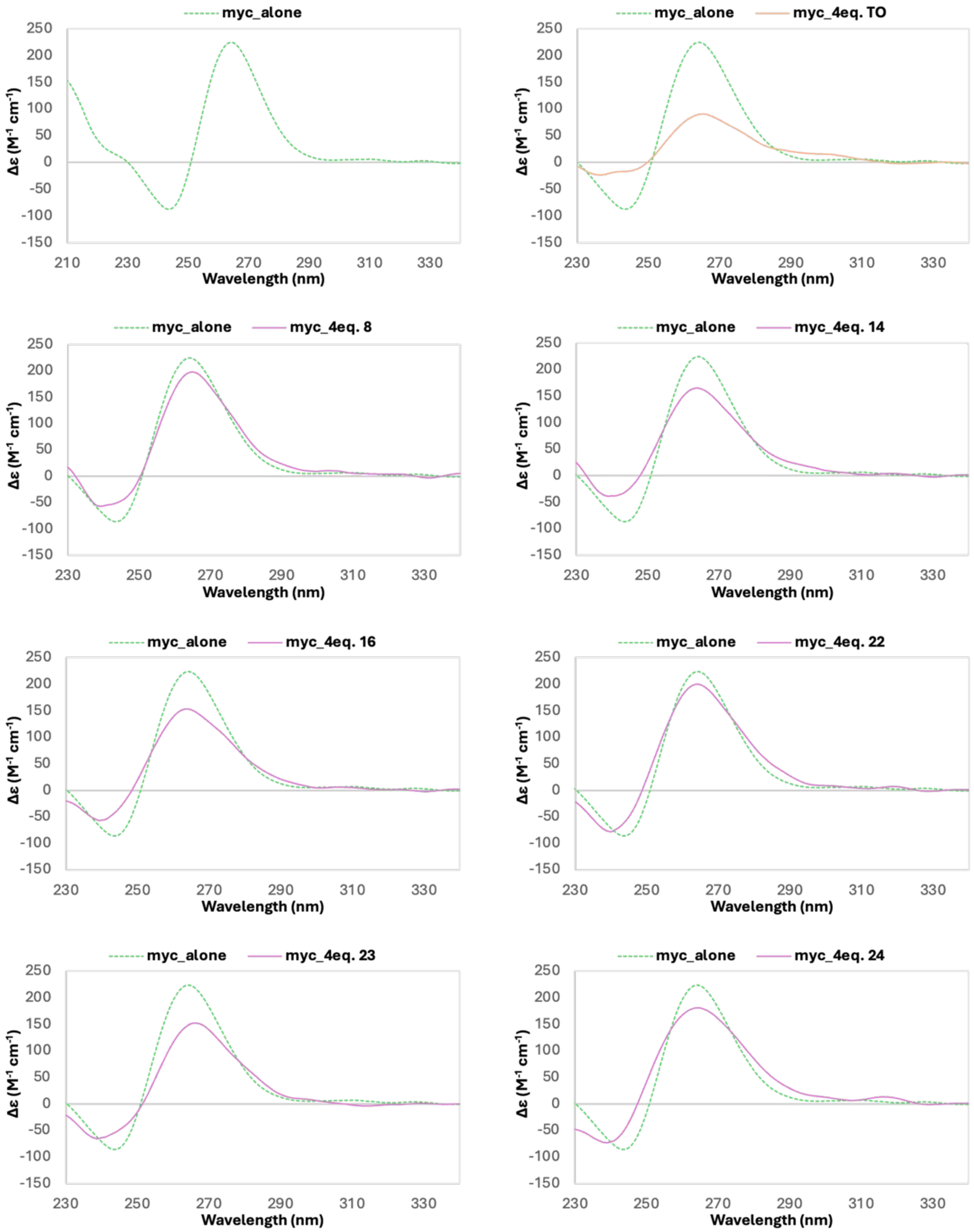

Figure S4. *CD spectra of myc G4 sequences alone and in the presence of 4 equivalents of TO, or 8, 14, 16, 22-24 heptacyclic ligands. Conditions: 2 µM myc, 8 µM compounds, 1 mM KCl, 99 mM LiCl in lithium cacodylate buffer 10 mM (pH 7.2).*

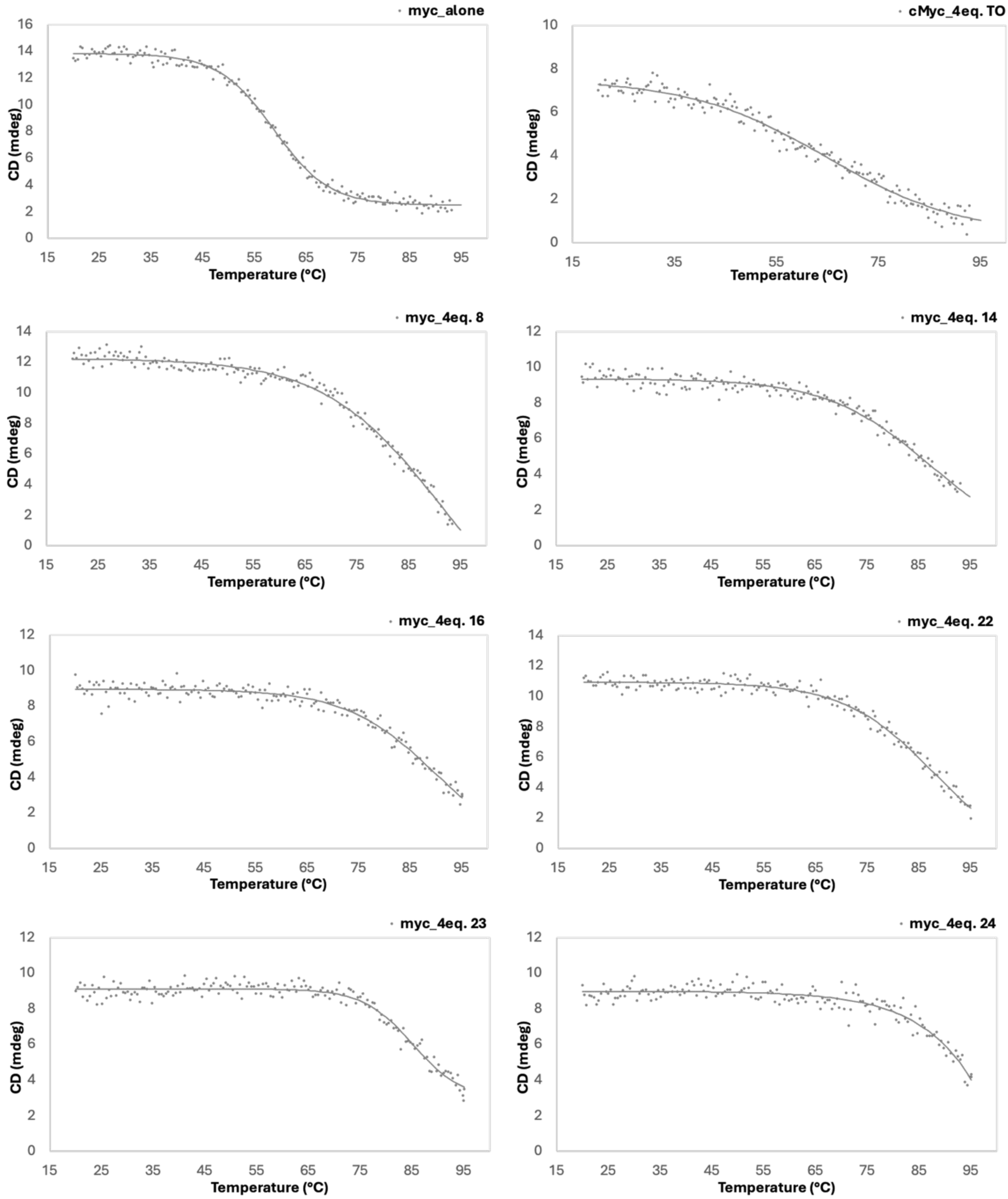

Figure S5. *CD-melting curves of myc recorded at 264 nm in the absence (myc_alone) or in the presence of ligands (TO,* ***8****,* ***14****,* ***16****,* ***22****,* ***23****, and* ***24****). Spectra were acquired while heating from 20 °C to 95 °C at a rate of 1 °C/min. Measurements were performed using a 2 μM oligonucleotide concentration, 8 μM compounds (TO or heptacyclic ligands), with 1 mM KCl and 99 mM LiCl, in 10 mM lithium cacodylate buffer (pH 7.2).*

Table S3. *Melting temperatures (T_m) of the myc G-quadruplex alone and in the presence of various ligands. The table reports the measured T_m values (in °C) with standard deviations (Dev. Std.) for myc alone, myc with TO (thiazole orange), and myc with different compounds (indicated by number) at 4 equivalents. ΔT_m values represent the stabilization effect relative to the unbound myc quadruplex. Combinations of ligands and TO at 4 equivalents each are also reported to assess possible additive or cooperative stabilization effects.*

	T_m [°C]	ΔT_m [°C]
myc alone	58.7 ± 0.2	
myc + 4eq. TO	64.9 ± 0.9	6 ± 1
myc + 4eq. **8**	91 ± 2	33 ± 2
myc + 4eq. **14**	87 ± 2	28 ± 2
myc + 4eq. **16**	90 ± 2	31 ± 2
myc + 4eq. **22**	88 ± 2	29 ± 2
myc + 4eq. **23**	85.2 ± 0.7	26.6 ± 0.9
myc + 4eq. **24**	>95.0	>36.3
myc + 4eq. TO + 4 eq. **8**	71 ± 1	12 ± 1
myc + 4eq. TO + 4 eq. **14**	81 ± 4	22 ± 4
myc + 4eq. TO + 4 eq. **16**	76 ± 2	17 ± 2
myc + 4eq. TO + 4 eq. **22**	89 ± 3	31 ± 3
myc + 4eq. TO + 4 eq. **23**	80 ± 2	21 ± 2
myc + 4eq. TO + 4 eq. **24**	78.3 ± 0.7	19.6 ± 0.9

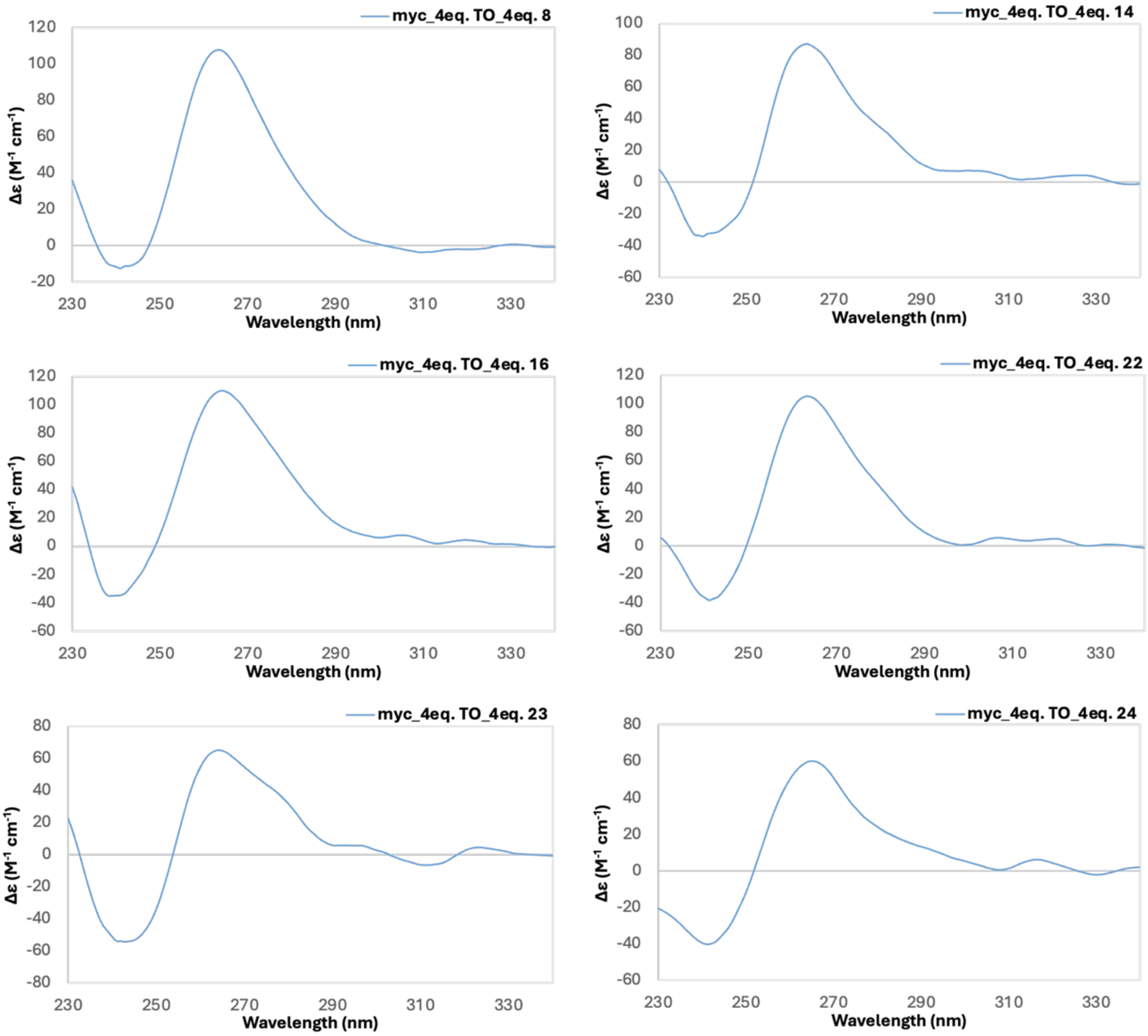

Figure S6. *CD spectra of myc G4 in the presence of 4 equivalents of TO and heptacyclic ligands. Conditions: 2 μM myc, 8 μM TO, 8 μM ligands, 1 mM KCl, 99 mM LiCl in 10 mM lithium cacodylate buffer (pH 7.2).*

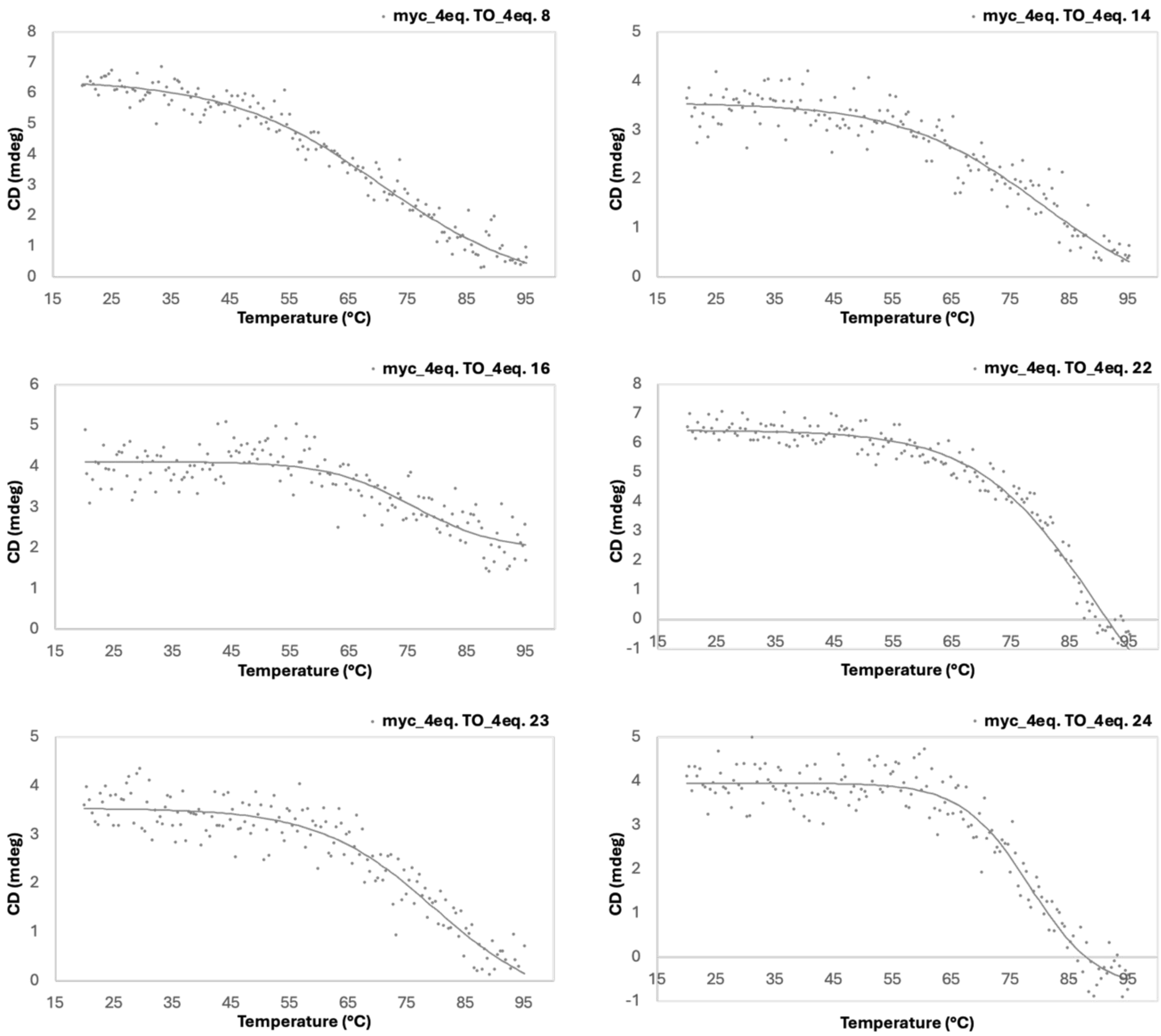

Figure S7. *CD-melting curves of myc recorded at 264 nm in the presence of 4 equivalents of TO and 4 equivalents of heptacyclic ligands (**8**, **14**, **16**, **22**, **23**, and **24**). Spectra were acquired while heating from 20 °C to 95 °C at a rate of 1 °C/min. Measurements were performed using a 2 µM oligonucleotide concentration, 8 µM TO, 8 µM heptacyclic ligands, with 1 mM KCl and 99 mM LiCl, in 10 mM lithium cacodylate buffer (pH 7.2).*

A)

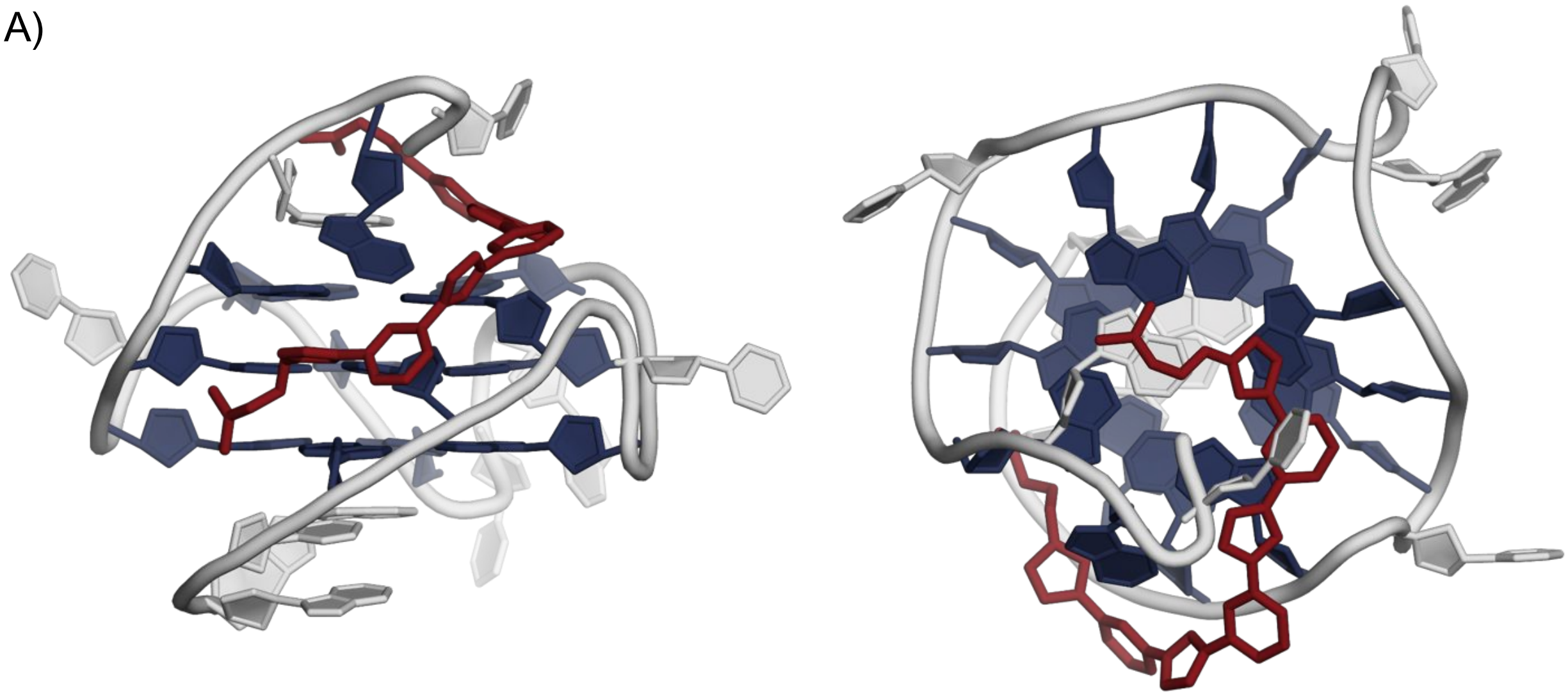

B)

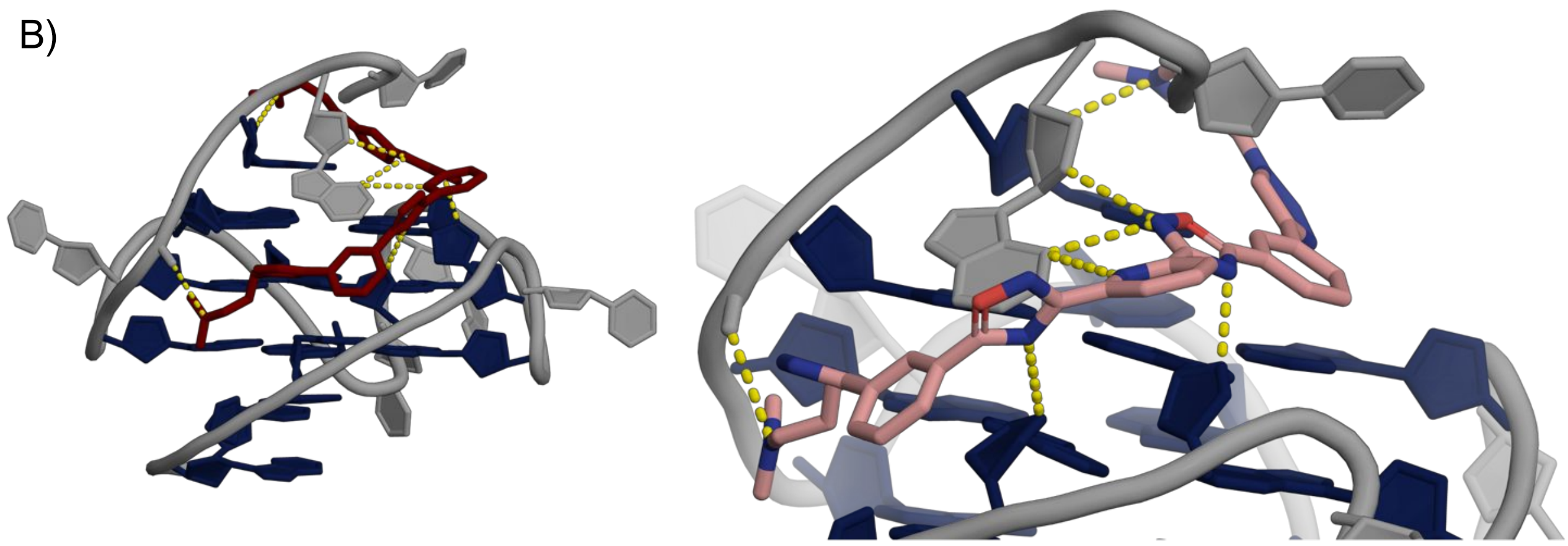

Figure *S8. Docking pose of compound **14** with the Myc G4 (PDB: 1XAV). A) The images show (left) a side view and (right) a top view. B) The images show seven hydrogen-bonding interactions established between the heteroatom-rich heptacyclic core and polar groups exposed within the groove. Ligand **14** is represented in red, the guanine tetrads in blue, and the DNA backbone and other nucleobases in grey.*

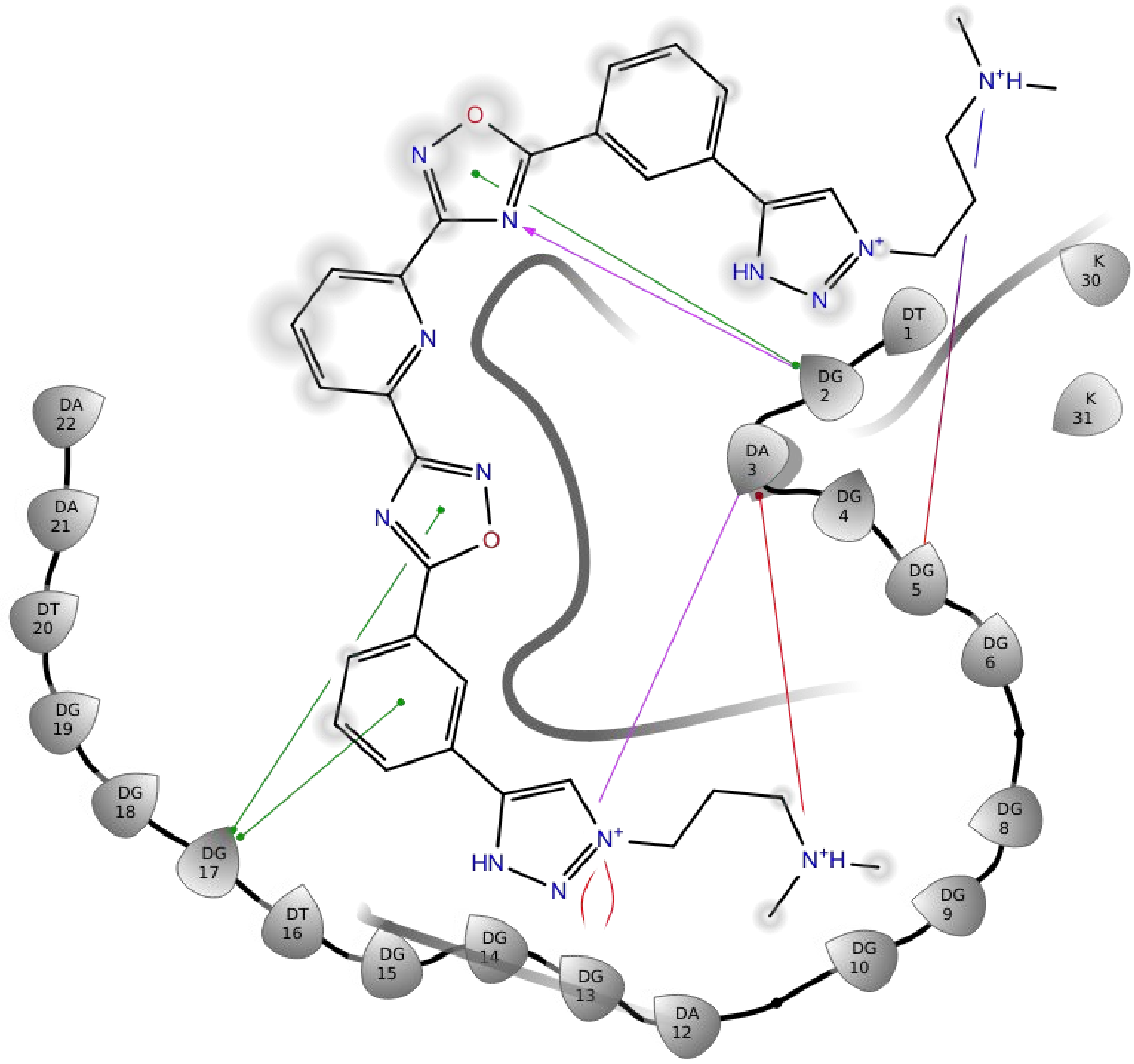

Figure ***S9***. *Two-dimensional interaction map of ligand 14 docked into the myc G-quadruplex (PDB: 1XAV). The diagram highlights the interactions identified in the selected docking pose, including hydrogen-bonding contacts (pink arrows) involving the heteroaryl scaffold, contacts mediated by the protonated dimethylamino side chains (red arrows), and interactions with nucleobases lining the G-quadruplex groove and terminal tetrad regions (H-π interaction, green arrows).*

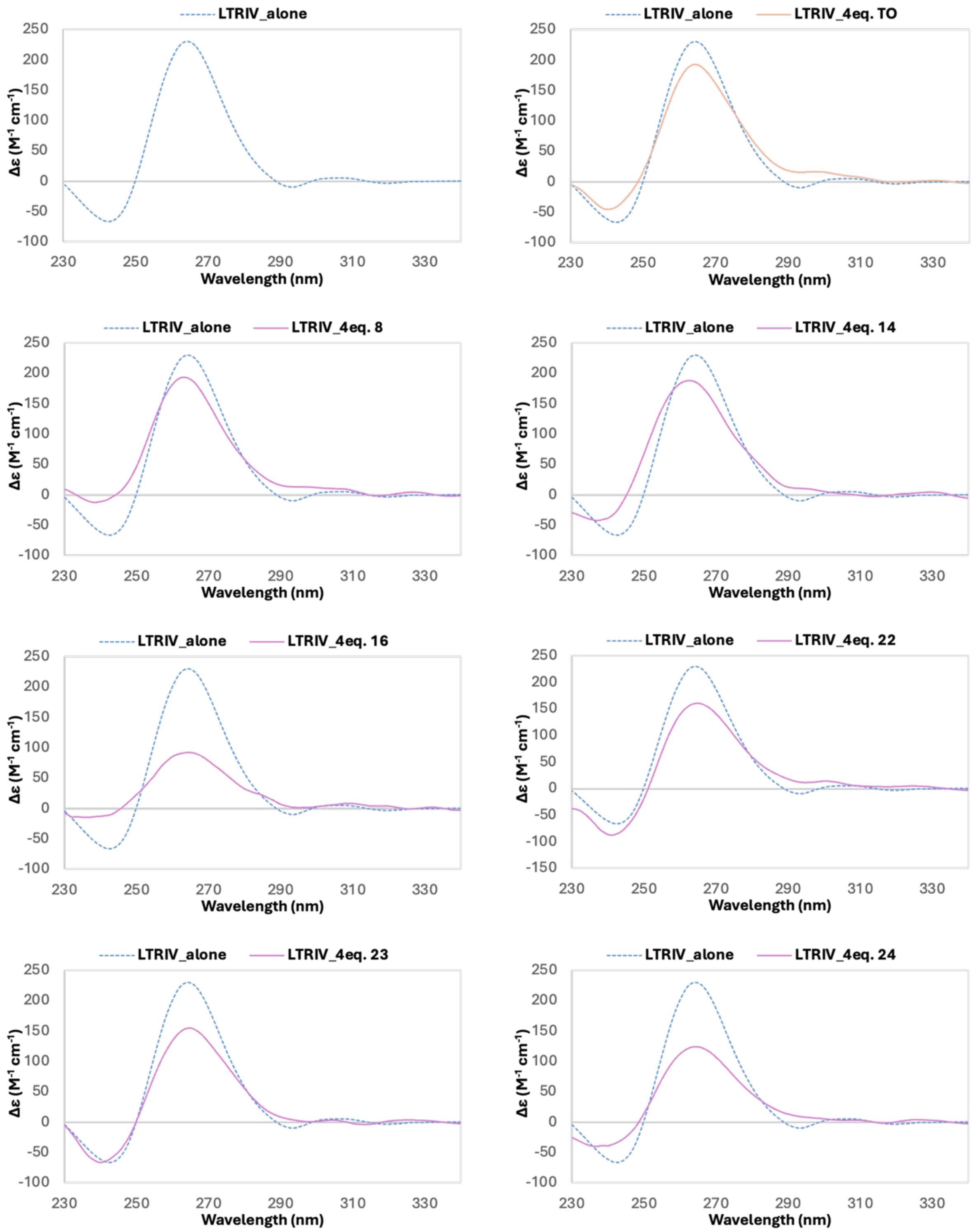

Figure S10. *CD spectra of LTRIV G4 sequences alone and in the presence of 4 equivalents of TO or heptacyclic ligands. Conditions: 2 µM LTRIV, 8 µM compounds, 100 mM KCl, in 10 mM lithium cacodylate buffer (pH 7.2).*

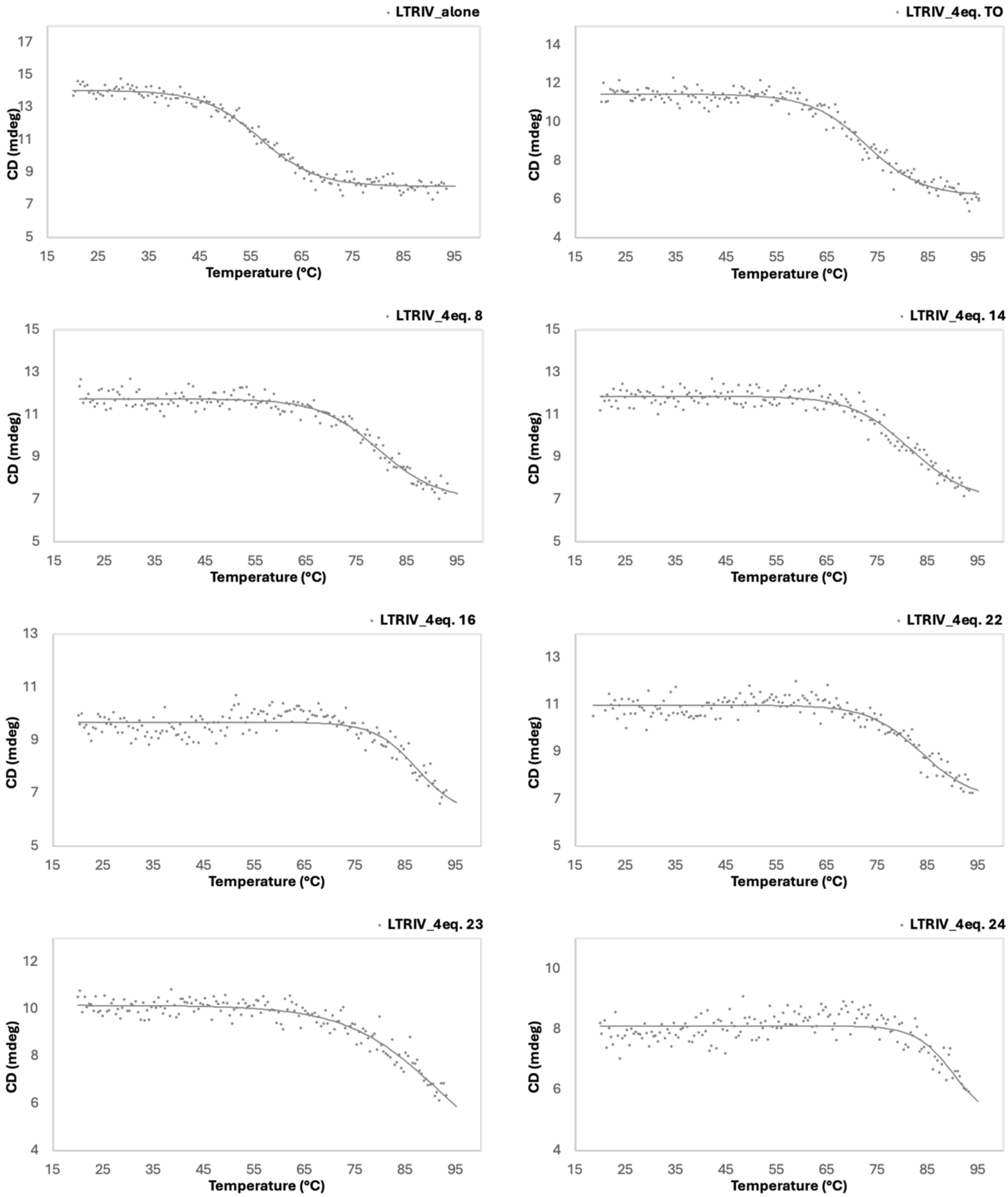

Figure S11. *CD-melting curves of LTRIV recorded at 264 nm in the absence (LTRIV_alone) or in the presence of 4 equivalents of TO or 4 equivalents of heptacyclic ligands (**8**, **14**, **16**, **22**, **23**, and **24**). Spectra were acquired while heating from 20 °C to 95 °C at a rate of 1 °C/min. Measurements were performed using a 2 µM oligonucleotide concentration, 8 µM TO, 8 µM heptacyclic ligands, with 100 mM KCl in 10 mM lithium cacodylate buffer (pH 7.2).*

Table S2. *Melting temperatures (T_m) of the LTR IV G-quadruplex in the absence and presence of different ligands. The table shows T_m values (in °C) with associated standard deviations (Dev. Std.) for LTR IV alone, with TO (thiazole orange), and with selected compounds (numbered **8**, **14**, **16**, **22**, **23**, and **24**) at 4 equivalents. ΔT_m values indicate the thermal stabilization relative to the unbound LTR IV quadruplex. Combinations of ligands with TO at 4 equivalents each are also reported, all showing complete stabilization beyond the detection limit (Tm > 95°C).*

	T_m [°C]	ΔT_m [°C]
LTR-IV alone	56.7 ± 0.3	
LTR-IV + 4eq. TO	72.7 ± 0.4	15.9 ± 0.7
LTR-IV + 4eq. **8**	79.4 ± 0.7	23 ± 1
LTR-IV + 4eq. **14**	81.4 ± 0.8	25 ± 1
LTR-IV + 4eq. **16**	87 ± 1	31 ± 1
LTR-IV + 4eq. **22**	84 ± 1	27 ± 1
LTR-IV + 4eq. **23**	92 ± 4	35 ± 4
LTR-IV + 4eq. **24**	90 ± 2	33 ± 2
LTR-IV + 4eq. TO + 4 eq. **8**	>95	>38.3
LTR-IV + 4eq. TO + 4 eq. **14**	>95	>38.3
LTR-IV + 4eq. TO + 4 eq. **16**	>95	>38.3
LTR-IV + 4eq. TO + 4 eq. **22**	>95	>38.3
LTR-IV + 4eq. TO + 4 eq. **23**	>95	>38.3
LTR-IV + 4eq. TO + 4 eq. **24**	>95	>38.3

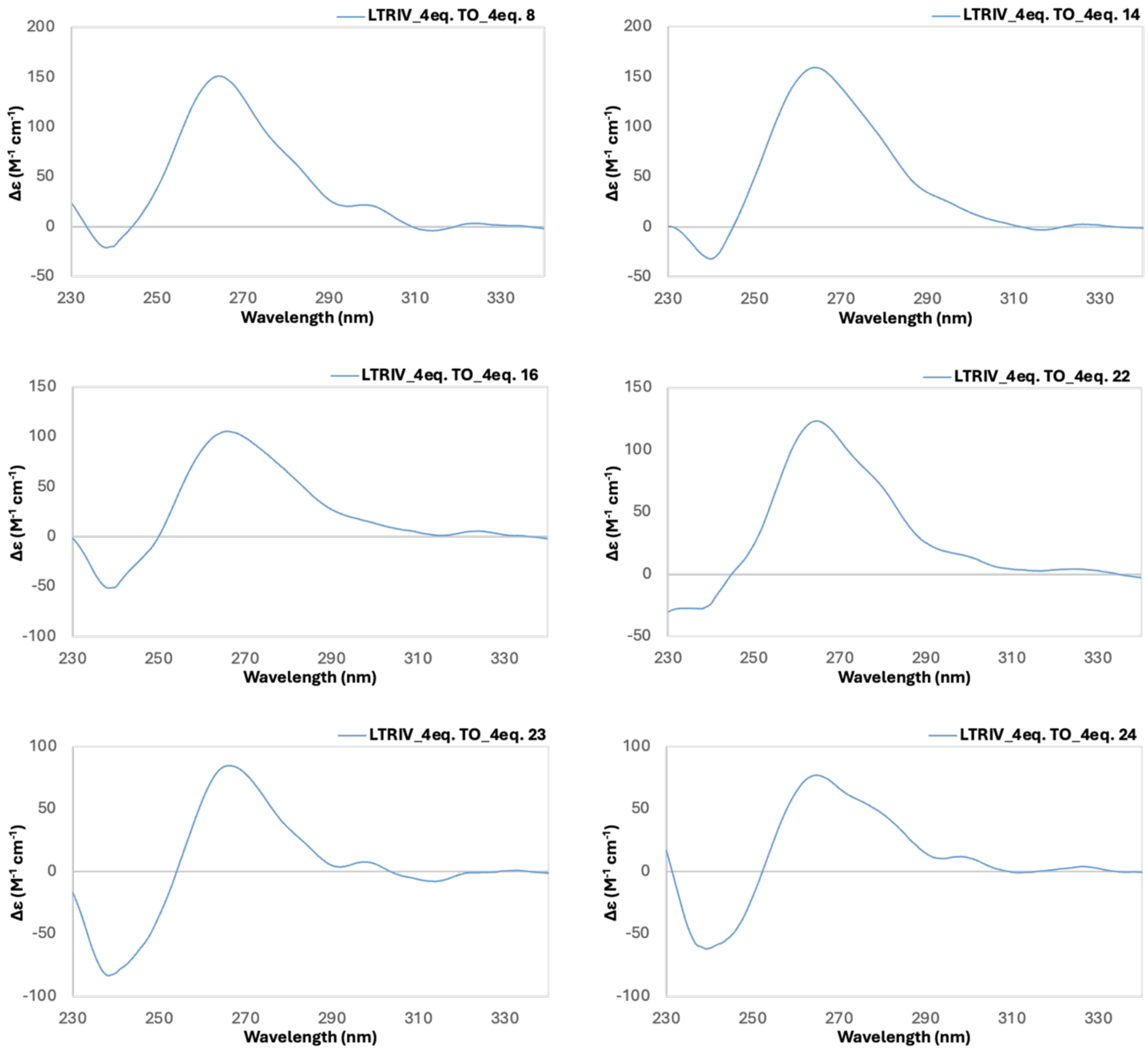

Figure S12. *CD spectra of LTRIV G4 in the presence of 4 equivalents of TO and heptacyclic ligands. Conditions: DNA 2 µM LTRIV, 8 µM TO, 8 µM compounds, 100 mM KCl, in 10 mM lithium cacodylate buffer (pH 7.2).*

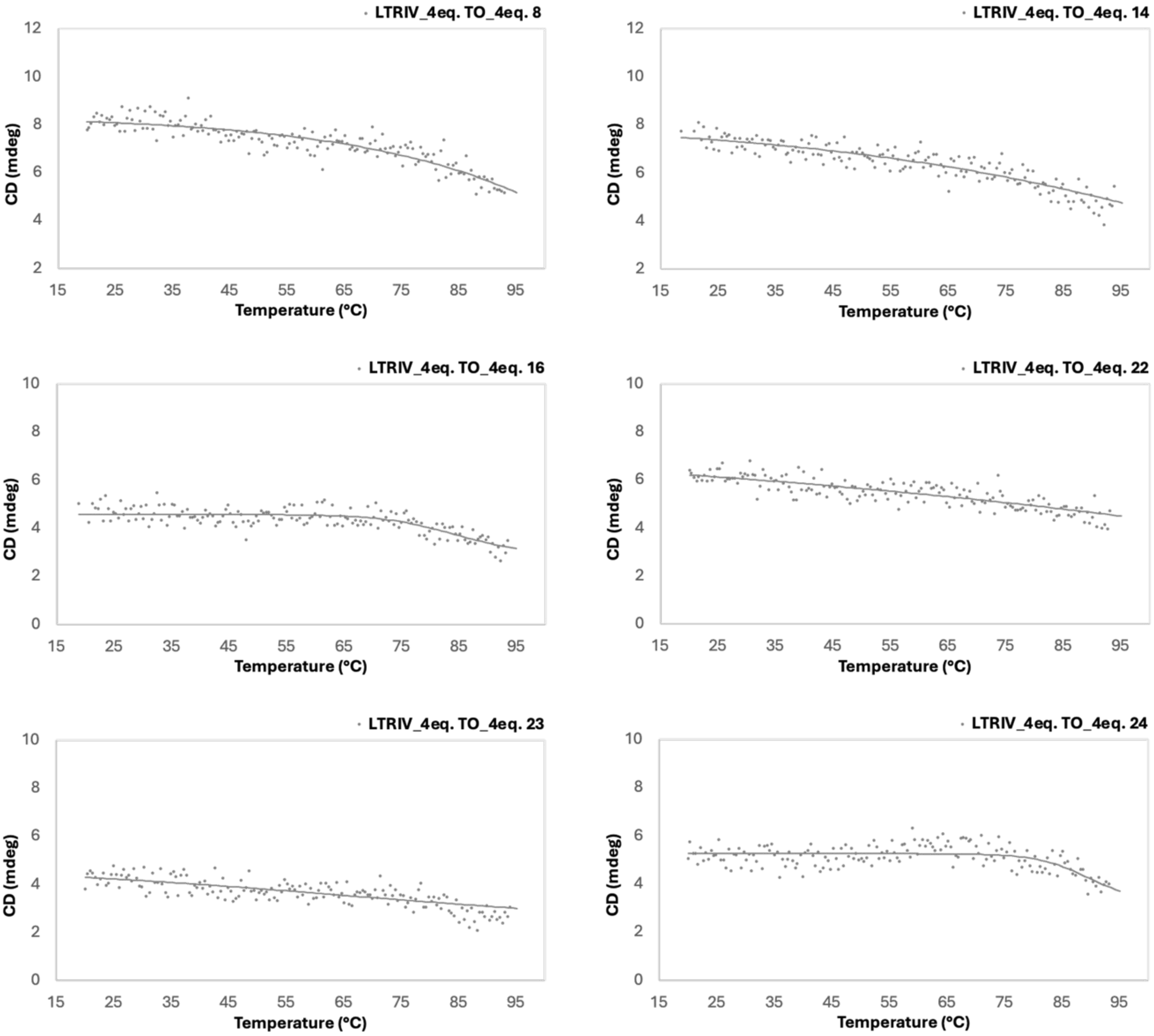

Figure S13. *CD-melting curves of LTRIV recorded at 264 nm in the presence of 4 equivalents of TO and 4 equivalents of heptacyclic ligands (**8**, **14**, **16**, **22**, **23**, and **24**). Spectra were acquired while heating from 20 °C to 95 °C at a rate of 1 °C/min. Measurements were performed using a 2 µM oligonucleotide concentration, 8 µM TO, 8 µM heptacyclic ligands, with 100 mM KCl in 10 mM lithium cacodylate buffer (pH 7.2).*

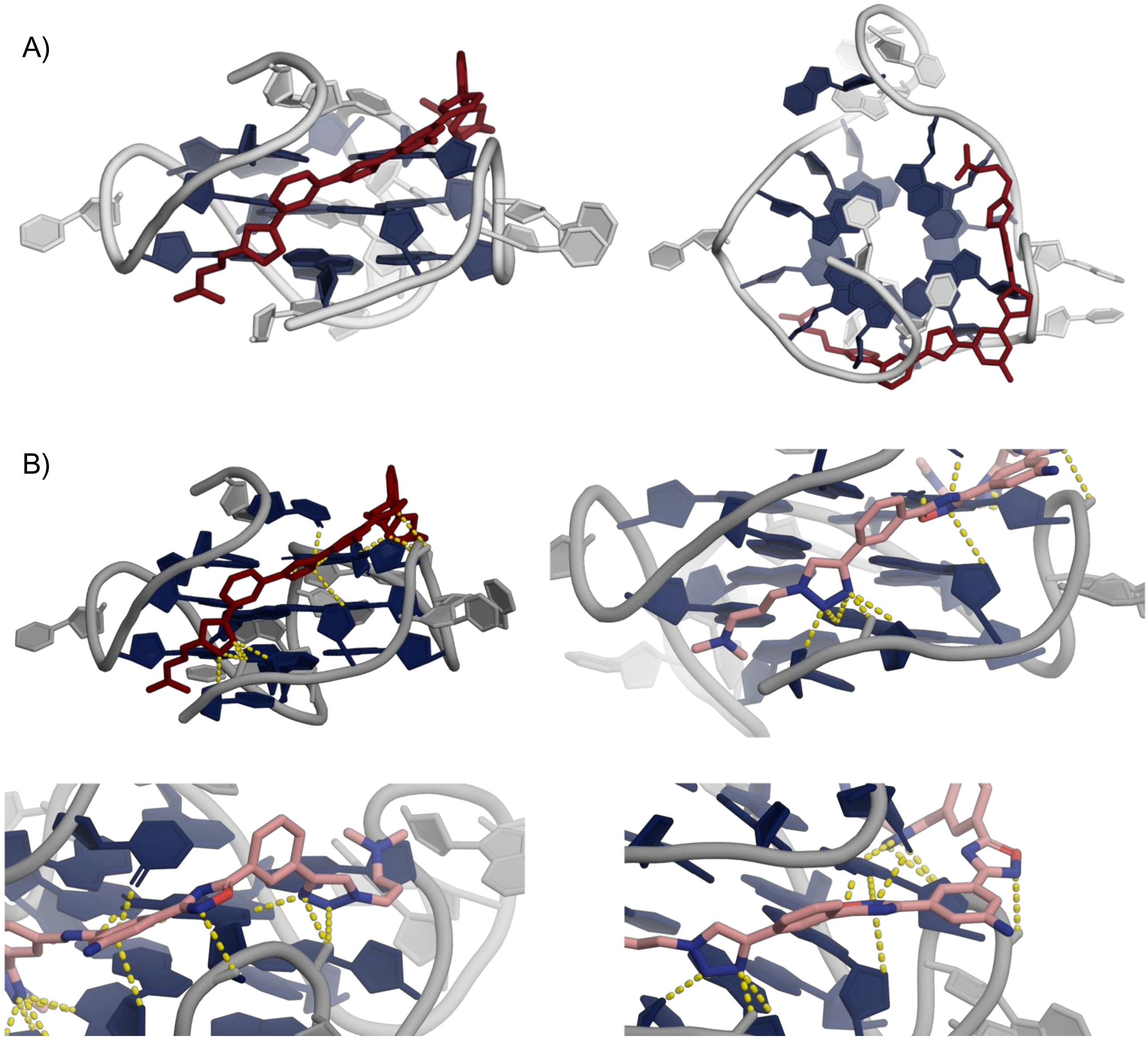

Figure S14. *Docking pose of compound **24** with the LTRIV G4 (PDB: 2N4Y). A) The images show (left) a side view and (right) a top view. B) The images show that ligand **24** is deeply accommodated within the groove region, where it establishes an extended network of hydrogen-bonding interactions involving both the heteroaryl scaffold and the terminal amino substituent. Ligand **24** is represented in red, the guanine tetrads in blue, and the DNA backbone and other nucleobases in grey.*

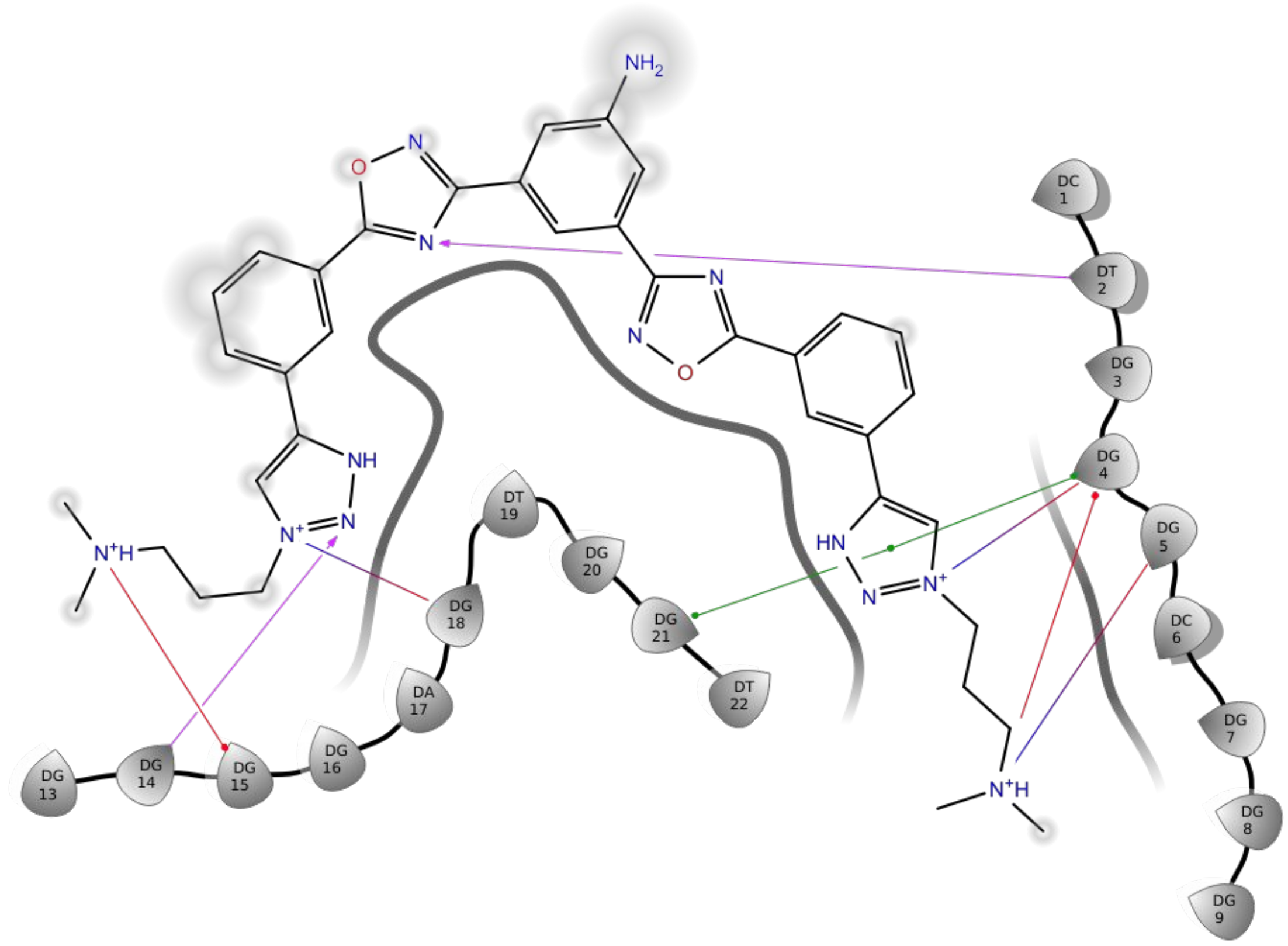

Figure *S15. Two-dimensional interaction map of ligand **24** docked into the myc G-quadruplex (PDB: 1XAV). The diagram highlights the interactions identified in the selected docking pose, including hydrogen-bonding contacts (pink arrows) involving the heteroaryl scaffold, contacts mediated by the protonated dimethylamino side chains (red arrows), and interactions with nucleobases lining the G-quadruplex groove and terminal tetrad regions (H-π interaction, green arrows).*

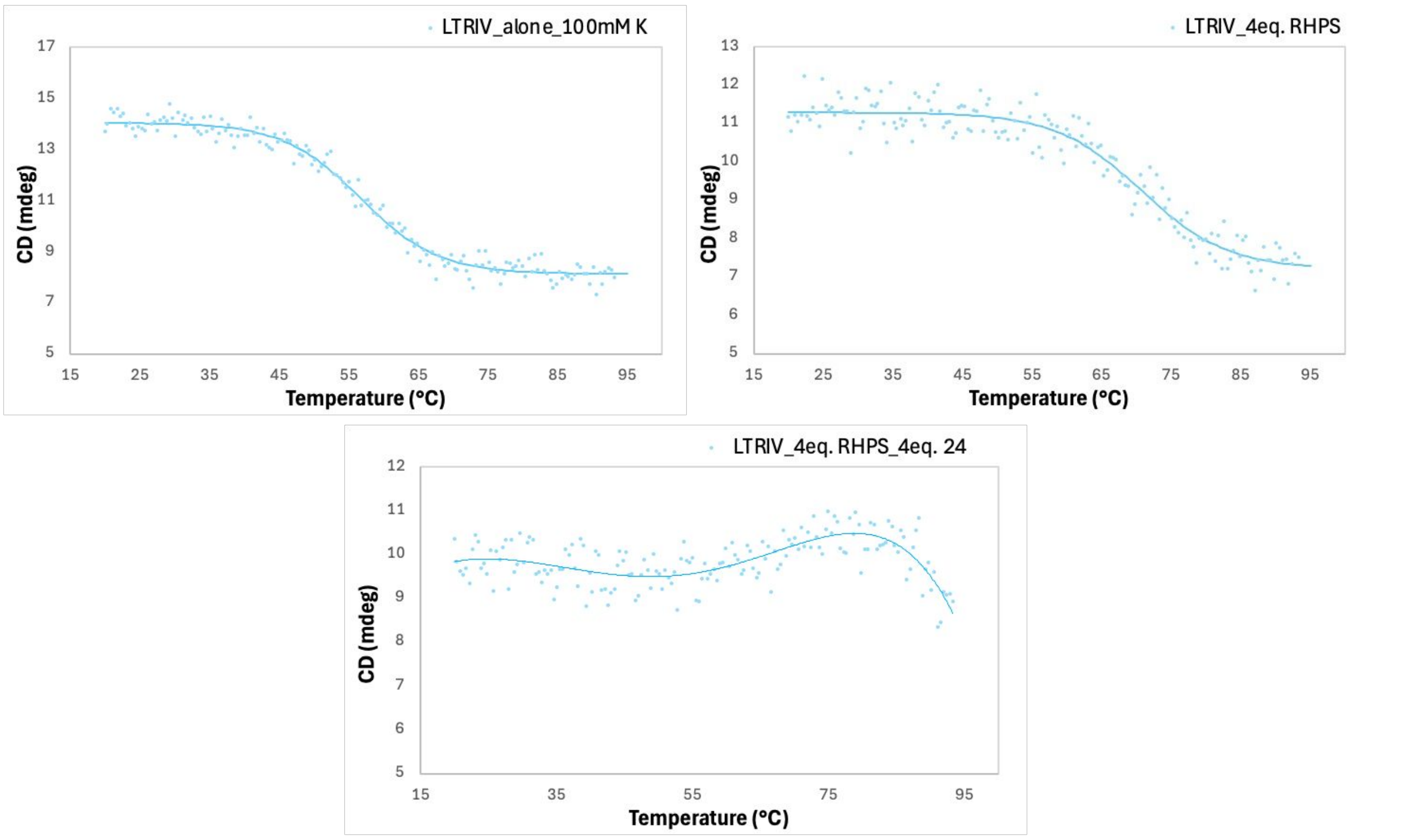

Figure S16. *CD-melting curves of LTRIV recorded at 264 nm alone, in the presence of 4 equivalents of RHSP4 and with 4 equivalents of heptacyclic ligand **24**. Spectra were acquired while heating from 20 °C to 95 °C at a rate of 1 °C/min. Measurements were performed using a 2 µM oligonucleotide concentration, 8 µM RHSP4, 8 µM **24**, with 100 mM KCl in 10 mM lithium cacodylate buffer (pH 7.2).*

Table S5. *Apparent association constants ($K_{1:1}$, M^{-1}) for the interaction of the heptacyclic ligands with LTR-IV G4, derived from UV-Vis titration data by HyperSpec analysis assuming either a 1:1 or a 1:2 ligand-G4 binding stoichiometry.*

	Log $K_{1:1}$ (M^{-1})	$K_{1:1}$ (M^{-1})
**8**	5.5 ± 0.2	3.35×10^5
**14**	5.37 ± 0.08	2.36×10^5
**16**	**6.0 ± 0.3**	**1.07×10^6**
**22**	5.09 ± 0.04	1.24×10^5
**23**	5.6 ± 0.3	3.80×10^5
**24**	**5.94 ± 0.04**	**8.76×10^5**

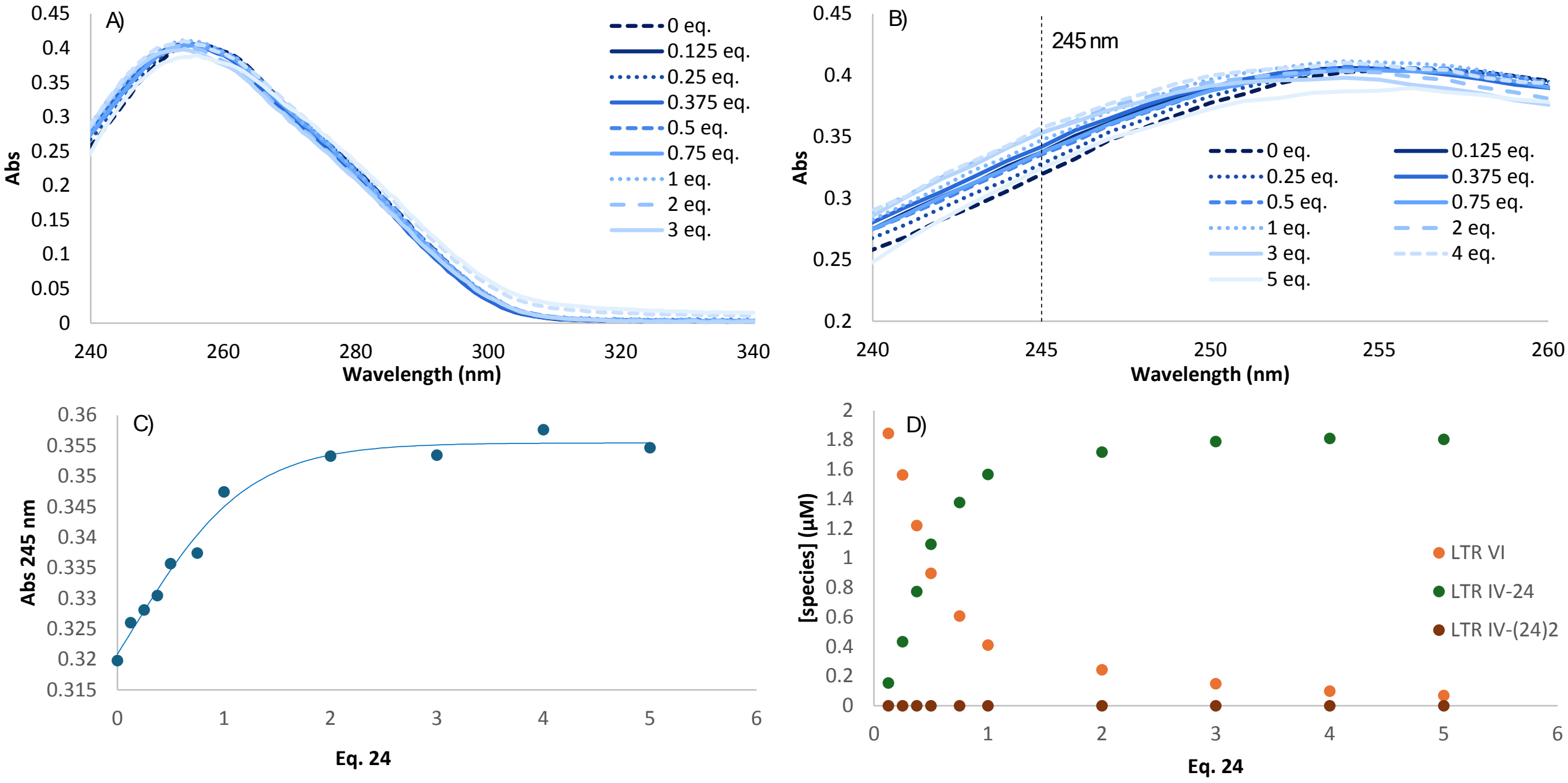

Figure S17. *UV-Vis titration of 2µM LTRIV with increasing equivalents of **24** (0-5 eq.) in 10 mM lithium cacodylate buffer (pH=7.2) containing 100 mM KCl. **A)** Full UV-Vis absorption spectra recorded in the 240-340 nm range. **B)** Magnified view of the 240-260 nm region highlighting the hyperchromic effect observed upon ligand addition; the dashed vertical line marks the wavelength (245nm) selected for the binding analysis. **C)** Experimental absorbance values at 245nm plotted against the equivalents of **24** (blue dots) and the corresponding non-linear fitting curve (solid line) based on a 1:1 binding model. **D)** Speciation plot derived from the fitting analysis showing the concentration profile of free G-quadruplex (LTR IV, orange dots), the 1:1 complex (LTR IV-**24**, green dots), and the negligible formation of higher-order stoichiometry species (LTR IV-(**24**)$_2$, brown dots) as a function of ligand equivalents.*

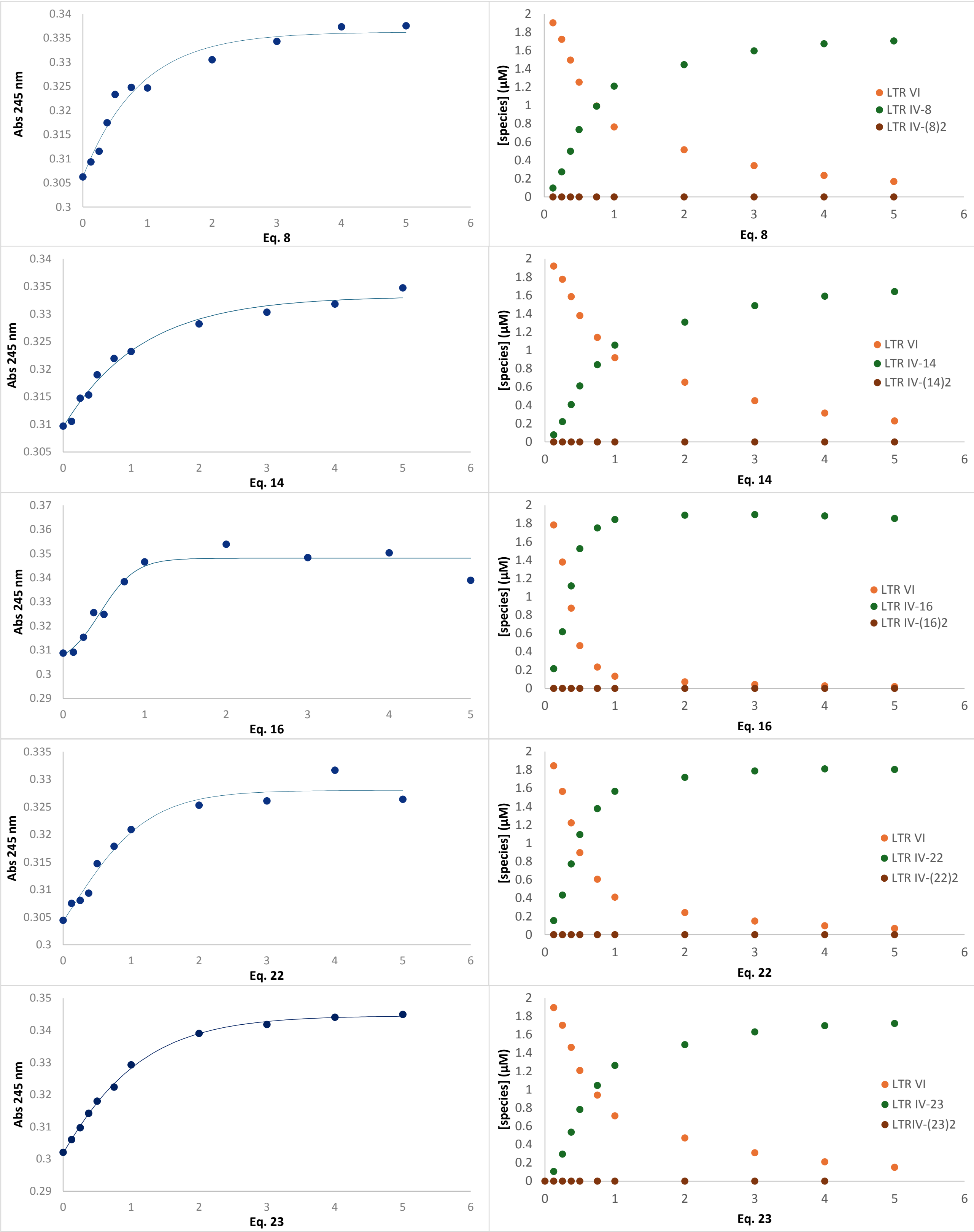

Figure S18. *UV-Vis titration of 2µM LTRIV with increasing equivalents of* ***8****,* ***14****,* ***16****,* ***22****, or* ***23*** *(0-5 eq.) in 10 mM lithium cacodylate buffer (pH=7.2) containing 100 mM KCl. The figure reports the experimental absorbance values at 245nm plotted against the equivalents of ligands (L) and the corresponding non-linear fitting curve based on a 1:1 binding model (left); and the speciation plot derived from the fitting analysis showing the concentration profile of free G-quadruplex (LTR*

*IV, orange dots), the 1:1 complex (LTR IV-**L**, green dots), and the negligible formation of higher-order stoichiometry species (LTR IV-(**L**)$_2$, brown dots) as a function of ligands equivalents (right).*

Analytical HPLC chromatograms

Ligand **8** - rt = 7.01 min, purity = 97.2%

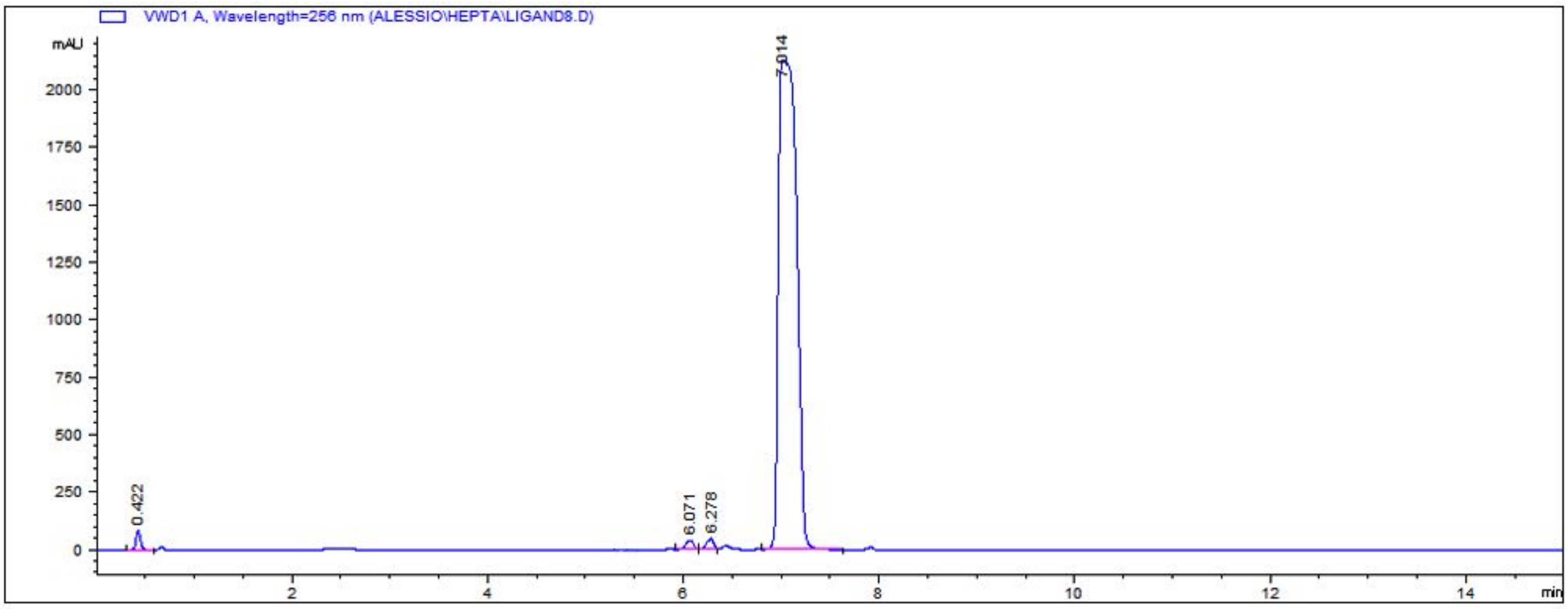

Ligand **14** – rt = 7.83 min, purity = 97.5%

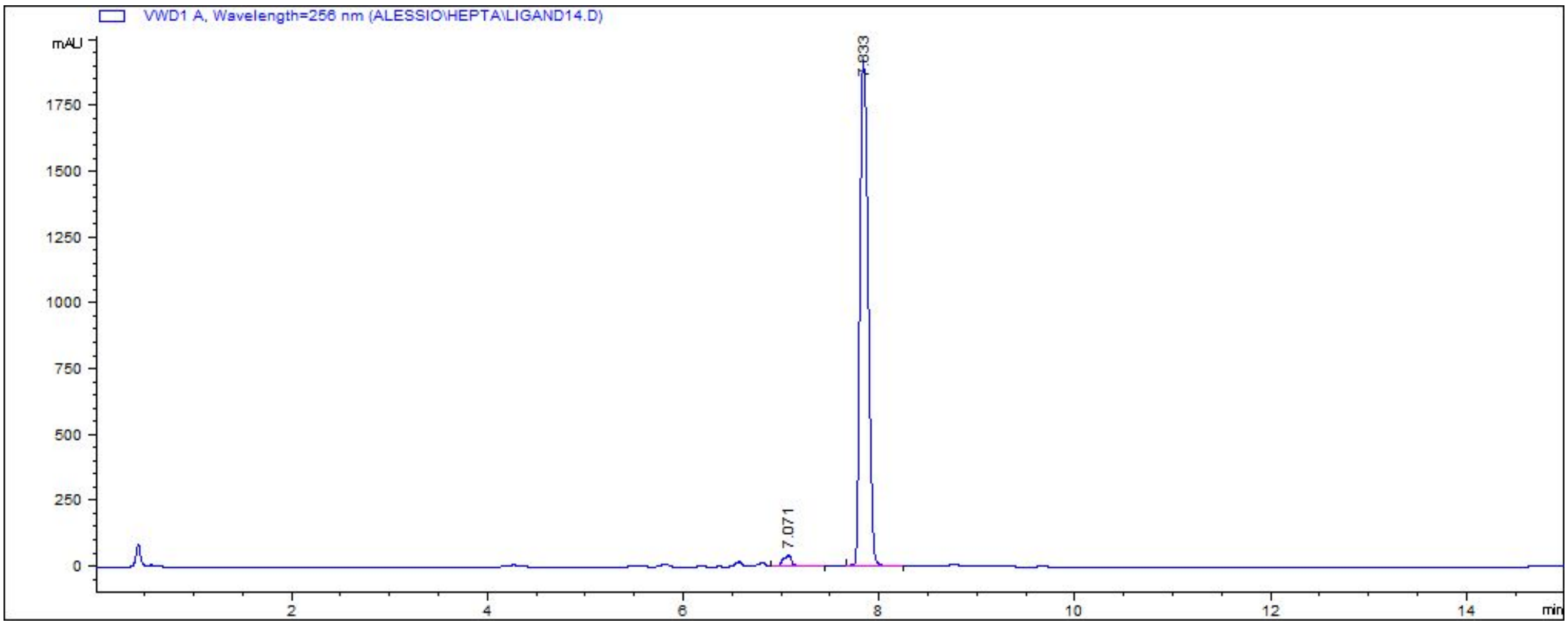

Ligand ***15*** *– rt = 6.69 min, purity = 98.6%*

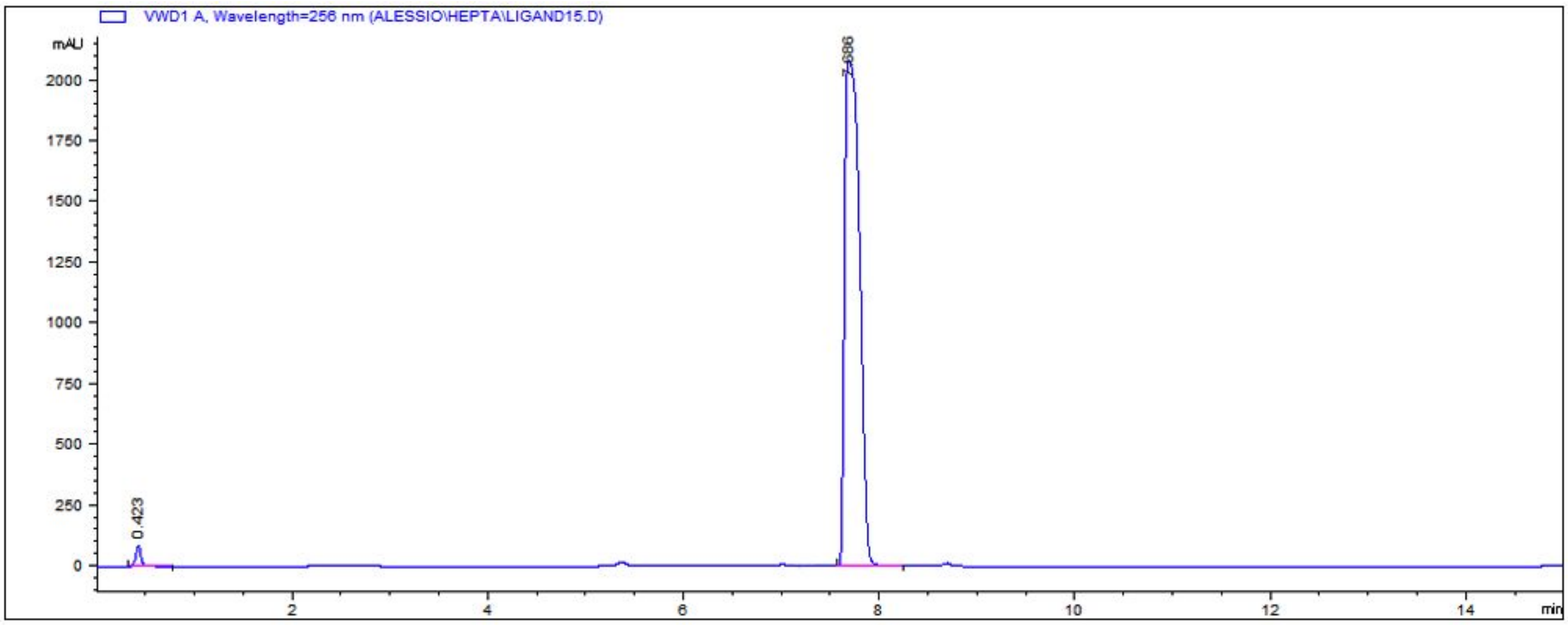

*Ligand **16** – rt = 8.53 min, purity = 97.9%*

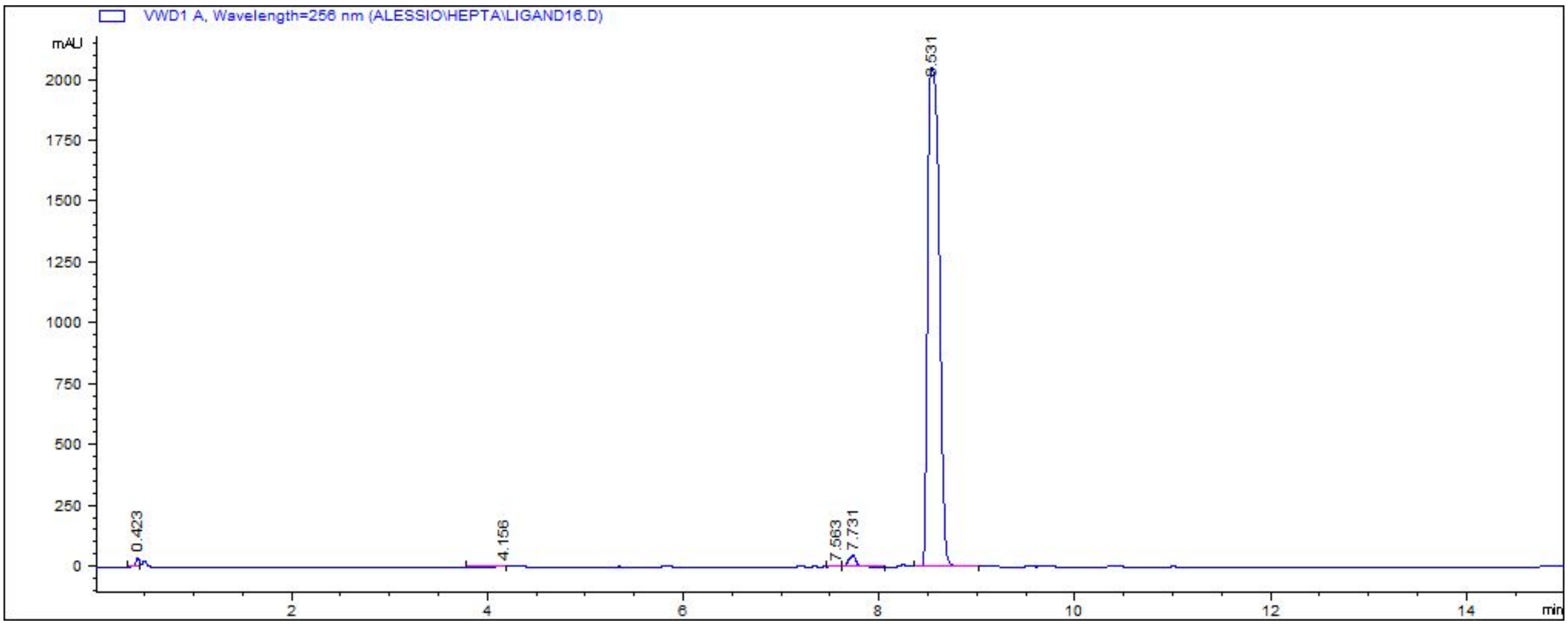

*Ligand **21** – rt = 8.08 min, purity = 96.9%*

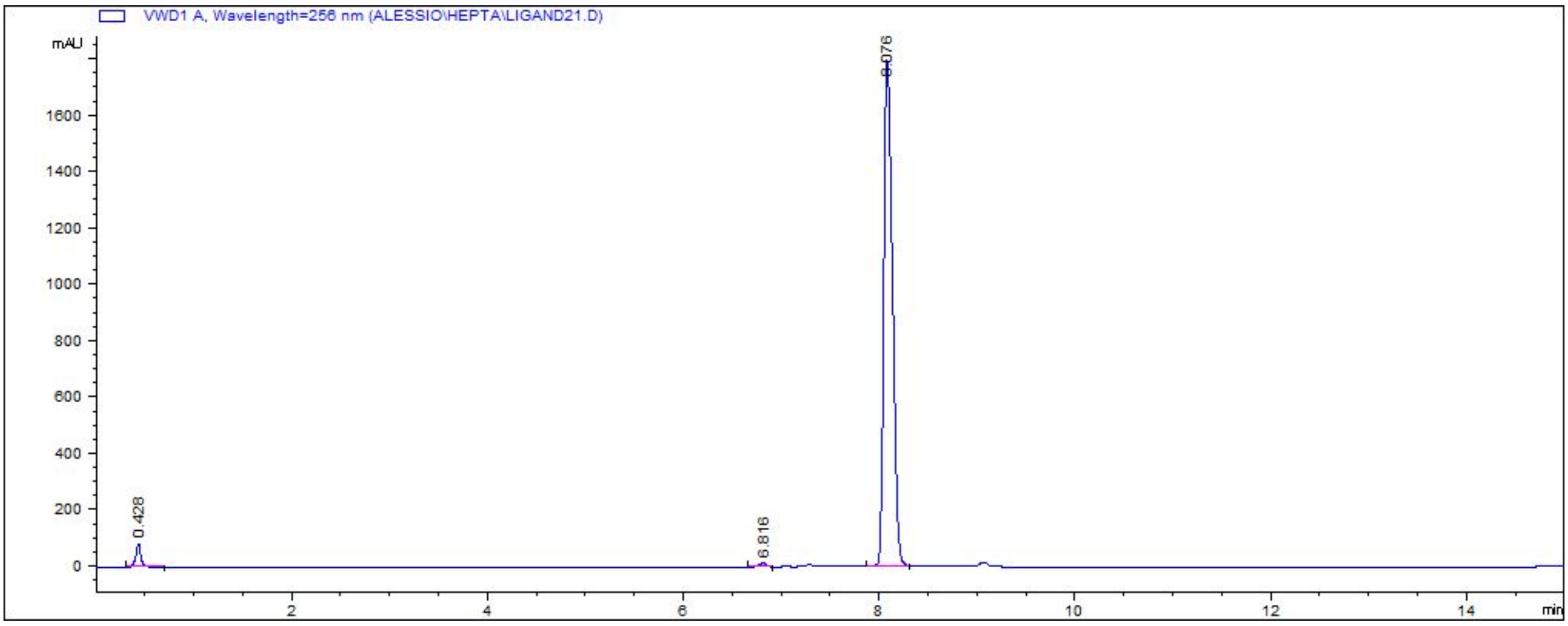

*Ligand **22** – rt = 8.77 min, purity = 97.6%*

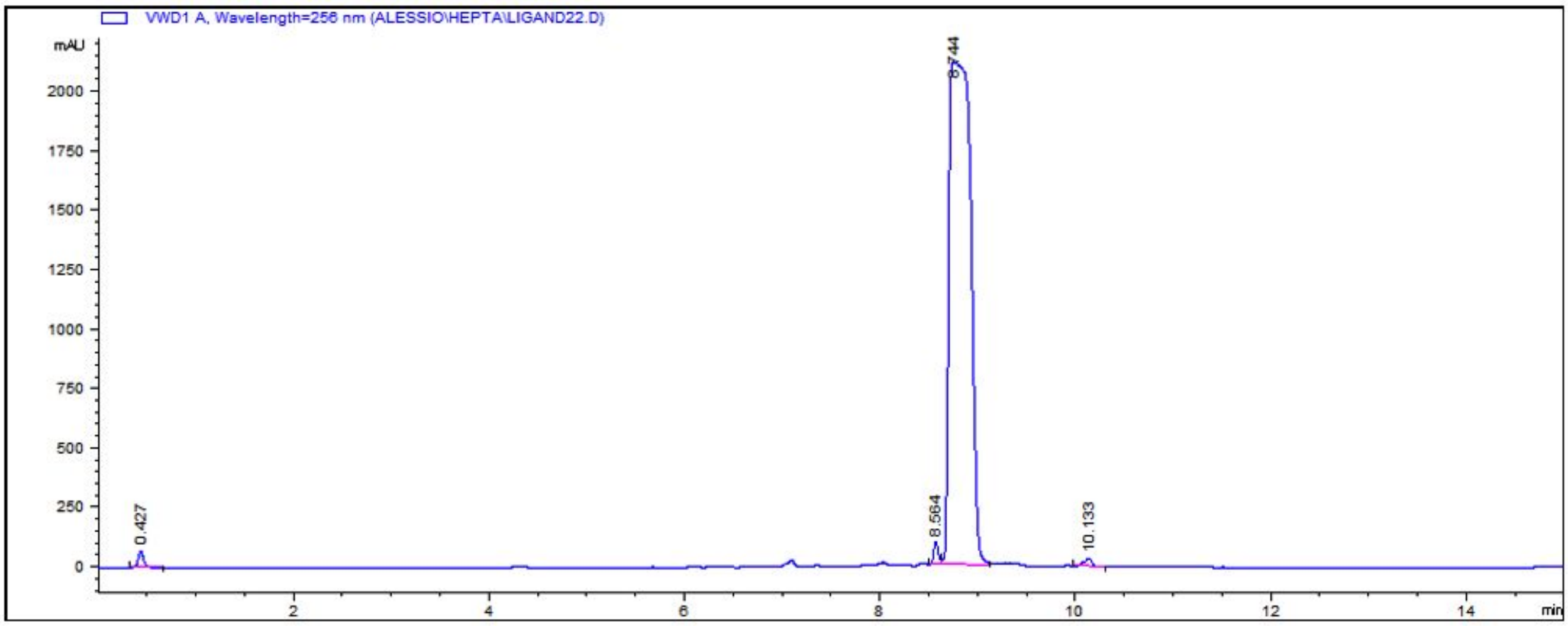

*Ligand **23** – rt = 7.12 min, purity = 97.3%*

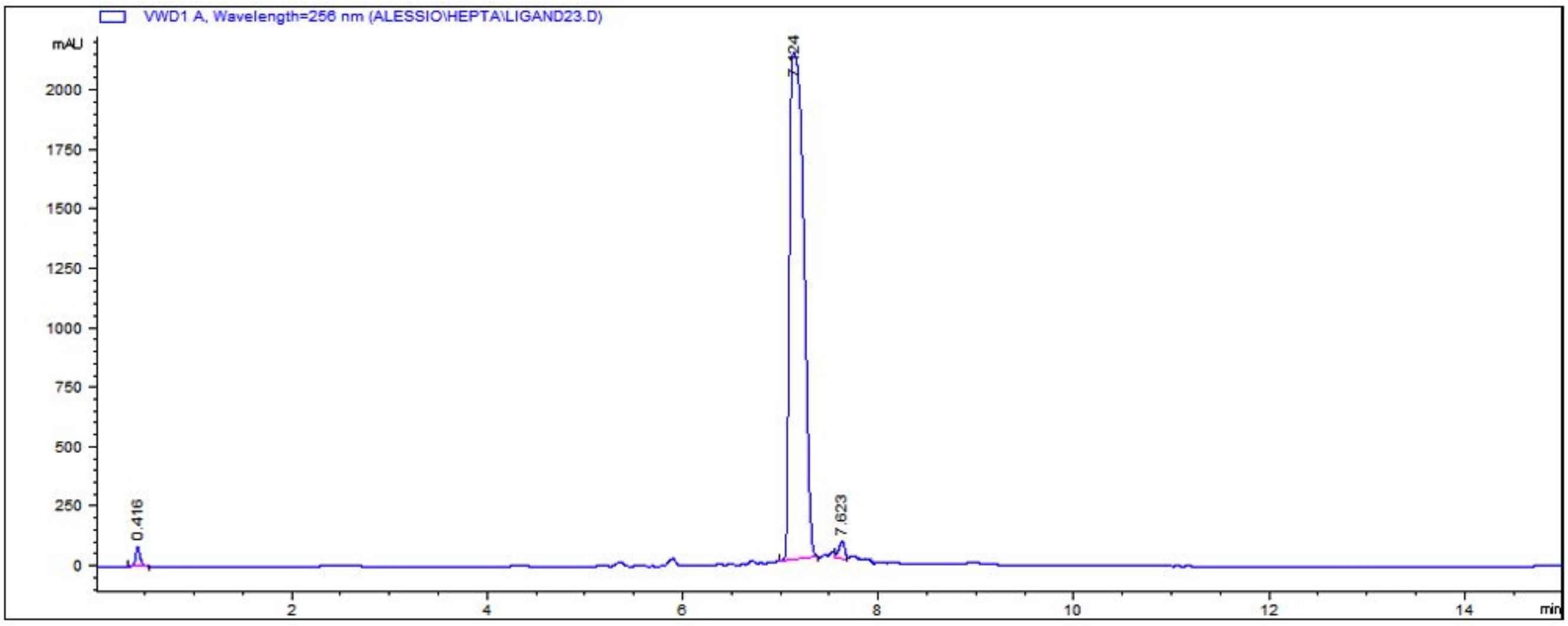

*Ligand **24** – rt = 7.67 min, purity = 96.8%*

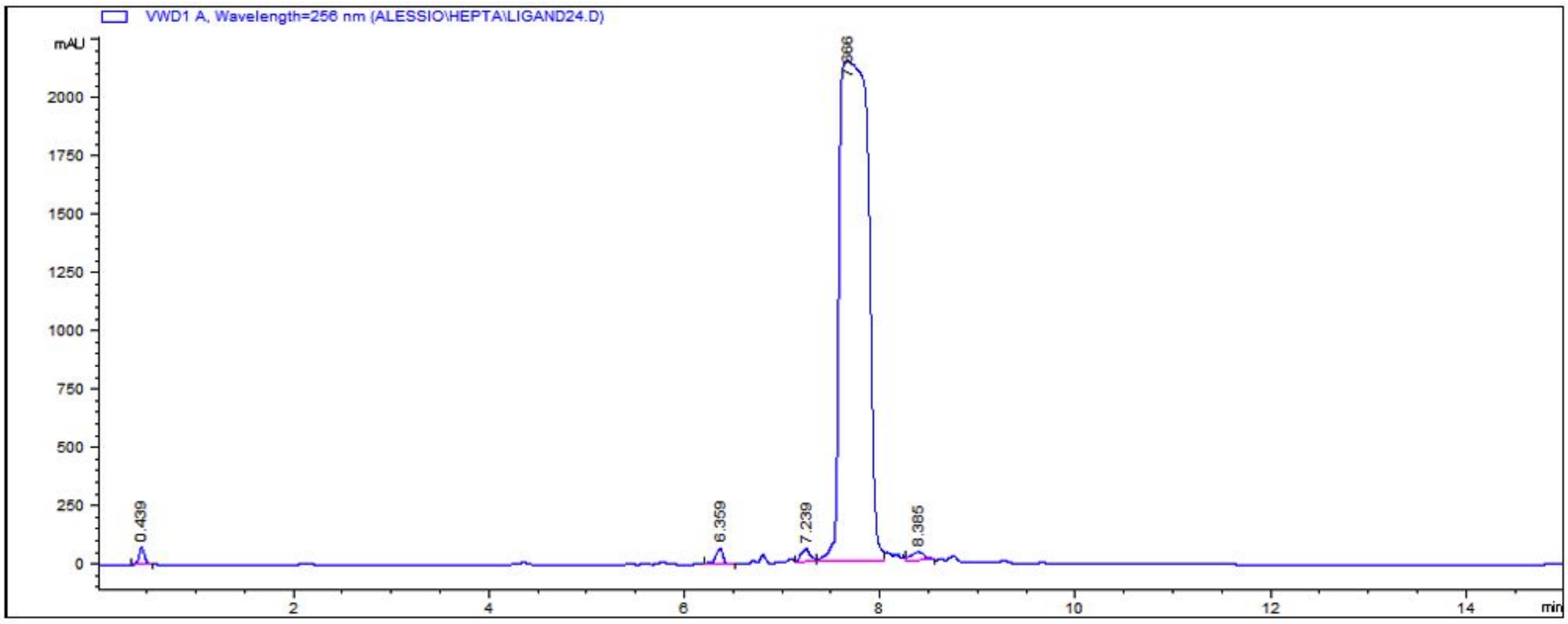

^{1}H and ^{13}C NMR spectra

*^{1}H NMR spectrum of compound **2** recorded in DMSO-d6 at 25°C, 300 MHz.*

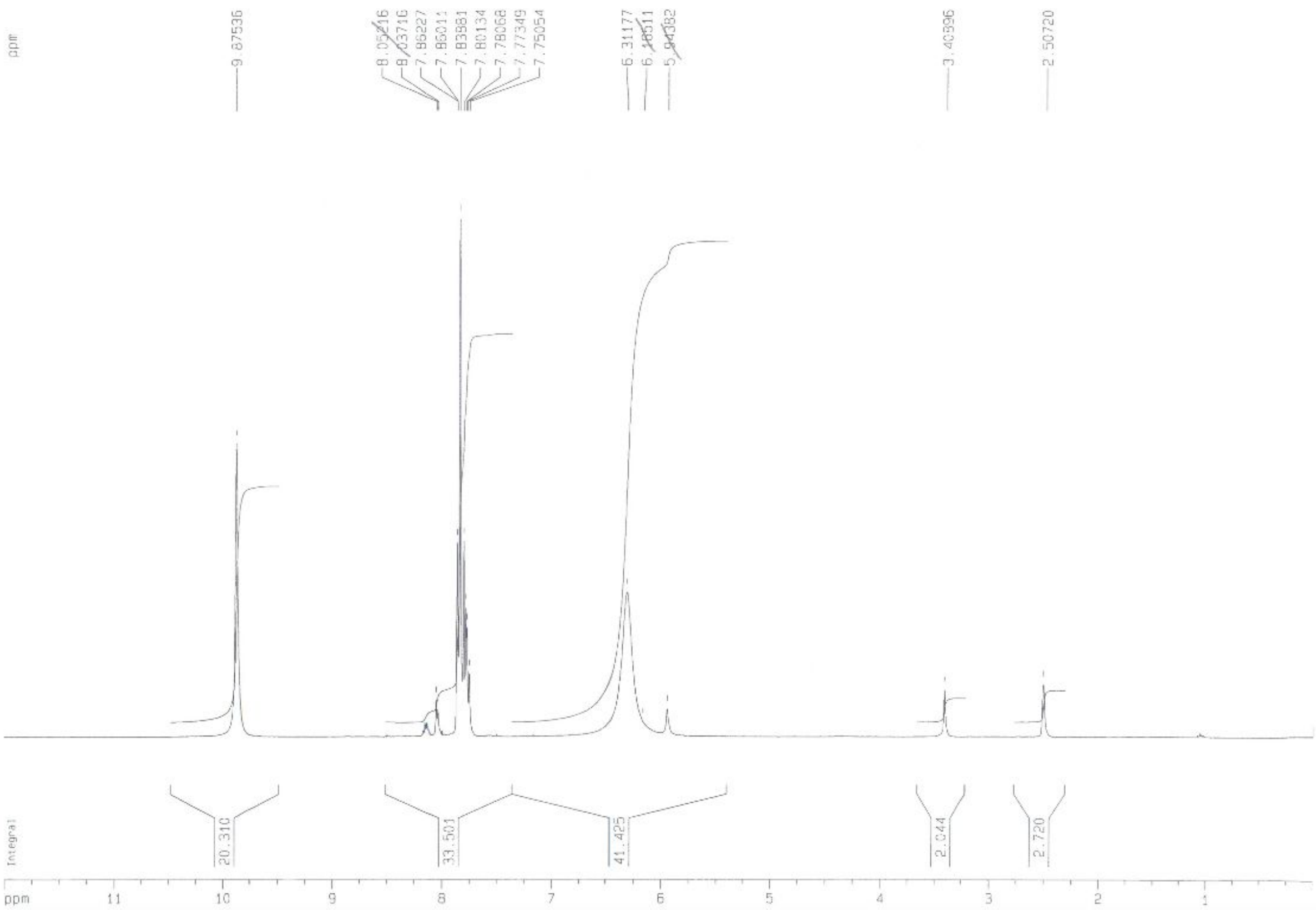

*$^{13}C\{1H\}$ NMR spectrum of compound **2** recorded in DMSO-d6 at 25°C, 75 MHz.*

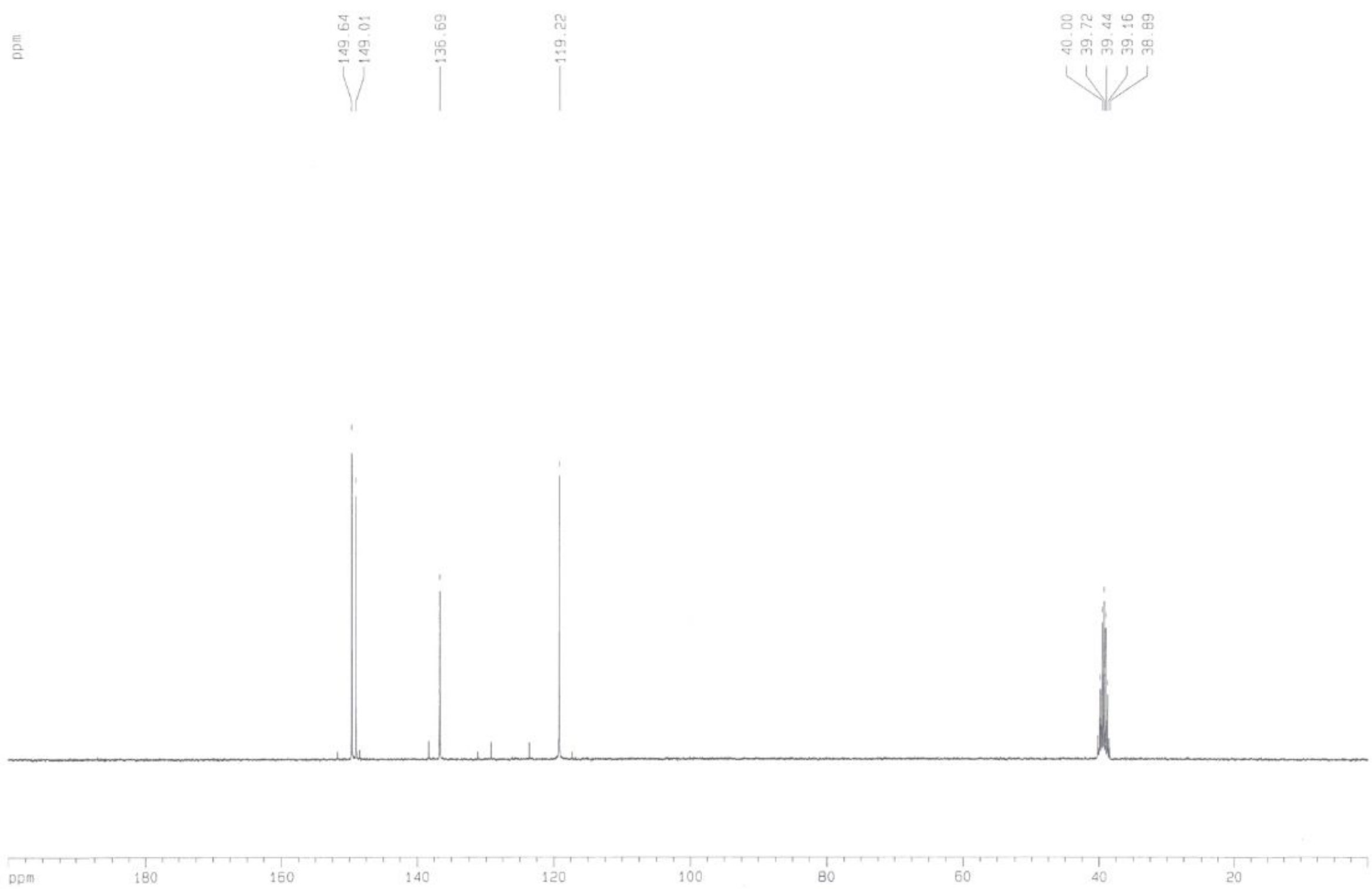

*^{1}H NMR spectrum of compound **10** recorded in CD_3OD at 25°C, 300 MHz.*

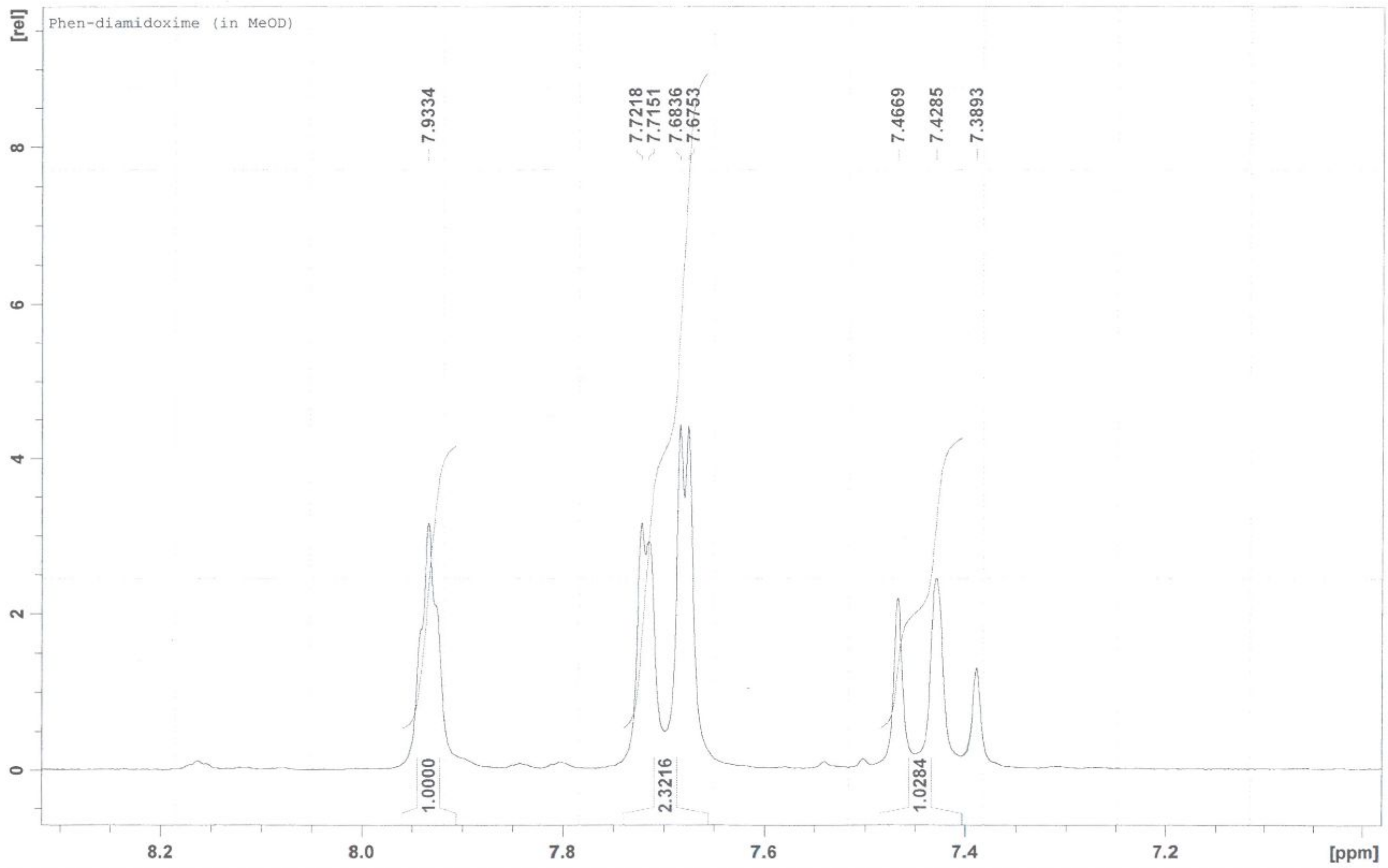

*^{1}H NMR spectrum of compound **18** recorded in DMSO-d6 at 25°C, 300 MHz.*

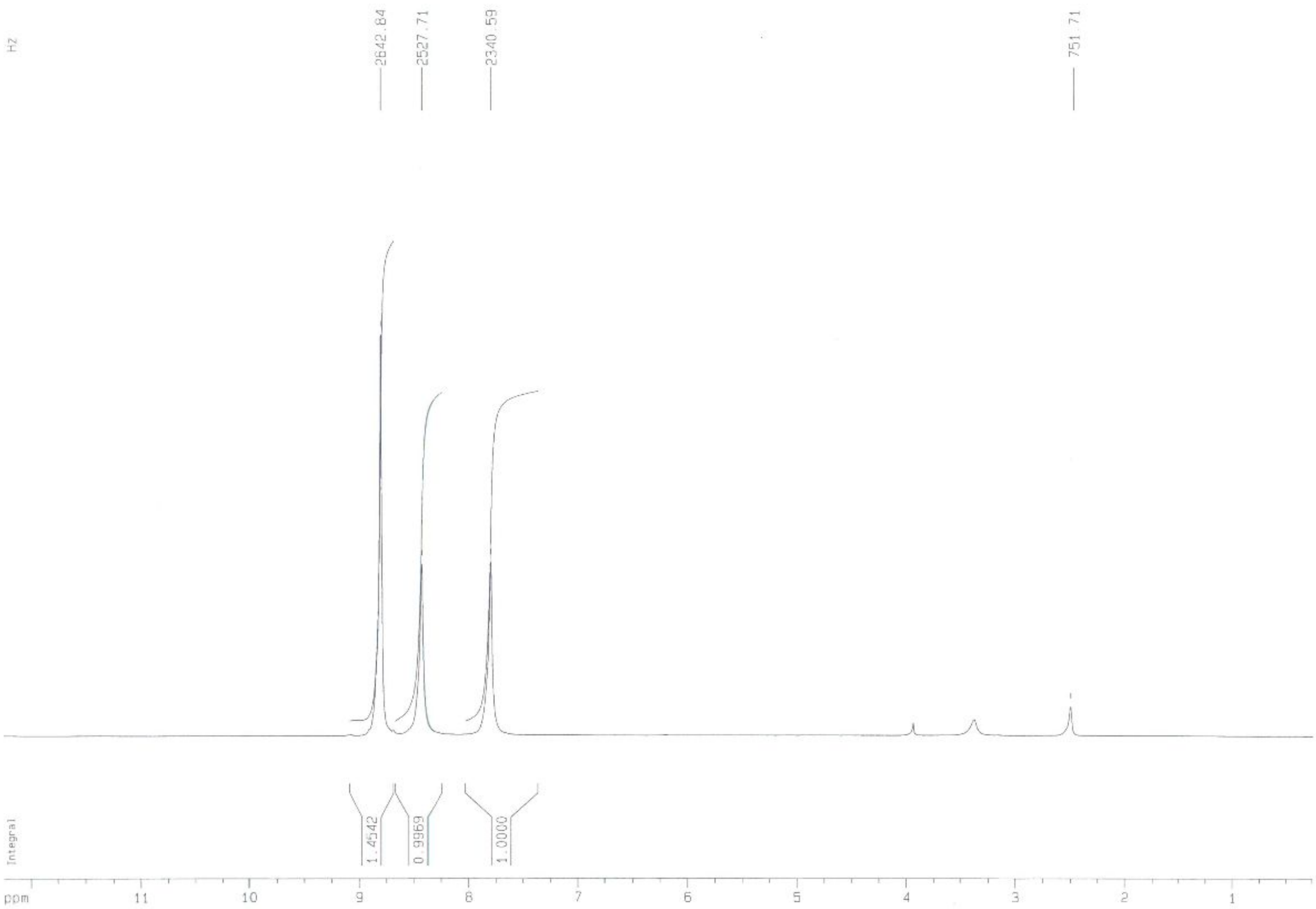

^{13}C{1H} NMR spectrum of compound ***18*** *recorded in DMSO-d6 at 25°C, 75 MHz.*

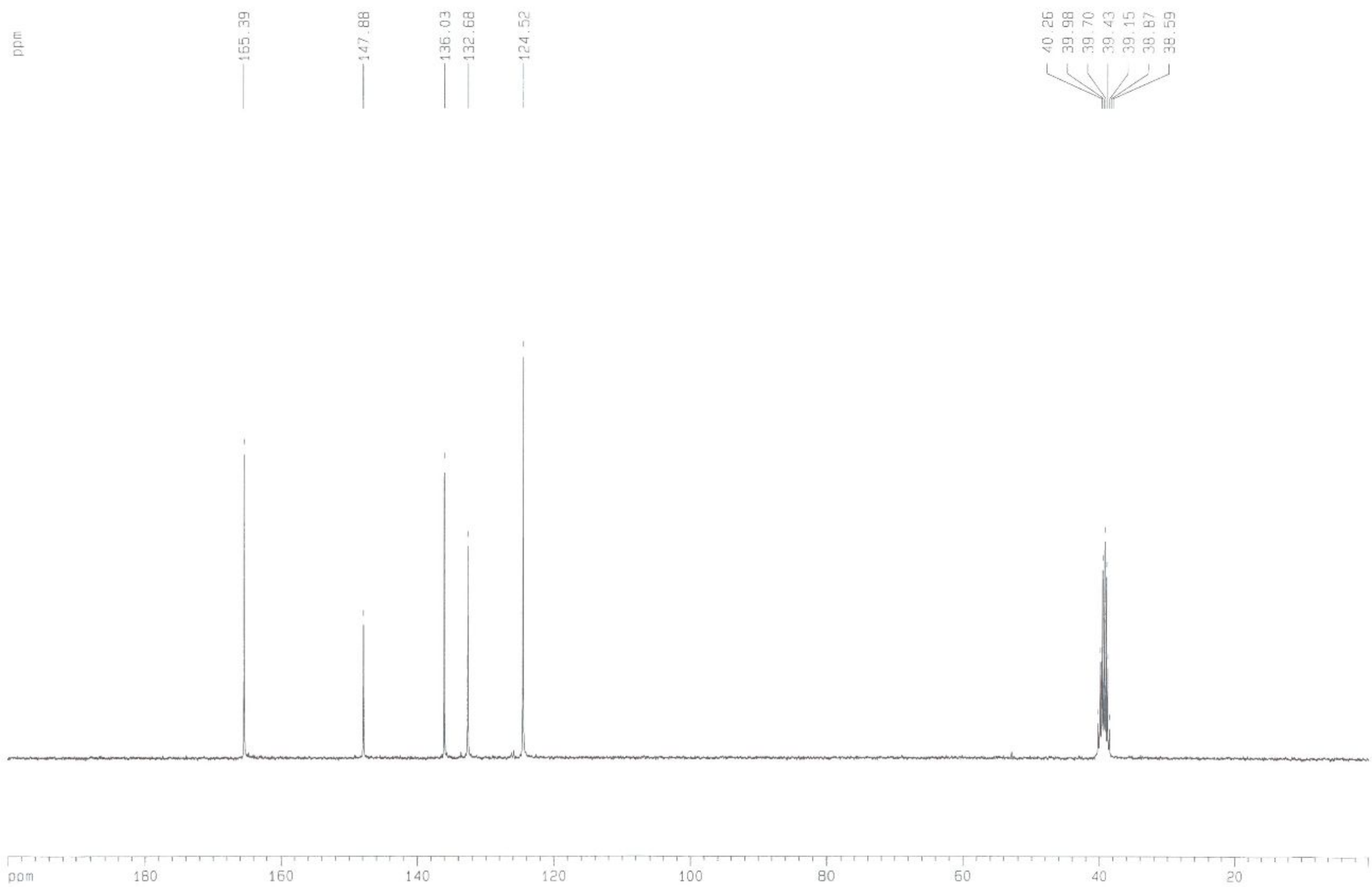

*^{1}H NMR spectrum of compound **19** recorded in DMSO-d6 at 25°C, 300 MHz.*

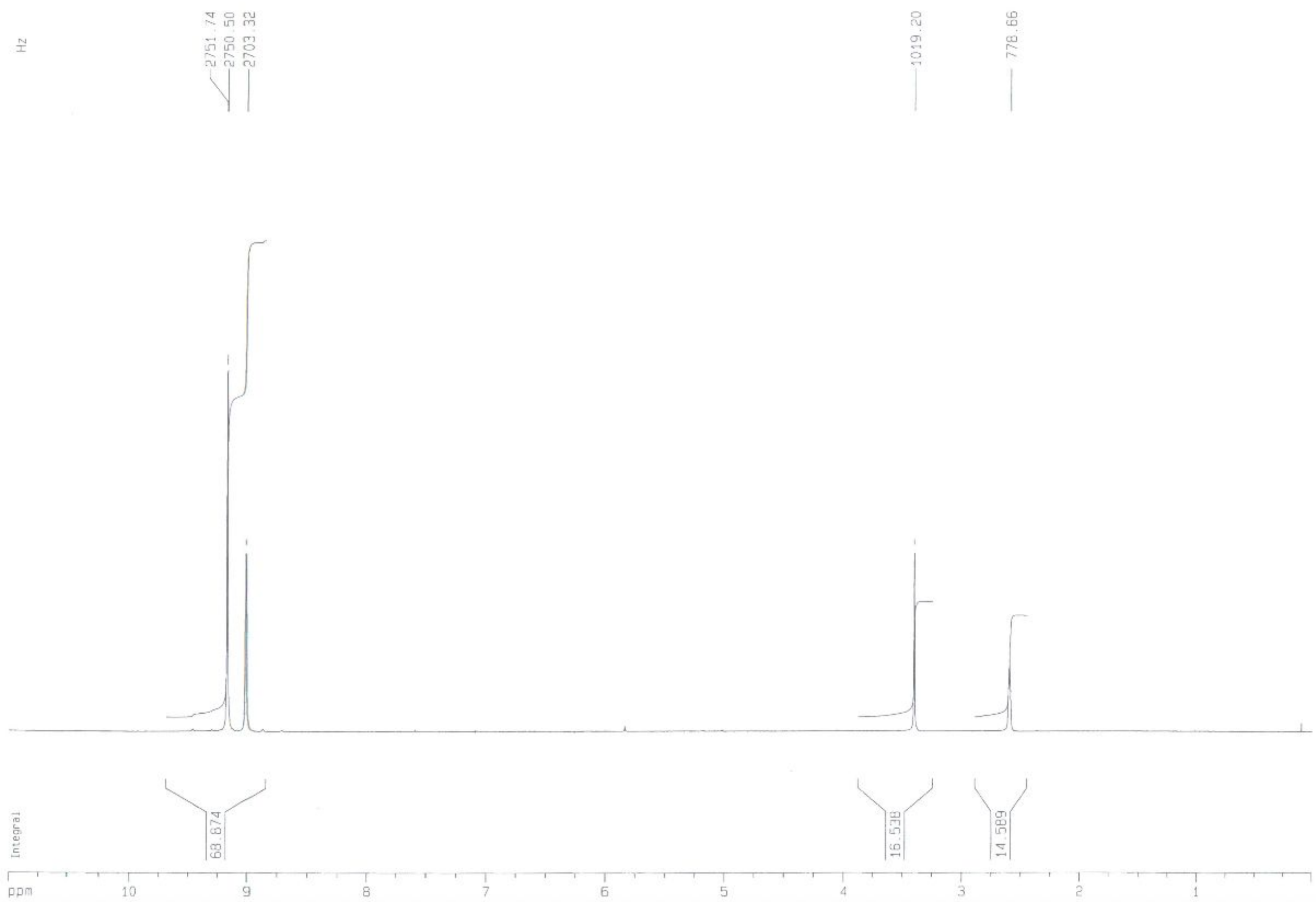

*^{13}C{1H} NMR spectrum of compound **19** recorded in DMSO-d6 at 25°C, 75 MHz.*

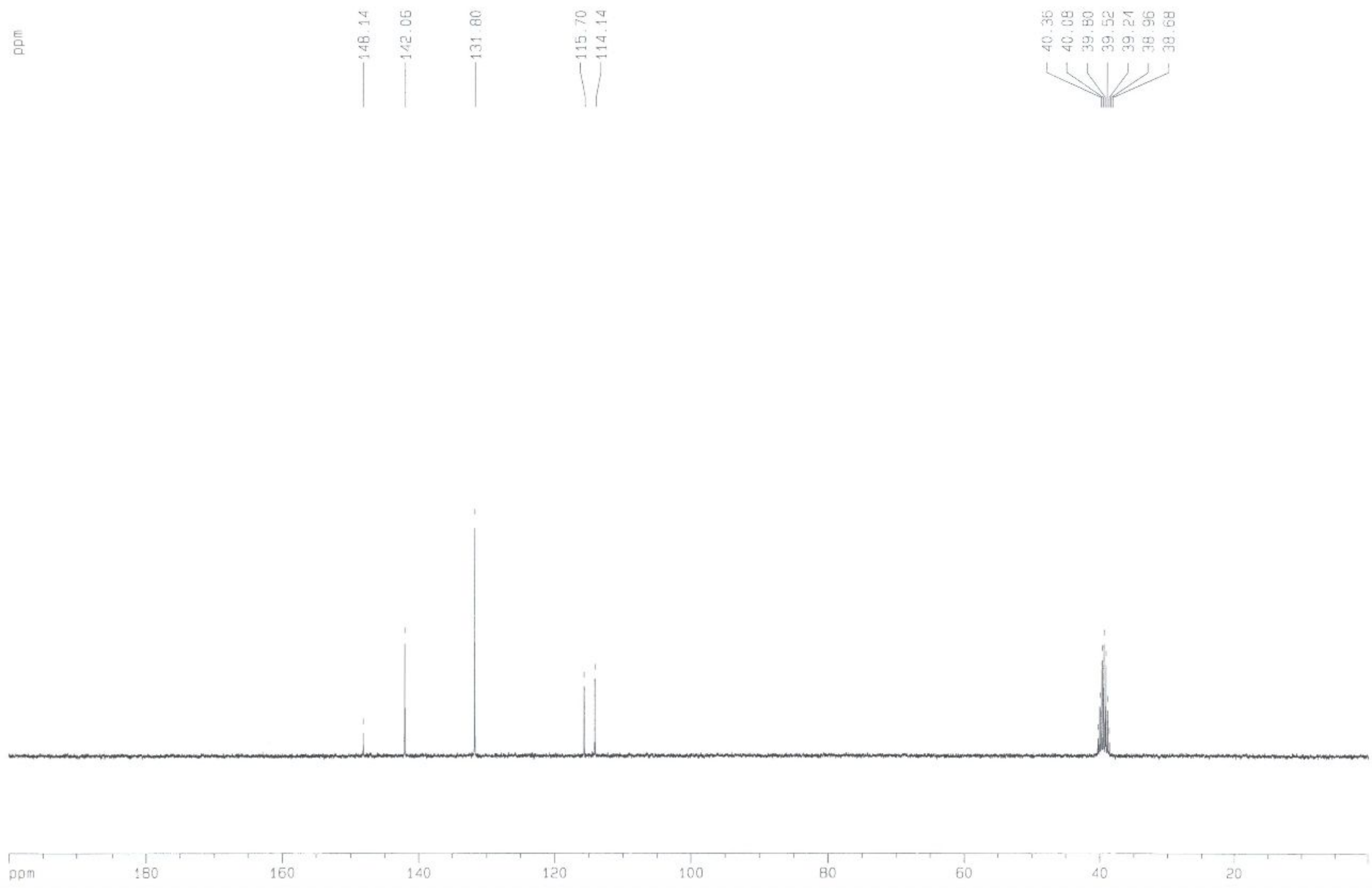

*^{1}H NMR spectrum of compound **20** recorded in DMSO-d6 at 25°C, 300 MHz.*

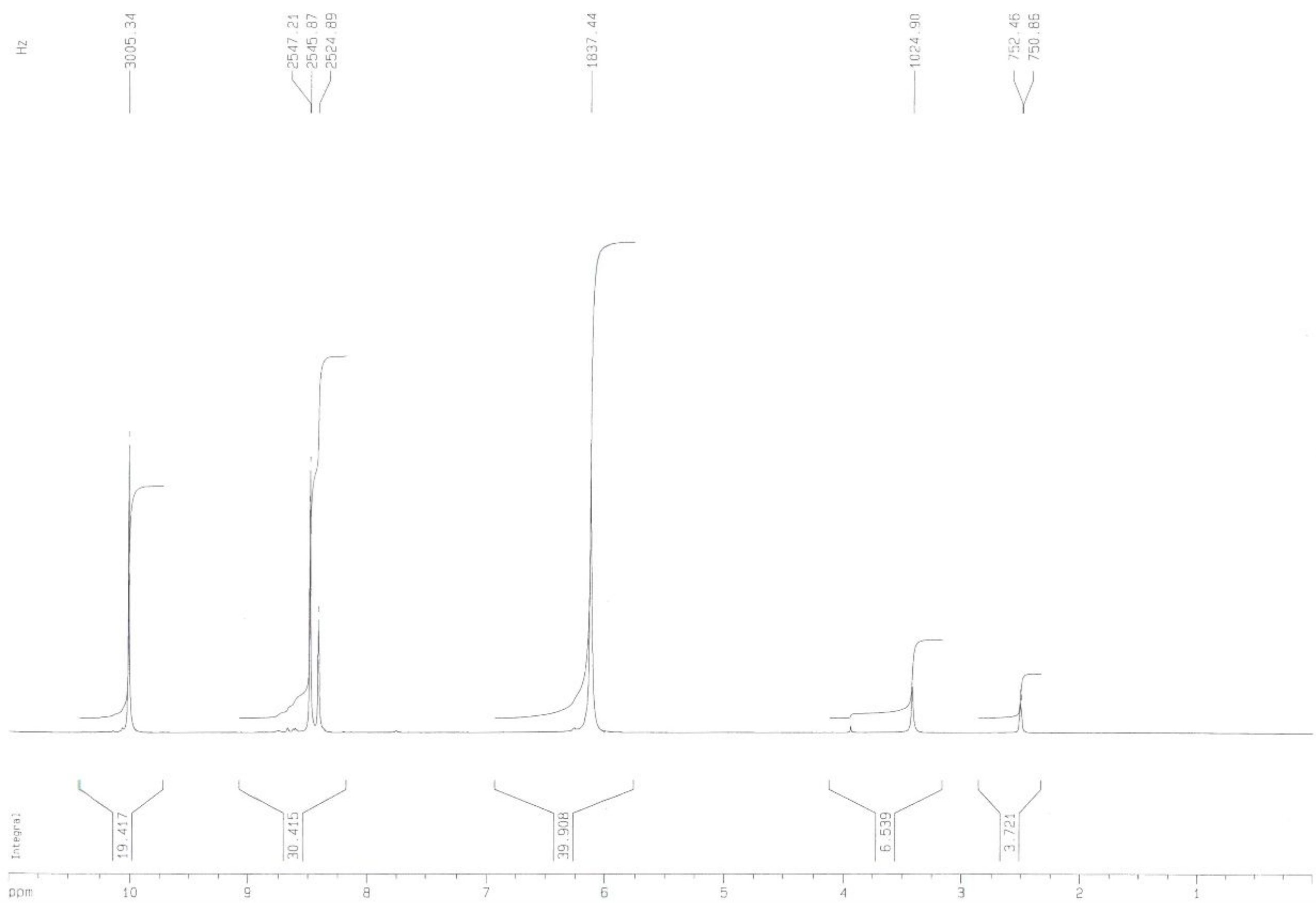

$^{13}C\{^1H\}$ *NMR spectrum of compound* ***20*** *recorded in DMSO-d6 at 25°C, 75 MHz.*

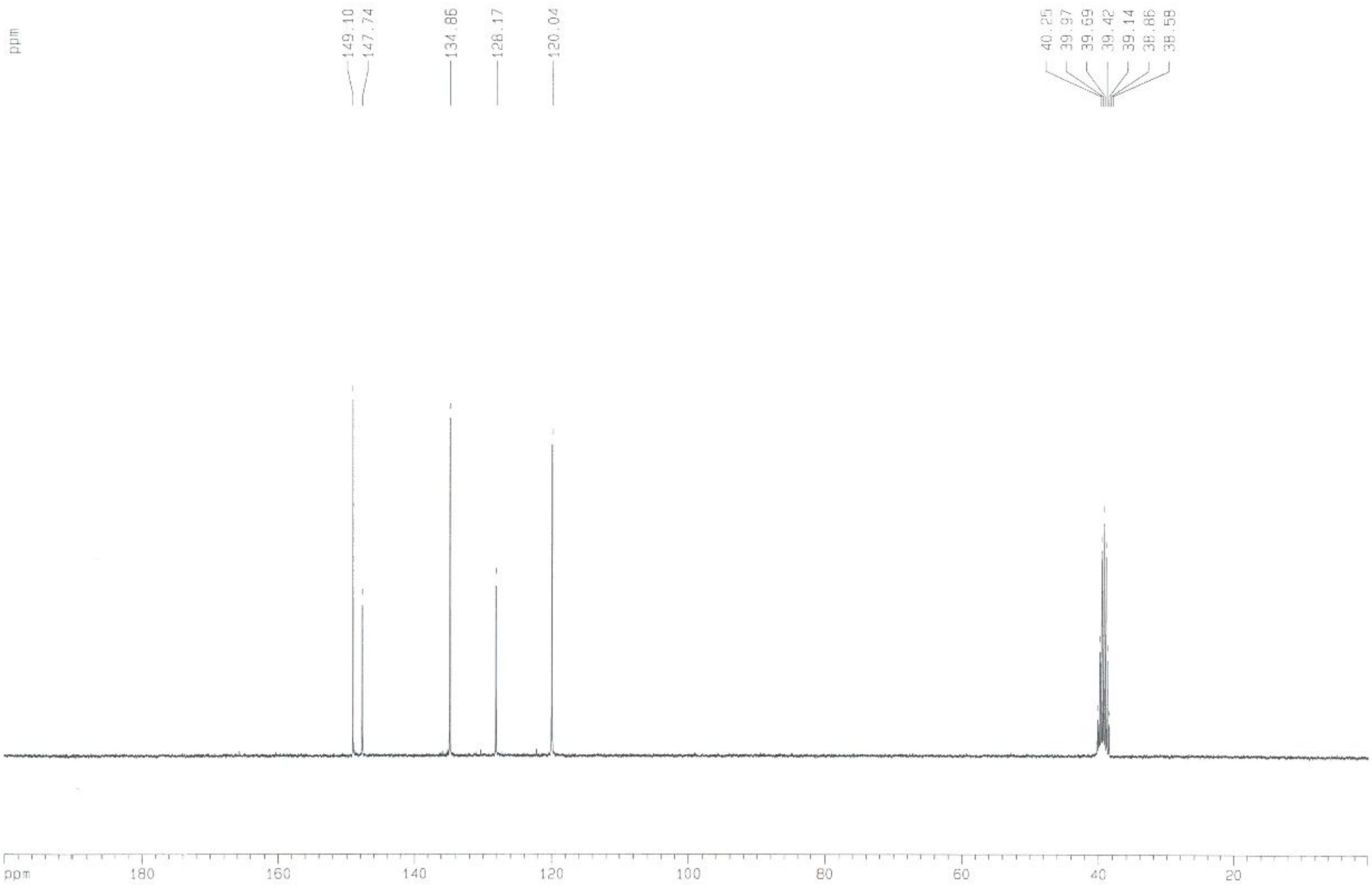

^{1}H NMR spectrum of compound **4** recorded in CD_3OD at 25°C, 300 MHz.

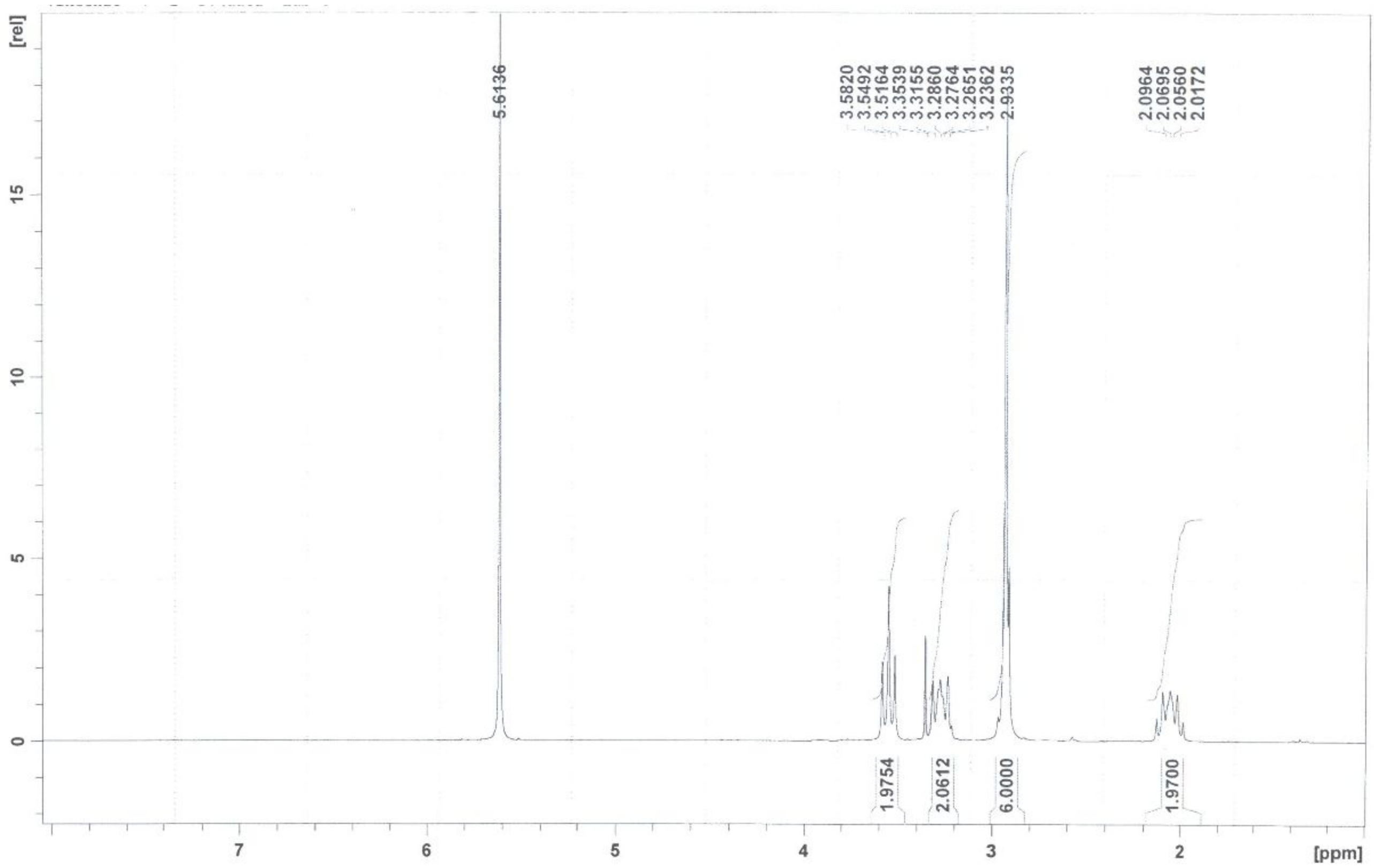

^{1}H NMR spectrum of compound **5a** recorded in $CDCl_3$ at 25°C, 300 MHz.

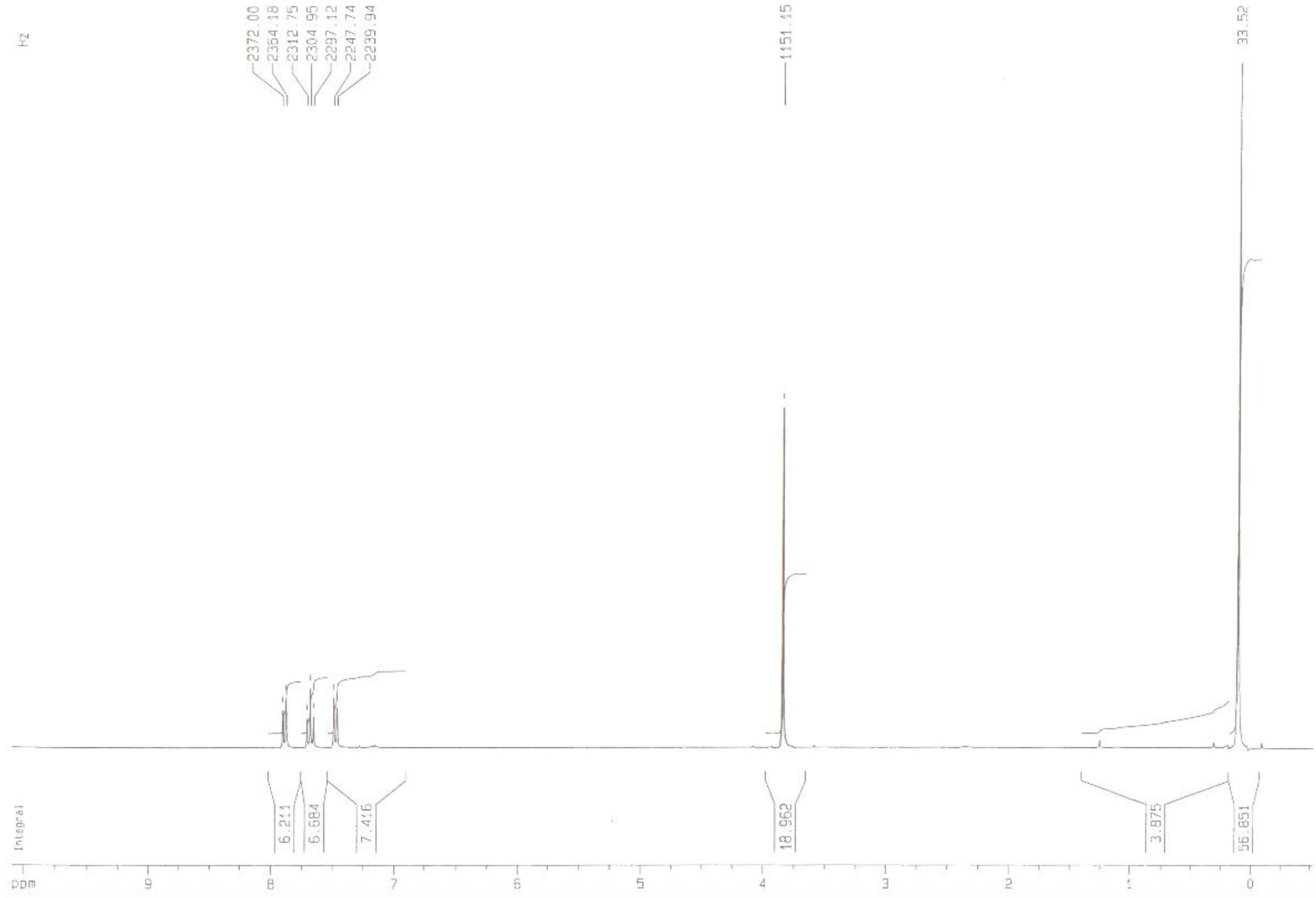

$^{13}C\{1H\}$ NMR spectrum of compound 5a recorded in $CDCl_3$ at 25°C, 75 MHz.

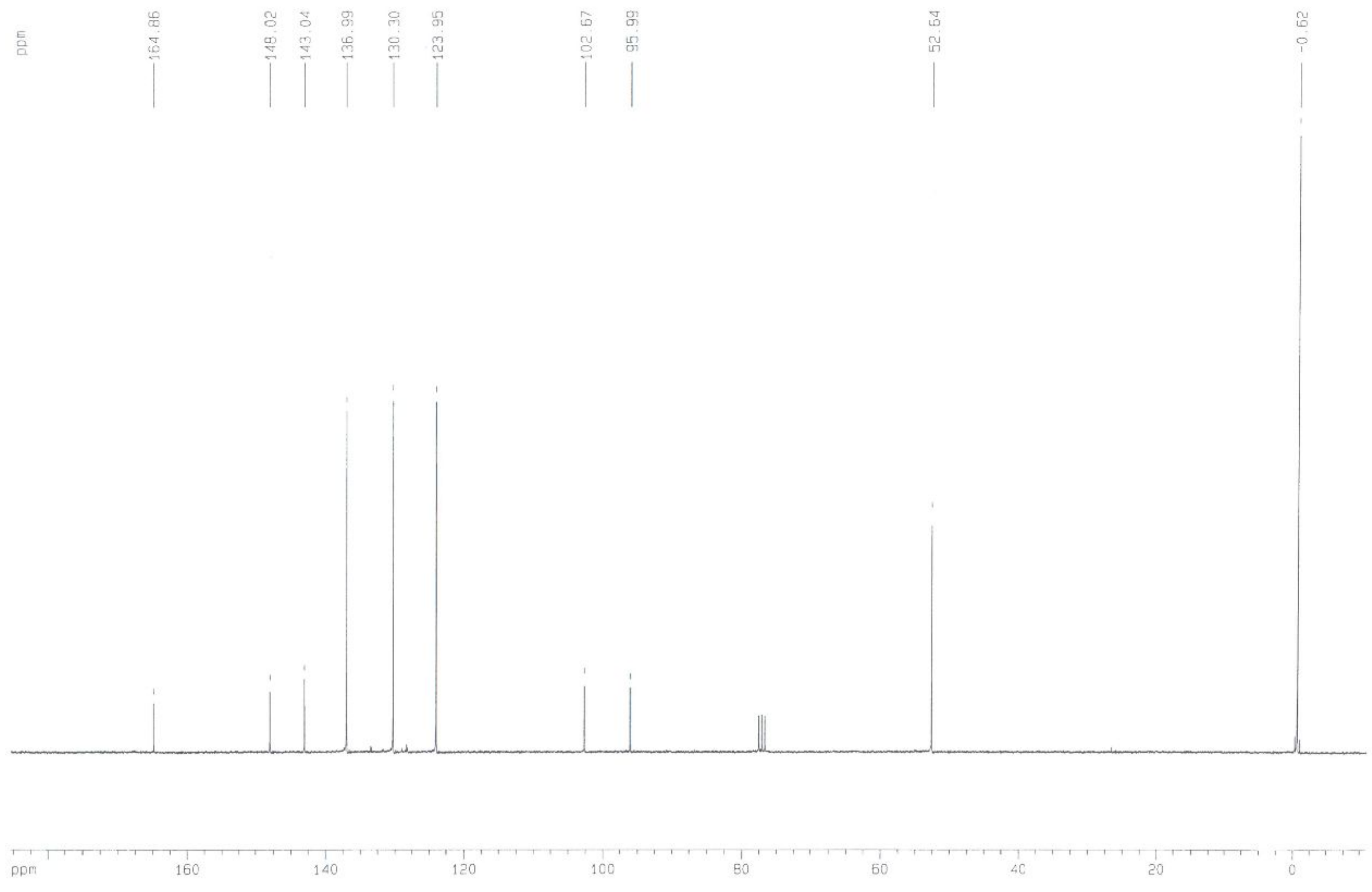

*^{1}H NMR spectrum of compound **6** recorded in $CDCl_3$ at 25°C, 300 MHz.*

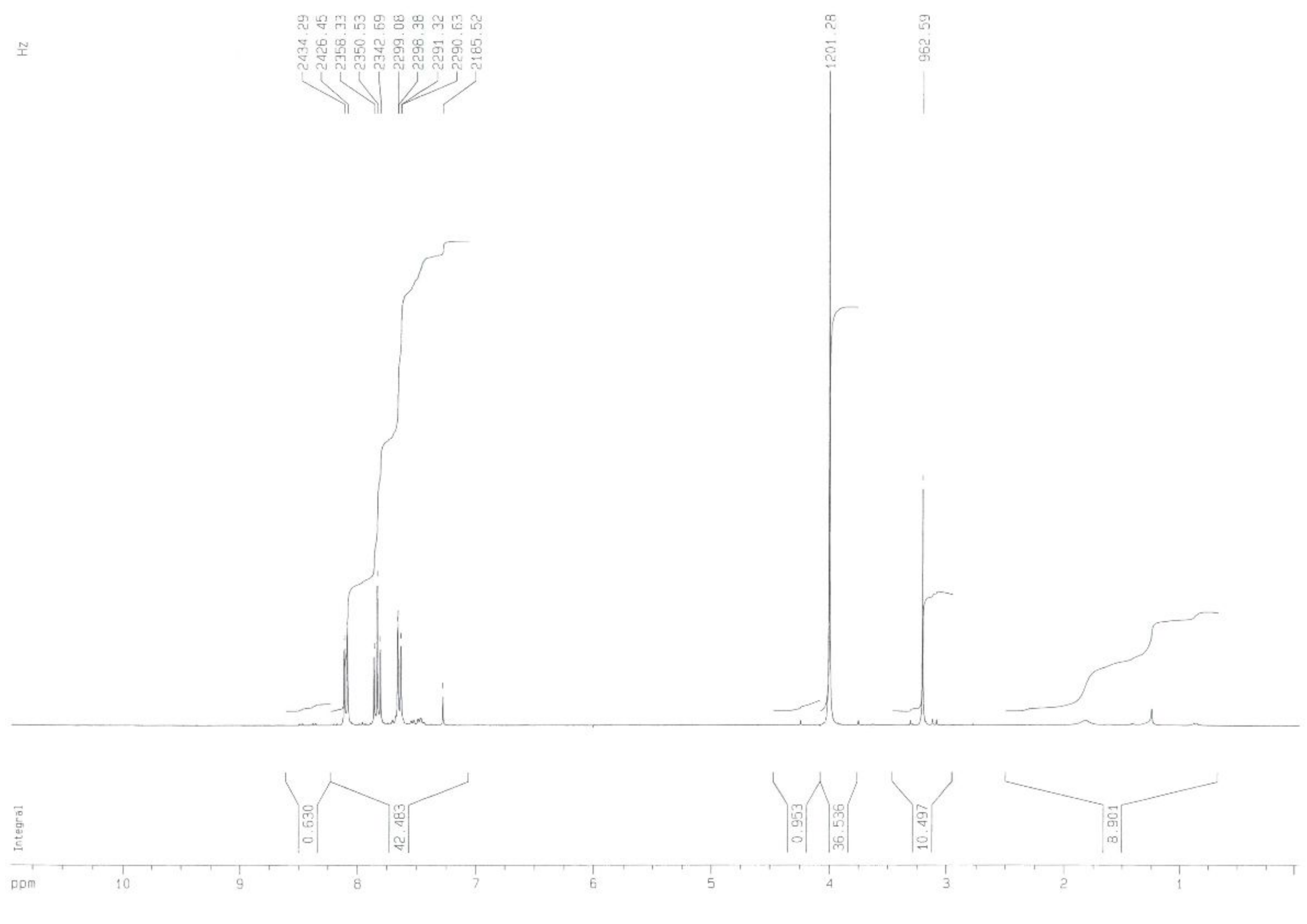

^{13}C{1H} NMR spectrum of compound ***6*** *recorded in* $CDCl_3$ *at 25°C, 75 MHz.*

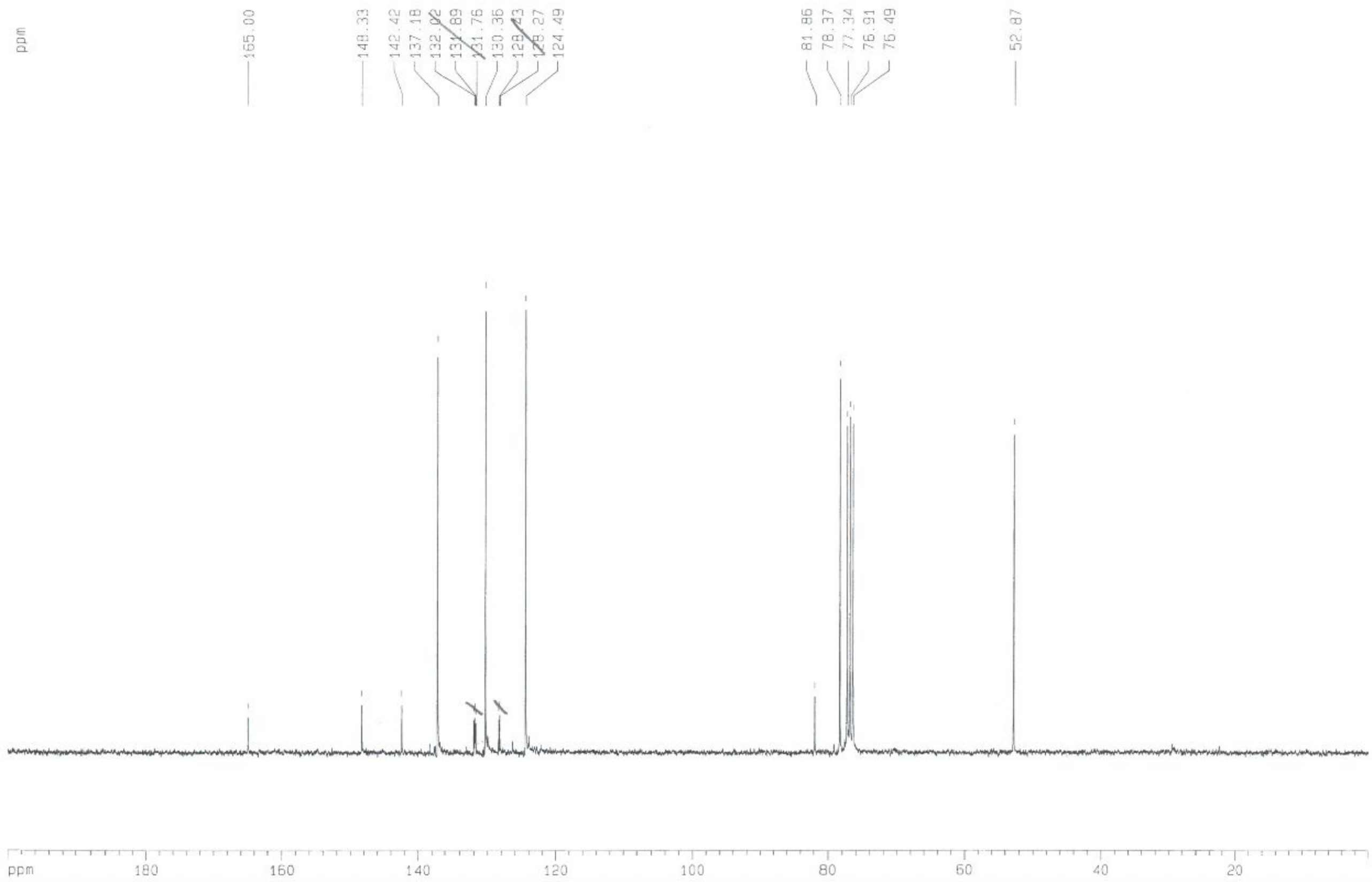

^{1}H NMR spectrum of compound ***6a*** *recorded in* $CDCl_3$ *at 25°C, 300 MHz.*

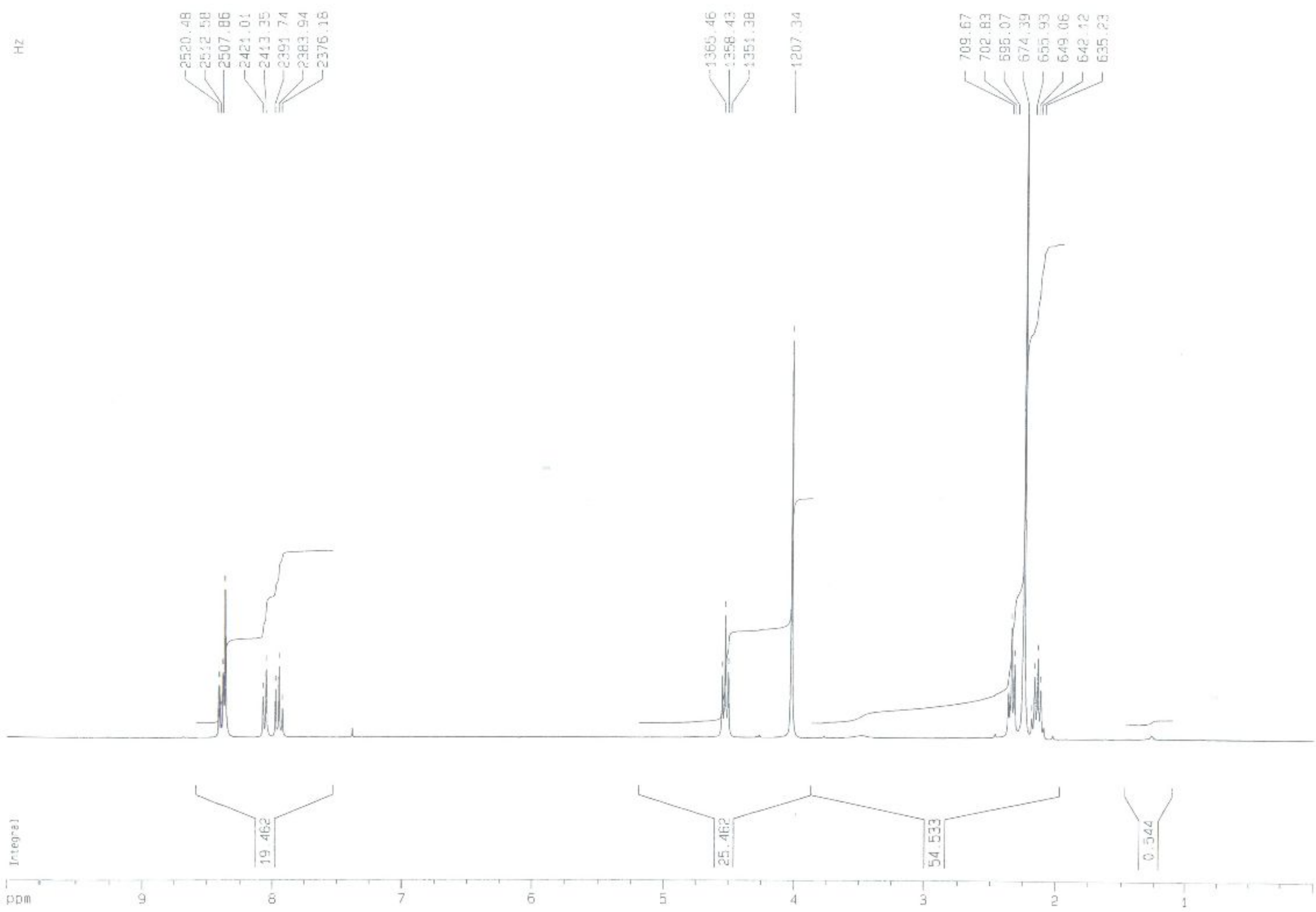

$^{13}C\{1H\}$ *NMR spectrum of compound* ***6a*** *recorded in* $CDCl_3$ *at 25°C, 75 MHz.*

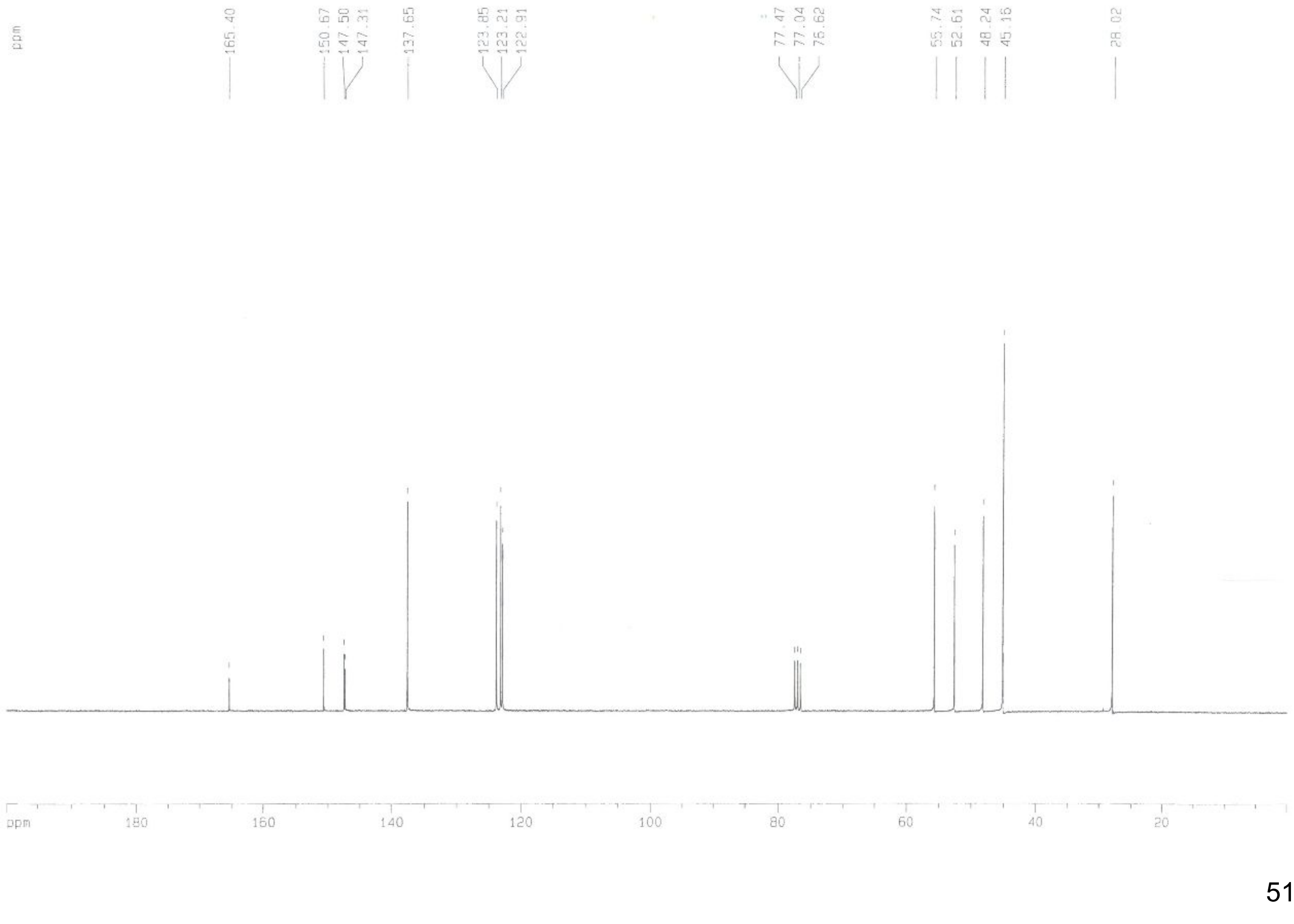

^{1}H NMR spectrum of compound ***12a*** *recorded in $CDCl_3$ at 25°C, 300 MHz.*

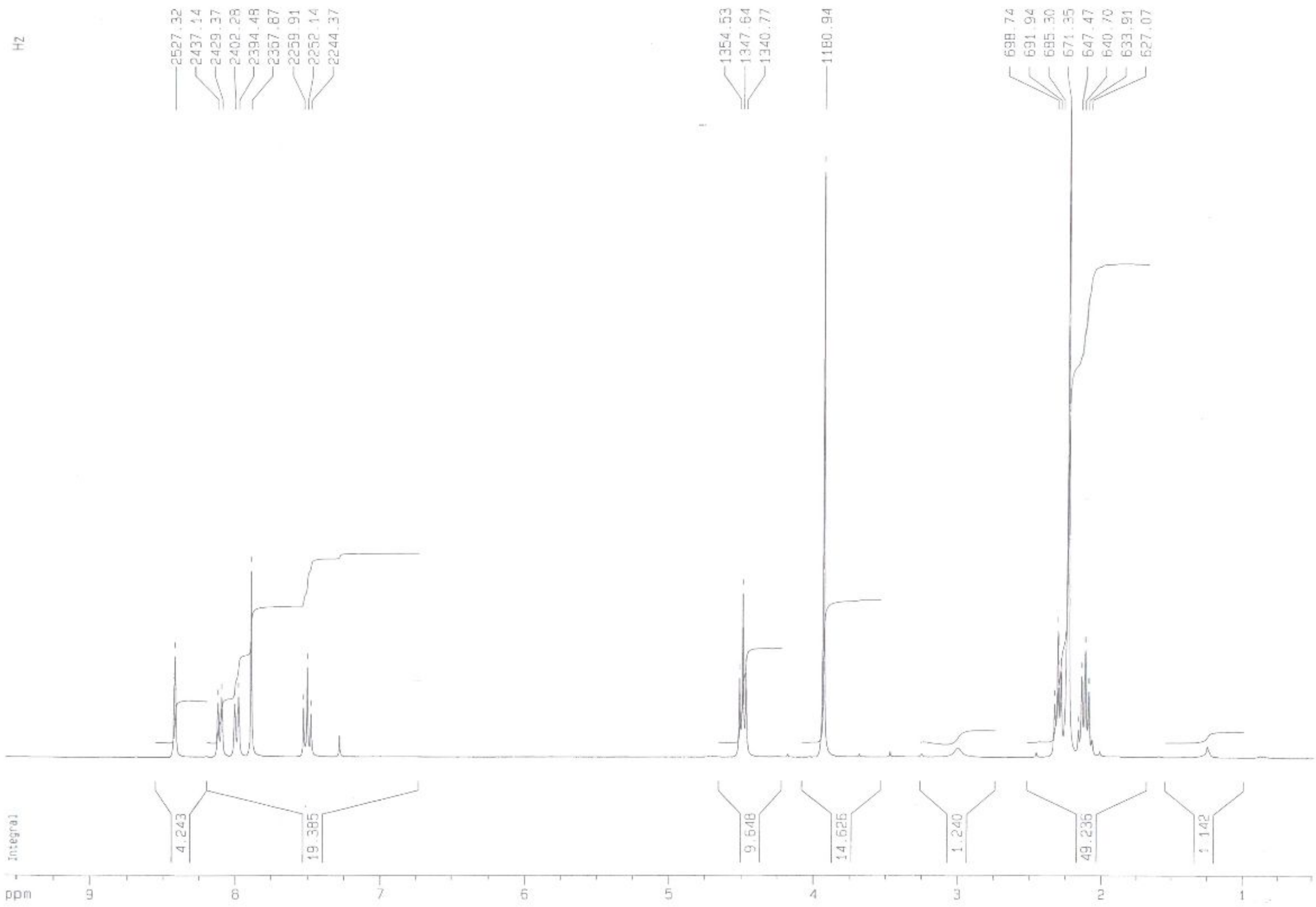

$^{13}C\{1H\}$ NMR spectrum of compound ***12a*** *recorded in $CDCl_3$ at 25°C, 75 MHz.*

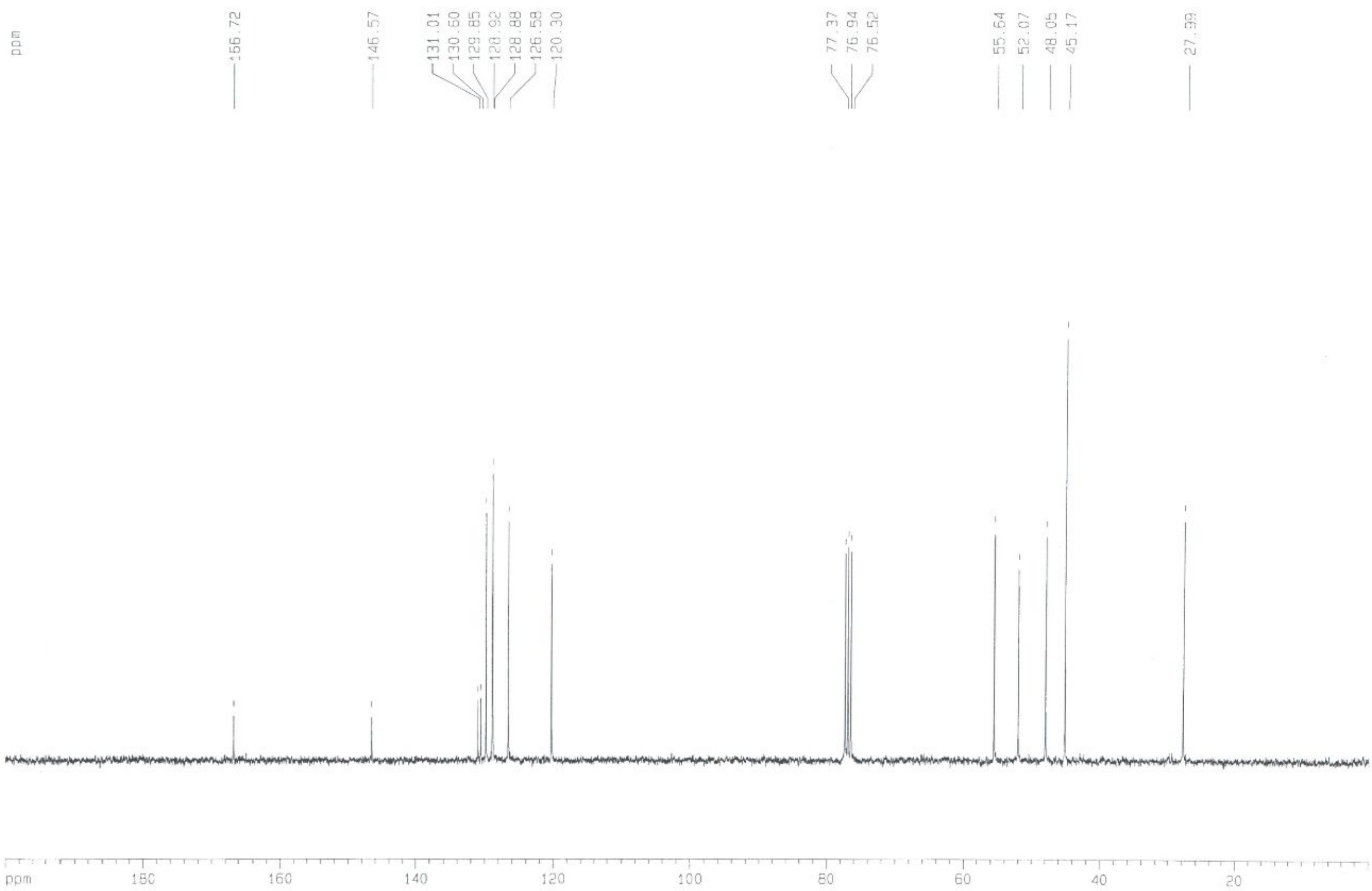

*^{1}H NMR spectrum of compound **7** recorded in DMSO-d6 at 25°C, 300 MHz.*

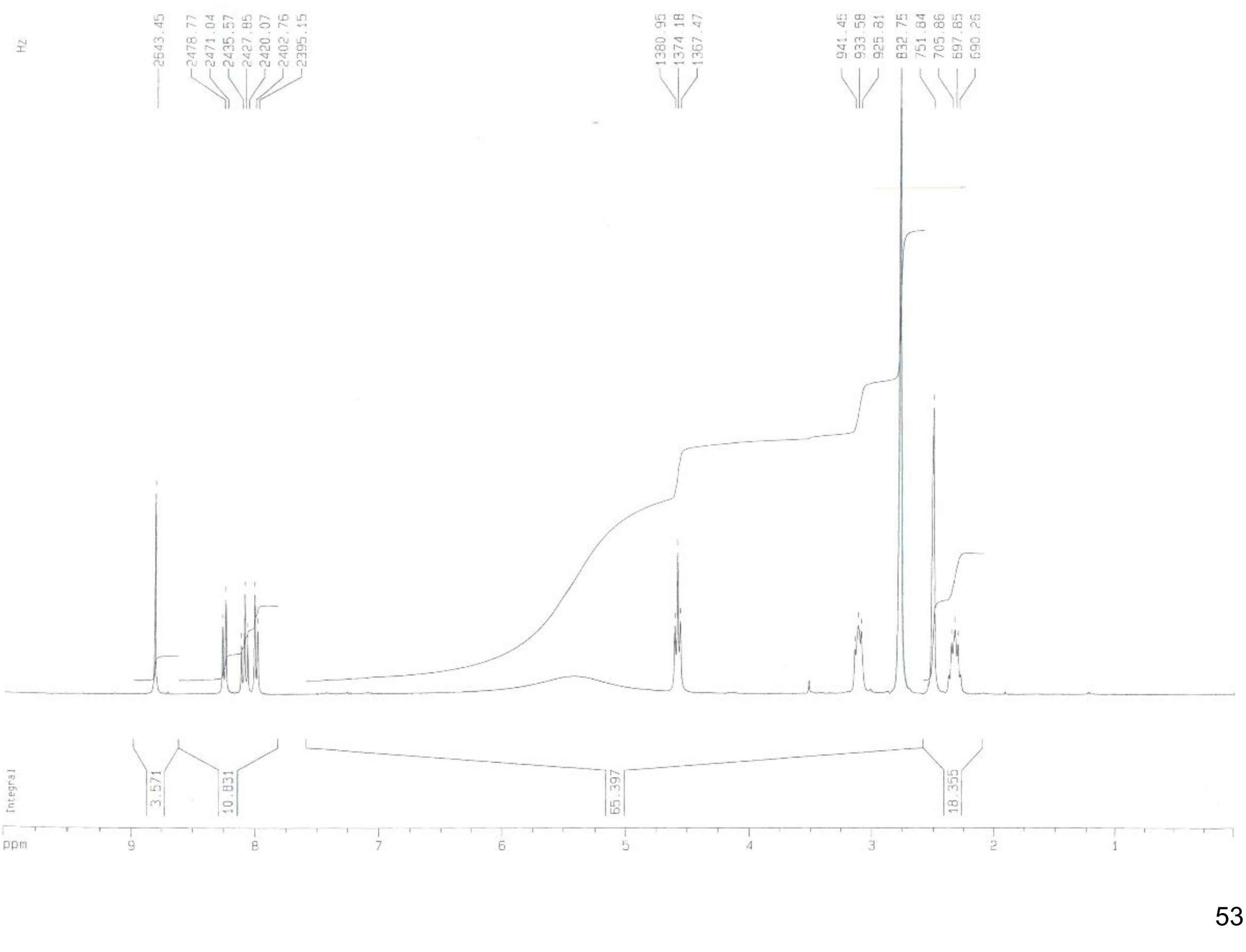

$^{13}C\{1H\}$ NMR spectrum of compound **7** recorded in DMSO-d6 at 25°C, 75 MHz.

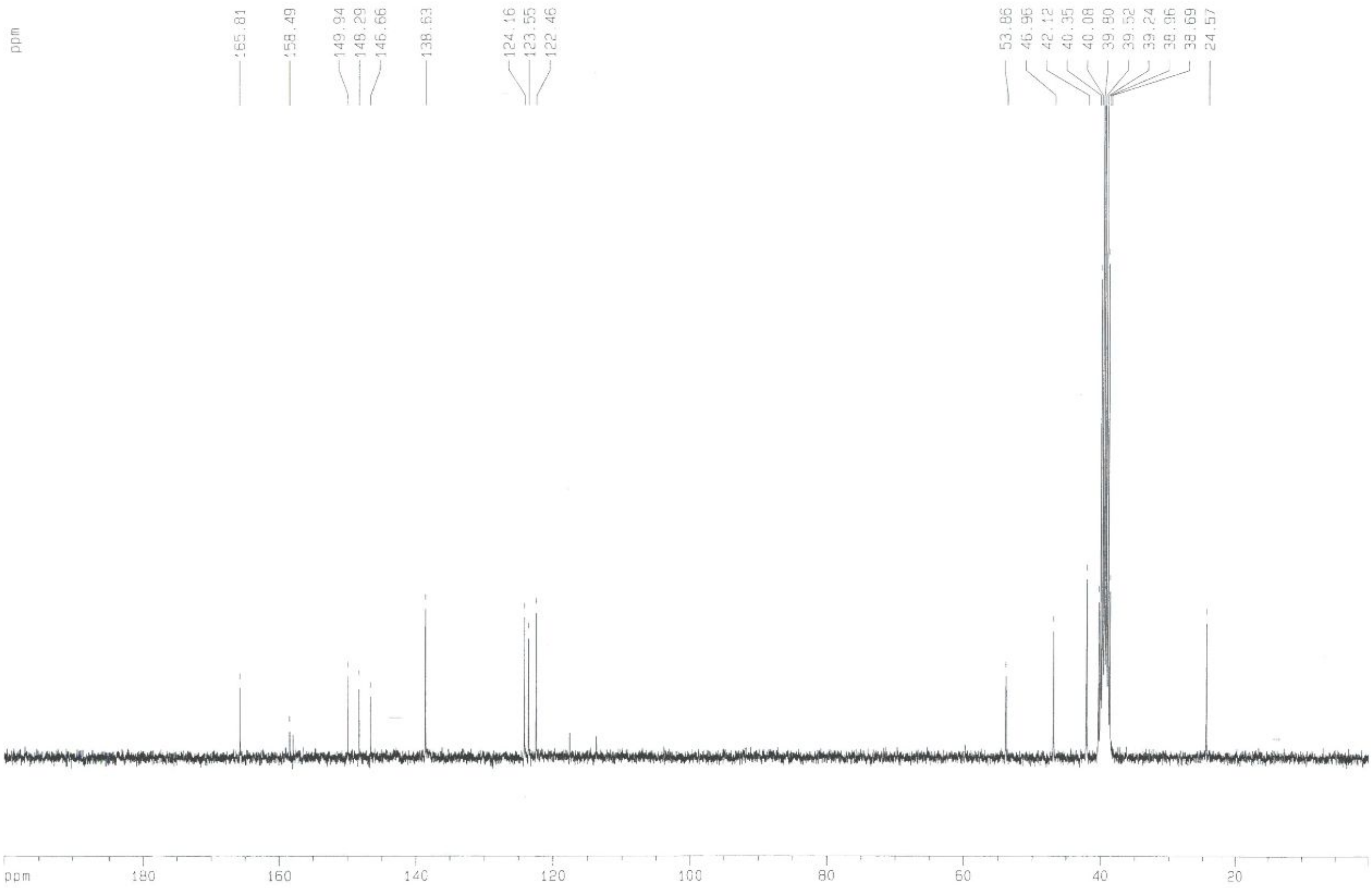

^{1}H NMR spectrum of compound **13** recorded in DMSO-d6 at 25°C, 300 MHz.

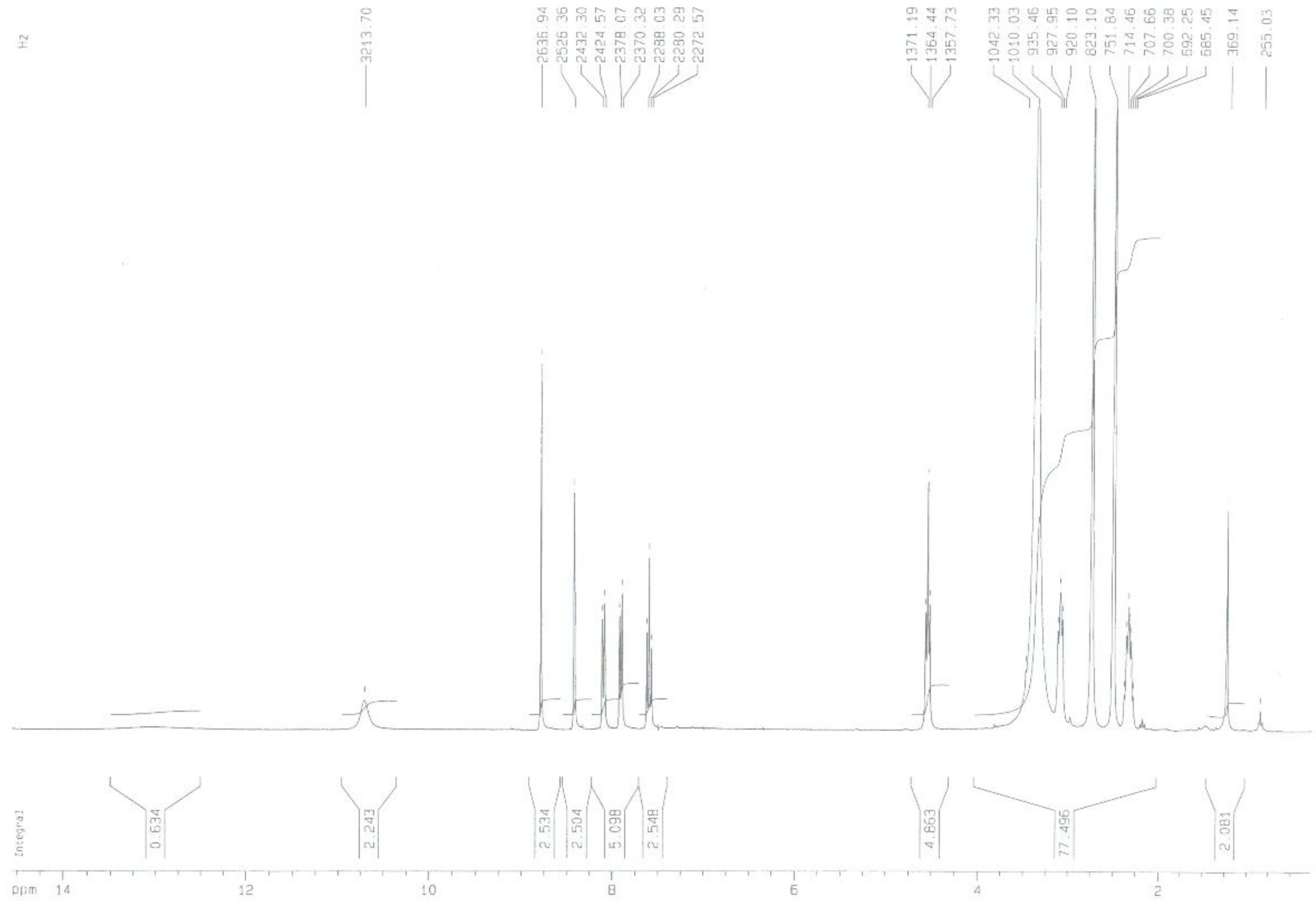

*^{13}C{1H} NMR spectrum of compound **13** recorded in DMSO-d6 at 25°C, 75 MHz.*

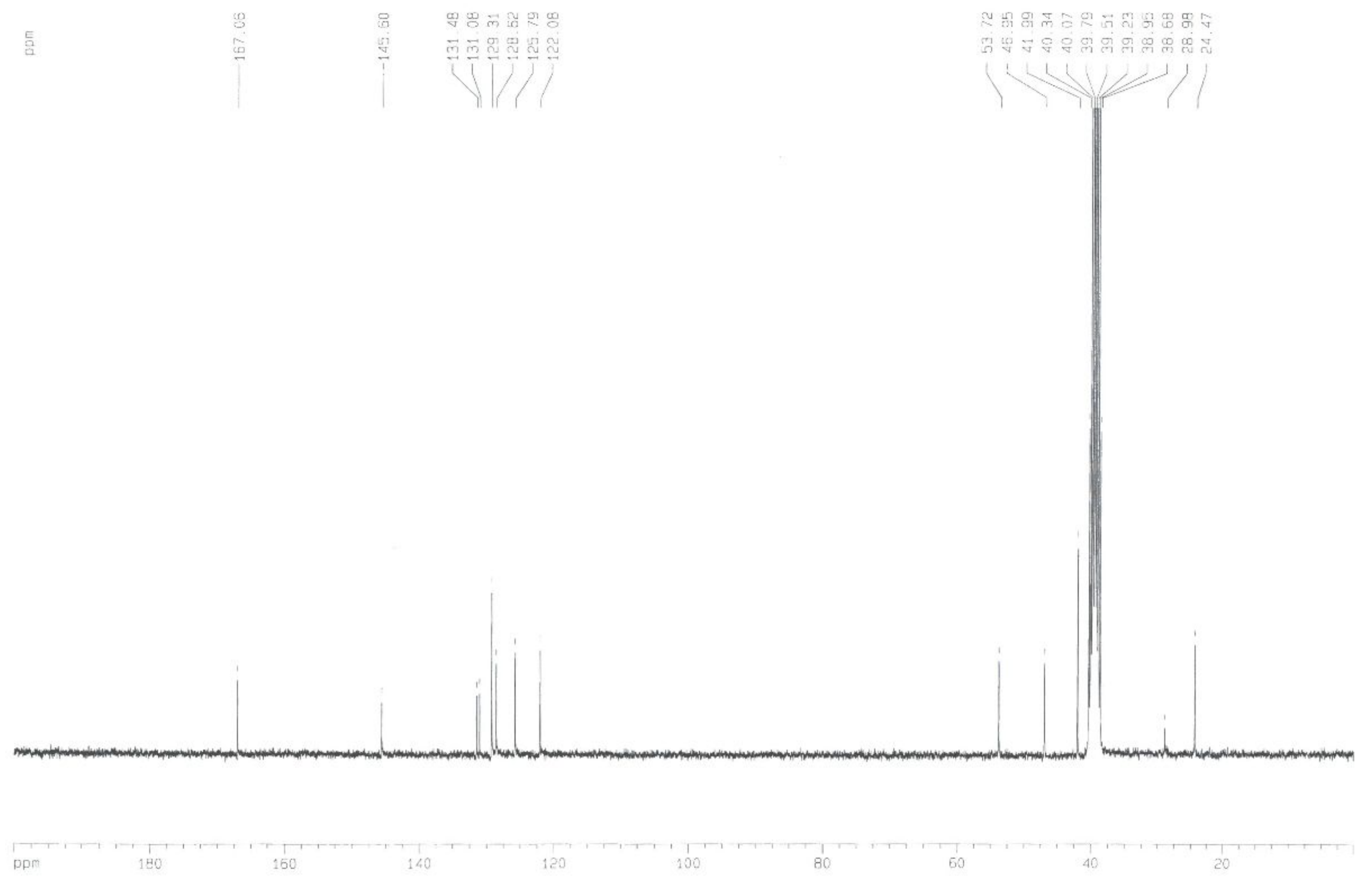

*^{1}H NMR spectrum of compound **8** recorded in $CDCl_3$ at 25°C, 300 MHz.*

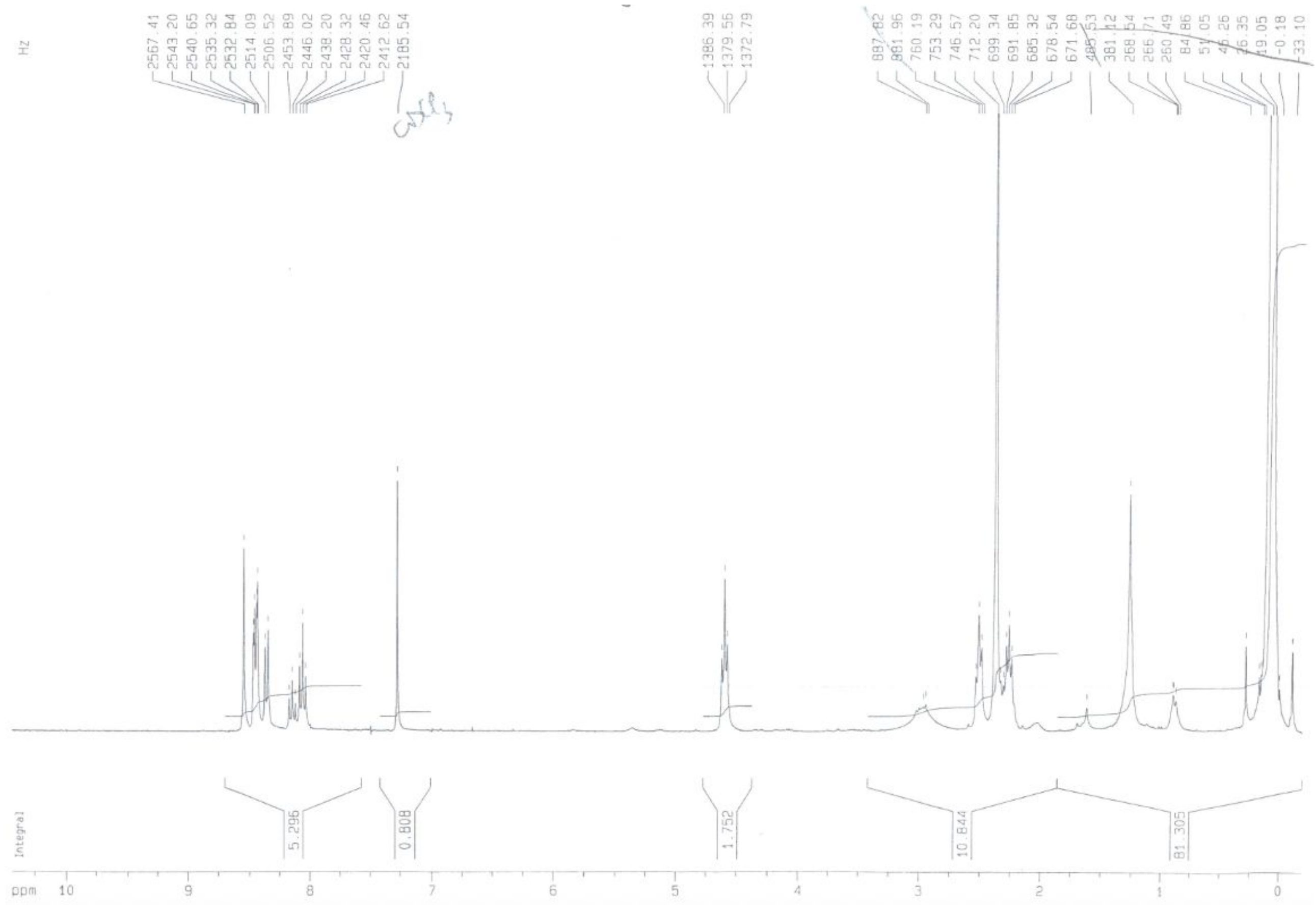

*$^{13}C\{1H\}$ NMR spectrum of compound **8** recorded in $CDCl_3$ at 25°C, 75 MHz.*

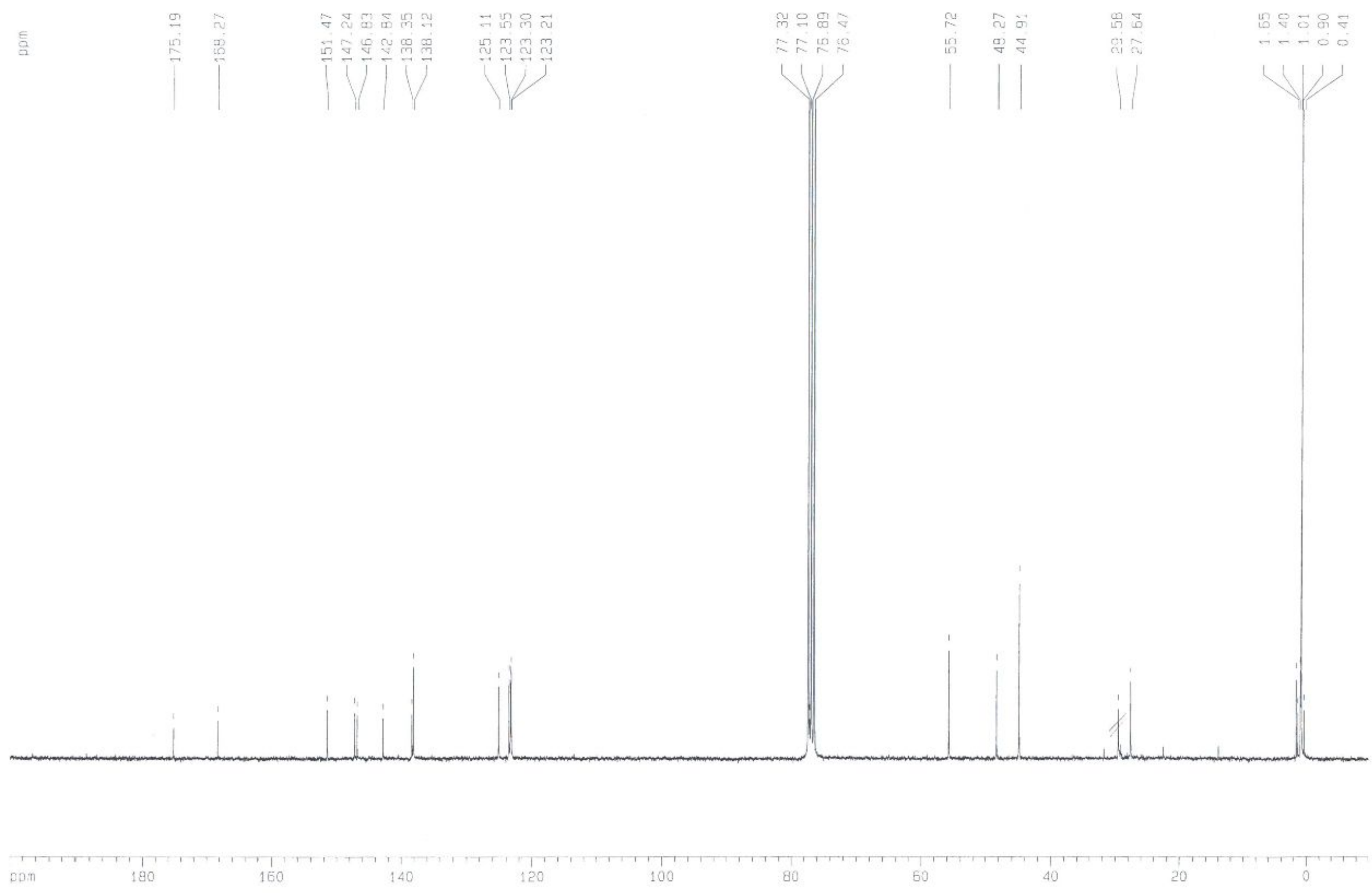

^1H NMR spectrum of compound **14** recorded in $CDCl_3$ at 25°C, 300 MHz.

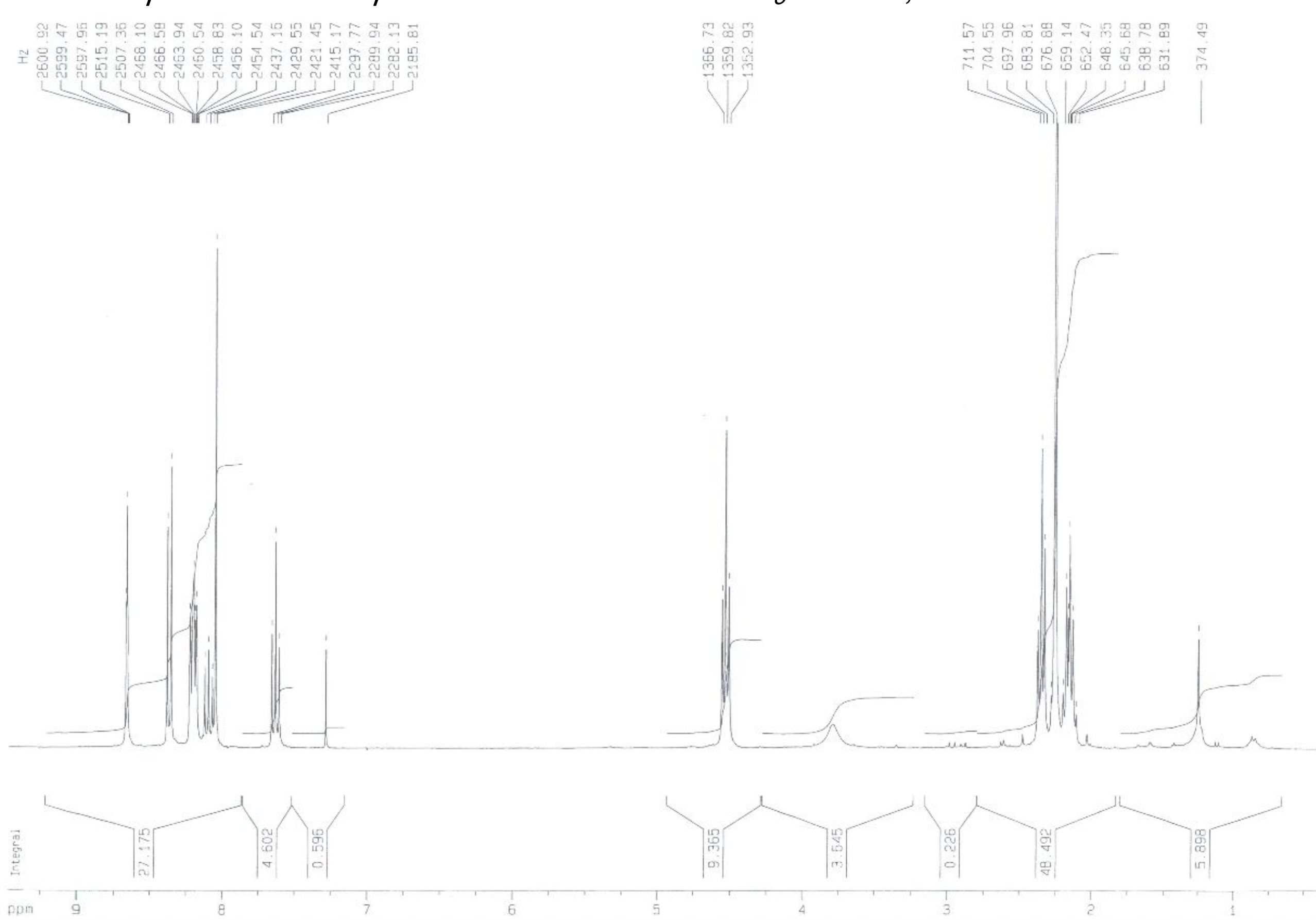

$^{13}C\{1H\}$ NMR spectrum of compound **14** recorded in $CDCl_3$ at 25°C, 75 MHz.

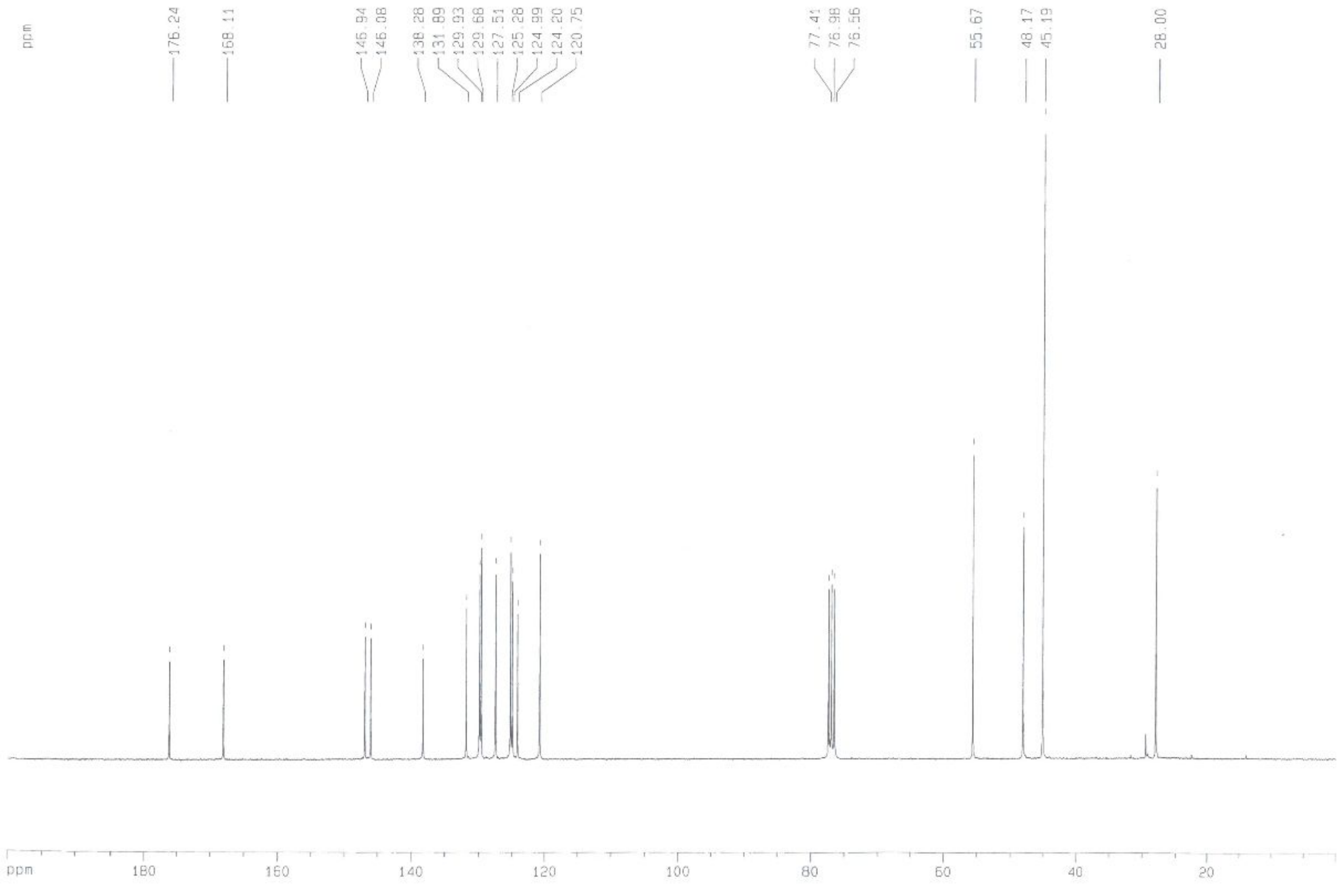

^{1}H NMR spectrum of compound **15** recorded in $CDCl_3$ at 25°C, 300 MHz.

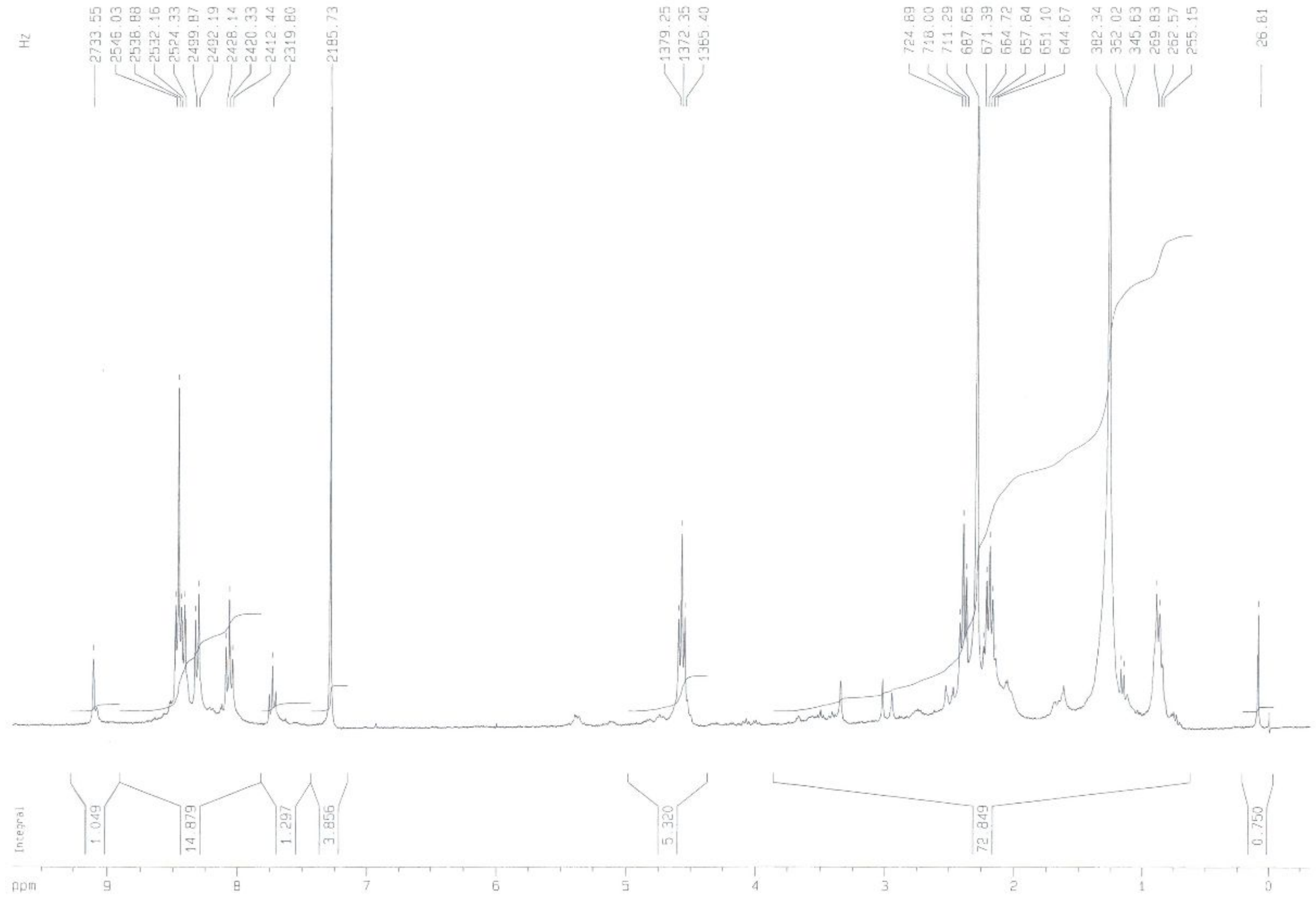

$^{13}C\{1H\}$ *NMR spectrum of compound **15** recorded in* $CDCl_3$ *at 25°C, 75 MHz.*

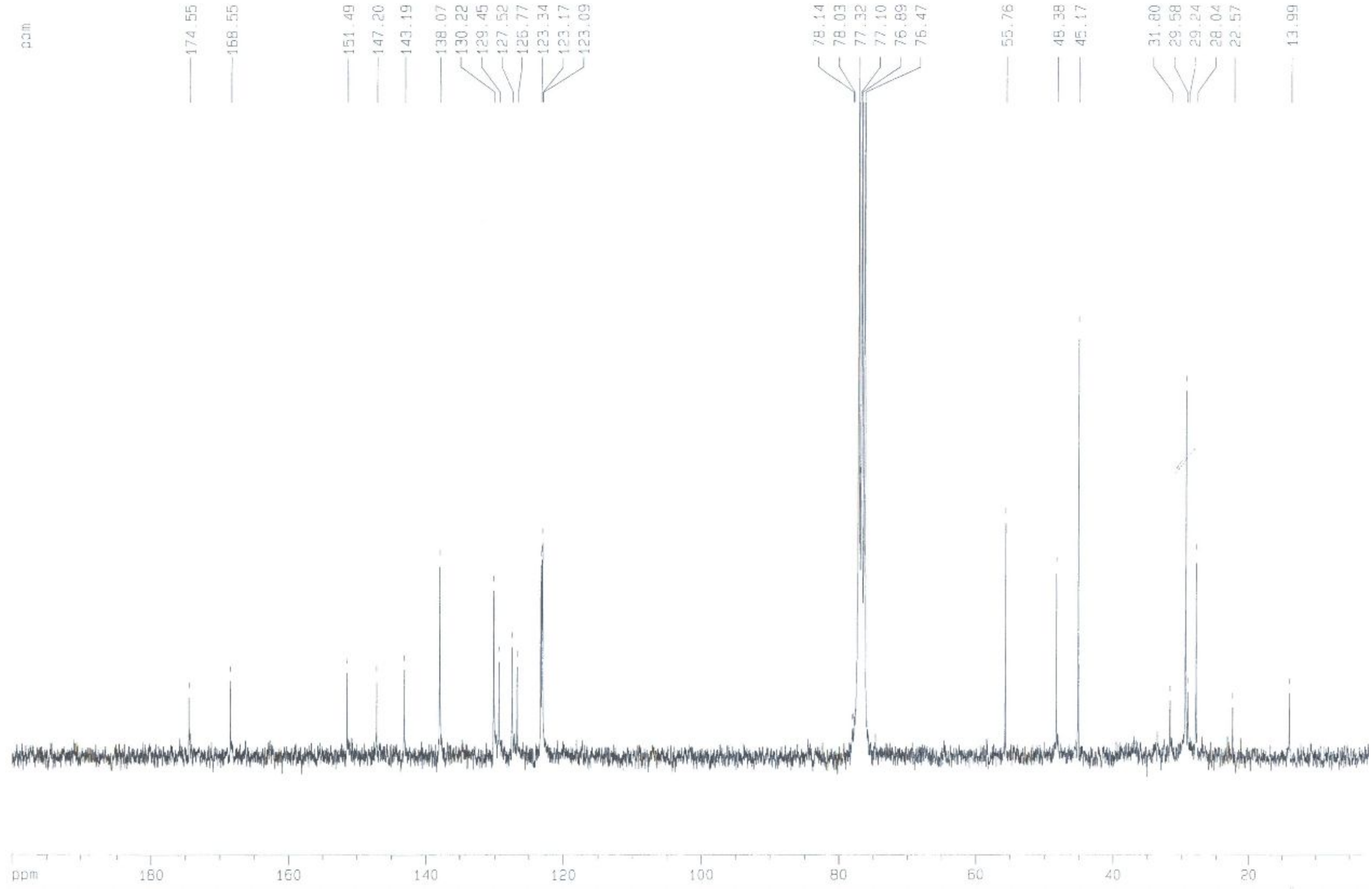

^{1}H NMR spectrum of compound **16** recorded in $CDCl_3$ at 25°C, 300 MHz.

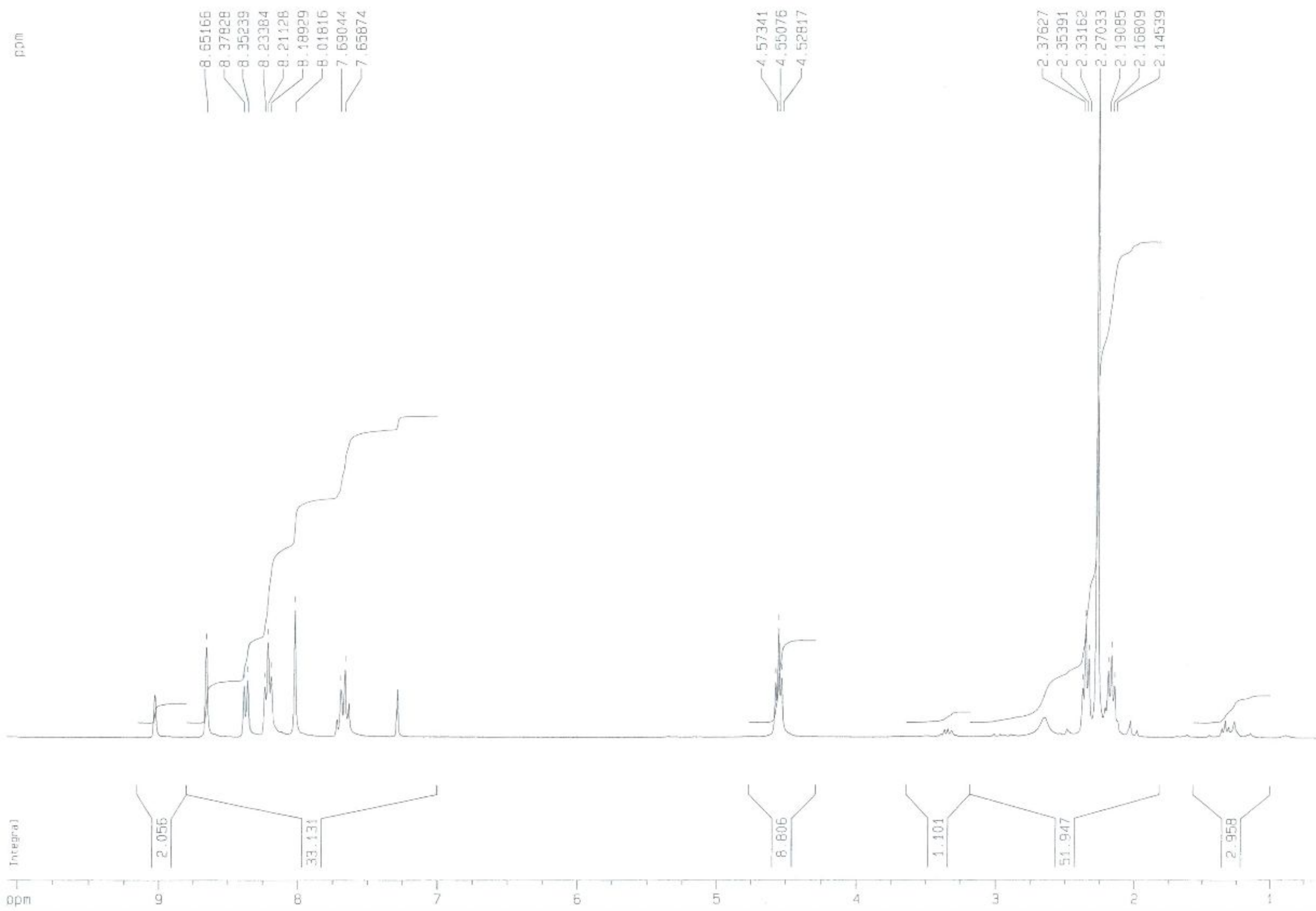

$^{13}C\{1H\}$ NMR spectrum of compound **16** recorded in $CDCl_3$ at 25°C, 75 MHz.

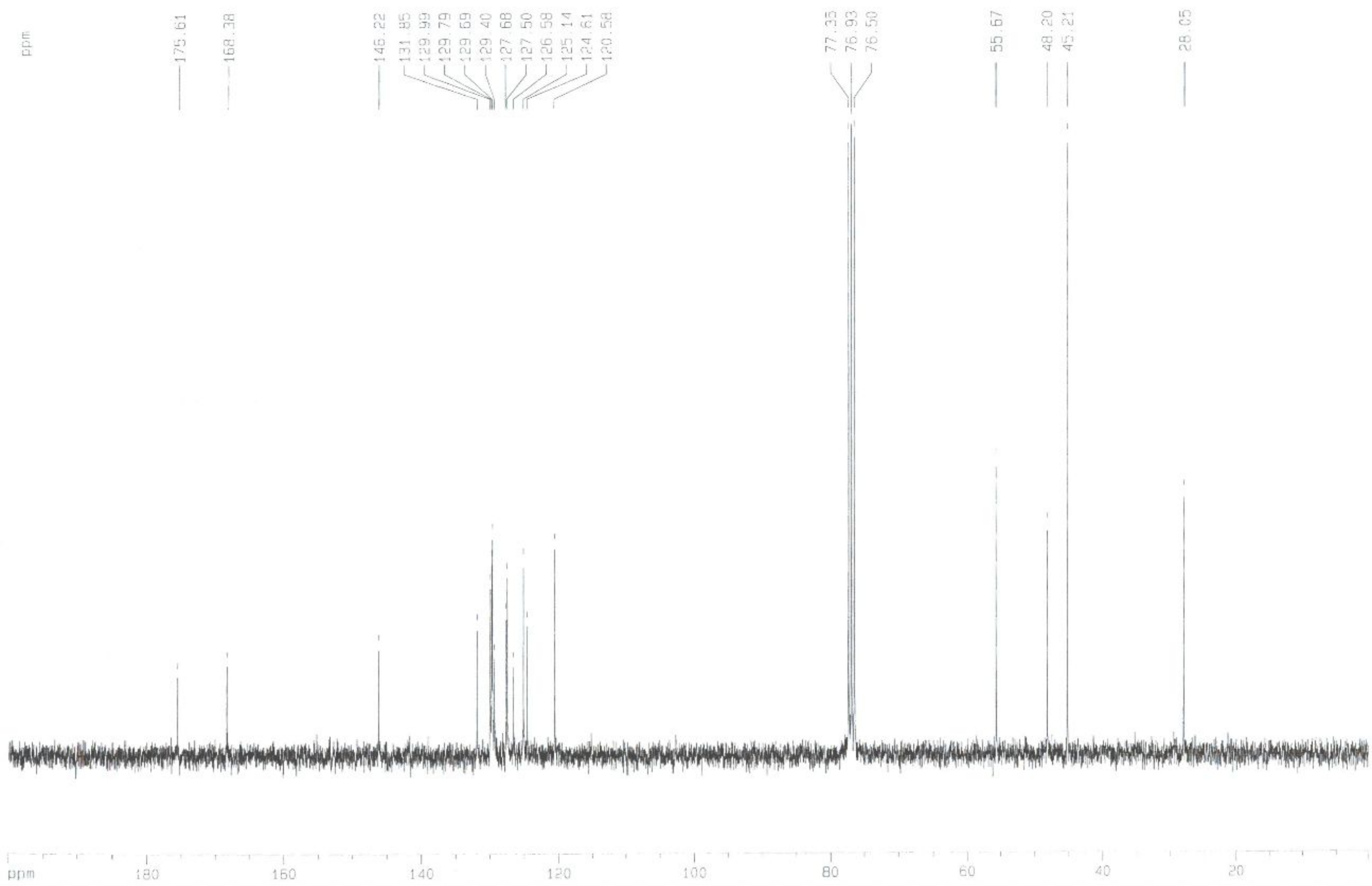

*^{1}H NMR spectrum of compound **21** recorded in $CDCl_3$ at 25°C, 300 MHz.*

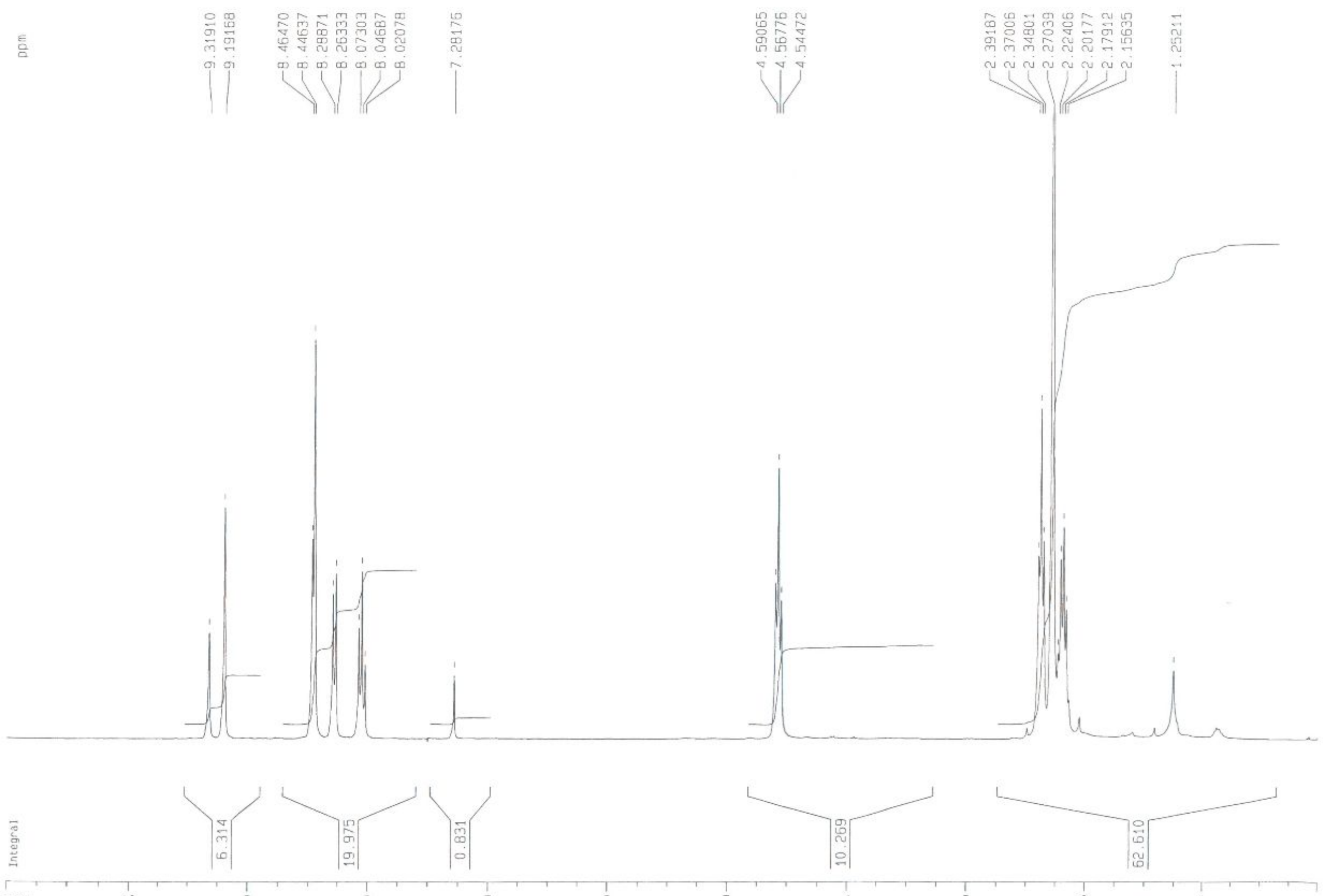

$^{13}C\{^1H\}$ *NMR spectrum of compound* ***21*** *recorded in* $CDCl_3$ *at 25°C, 75 MHz.*

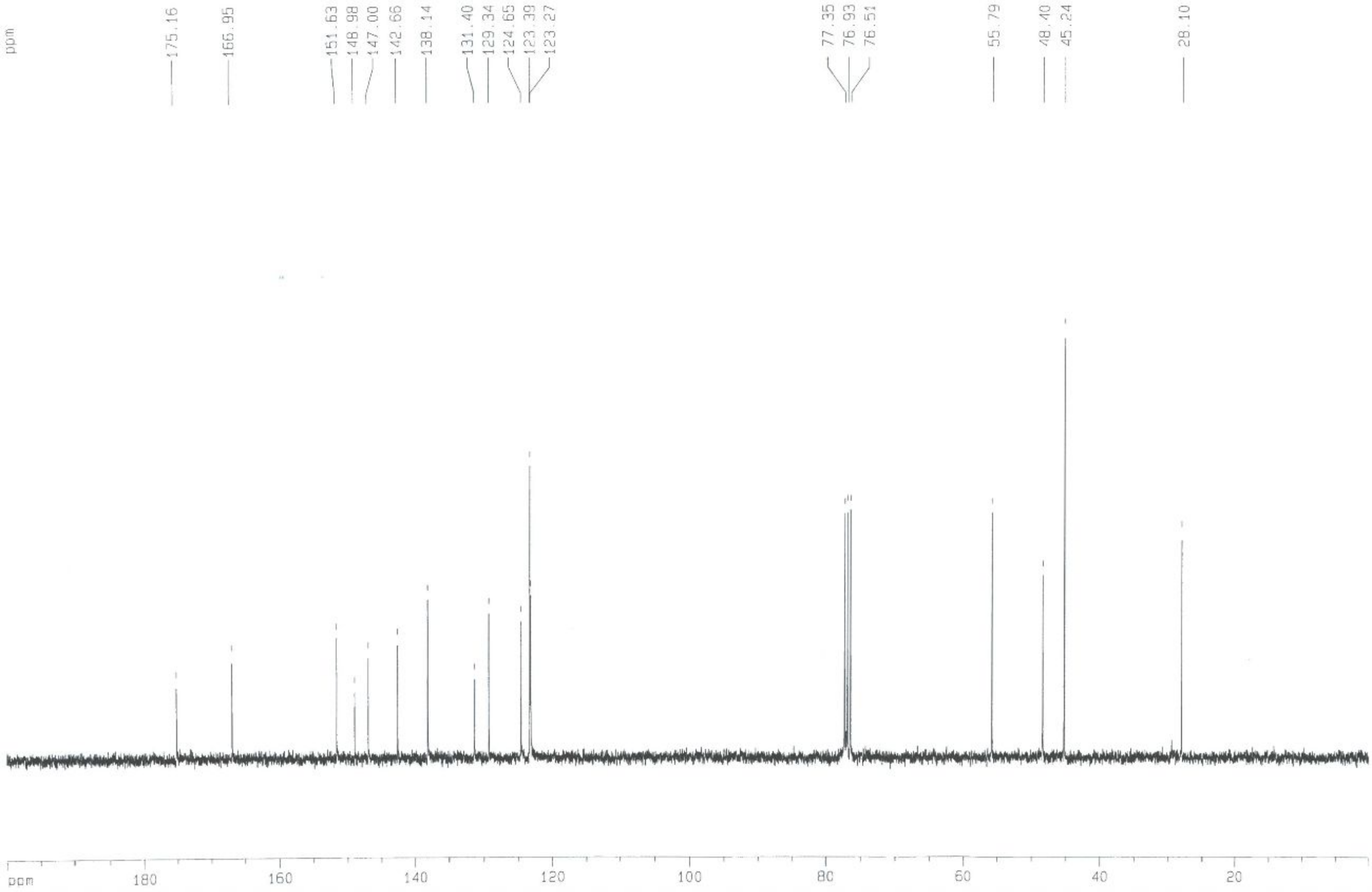

^1H *NMR spectrum of compound* ***22*** *recorded in* $CDCl_3$ *at 25°C, 300 MHz.*

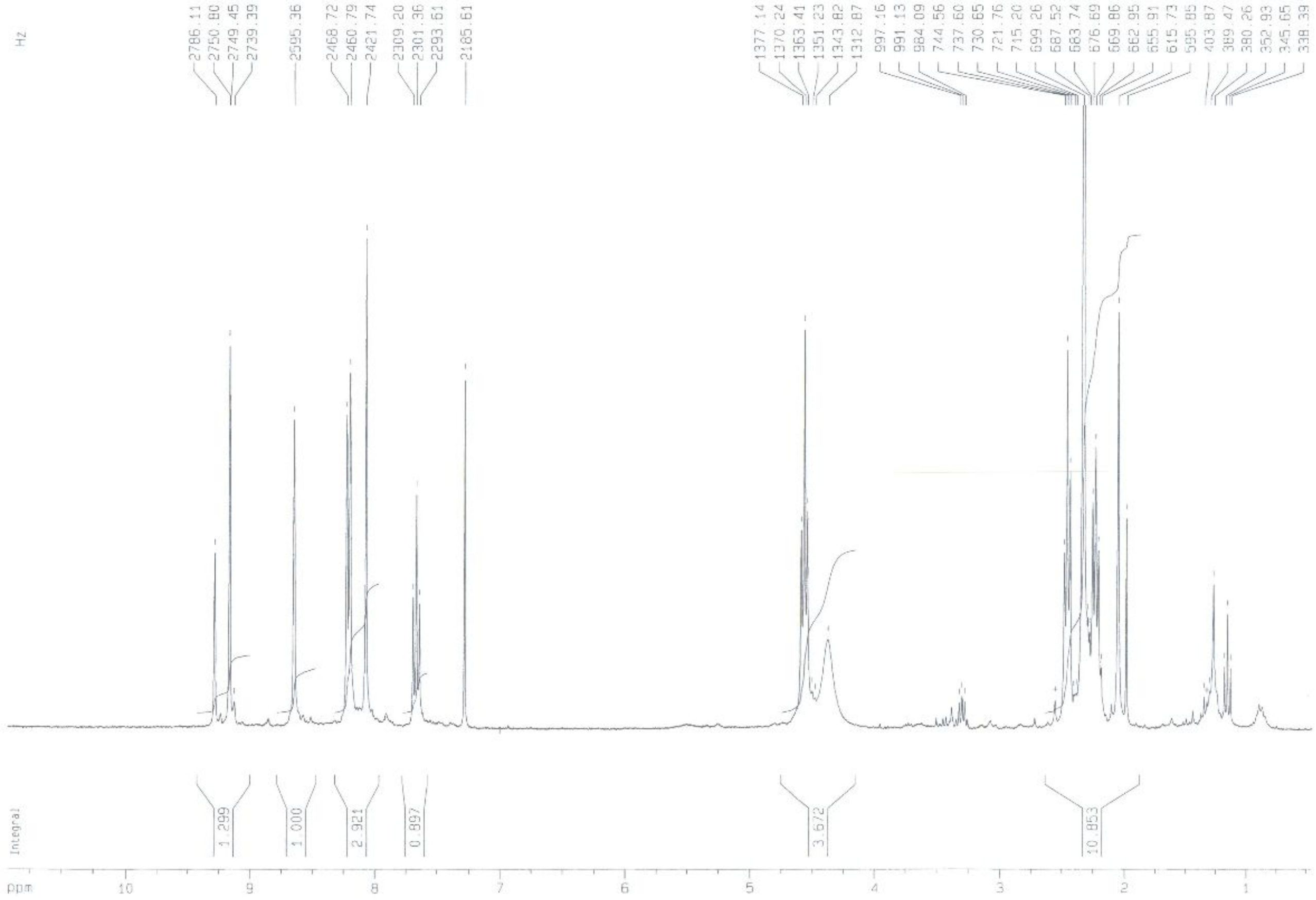

$^{13}C\{1H\}$ NMR spectrum of compound **22** recorded in $CDCl_3$ at 25°C, 75 MHz.

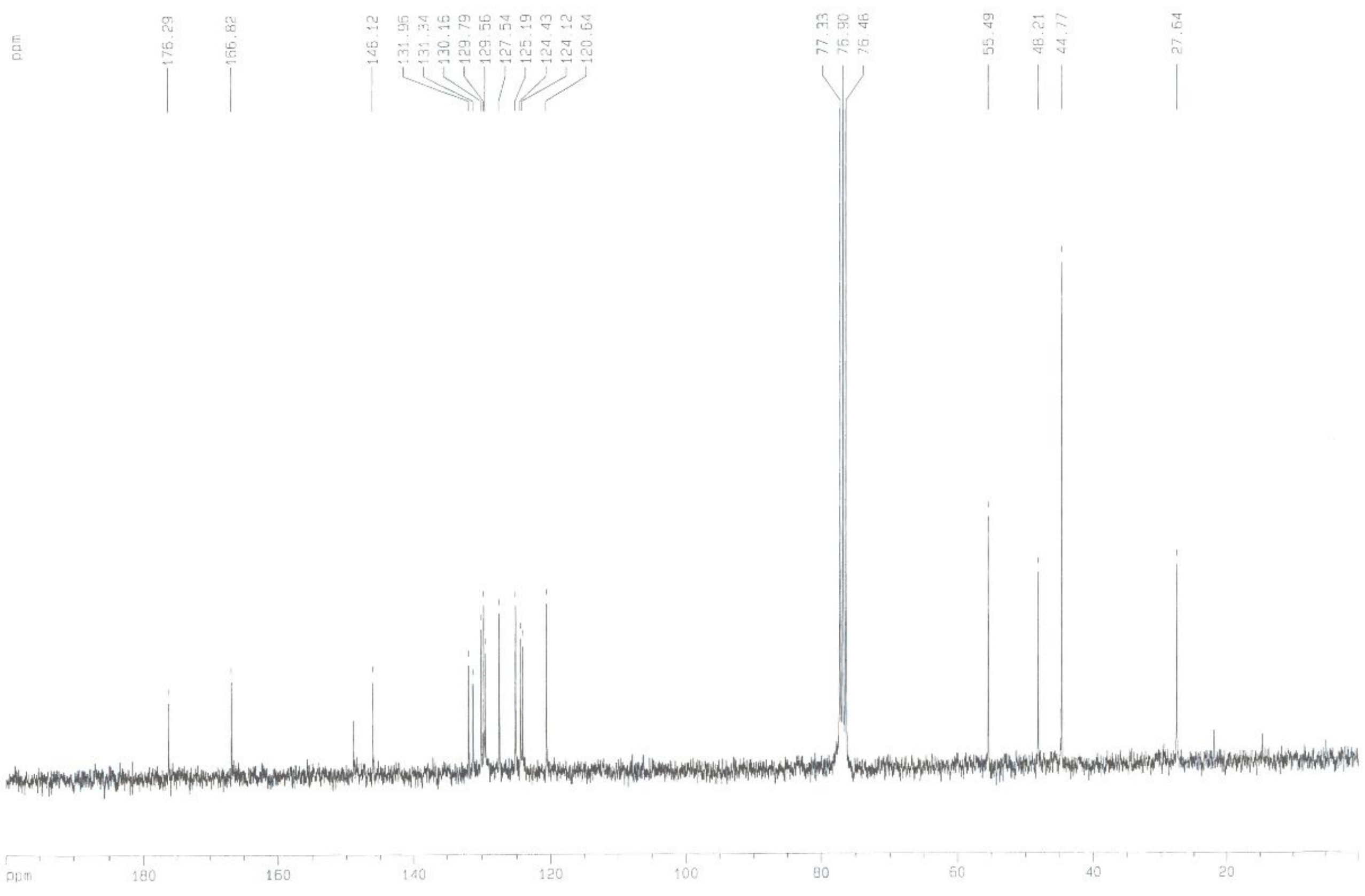

*^{1}H NMR spectrum of compound **23** recorded in $CDCl_3$ at 25°C, 300 MHz.*

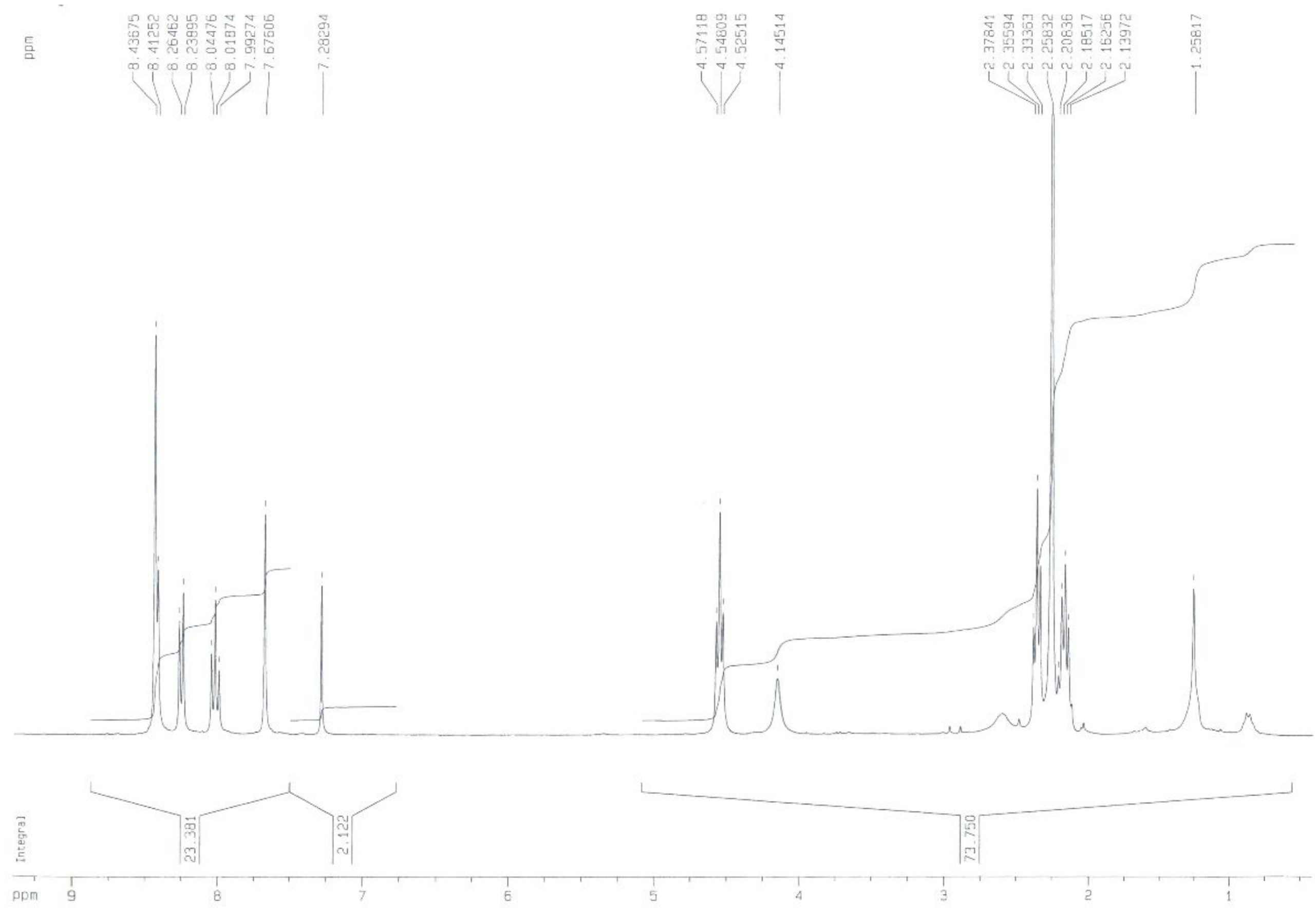

*$^{13}C\{1H\}$ NMR spectrum of compound **23** recorded in $CDCl_3$ at 25°C, 75 MHz.*

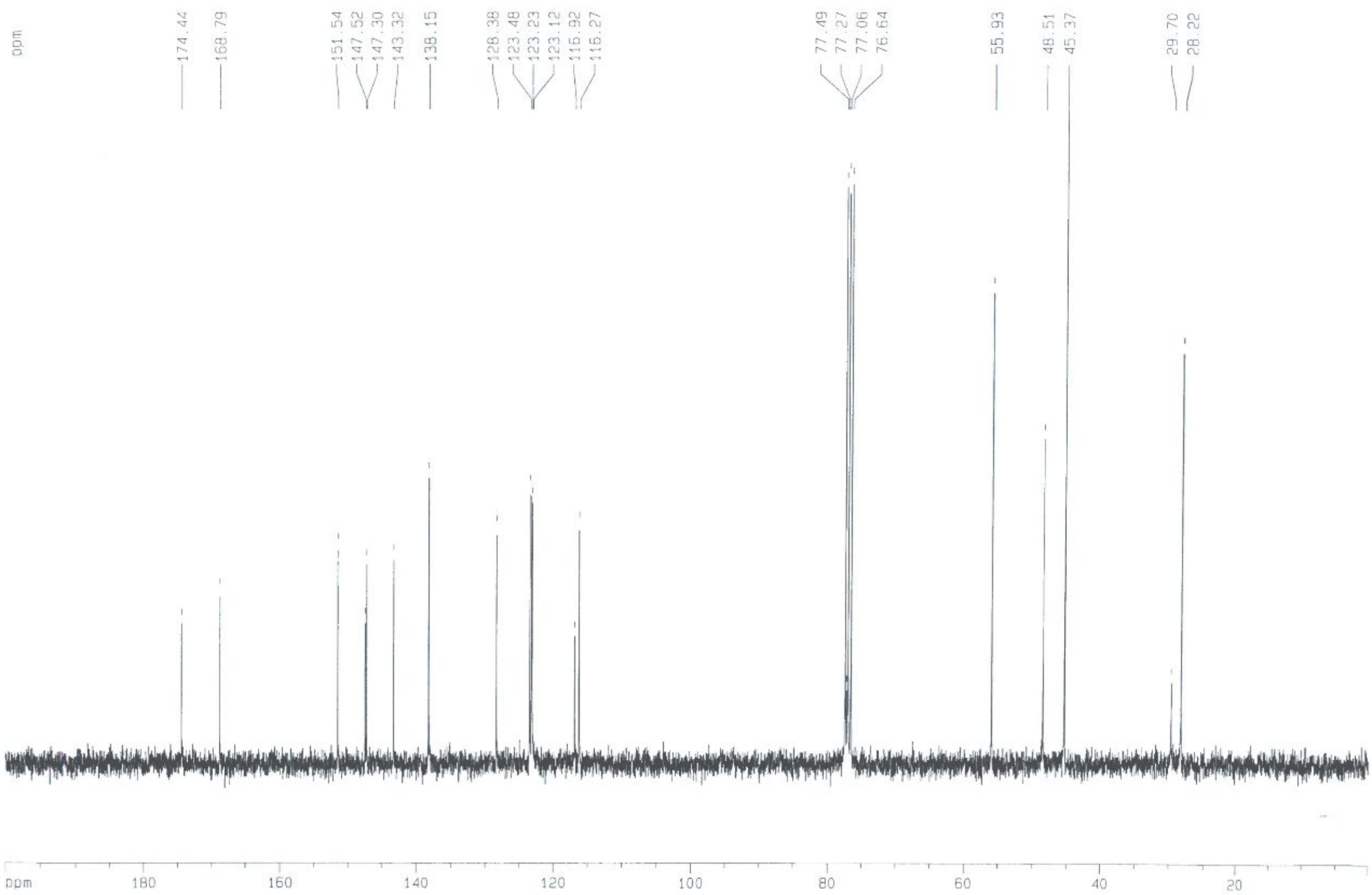

*^{1}H NMR spectrum of compound **24** recorded in $CDCl_3$ at 25°C, 300 MHz.*

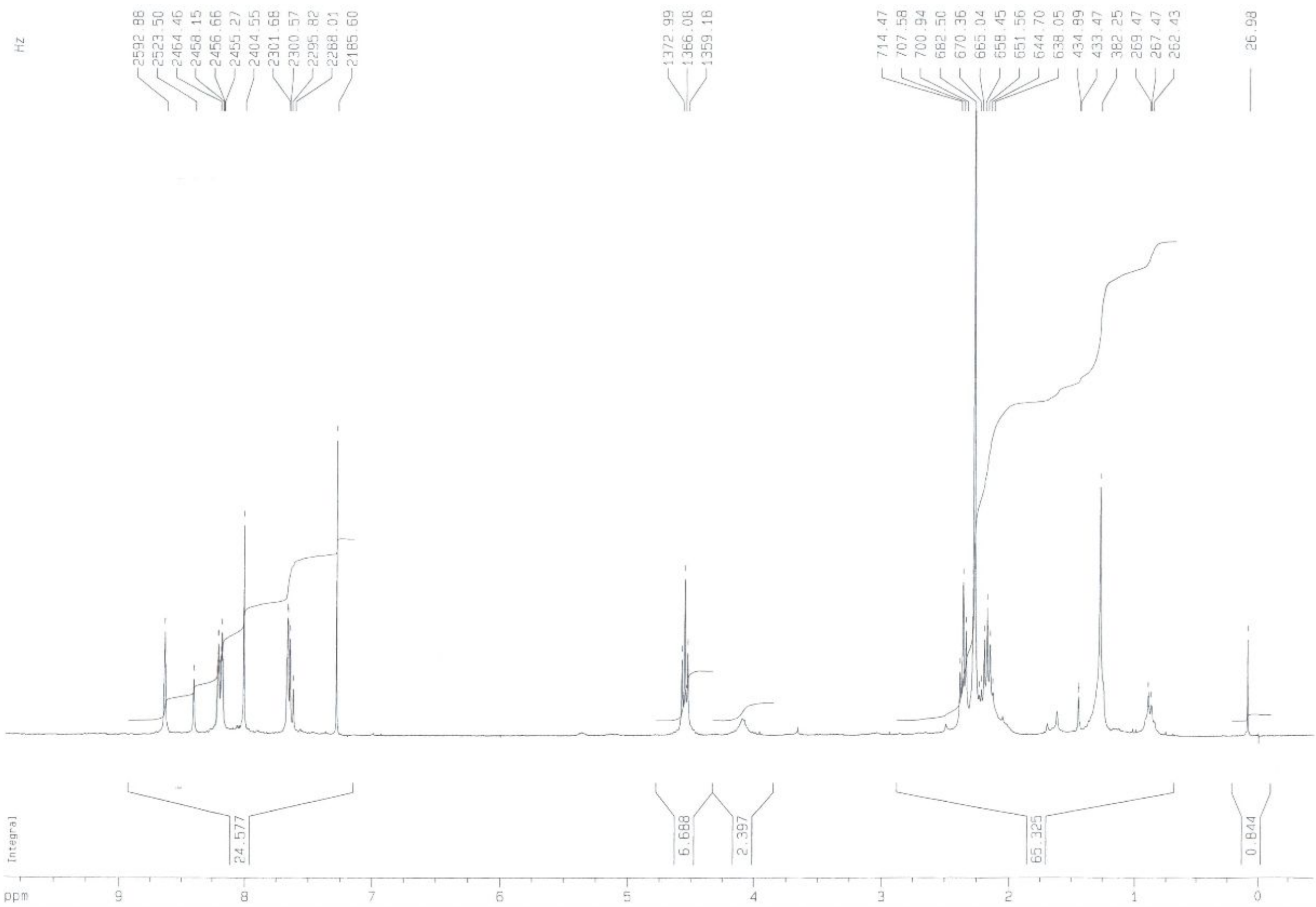

*$^{13}C\{^1H\}$ NMR spectrum of compound **24** recorded in $CDCl_3$ at 25°C, 75 MHz.*

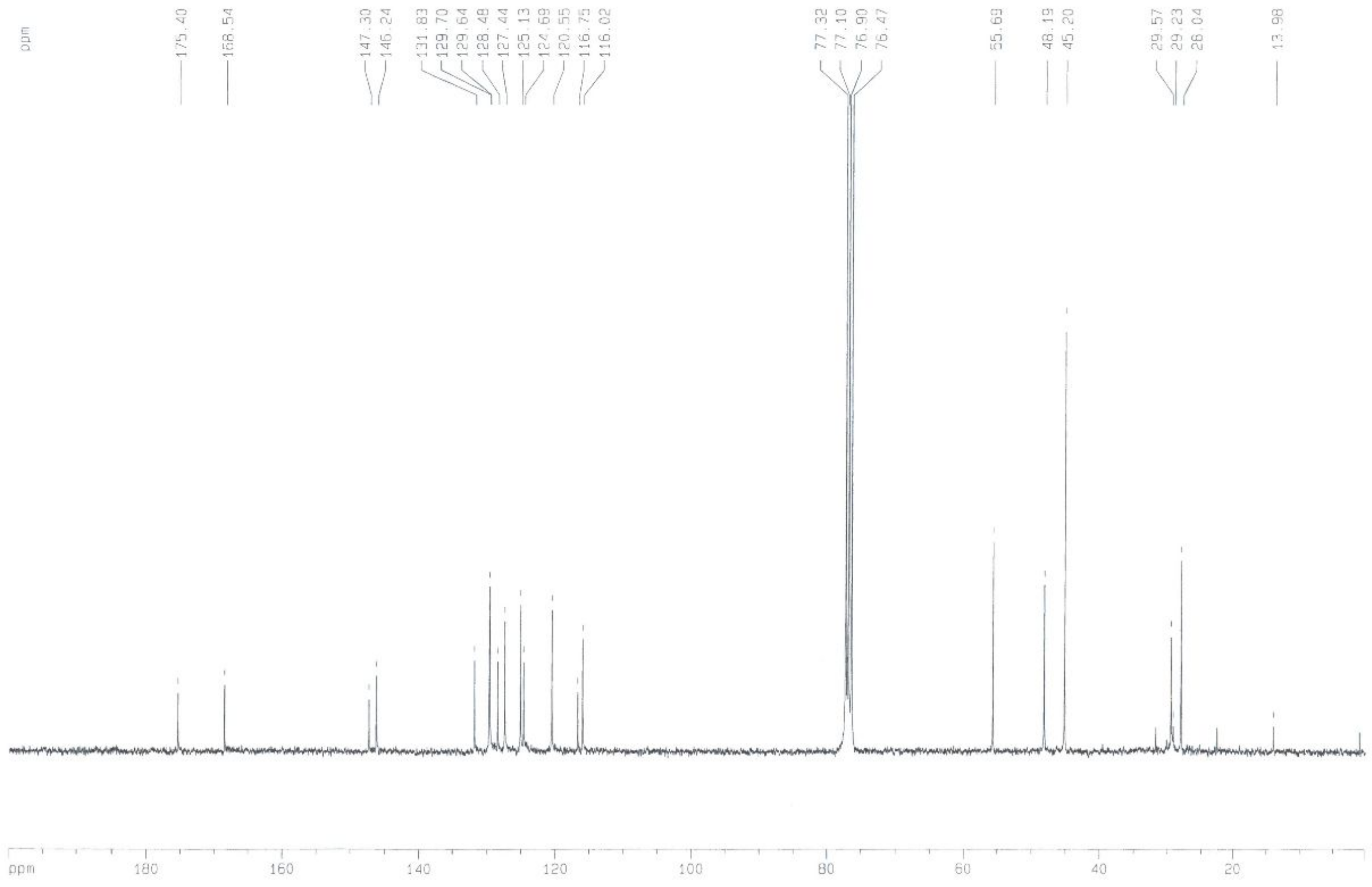

MS spectra for the compounds 8, 14-16, 21-24

*Electrospray ionization mass spectrum (ESI-MS) of compound **8** recorded in positive ion mode.*

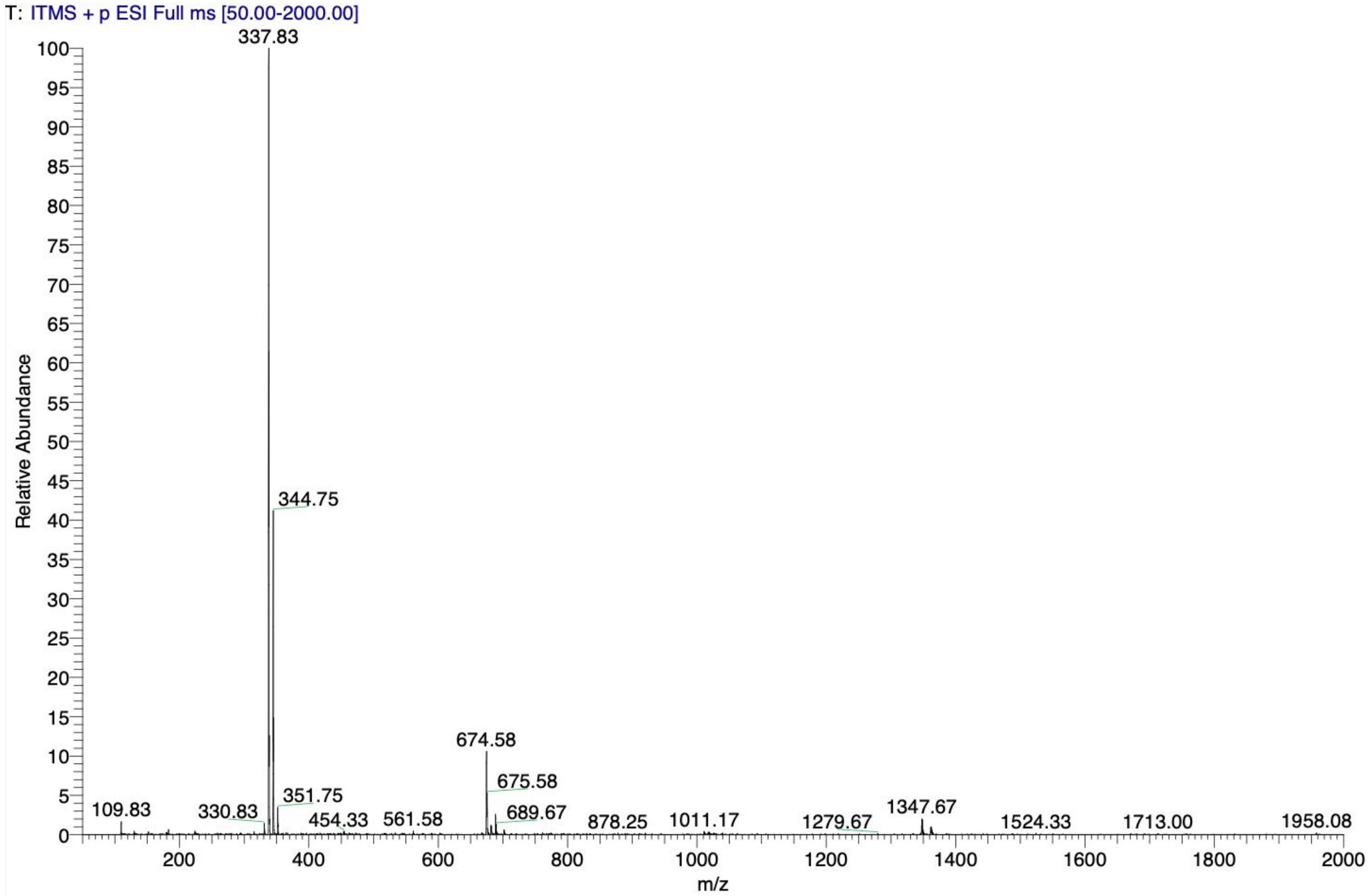

*Electrospray ionization mass spectrum (ESI-MS) of compound **14** recorded in positive ion mode.*

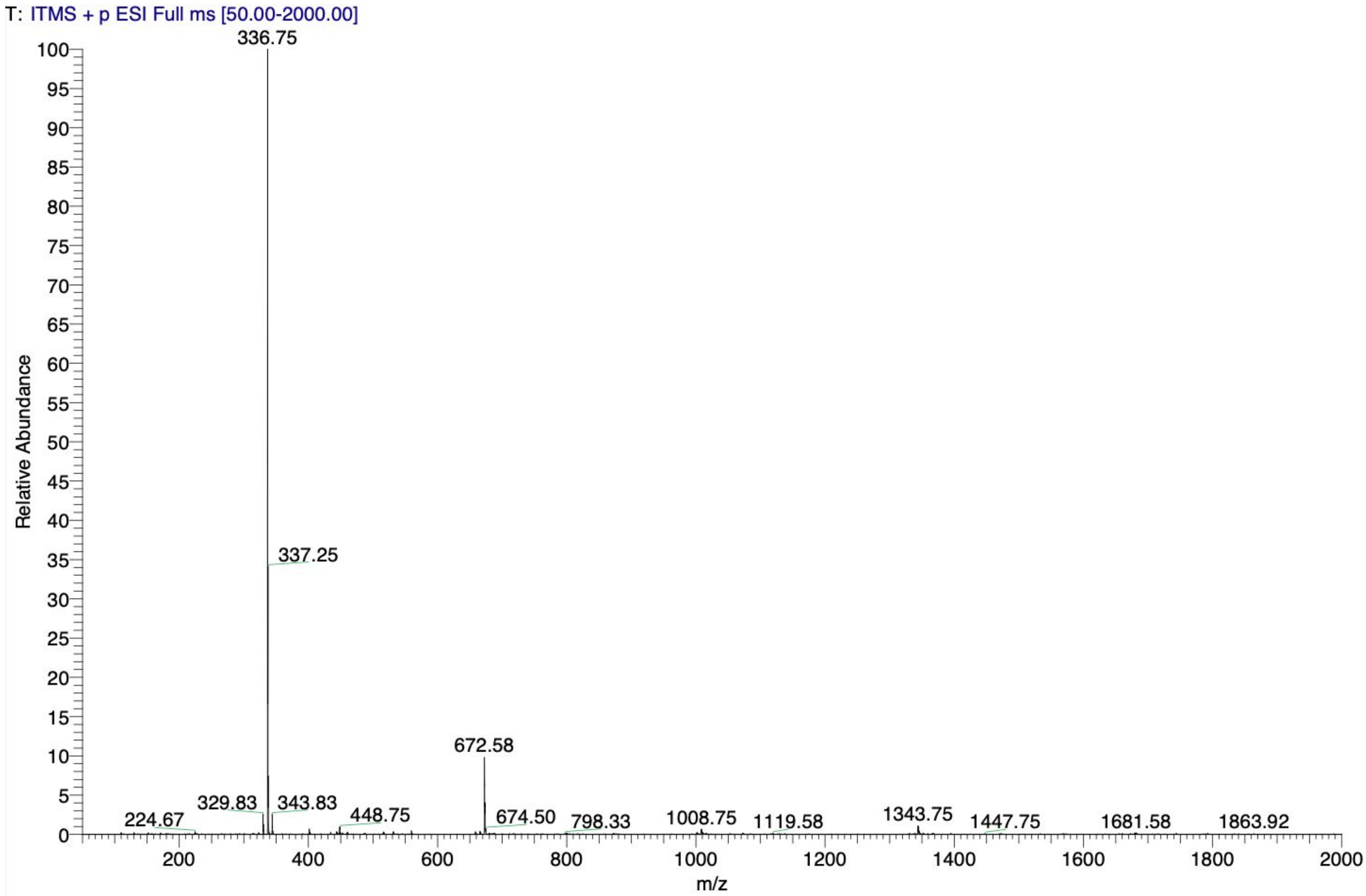

*Electrospray ionization mass spectrum (ESI-MS) of compound **15** recorded in positive ion mode.*

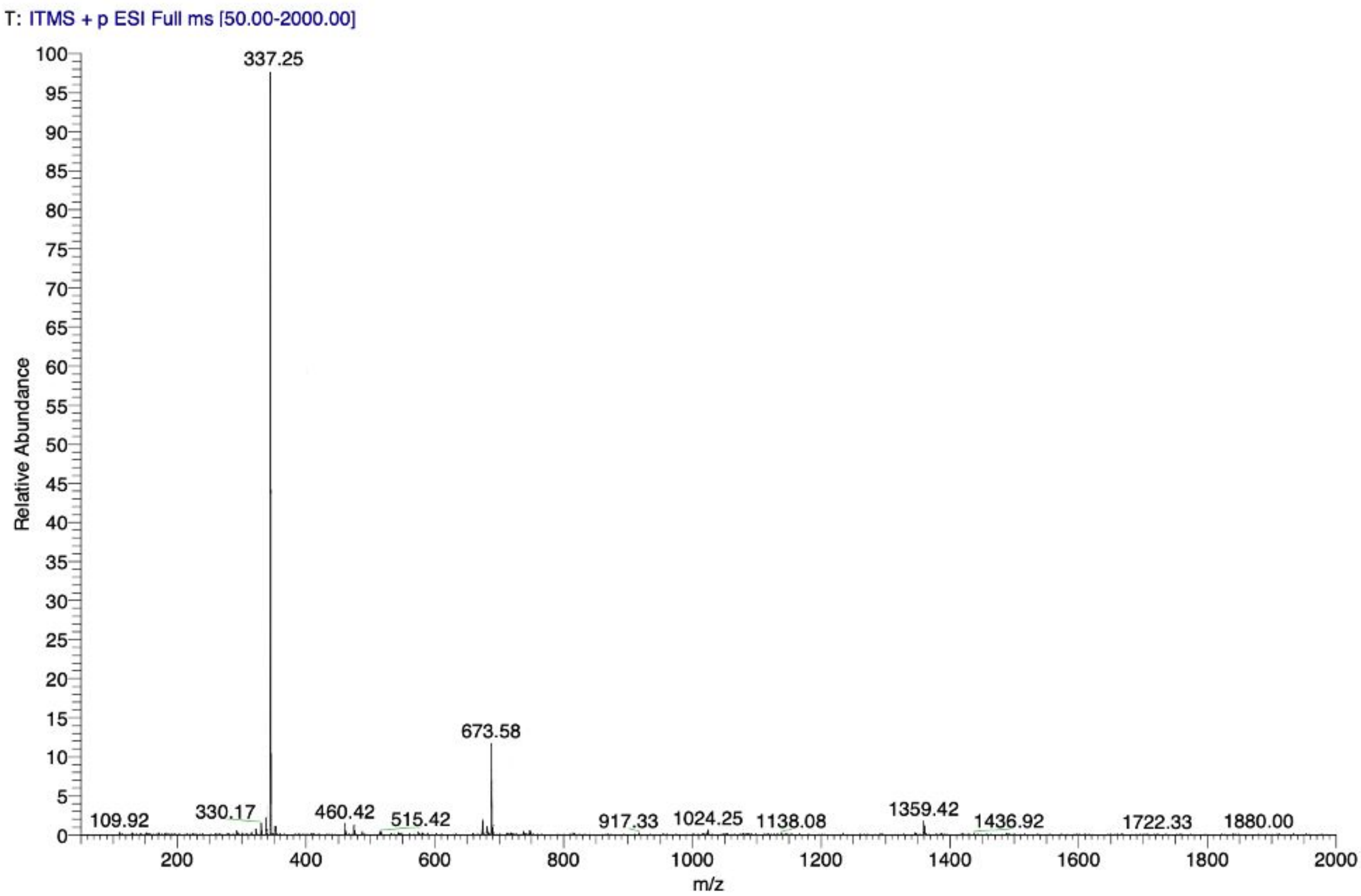

*Electrospray ionization mass spectrum (ESI-MS) of compound **16** recorded in positive ion mode.*

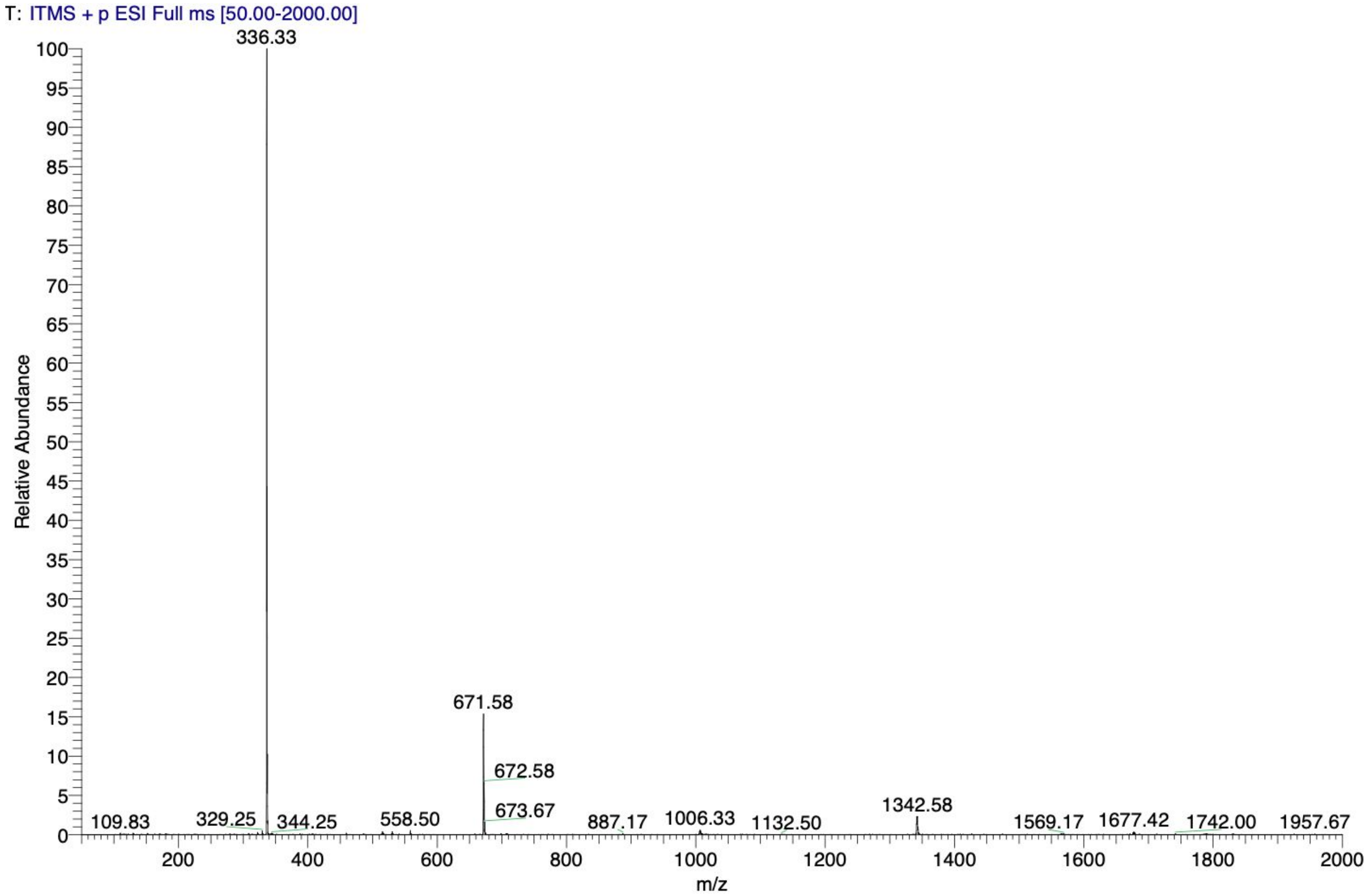

*Electrospray ionization mass spectrum (ESI-MS) of compound **21** recorded in positive ion mode.*

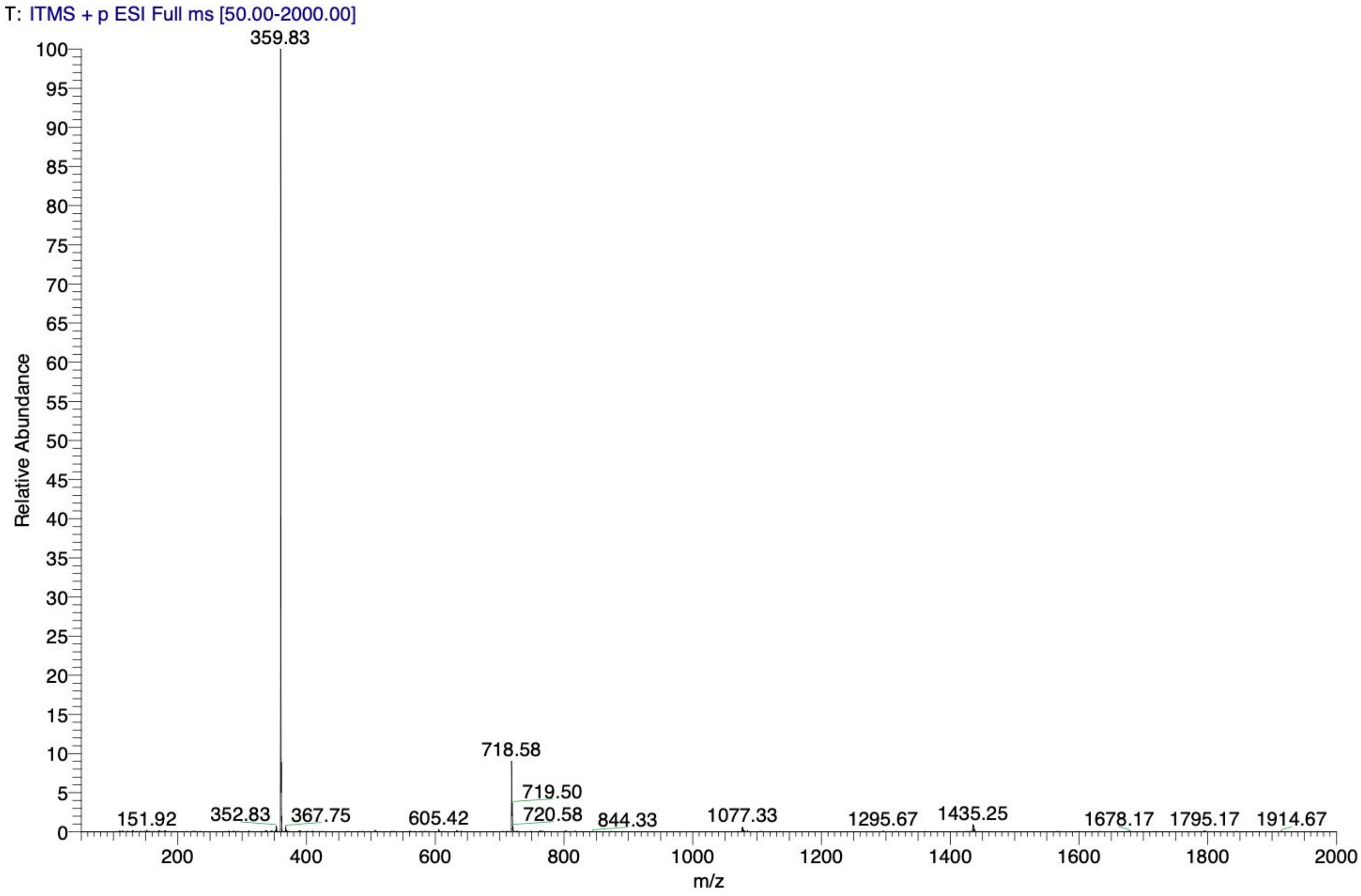

*Electrospray ionization mass spectrum (ESI-MS) of compound **22** recorded in positive ion mode.*

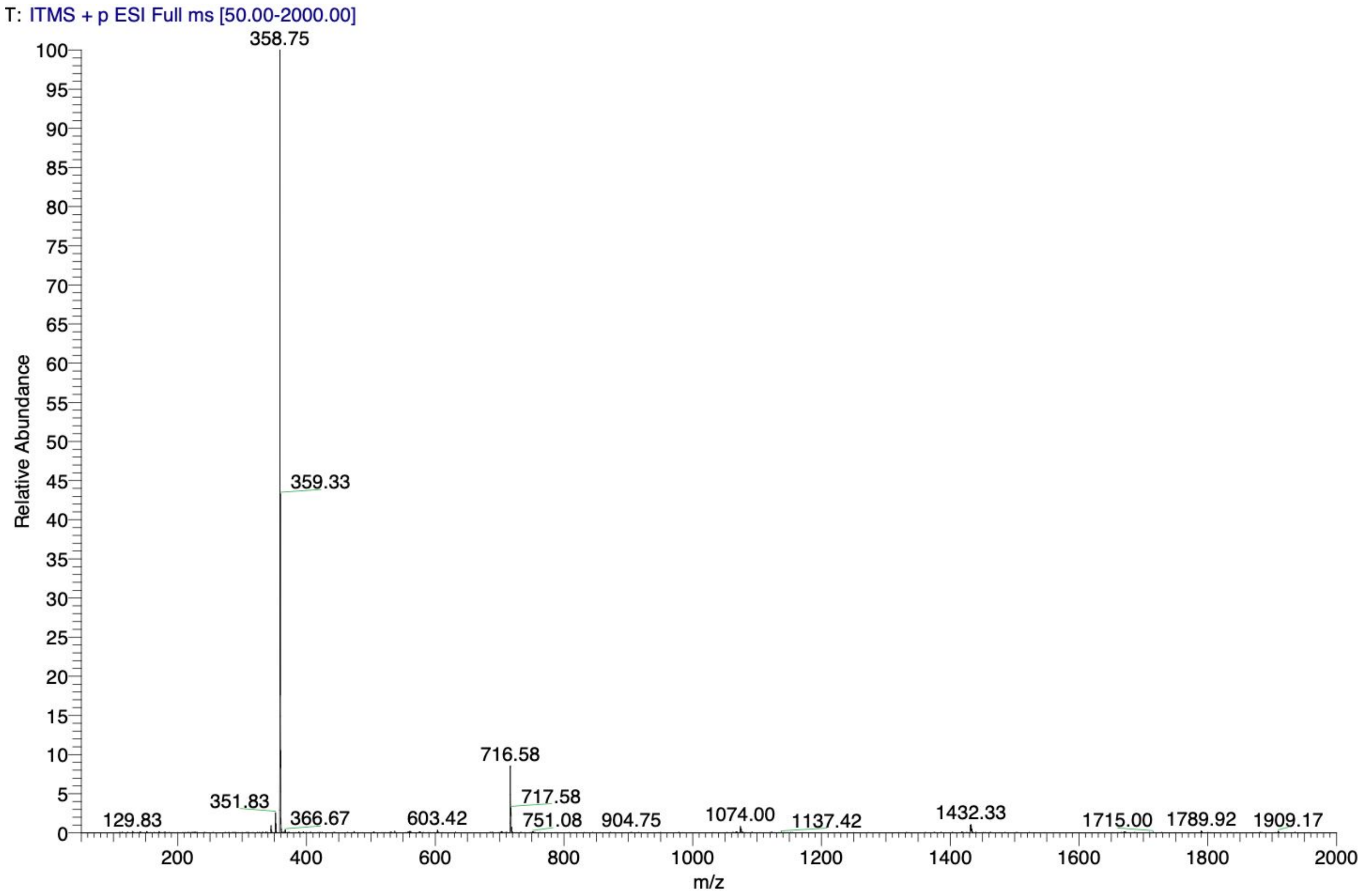

*Electrospray ionization mass spectrum (ESI-MS) of compound **23** recorded in positive ion mode.*

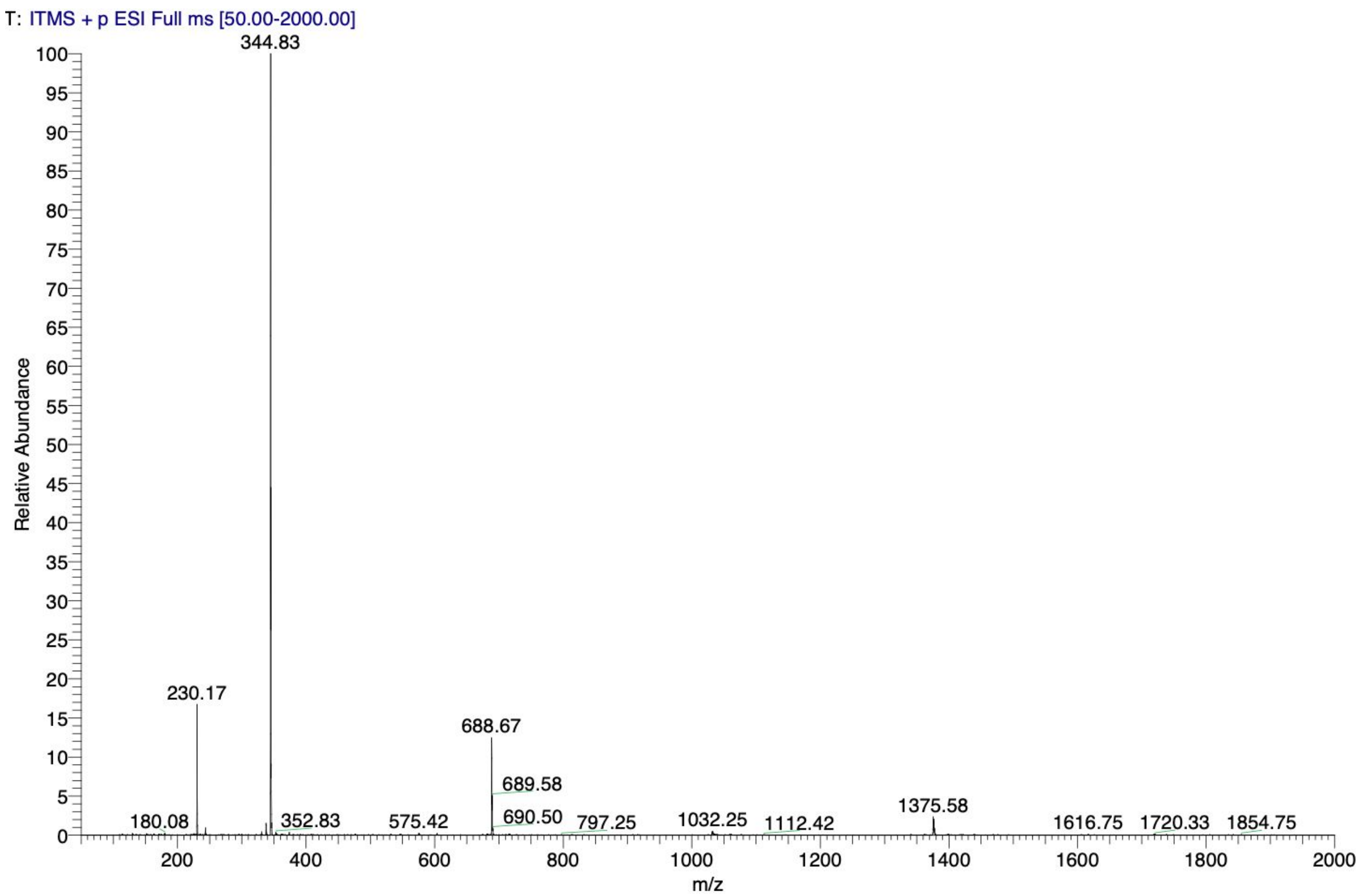

*Electrospray ionization mass spectrum (ESI-MS) of compound **24** recorded in positive ion mode.*

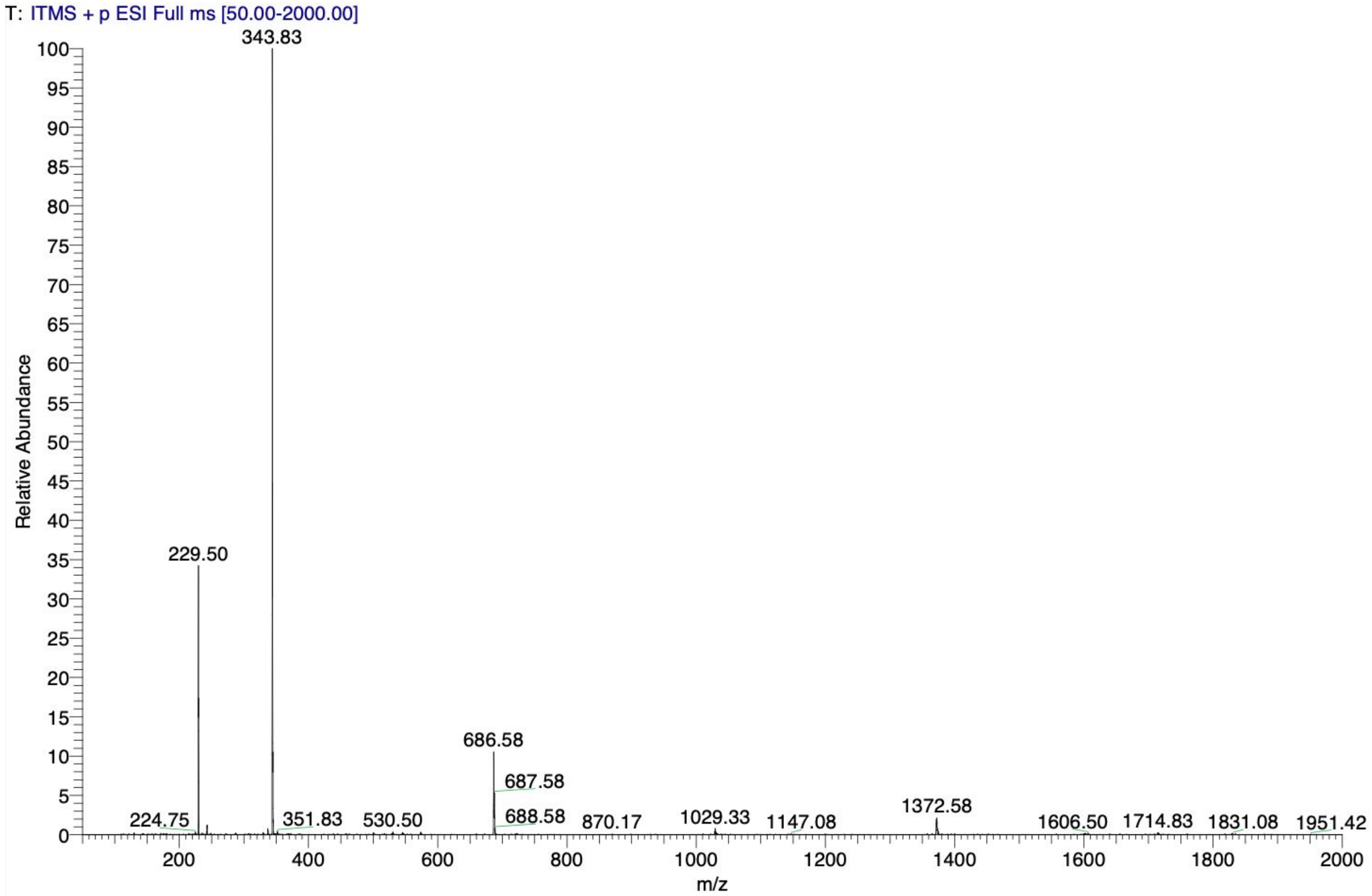

REFERENCES

(1) Ambrus, A.; Chen, D.; Dai, J.; Bialis, T.; Jones, R. A.; Yang, D. Human telomeric sequence forms a hybrid-type intramolecular G-quadruplex structure with mixed parallel/antiparallel strands in potassium solution. *Nucleic Acids Res* **2006**, *34* (9), 2723–2735. DOI: 10.1093/nar/gkl348.

(2) Butovskaya, E.; Heddi, B.; Bakalar, B.; Richter, S. N.; Phan, A. T. Major G-Quadruplex Form of HIV-1 LTR Reveals a (3 + 1) Folding Topology Containing a Stem-Loop. *J Am Chem Soc* **2018**, *140* (42), 13654–13662. DOI: 10.1021/jacs.8b05332.

(3) De Nicola, B.; Lech, C. J.; Heddi, B.; Regmi, S.; Frasson, I.; Perrone, R.; Richter, S. N.; Phan, A. T. Structure and possible function of a G-quadruplex in the long terminal repeat of the proviral HIV-1 genome. *Nucleic Acids Res* **2016**, *44* (13), 6442–6451. DOI: 10.1093/nar/gkw432.

(4) Monsen, R. C.; DeLeeuw, L.; Dean, W. L.; Gray, R. D.; Sabo, T. M.; Chakravarthy, S.; Chaires, J. B.; Trent, J. O. The hTERT core promoter forms three parallel G-quadruplexes. *Nucleic Acids Res* **2020**, *48* (10), 5720–5734. DOI: 10.1093/nar/gkaa107.
(5) Phan, A. T.; Modi, Y. S.; Patel, D. J. Propeller-Type Parallel-Stranded G-Quadruplexes in the Human c-myc Promoter. *J Am Chem Soc* **2004**, *126* (28), 8710–8716. DOI: 10.1021/ja048805k.
(6) Phan, A. T.; Kuryavyi, V.; Gaw, H. Y.; Patel, D. J. Small-molecule interaction with a five-guanine-tract G-quadruplex structure from the human MYC promoter. *Nat. Chem. Biol.* **2005**, *1* (3), 167–173. DOI: 10.1038/nchembio723.
(7) Li, S.; Cai, L.; Ji, H.; Yang, L.; Li, G. Pd(II)-catalysed meta-C–H functionalizations of benzoic acid derivatives. *Nat. Commun.* **2016**, *7* (1), 10443. DOI: 10.1038/ncomms10443.
(8) Feng, Y.-S.; Xie, C.-Q.; Qiao, W.-L.; Xu, H.-J. Palladium-Catalyzed Trifluoroethylation of Terminal Alkynes with 1,1,1-Trifluoro-2-iodoethane. *Org. Lett.* **2013**, *15* (4), 936–939. DOI: 10.1021/ol400099h.
